# Evolutionary and developmental analysis reveals *KANK* genes were co-opted for vertebrate vascular development

**DOI:** 10.1038/srep27816

**Published:** 2016-06-13

**Authors:** Monica R. Hensley, Zhibin Cui, Rhys F. M. Chua, Stefanie Simpson, Nicole L. Shammas, Jer-Yen Yang, Yuk Fai Leung, GuangJun Zhang

**Affiliations:** 1 Department of Comparative Pathobiology;.725 Harrison Street, West Lafayette, IN 47907, USA; 2 Purdue University Center for Cancer Research; 725 Harrison Street, West Lafayette, IN 47907, USA; 3 Department of Basic Medical Sciences; 725 Harrison Street, West Lafayette, IN 47907,USA; 4 Department of Biological Sciences, 725 Harrison Street, West Lafayette, IN 47907,USA; 5 Integrative Neuroscience Center; 725 Harrison Street, West Lafayette, IN 47907, USA; 6 Purdue Institute for Inflammation, Immunology and Infectious Diseases (PI4D), 725 Harrison Street, West Lafayette, IN 47907, USA

## Abstract

Gene co-option, usually after gene duplication, in the evolution of development is found to contribute to vertebrate morphological innovations, including the endothelium-based vascular system. Recently, a zebrafish *kank* gene was found expressed in the vascular vessel primordium, suggesting *KANK* genes are a component of the developmental tool kit for the vertebrate vascular system. However, how the *KANK* gene family is involved in vascular vessel development during evolution remains largely unknown. First, we analyzed the molecular evolution of the *KANK* genes in metazoan, and found that *KANK1*, *KANK2*, *KANK3* and *KANK4* emerged in the lineage of vertebrate, consistent with the two rounds of vertebrate whole-genome duplications (WGD). Moreover, *KANK* genes were further duplicated in teleosts through the bony-fish specific WGD, while only *kank1* and *kank4* duplicates were retained in some of the examined fish species. We also found all zebrafish *kank* genes, except *kank1b*, are primarily expressed during embryonic vascular development. Compared to invertebrate *KANK* gene expression in the central nervous system, the vascular expression of zebrafish *kank* genes suggested *KANK* genes were co-opted for vertebrate vascular development. Given the cellular roles of *KANK* genes, our results suggest that this co-option may facilitate the evolutionary origin of vertebrate vascular vessels.

One of the central goals of evolution is to understand the morphological complexity of living organisms such as vertebrates. This subphylum is defined by the presence of key morphological characteristics including neural crest cells and their derivatives such as craniofacial structures, neurogenic placodes, elaborate segmented brain, endoskeleton (bone and cartilage), and endothelium-based vasculature^1^,^2^,^3^. Decades of developmental genetic studies have revealed that such morphological structures are controlled by the genetic tool kit: developmental regulatory genes and tissue-specific genes^4^. Thus, the molecular phylogeny of such genes in the tool kit could provide us key information about the evolutionary origin of novel morphological structures. Whole genome duplications (WGD) were found as a way for increasing the diversity of genetic tool kits, and the vertebrate innovations were associated with the early WGD, around the origin of the subphylum^5^,^6^,^7^,^8^. WGD does not only increase the size of gene families, but also create novel functions of the duplicates (sub- or neo-functionalization) by co-option^9^,^10^,^11^,^12^. For example, we previously found WGD to contribute to the origin of vertebrate novelties such as cartilage by increasing the number of clade A collagen genes^13^,^14^,^15^.

The *KANK* protein family is characterized by an N-terminal KN motif, coiled-coil motifs, and four to five ankyrin repeat domains located in the C-terminus^16^. In *C. elegans, VAB-19*, the orthologous gene of *KANK*, is localized at the basement membrane and in trans-epidermal attachments^17^,^18^. VAB-19 is also required for later localization of attachment structures to muscle-adjacent regions of epidermis due to its role in actin remodeling mediated by Ras to Rac, key regulators of cell migration^19^. Fruit fly, also have a *KANK* gene, *CG10249*, which was found to be expressed in the midline of the central nervous system^20^. CG10249 is also expressed at the attachment region of muscle and epidermal cells in fruit fly embryos, and it interacted with EB1 at the microtubule plus end^20^,^21^. In human, there are four *KANK* genes: *KANK1*, *KANK2*, *KANK3* and *KANK4*^16^. *KANK1* was originally identified as a candidate tumor suppressor gene on chromosome 9p in renal cell carcinoma patients, and when overexpressed in renal cell lines it was found to inhibit cell growth^22^. In addition, *KANK1* functions as a regulator of actin polymerization, actin stress fiber formation, and cell migration through RhoA signaling^23^,^24^. Similar to *KANK1*, the other three human *KANK* genes were also found to be able to regulate actin polymerization and cell mobility^16^. An actin stress fiber is a bundle of approximately 10–30 actin filaments, and it plays important roles in cell migration and contractility of non-muscle vasculature cells. The stress fibers in these cells are important in dealing with mechanical stresses such as hydrostatic pressure, blood flow shear, and cyclic stretch^25^. Moreover, during vasculogenesis, endothelial cells undergo dramatic polarization, migration, rearrangements, and cell shape alterations^26^,^27^. All of the changes rely on cyctoskeleton and actin polymers which are critical for making the vascular capillary structures^26^,^27^. Recently, *kank3* was found expressed in the blood vessel primordium of zebrafish embryos^28^, suggesting *KANK* genes may play important roles in vertebrate blood vessel development. However, how the KANK gene family is involved in vascular vessel development as a component of the genetic tool kit during evolution remains largely unknown.

To explore this direction, we took advantage of the zebrafish model system, which has a complex closed endothelial circulatory system. Zebrafish vasculature anatomy, the vessel assembling process and the molecular regulatory mechanisms were found to be similar to humans^29^,^30^. We first analyzed the evolutionary history of the *KANK* gene family in major taxa of metazoan, and then examined expression patterns of zebrafish *kank* genes during early development. We found that the *KANK* gene family was expanded through WGD and the zebrafish *kank* genes were primarily expressed during vascular development. As invertebrate KANK genes are not expressed in vascular system during development, the vascular expression domains of zebrafish *kank* genes suggested that the *KANK* gene family is co-opted for vertebrate endothelial vascular development after vertebrate WGD around the separation of vertebrate subphylum.

## Results

### Vertebrates have four groups of *KANK* genes

**Figure 1:**
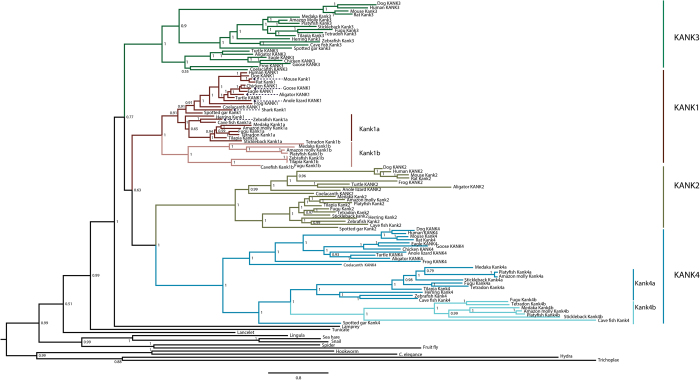
Extended majority-rule consensus tree for the Bayesian phylogenetic analysis of KANK proteins. Numbers at each node indicate posterior probability (pp) values based on twenty million runs. Branch lengths are proportional to means of the pp densities for their expected replacements per site. The ML phylogenetic tree (Fig. S1.) was generally in agreement with the BP phylogeny: all the invertebrates have one KANK, while there are four KANKs in vertebrates (KANK1-KANK4). There are extra Kank1 and Kank4 proteins in teleosts. The tunicate (urochordate) and lancelet (cephalochordate) form the closest outgroups of vertebrates. The tree was rooted with *Tricoplax* and *Hydra*.

To better understand how novel morphological characters can arise at the cellular and developmental level, it is important to learn the evolutionary history of the key genes of the related developmental tool kit. Because we were interested in a large span of evolutionary time, and DNA sequence most likely underwent multiple substitutions through this time, we chose KANK protein sequences from major metazoan taxa for phylogenetic analysis. We first retrieved KANK protein sequences from Ensembl and other genome databases by BLASTp search. Then multiple protein sequences were aligned using MUSCLE program^31^. We found that all examined KANK proteins possess both KN motifs at the N-terminus and ankyrin repeat domains at the C-terminus. Molecular phylogeny analyses were performed using Bayesian analysis (BP) and maximum likelihood (ML) methods^32^. We were able to trace back KANK to the basal metazoans such as *Trichoplax* and *Hydra* (Fig. 1 and Fig. S1). The invertebrate KANK proteins form a distinct group from the vertebrate KANKs, and the tunicate KANK forms the closest outgroup of vertebrate KANK proteins. The overall phylogenetic relationship of KANKs is consistent with our current knowledge on the phylogeny of metazoan taxa^33^,^34^.

In vertebrates, there are four distinct clades of KANK proteins, KANK1, KANK2, KANK3 and KANK4 (Fig. 1 and Fig. S1). This is consistent with the current knowledge that humans and mice have four *KANK* genes. The appearance of these four clades arose around the origin of the vertebrates, suggesting that the birth of the four KANK clades was one of the consequences of the consecutive WGD events^7^,^35^. Within each vertebrate KANK clade, sarcopterigii (lobe-finned fish) and actinoperigi (ray-finned fish) generally formed distinct groups. Interestingly, the lamprey KANK formed an outgroup of the gnathostomes in the BP phylogenetic tree (Fig. 1), while it was clustered within gnathostome KANK groups with low supporting bootstrap value (233/1000) in the ML phylogenetic tree (Fig. S1). This may be due to the fact that the lamprey genome might have undergone unusual independent genomic events, as evidenced by other lamprey genes such as collagens and *HOX* genes^36^,^37^.

### Loss of *KANK* genes in certain vertebrate taxa

Since it is known that some duplicated genes may be lost during evolution^38^, we examined the presence of the four KANK genes in the major taxa, and found that the *KANK2* gene is missing from the birds’ genomes (chicken, turkey *etc*.). To confirm this loss, we did a Blast search for *KANK2* orthologous genes in the bird genomes, however, no *KANK2* ortholog was found. In addition, we found that *KANK3* was missing from the current anole lizard genome, yet both *KANK2* and *KANK3* genes were identified in their relatives (turtle and crocodile) (Fig. 1 and Fig. S1). These data suggested the losses of *KANK2* and *KANK3* happened independently in the bird and lizard lineages, respectively. To further confirm these findings, we examined the evolutionarily conserved syntenies that contain these genes in the Synteny Database^39^ and Genomicus browser^40^. We found that the *KANK2* and *DOX6* genes are linked together in other jawed vertebrates, but these two genes are missing from current bird genomes. Similarly, *KANK3* is linked with *ANGPTL4*, *RPS28* and *NDUFA7A* in basal and higher vertebrates, but the synteny is missing from anole lizard genome.

### Duplicated *kank* genes in teleosts

**Figure 2:**
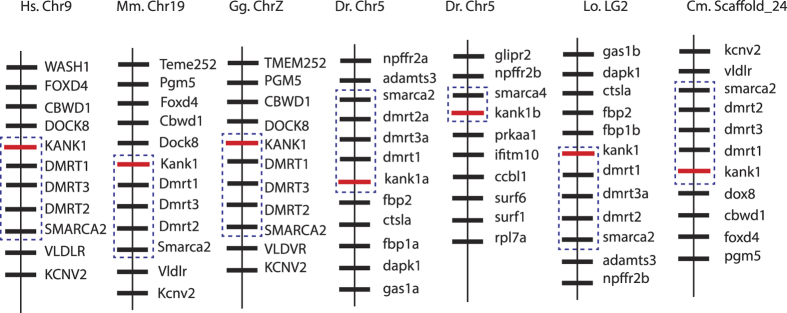
*KANK1* gene is located in an evolutionary conserved synteny in vertebrates. Seven representative vertebrate species were analyzed. The illustration of the genes and their sizes are not proportional to the length of the bars. *KANK1* is highlighted in red, and the synteny is highlighted in blue dashed-line boxes. The zebrafish *kank1a* is within the evolutionary conserved synteny. The zebrafish *kank1b* is distinct from the rest as it is only linked with *smarca4*. Hs, human; Mm, mouse; Gg, chicken; Dr, zebrafish; Lo, Spotted gar; Cm, elephant shark. Chr, chromosome. LG2, linkage group 2.

Bony fish are the most diverse group of species in the vertebrate subphylum. We found that of the four *kank* genes, *kank1* and *kank4* genes were duplicated in teleosts (Fig. 1 and Fig. S1). For the *KANK1* genes, *kank1a* is grouped with *KANK1* genes of tetrapod, spotted gar and elephant shark, rather than the teleost *kank1b*; suggesting that *kank1b* diverged from its ancestral state. Although widely used, phylogenetic analysis alone may not be sufficient for determining the gene orthologous relationships, especially in the unique situation of gene duplications followed by gene losses or rapid lineage-specific gene expansions^38^. To confirm the closer relationship between tetrapod *KANK1* and teleost *kank1a*, we did a syntenic analysis on the represented vertebrate *KANK1* genes. Interestingly, we found the *kank1a* in teleost are still in an evolutionarily conserved synteny, while *kank1b* has lost its neighboring genes and is only linked with the *smarca4* gene in zebrafish (Fig. 2). To further examine the relationships between the duplicated *kank1* genes in teleosts, we performed protein domain analysis using SMART program^41^. We found that both zebrafish Kank1a and Kank1b have similar protein domains when compared to human KANK1, except that Kank1b is 300aa shorter than Kank1a in the non-conserved middle region (Fig. S2). Furthermore, we analyzed the genomic intron-exon structural differences between zebrafish *kank1a* and *kank1b*. *Kank1a* has a similar intron-exon structure with human and spotted gar, while *kank1b* has a distinct structure with regard to the size and arrangement of exons (Fig. S3). Within the Kank4 clade, Kank4a and Kank4b clustered more closely compared to tetrapod *KANK4* genes and both clustered with spotted gar, indicating that the two groups of genes appeared at the same time within this lineage (Fig. 1 and Fig. S1). Interestingly, zebrafish and herring have only a single *kank4* gene, and they are located within the *kank4a* lineage suggesting that *kank4b* genes were lost in these two species.

### Zebrafish *kank* genes have similar yet distinct expression patterns

WGD usually leads to gene co-option, the functional specialization of paralogous genes, and contributes to vertebrate morphological innovations. Recently, *kank3* was found expressed in the blood vessel primordium of zebrafish embryos^28^, suggesting *KANK* genes may play important roles in vertebrate vascular development. As zebrafish are a widely used vertebrate model system in developmental biology, we reasoned that examining the gene expression patterns of the five zebrafish *kank* genes might shed light on the evolutionary origin of the vertebrate vascular system.

#### Expression of *kank1a* and *kank1b*

**Figure 3:**
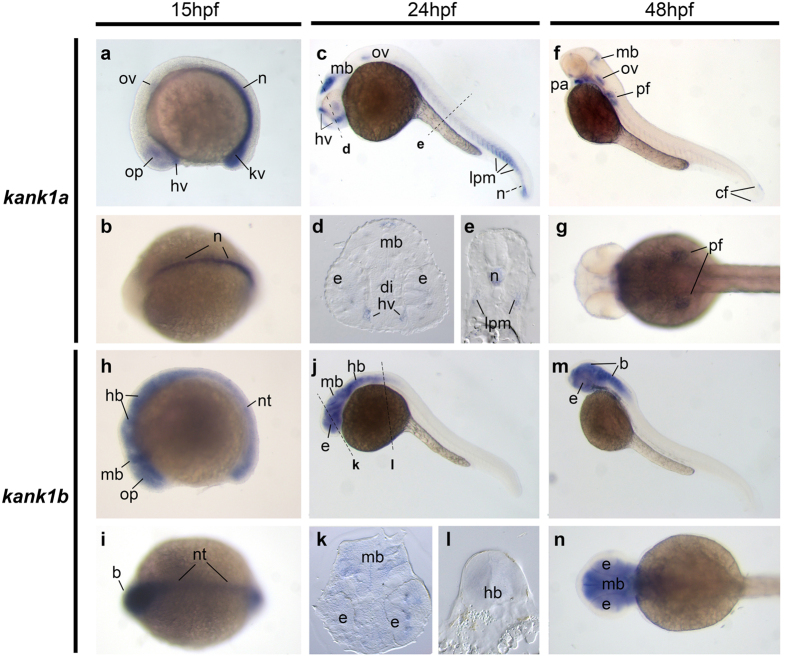
Gene expression patterns of *kank1a* and *kank1b* in zebrafish embryos. Whole-mount *in situ* hybridization of zebrafish embryos at stages 15 hpf (**a**,**b**,**h** and **i**), 24 hpf (**c**–**e**,**j**–**l**) and 48 hpf (**f**,**g**,**m**,**n**). Anterior is to the left in all the whole-mount images, and dorsal is to the top in all transverse sections. (**a**,**b**). Lateral and dorsal view of the expression of *kank1a* at 15 hpf. (**c**,**f**). Lateral view of *kank1a* expression at 24 hpf and 48 hpf, respectively. (**h**,**i)**. Lateral and dorsal view of the expression of *kank1b* at 15 hpf. (**j**,**m**). Lateral view of kank1b expression at 24 hpf and 48 hpf, respectively. The dashed lines indicate the positions of sections. The letters below the dashed lines correspond to the panels. *b*, brain; *cf*, caudal fin bud; *di*, diencephalon; *e*, eye; *hb*, hindbrain; *hv*, head blood vessels; *kv*, Kupffer’s vesicle; *lpm*, lateral plate mesoderm; *mb*, midbrain; *n*, notochord; *nt*, neural tube; *op*, optic vesicle; *ov*, otic vesicle; *pa*, pharyngeal arch; *pf*, pectoral fin.

Zebrafish have two *kank1* genes, *kank1a* and *kank1b*. At the developmental stage 15 hpf (hours post fertilization), *kank1a* is expressed in the optic vesicles, otic vesicles, head blood vessels, notochord and Kupffer’s vesicle (Fig. 3a,b), while *kank1b* is expressed throughout the central nervous system (Fig. 3h,i). By 24 hpf, *kank1a* is expressed within distinct patches of the midbrain, head blood vessels, otic vesicles, notochord, and lateral plate mesoderm, which gives rise to trunk intersegmental vessels (Fig. 3c–e). In contrast to *kank1a’s* distinct expression patterns, *kank1b* is expressed broadly throughout the midbrain, hindbrain and eyes at 24 hpf (Fig. 3j–l), with this expression pattern continued to 48 hpf (Fig. 3m,n). By 48 hpf, *kank1a* expression in the notochord and somites has decreased anteriorly to posteriorly. In addition, while *kank1a* is still expressed in the midbrain and otic vesicle, at 48 hpf, there is additional expression in the pharyngeal arch, pectoral and caudal fin buds (Fig. 3f,g).

#### Expression of *kank2*, *kank3*, and *kank4*

**Figure 4:**
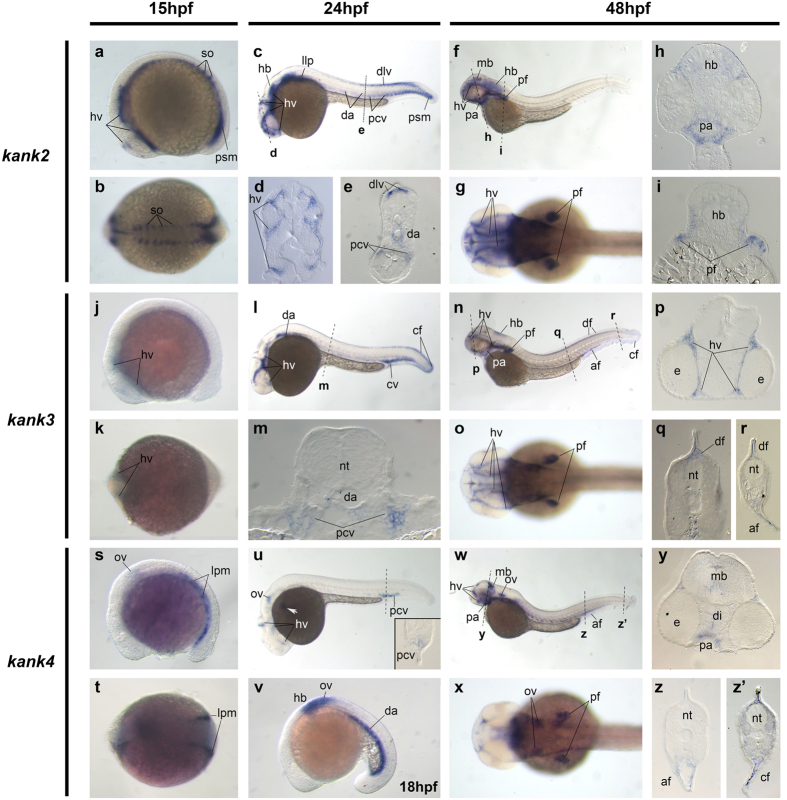
*Kank2*, *kank3* and *kank4* gene expression during zebrafish development. Whole mount *in situ* hybridization of zebrafish embryos at stages 15 hpf (**a**,**b**,**j**,**k**,**s**,**t**), 18 hpf (**v**), 24 hpf (**c**–**e**,**l**,**m**,**u**) and 48 hpf (**f**–**i**,**n**–**q**,**w**–**z’**). Anterior is to the left in all whole-mount images, and dorsal is to the top in all transverse sections. (**a**–**i**). Gene expression of *kank2*. (**j**–**r**). Gene expression of *kank3*. (**s**–**z’**). Gene expression of *kank4*. The dashed lines indicate the positions of section. The letters below the dashed lines correspond to the panels. The inset within panel (**u)** is the transverse section of the dashed line above. The white arrow points to the *kank4* expression on the vascular vessels on the surface of the yolk. *af*, anal fin bud; *cf*, caudal fin bud; *cv*, caudal vein; *da*, dorsal aorta; *df*, dorsal fin bud; *dlv*, dorsal longitudinal vein; *e*, eye; *hb*, hindbrain; *hv*, head vessels; *llp*, lateral line premordia; psm, presomatic mesoderm; *lpm*, lateral plate mesoderm; *mb*, midbrain; *nt*, neural tube; *ov*, otic vesicle; *pa*, pharyngeal arch; *pcv*, posterior cardinal vein; *pf*, pectoral fin bud; *so*, somite.

Next, we examined *kank2* gene expression, which is strongly expressed in the head blood vessels, somites and presomitic mesoderm of the tail bud at 15 hpf (Fig. 4a,b). At 24 hpf, it is expressed in the head blood vessels, midbrain, and lateral line primordia, with expanding expression from the presomatic mesoderm to the trunk blood vessels (Fig. 4c–e). By 48 hpf, *kank2* expression is restricted to the head region with retained expression in the head blood vessels, midbrain and hindbrain. New expression was detected in the pharyngeal arches, as well as the pectoral and annual fin buds (Fig. 4f–i). *Kank3* has limited expression in the head blood vessel at 15 hpf (Fig. 4j,k). By 24 hpf, *kank3* expression in the head blood vessels remains, with additional expression in the dorsal aorta, caudal vein, and caudal fin bud (Fig. 4l,m). *Kank3* retains this expression pattern at 48 hpf (Fig. 4n–r). Our *kank3* expression results are consistent with previous reports that this gene is expressed in the head blood vessels during zebrafish development^28^. Lastly, at 15 hpf, *kank4* is expressed in the otic vesicles and lateral plate mesoderm, which gives rise to angioblasts in zebrafish (Fig. 4s,t). When the embryo is at 18 hpf, *kank4* expression is also found in the hindbrain and dorsal aorta (Fig. 4v). The dorsal aorta expression of *kank4* is greatly reduced anteriorly to posteriorly by 24 hpf, where its expression is also seen in the head blood vessels, otic vesicle and in the posterior cardinal veins (Fig. 4u). At 48 hpf, *kank4* is still expressed in the otic vesicle and head blood vessels, with additional expression in the midbrain, pharyngeal arch, anal and caudal fin (Fig. 4w–z’).

## Discussion

Understanding the morphological complexity of living organisms such as vertebrates is one of the central questions for evolutionary biology. The separation of the vertebrate lineage was accompanied with key morphological characteristics, one of which was the endothelium-based vascular system. Such morphological innovations are usually generated through tinkering or modification of the corresponding developmental genetic tool kits. Thus, the evolution and gene expression of key genes in such genetic tool kits provide us important information for understanding the evolutionary origin of morphological novelties. Here, we first analyzed the evolution of the *KANK* gene family and then examined the zebrafish *kank* gene expression patterns during early development. Our results suggested *KANK* genes were co-opted for vertebrate vascular development after vertebrate WGD, and *KANK* gene duplication and diversification may facilitate the evolutionary origin of vertebrate vascular vessels.

### Evolutionary history of *KANK* genes

The *KANK* genes form an evolutionary conserved gene family. We were able to track their evolution as far back as basal metazoans such as placozoa and cnidarian. In the invertebrate species we analyzed, there is only one *KANK* gene in each species, while there are four *KANK* genes (*KANK1*-*KANK4*) in vertebrates. The expansion of the four *KANK* gene clades coincides with the origin of vertebrates. As there are two consecutive WGD (2R) in the vertebrate common ancestors, the four *KANK* genes are most likely the result of these whole genome duplications^5^,^6^,^7^,^8^. In addition, we found duplicates of *kank1* and *kank4* in teleosts. This is consistent with the bony fish specific WGD (3R)^42^,^43^. The duplicated paralogous genes through WGD (ohnolog) can be lost distinctively in different species, and only about one third of duplicated genes are retained in the zebrafish genome^38^,^44^. For example, zebrafish have only one *kank4*, while other fish such as fugu, amazon molly, platyfish, *etc*. have two *kank4* genes (*kank4a* and *kank4b*).

We did not find any duplicates of *kank2* or *kank3* in the bony fish species we analyzed, suggesting that one of the ohnologs of *kank2* and *kank3* were lost after the teleost WGD. To our surprise, we did not find any *KANK2* genes in the currently sequenced representative bird genomes. However, this gene was found in lizard, turtles and crocodiles, indicating that the *KANK2* gene was lost specifically in the lineage of birds. Currently, *KANK2* is known for its functions as a steroid receptor coactivator, inhibitor of apoptosis and regulator of actin dynamics in kidney podocyte foot processes^45^,^46^,^47^. The biological consequences of *KANK2* gene loss in birds are currently unknown.

**Figure 5:**
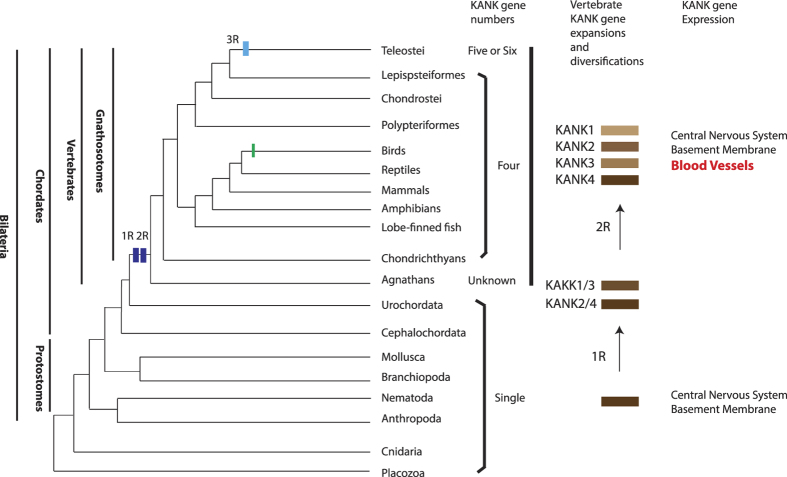
The molecular evolution of *KANK* genes and the origin of vertebrate blood vessels. The phylogenetic relationships of bilateria and vertebrata are based on the recent phylogenomic analyses and our past collagen gene phylogenetic analyses. The branch lengths are not proportional to the time of diversification. The two vertical dark blue bars on the tree indicate the WGD events (R1 and R2) at the origin of the vertebrates. The other light blue vertical bar represents the teleost specific WGD. The green bar on the bird lineage indicates the loss of *KANK2*. The process of *KANK* gene duplication and diversification through WGD is illustrated by the brown bars. The co-option of the vertebrate four *KANK* genes’ expression coincides with the vertebrate vascular vessels.

Based on our analysis on *KANK* genes’ evolution and our current knowledge of the WGD, we propose a model for *KANK* gene evolution, as shown in Fig. 5. Briefly, an invertebrate *KANK* gene was duplicated twice through the 2 consecutive WGD (2R) and created the vertebrate *KANK1*-*KANK4*. In teleost, all the *kank1*-*kank4* were further duplicated through bony fish specific WGD (3R), and one of the duplicates of *kank2* and *kank3* were lost, and only one copy of each of these two genes retained in examined current teleost species. Similarly, in birds, *KANK2* may have been lost independently after the separation of the bird lineage.

### Overlapping and distinct expression patterns of zebrafish *kank* genes

Gene expression usually corresponds to gene’s functions during embryonic development. We have found that zebrafish *kank1a*, *kank1b* and *kank4* are expressed in the central nerve system, while *kank2*, *kank3*, and *kank4* are mainly expressed in the blood vessel primordium. Additionally, *kank1a*, *kank2*, *kank3*, and *kank4* are also expressed in the paired and median fin folds. These expression domains suggest a developmental function for *kank* genes in these tissue and organs. These zebrafish *kank* genes’ overlapping and distinct expression domains most likely resulted from WGD and subsequent gene-coption: sub-functionalization and neo-functionalization^9^. After gene duplication, the two duplicates may simply split the original functions of ancestral gene, leading to sub-functionalization. Or, one gene duplicate may retain the original function(s) of the ancestral gene, while the other duplicated gene may gain new expression domain(s) and evolve new functions, leading to neo-functionalization. Usually gene co-options were caused by changes in the gene regulatory regions after gene duplication, as coding regions are generally subject to more functional constrains^11^. Consistent to this, we found all the human and zebrafish KANK genes share very similar protein structures (Fig. S2), and all four human KANK genes have been reported to have the same functions in regulation of cytoskeletons^16^.

### *KANK* genes were co-opted for vascular vessel development in vertebrates

The sub-functionalization and neo-functionalization of developmental genes usually lead to gene co-options for new developmental expression domains that are related to morphological novelties^9^,^10^,^11^,^12^. One of the vertebrate innovations is the blood vascular system that is essential for transportation of oxygen, nutrition, and waste products^3^,^30^. Compared to invertebrates, the major difference is the endothelial lining which makes the blood circulation more efficient^3^,^48^,^49^. Because blood vessels do not fossilize as do skeletons, it is unrealistic to trace the evolution of the circulatory system using fossils. Thus, molecular evolution and developmental studies are important to understand the evolutionary origin of this system.

Recently, the expression of zebrafish *kank3* was reported in the blood vessel primordium^28^, suggesting the *KANK* genes are a component of the genetic tool kit for vertebrate blood vessel development. The fruit fly *KANK* gene was found expressed in the central nervous system^20^, where we also found zebrafish *kank1a*, *kank1b* and *kank4* to be expressed. This suggests that one of the primitive functions of *KANK* genes is regulating development of the central nervous system. The new expression domain of *kank* genes (*kank1a*, *kank2*, *kank3* and *kank4*) in zebrafish blood vessel primordium revealed that the *KANK* genes were co-opted for vascular development after the two rounds of vertebrate WGDs (Fig. 5). Given the regulatory functions of *KANK* genes in cytoskeleton and cell migration^16^,^23^,^24^, and how vasculogenesis requires extensive cell shape change and movement, the co-option of *KANK* genes from neural development to vascular development might facilitate the evolutionary origin of the vertebrate vascular system. Interestingly, the similarities of neural and vascular development have long been noticed since both processes utilize very similar molecular regulatory networks, such as VEGF and Ephrin–Eph signaling^49^,^50^. Alternatively, the zebrafish *kank* genes only express specifically in blood vessels of fish. This is unlikely since most orthologous genes in zebrafish express in similar locations during development compared with that in tetrapod^51^,^52^,^53^. These include *vegf* and other vascular regulatory genes such as *vecdn*, *estrp* and *fli1*^29^,^30^. It is even more unlikely that all five *kank* ohnologs are fish specific. Future studies should define *KANK*s’ role in the evolution of blood vessels in vertebrates by determining the expression and functions of *KANK*s in urochordates, cephalochordates, agnathans, chondrichthyans, and tetrapods.

## Methods

### Zebrafish strains and husbandry

Zebrafish were raised and maintained following the procedures described in the zebrafish book^54^. The Purdue animal housing facility is an AAALAC-approved animal facility and all experiments were carried out according to the protocols approved by the Purdue Animal Care and Use Committee (*PACUC*) (Protocol # 1210000750). The wild type line used in this study is of the TAB background. The zebrafish embryos were sorted and staged using the Kimmel’s staging guide^55^.

### KANK protein sequence retrieval and analysis

KANK protein sequences were identified by a BLASTp search using human KANK1 sequences as a query. The lamprey KANK sequence was retrieved from the Japanese lamprey genome website (http://jlampreygenome.imcb.a-star.edu.sg), and the lancelet KANK sequence was retrieved from the Chinese lancelet genome website (http://mosas.sysu.edu.cn/genome/index.php). The rest of the KANK protein sequences of the representative’s metazoan taxa were retrieved from either Ensembl or NCBI^56^,^57^ (Table S1). The longest sequence was preferentially chosen when there were multiple sequences. Multiple protein sequences were aligned using MUSLE program^31^, and the FASTA format alignment can be found in the supplementary File 1. To identify the best evolutionary model for phylogenetic analysis, we carried out a best model test using maximum likelihood and default parameters in MEGA6^58^. The models with lowest BIC (Bayesian Information Criterion) scores were considered to describe the substitution pattern the best, and JTT+G was chosen. Then, we constructed phylogenetic trees using Bayesian analysis (BP) and maximum likelihood (ML) methods that are currently the most reliable for inferring phylogeny^32^. The ML and BP analyses were conducted as described using PhyML 3.1 and MrBayes 3.2.6, respectively^59^,^60^. For BP phylogenetic analysis, 20 million generations were run using the following parameters: nruns = 2, nchains = 4, aamodel = fixed(Jones), rates = gamma ngammacat = 8, samplefreq = 500, burninfrac = 0.25. ML phylogenetic analysis was performed using JTT + G with 1000 bootstrap replicates. The final phylogenetic trees were viewed and generated with FigTree V1.4.2 (http://tree.bio.ed.ac.uk/software/figtree). Gene intron-exon structures were analyzed using the longest transcripts in Ensembl. The synteny of *KANK* genes were analyzed using Ensembl, UCSC genome browser, Synteny Database^39^ and Genomicus browser^40^. Protein domain analysis was carried out using the SMART online tool^41^.

### Gene cloning, in situ hybridization and imaging

Full-length coding regions of zebrafish *kank* genes were amplified by RT-PCR using gene specific primers designed according to the current DNA sequence in Ensembl. One-three day old zebrafish embryos were mixed for total RNA isolation using TRIzol reagent (Thermo Fisher) and reverse transcription was performed using SuperScript® III First-Strand Synthesis System (Thermo Fisher) following the manual’s instructions. Phusion® High-Fidelity DNA Polymerase master mix (New England Biolabs) was used for PCR amplification. PCR primers used here are: *kank1a*: 5′ATGGCCCAAACCATGCACATTAACG 3′ and 5′GACAGAACCTGAAGGAATAGCAGCTC 3′; *kank1b*: 5′ATGACTCAAGAAACGCACTTCCACC 3′, 5′CCATATTCGTCGTGTGATTCAACACC3′; *kank2*: 5′ATGACAATCATGGCTCAGGTGCT 3′, 5′CTTCAGTTCAGAGGACGAAGGAGGA 3′; *kank3*: 5′ATGACCCAATCTGTGCACATAACCT 3′, 5′TTTCTCCGAGGGCCATGTTTTTCTA 3′; *kank4*: 5′ATGGACAAGAAAAGTGCAAATGGCT 3′, 5′GAGCGGGGCCGCTGAATCT 3′. The PCR products were purified using GeneJET Gel Extraction Kit (Thermo Scientific) before they were cloned into pJet1.2 vectors using the CloneJET PCR Cloning Kit (Thermo Scientific). Orientations of gene inserts were verified by Sanger sequencing. Riboprobes were synthesized through *in vitro* transcription using T7 DNA polymerase (New England Biolabs) and DIG RNA Labeling Mix (Roche, 11277073910). Then the riboprobes were purified by SigmaSpin^TM^ post-reaction clean-up columns (Sigma, S5059). Whole mount i*n situ* hybridization was carried out according to previously published method^61^ with the following modification for proteinase K treatment: 8–18 hours embryos, no treatment; 24 hours embryos, 5 minutes; 48 hours embryos for 30 minutes at room temperature. The riboprobe hybridization was carried out at 65 °C. For histological analysis, post-hybridization embryos were equilibrated in 15% sucrose then 30% sucrose in 20% gelatin, after which they were embedded in 20% gelatin for cryosectioning (6–12 μm). Images were acquired using AxioCam MRc camera on Zeiss SteREO Discovery.V12 and Axio Imager 2 compound microscope.

## Additional Information

**How to cite this article**: Hensley, M. R. *et al*. Evolutionary and developmental analysis reveals *KANK* genes were co-opted for vertebrate vascular development. *Sci. Rep*. **6**, 27816; doi: 10.1038/srep27816 (2016).

## Supplementary Material

Supplementary Information
