# Supplementary material for: Evolutionary and developmental analysis reveals KANK genes were co-opted for vertebrate vascular development
Source: Sci Rep. 2016 Jun 13;6:27816. doi: 10.1038/srep27816 (PMC4904190; doi:10.1038/srep27816)
Supplement: Supplementary Information [file srep27816-s1.pdf]

# Evolutionary and developmental analysis reveals *KANK* genes were co-opted for vertebrate vascular development

Monica R. Hensley[1]*, Zhibin Cui[1]*, Rhys F.M. Chua[1], Stefanie Simpson[1], Nicole L. Shammas[1], Jer-Yen Yang[2,3], Yuk Fai Leung[4,5] GuangJun Zhang[1,2,5,6#]

## Supplementary Information

## <u>Supplementary Figures</u>

**Figure S1. Maximum likelihood phylogeny of KANK proteins, as obtained with JTT plus gamma distances.** Numbers around each node indicate bootstrap values based on 1,000 replicates. Branch lengths are proportional to expected replacements per site. Consistent with BP phylogeny, the vertebrate KANKs forms four groups. The tree was rooted with *Tricoplax* and *Hydra*.

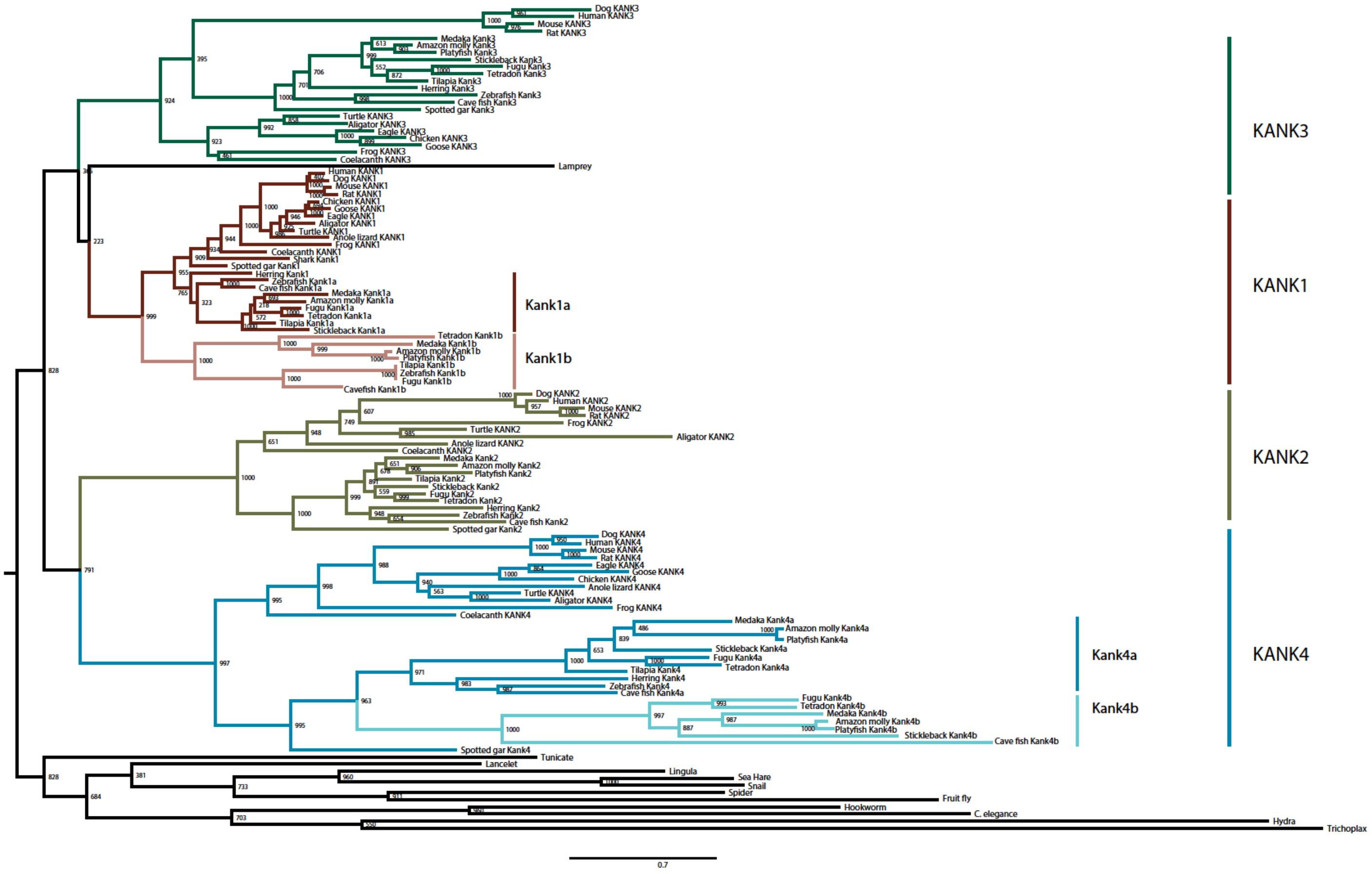

**Figure S2. Comparison of the KANK protein domains of human and zebrafish.** (**a**). Human KANK protein domains. (**b**). Zebrafish Kank protein domains. Purple bars indicate low compositional complexity regions. Green bars represent coiled-coil regions. Vertical colored thin lines show the intron positions. KN motif and ankyrin repeats are shown in square boxes with “KN-motif” and “ANK” respectively. A ruler with amino acid numbers is below each protein diagram.

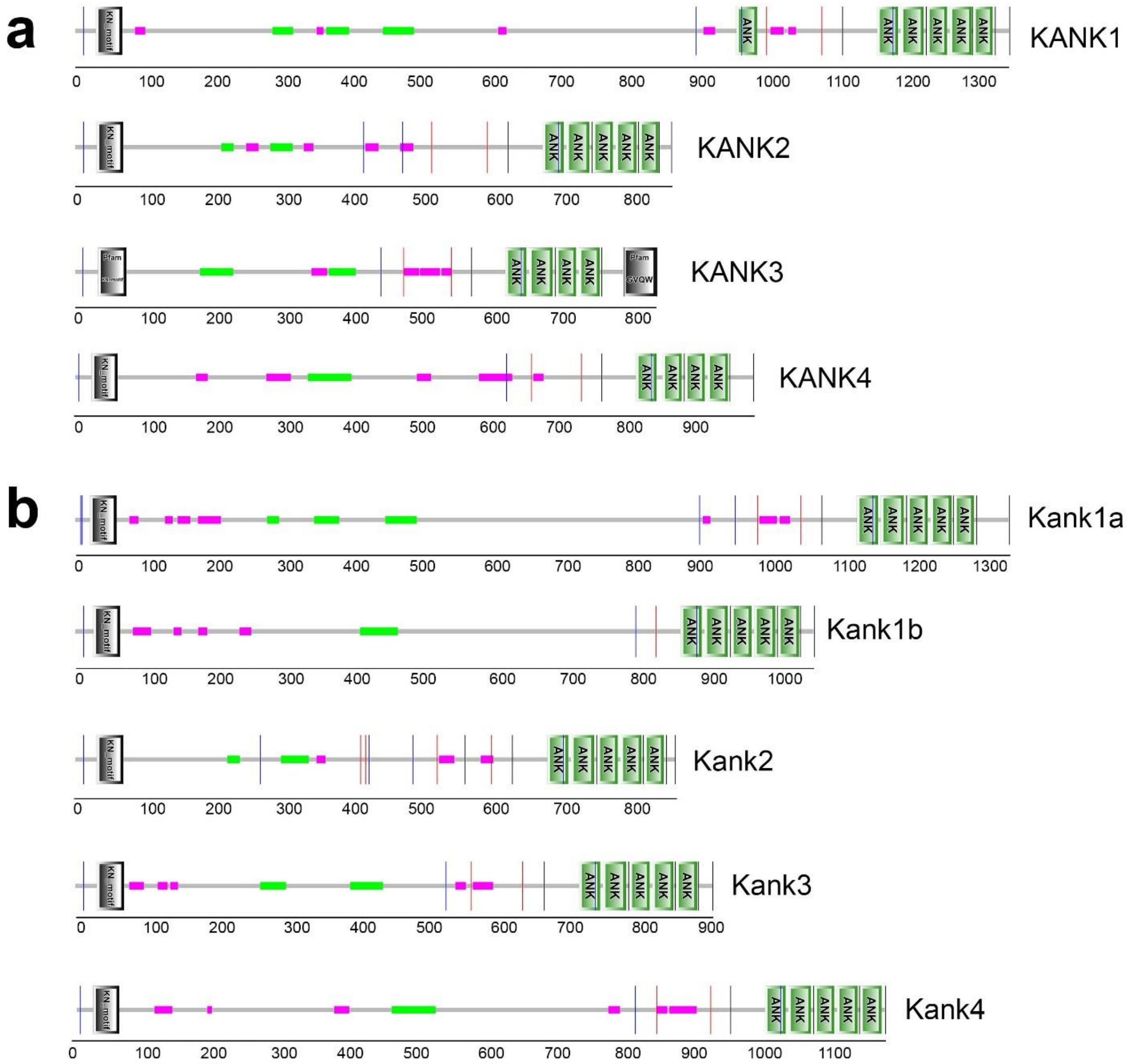

**Figure S3. Zebrafish *kank1a* and *kank1b* have different intron-exon structures.** These gene structures were retrieved from Ensemble using the longest transcripts. (**a**) Spotted gar *kank1*, Transcript: *kank1*-201 ENSLOCT00000014107. (**b**). Human *KANK1*, Transcript: *KANK1*-001 ENST00000382303. (**c**). Zebrafish *kank1a*, *kank1*-001 ENSDART00000136521. (**d**). Zebrafish *kank1b*, Transcript: *KANK1* (2 of 2)-201 ENSDART00000085512. The solid red boxes represent the gene coding regions, and the empty boxes indicate untranscribed regions. Red lines indicate the introns.

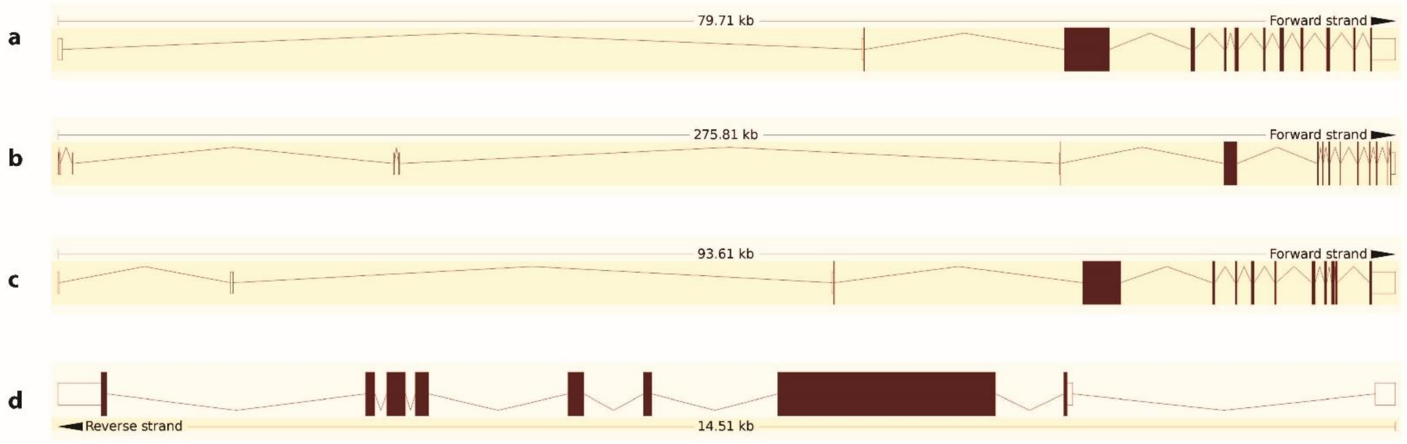

## **Supplementary Table**

## Table S1. The sources of KANK protein sequences used for the phylogenetic analyses.

| Common name | Scientific name | KANK1 or Kank1a | Kank1b | KANK2 | KANK3 | KANK4 or Kank4a | Kank4b |
|---|---|---|---|---|---|---|---|
| Alligator | *Alligator sinensis* | XP_006022667.1 | | XP_006023540.1 | XP_014376372.1 | XP_006027522.1 | |
| Amazon molly | *Poecilia formosa* | kank1-201 ENSPFOT00000001152 | si:dkey-226m8.9-201 ENSPFOT00000010906 | kank2-201 ENSPFOT00000029317 | kank3-201 ENSPFOT00000000137 | a: kank4-201 ENSPFOT00000006984 | b: KANK4 (2 of 2)-201 ENSPFOT00000023801 |
| Anole lizard | *Anolis carolinensis* | XP_008101527.1 | | XP_008101990.1 | | XP_008107551.1 | |
| Atlantic herring | *Clupea harengus* | XP_012684054.1 | | XP_012689245.1 | XP_012695057.1 | XP_012697624.1 | |
| C. elegans | *Caenorhabditis elegans* | T22D2.1 | | | | | |
| Cave fish | *Astyanax mexicanus* | kank1-201 ENSAMXT00000012876 | XP_007257032.1 OR si:dkey-226m8.9-201 ENSAMXT00000012956 | XP_007238267.1 | kank3-201 ENSAMXT00000014761 | kank4-201 ENSAMXT00000010815 | XP_007250784.1 |
| Chick | *Gallus gallus* | KANK1-201 ENSGALT00000016515 | | | KANK3-201 ENSGALT00000041280 | XP_422516 | |
| Coelacanth | *Latimeria chalumnae* | XP_006001260 | | XP_005994226.1 | XP_005989925.1 | XP_014352296.1 | |
| Dog | *Canis lupus familiaris* | KANK1-201 ENSCAFT00000003130 | | KANK2-201 ENSCAFT00000027519 | KANK3-202 ENSCAFT00000029379 | KANK4-201 ENSCAFT00000049528 | |
| Frog | *Xenopus tropicalis* | ANKRD15-201 ENSXETT00000004865 | | XP_002942889.2 | XP_002938597 | XP_002931658 | |
| Fugu | *Takifugu rubripes* | kank1-203 ENSTRUT00000040327 | si:dkey-226m8.9-201 ENSTRUT00000047389 | kank2-202 ENSTRUT00000028247 | kank3-202 ENSTRUT00000018377 | kank4-202 ENSTRUT00000047507 | b: XP_011614899.1 |
| Fruit fly | *Drosophila melanogaster* | CG10249 FBgn0027596 | | | | | |
| Elephant shark | *Callorhinchus milii* | NCBI AFO94041 | | | | | |
| Golden eagle | *Aquila chrysaetos* | XP_011588687.1 | | | XP_011591736.1 | XP_011570405.1 | |
| Hookworm | *Ancylostoma ceylanicum* | EYC07694.1 | | | | | |
| Human | *Homo sapiens* | KANK1-201 ENST00000619269 | | KANK2-003 ENST00000589359 | KANK3-002 ENST00000593649 | KANK4-001 ENST00000371153 | |
| Hydra | *Hydra vulgaris* | XP_012562942.1 | | | | | |
| Lamprey | *Lethenteron japonicum* | JL1710 unnamed protein product 1-727 (http://jlampreygenome.imcb.a-star.edu.sg ) | | | | | |
| Lancelet | *Branchiostoma belcheri* | BbP_088960R (http://mosas.sysu.edu.cn/genome/index.php) | | | | | |

| | | | | | | | |
|---|---|---|---|---|---|---|---|
| Lingula | *Lingula anatina* | XP_013392739.1 | | | | | |
| Medaka | *Oryzias latipes* | XP_011471394.1 | XP_011479791.1 | KIAA0172-201 ENSORLT00000006722 | XP_011472324.1 | a: kank4-201 ENSORLT00000013200 | XP_011484789.1 |
| Mouse | *Mus musculus* | Kank1-001 ENSMUST00000049400 | | Kank2-201 ENSMUST00000034717 | Kank3-001 ENSMUST00000048560 | Kank4-001 ENSMUST00000102790 | |
| Painted turtle | *Chrysemys picta* | XP_005296814.1 | | XP_008173664.1 | XP_005283841.1 | XP_008166090.1 | |
| Platyfish | *Xiphophorus maculatus* | | XP_014327830.1 | kank2-201 ENSXMAT00000000194 | kank3-201 ENSXMAT00000002486 | a: XP_014326532.1 | b: XP_014328965.1 |
| Rat | *Rattus norvegicus* | Kank1-201 ENSRNOT00000021496 | | Kank2-201 ENSRNOT00000014135 | Kank3-201 ENSRNOT00000009532 | Kank4-201 ENSRNOT00000066139 | |
| Sea hare | *Aplysia californica* | XP_005108295.2 | | | | | |
| Spider | *Stegodyphus mimosarum* | KFM78514.1 | | | | | |
| Snail | *Biomphalaria glabrata* | XP_013078178.1 | | | | | |
| Spotted gar | *Lepisosteus oculatus* | kank1-201 ENSLOCT00000014107 | | XP_006631656.1 | kank3-201 ENSLOCT00000003117 | kank4-201 ENSLOCT00000007896 | |
| Stickleback | *Gasterosteus aculeatus* | kank1-201 ENSGACT00000019197 | | kank2-201 ENSGACT00000018346 | kank3-201 ENSGACT00000010886 | a: kank4-201 ENSGACT00000009094 | b: KANK4 (2 of 2)-201 ENSGACT00000021428 |
| Swan goose | *Anser cygnoides* | XP_013047600.1 | | | XP_013051691.1 | XP_013031592.1 | |
| Tetradon | *Tetraodon nigroviridis* | kank1-202 ENSTNIT00000001422 | KANK1 (1 of 2)-201 ENSTNIT00000010479 | kank2-201 ENSTNIT00000021984 | kank3-201 ENSTNIT00000013290 | a: KANK4 (1 of 2)-201 ENSTNIT00000010852 | kank4-201 ENSTNIT00000021496 |
| Tilapia | *Oreochromis niloticus* | kank1-201 ENSONIT00000017876 | si:dkey-226m8.9-201 ENSONIT00000014023 | kank2-201 ENSONIT00000001905 | kank3-201 ENSONIT00000015998 | kank4-201 ENSONIT00000011121 | |
| Trichoplax | *Trichoplax adhaerens* | XP_002114910.1 OR TriadT58901 | | | | | |
| Tunicate | *Ciona intestinalis* | ENSCINT00000023213 | | | | | |
| Zebrafish | *Danio rerio* | kank1-001 ENSDART00000136521 | si:dkey-226m8.9-201 ENSDART00000085512 | kank2-001 ENSDART00000154031 | kank3-201 ENSDART00000115050 | kank4-001 ENSDART00000155790 | |

# Supplementary File

## Supplementary File 1: KANK protein alignment

```
                             10        20        30        40        50        60        70        80        90       100
                     ....|....|....|....|....|....|....|....|....|....|....|....|....|....|....|....|....|....|....|....|
Hydra                ----------------------------------MLEKKR-------------QQLNEKKEFSEPLNVLRKREKFSFSPIYLSVVGFNQRADK-------
Trichoplax           ----------------------------------MAPN-----------------------------------------EYNQMINDKTELLIHFAK---
Aplasia              --------------------------------MASTKVTFVTGTAEVRRRDSSRSGYPEEPQQALTTLPNAPSQVVFVDGKCSCCPYGYHIDLDFLN---
Snail                ---------------------------------MATSKVTFVTGTAEVKRRDPQGLNSLHPDQHLTTEPQKPLQVTYVDGKCTCCPYGYHIDLDFLN---
Fruitfly             MSVRPNNAGDMLLHNSVIGGYDESKFAEKITHLTPTEQEAVKRFWRSLVLRSQRAFIEENARPIASSANPAPAGITRSVRSCNCCPYGYHIDLDFVR---
Hookworm             -------------------------------MLIDFSATQILQKI--------DIPDAPPKPPHRSTTNGRATP-------SKQHNSGPRTEFASLRRGR
C.elegance           ---------------MRDIGGSMSSASTSATIVKRSPPNDTSTSSGVSCDVSPNSMTSPSSGSDQNQNNNSL-------NKCKCCPYGYHIDLGFVS---
Tunicate             ---------MEFRKLKGLWKNGGTKPETPIFHENCTLTSHAEKTNSNTNNSNSNRNMQDKASTNRLESLEEIRPEVTAEGYGLRTPYGTHLNLDFVK---
Spider               ----------------------------------MPEK---------------KSNSDSKMPS----------------SQCGCCPYGFHIDRDFVR---
Lingula              ----------------------------------MTSSSTLVQQ---------QQTMDPTPQGQPVEG-----------GRCSCCPYGYHIDLDFVR---
Lancelet             --------------------------------MRKATSAVDYANMRDKDVEKPADAESGFGDMESDTPEANPKVDLSPDGKCTCCPYGYHIDLDFLK---
Medaka Kank4b        ----------------------------------MAEN---------------GVKGKP--------------------PYSVETPYGFQIDLDFLK---
Amazon molly Kank4b  ----------------------------------MSRGLIARHKM--------AENGVKGKP-----------------PYSVETPYGFQLDLDFLK---
Platyfish Kank4b     ----------------------------------MSRGLIVRHKM--------AENGVKGKP-----------------PYSVETPYGFQIDLDFLK---
Stickleback Kank4b   ----------------------------------MAAV--------------------------------------------------------------
Fugu Kank4b          ----------------------------------MMEGL--------------KGSTLPLKTKDNGVKGKP--------PYSVETPYGFHLDLDFLK---
Tetraon Kank4b       ----------------------------------MAAA--------------------------------------------------------------
Cave fish Kank4b     ----------------------------------MDAQ---------------KVNGVAPKENGHGSRGRPP-------CYSVETPYGFHLDLDFLK---
Medaka Kank4a        ----------------------------------MEKY-SANGIQ--------TSAKEGGVQRKQL-------------PYSVETPYGFHLDLDFLK---
Fugu Kank4a          ----------------------------------MEKK-NANGVQ--------TKASEGGA---PKKQL----------PYSVETPYGFYLDLDFLK---
Tetradon Kank4a      ----------------------------------MEKK-NANGVQ--------TKAGEGGAPKKQL-------------PYSVETPYGFYLDLDFLK---
Stickleback Kank4a   ----------------------------------MC---PSIDAK--------TKASEGSI---QKKQL----------PYSVETPYGFHLDLDFLK---
Tilapia Kank4a       ----------------------------------MDKK-SANGFQ--------SKAGEGGVQRKQL-------------PYSVETPYGFHLDLDFLK---
Platyfish Kank4a     ----------------------------------MMDKKSANGFQ--------TKVNEGGVQRKQL-------------PYSVETPYGFHLDLDFLK---
Amazon Molly Kank4a  ----------------------------------MMDKKSANGFQ--------TKVNEGGVQRKQL-------------PYSVETPYGFHLDLDFLK---
Herring Kank4        ----------------------------------MMDKK--------------SANGYPSKAPEGGSQRKQI-------PYSVETPYGFHLDLDFLK---
Zebradish Kank4      ----------------------------------MDKK---------------SANGFATKASESGVQRKQL-------PYSVETPYGFHLDLDFLK---
Cave fish Kank4a     ----------------------------------MDK----------------KSANGFSKASETGGQRKQL-------PYSVETPYGFTLDLDFLK---
Mouse KANK4          ----------------------------------MEKI---------------DGKDQSSQGDEEKEPPKSY-------PYSVETPYGFHLDLDFLK---
Rat KANK4            ----------------------------------MEKM---------------DAKDQSPQGDEEKEPPKSY-------PYSVETPYGFHLDLDFLK---
Dog KANK4            ----------------------------------MEKT---------------DAKDQPSQGDEEKGPPKSH-------PYSVETPYGFHLDLDFLK---
Human KANK4          ----------------------------------MEKT---------------DAKDQSSQGDEEKDPPKSH-------PYSVETPYGFHLDLDFLK---
Eagle KANK4          ----------------------------------MEKT---------------DADGQPPKPDGERE---QPRSL----PYSVETPYGFRLDLDFLK---
Chicken KANK4        ----------------------------------MEKT---------------DAAGQPPLPNGDGEQPRSL-------PYSLETPYGFHLDLDFLK---
Goose KANK4          ----------------------------------MEKT---------------DATGQPPQPNGDGQQPRSL-------PYSVETPYGFHLDLDFLK---
Anole lizard KANK4   ----------------------------------ME-----------------TTDGDDWTSKAEDKDPPPS-------PYSVETPYGFHLDLDFLK---
Turtle KANK4         ----------------------------------MEKA---------------DAGNQPSKPAEDKDQPRSL-------PYSVETPYGFHLDLDFLK---
Aligator KANK4       ----------------------------------MEET---------------NAGDQPSKPDGDTDQPRSL-------PYSVETPYGFHLDLDFLK---
Frog Kank2           ---------------------------------MMAQVLHMEAHF--------PGKPGPPSLSSMKDPEA---------AYSVETPYGYRLDLDFLK---
Mouse KANK2          ----------------------------------MAQVLHVPAPF--------PGTPGQASPAAFPSKEPDP-------PYSVETPYGYRLDLDFLK---
Rat KANK2            ----------------------------------MAQVLHVPAPF--------PGTPGQASSAAFPNKEPDP-------PYSVETPYGYRLDLDFLK---
Dog KANK2            ----------------------------------MAQVLHVPAPF--------PGAPGPASPPAFPAREPDP-------PYSVETPYGYRLDLDFLK---
Human KANK2          ----------------------------------MAQVLHVPAPF--------PGTPGPASPPAFPAKDPDP-------PYSVETPYGYRLDLDFLK---
Aligator KANK2       ----------------------------------MAQVLHMDAGF--------PGKPAPPSPPSRGGPAPAPSP-----PYSVETPYGYRLDLDFLK---
Spotted gar Kank2    ----------------------------------MAVSGAI------------SAIPNRVSPGSTEER--------------------------------
Cave fish Kank2      ----------------------------------MAQVLHMETSF--------PGKINSPAPASLHAKDPEA-------PYSVETPYGYRLDLDFLK---
Herring Kank2        ----------------------------------MAQVLHMDPSF--------PGKLNPPAPPSLHGKDAEA-------PYSVETPYGYRLDLDFLK---
Zebrafish Kank2      -------------------------------MTIMAQVLHMDSSF--------PGKINPPVPPSLHAKDQEA-------PYSVETPYGYRLDLDFLK---
Tetradon Kank2       ----------------------------------MAQVLHMDPGF--------PGKLNPPAPPSLHGKEQEA-------PYSVETPYGYRLDLDFLK---
```

```
Fugu Kank2             ----------------------------------MAQVLHMDPGF--------PGKLNPSAPPSLHGKEQEA-------PYSVETPYGYRLDLDFLK---
Medaka Kank2           ----------------------------------MAQVLHMDPTF--------PGKLNSPAPPSLHAKEQEA-------PYSVETPYGYRLDLDFLK---
Sticklebakc Kank2      ----------------------------------MAQVLHMDPGF--------PGKLNPPAPPSLHGKEQEA-------PYSVETPYGYRLDLDFLK---
Tilapia Kank2          ----------------------------------MAQVLHMDPGF--------PGKLNPPAPPSLHAKEQEA-------PYSVETPYGYRLDLDFLK---
Amazon molly Kank2     ----------------------------------MAQVLHMDPGF--------PGKLNSPAPPTLHTKEQEA-------PYSVETPYGYRLDLDFLK---
Platyfish Kank2        ----------------------------------MAQVLHMDPGF--------PGKLNSPAPPTLHTKEQEA-------PYSVETPYGYRLDLDFLK---
Coelacanth KANK2       ----------------------------------MAQVLHMETNF--------PGKSNPTSPTAFRGKDQDL-------PYSVETPYGYRLDLDFLK---
Anole lizard KANK2     ----------------------------------MAQVLHMETAF--------PGKPNPTSATSFHAKEQDM-------PYSVETPYGYRLDLDFLK---
Turtle KANK2           ----------------------------------MAQVLHMESSF--------PGKPTPTSPATFGAQEPPL-------PYSVETPYGYRLDLDFLK---
Tetradon Kank1b        ----------------------------------KTIP---------------------------------------------ETPKQTDLE--------
Medaka Kank1b          ----------------------------------MAQGSYTLRLV--------SGPNAEEAI-----------------PTHLGVDYNYQLD--------
Amazon molly Kank1b    ----------------------------------------------------------------------------------------------------
Platyfish Kank1b       ----------------------------------MAQNNYILRNV--------PGQKSKDCI-----------------PEYQGTESHYPLDLGGIQ---
Frog Kank4             ----------------------------------MERT---------------NGEDILSKTEKKEKRM----------SYSVETPYGFHLDLDFLK---
Zebrafish Kank1b       ----------------------------------MTQETHFHPNQ--------QDWNEITSDAFQKS------------PFSVNTPYGYQLDLDFVK---
Tilapia Kank1b         ----------------------------------MTQETHFHPNQ--------QDWNEITSDAFQKS------------PFSVNTPYGYQLDLDFVK---
Fugu Kank1b            ----------------------------------MTQETHFHPNQ--------QDWNEITSDAFQKS------------PFSVNTPYGYQLDLDFVK---
Cavefish Kank1b        ----------------------------------MAQETYIYMNV--------ADTLESSANTYHSS------------SFCLSTPYGYQLDLDFVK---
Spotted gar Kank4      ----------------------------------MNKS---------------NANGLSSKPCESGGQRKQL-------PYSVETPYGFHLDLDFLK---
Coelacanth KANK4      -------------------------------MAVPERQPAQAEKHDREHVIFVPGNGDSSKTSENGNQQKQL-------PYSLETPYGFHLDLDFLK---
Frog Kank1             ----------------------------------MEQAENLNGNIYKGYCGDNTGDEENIQIGENDKGGNA--------AYFVETPYGYQLDLDFLK---
Shark Kank1            ----------------------------------MAHTVINESV---------PEKGEASLNGEDEKDPQS--------PYSVETPYGYQIDLDFLK---
Coelacanth KANK1       ----------------------------------MAHTTQTNGSI--------SEKGNEVVNGEDDKDTQA--------PYFVETPYGYQLDLDFLK---
Mouse KANK1            ----------------------------------MAYTTKVNGCA--------AEKADGVLNGDHNKEKKD--------PYFVETPYGFQLDLDFVK---
Rat KANK1              ----------------------------------MAYTTKVNG----------CENADGVLNGDRNKEKKD--------PYFVETPYGFQLDLDFVK---
Dog KANK1              ----------------------------------MAHITKVNGCA--------SGKAEDVLNGDHDKEQKD--------PYFVETPYGYQLDLDFLK---
Human KANK1            ----------------------------------MAHTTKVNGSA--------SGKAGDILSGDQDKEQKD--------PYFVETPYGYQLDLDFLK---
Anole lizard KANK1     ----------------------------------MAHPVNINGGS--------TEKDENALNGEDERDQKD--------PYFVETPYGYQLDLDFLK---
Eagle KANK1            ---------------------------------MKLRQPYVAGKFAVWGQRHVANSEKEEVTINGDDEKERKD------PYFVETPYGYQLDLDFLK---
Chicken KANK1          ----------------------------------MAYPANTNGSA--------AEKEEVIINGKDENERKY--------PYFVETPYGYQLDLDFLK---
Goose KANK1            ----------------------------------MAYPANTNGSA--------AEKEEVAVNGEDEKERKD--------PYFVETPYGYQLDLDFLK---
Turtle KANK1           ----------------------------------MAYPATISGST--------SEKEEGVLSGEDDKEQKD--------PYFVETPYGYQLDLDFLK---
Aligator KANK1         ----------------------------------MAYTANINGNN--------SEKEESHLNGKDDKEQKD--------LYFVETPYGYQLDLDFLK---
Spotted gar Kank1      ----------------------------------MAQTAQINGSI--------AEKGEGGLNADDDKDSLA--------PYYLETPYGYQLDLDFLK---
Herring Kank1          ----------------------------------MAQTLHINGNV--------SEKDEGEKDPVA--------------PYYLETPYGFQLDLDFLK---
Zebrafish Kank1a       ----------------------------------MAQTMHINGNA--------SEKDEDEKD-----------------PYCLETPYGYQLDLDFLK---
Cave fish Kank1a       ----------------------------------MAQTMQLNGNT--------TEKDEEDKESVA--------------PYYLETPYGYQLDLDFLK---
Stickleback Kank1a     ----------------------------------MAQTMHVNGNG--------SERGQRCPSADGEKDAAA--------PYYVETPYGYQLDLDFLK---
Medaka Kank1a          ----------------------------------MAQAAQVKGSS--------PGTVGGGEEKD---------------SYYVETPYGYQLDLDFLK---
Fugu Kank1a            ----------------------------------------------------------------------------------------------------
Tetradon Kank1a        ----------------------------------------------------------------------------------------------------
Tilapia Kank1a         ----------------------------------------------------------------------------------------------------
Amazon molly Kank1a    ----------------------------------MAQTVNVNGNG--------PERAQTCPGAGDEKD-----------SYYVETPYGYQLDLDFLK---
Mouse KANK3            ----------------------------------MAKFV-LNQNL--------PDLGGPPLYPGPTGSARSPSS-----PYSVETPYGFHLDLDFLK---
Rat KANK3              ----------------------------------MAKFV-LNQNL--------PDLGGPSLYPGPTGSARSPSS-----PYSVETPYGFHLDLDFLK---
Dog KANK3              ----------------------------------MAKFA-LNQNL--------PDLGGPRLCPGPTAGARSPSS-----PYSVETPYGFHLDLDFLK---
Human KANK3            ----------------------------------MAKF-ALNQNL--------PDLGGPRLCPVPAAGGARSPSS----PYSVETPYGFHLDLDFLK---
Lamprey                ----------------------------------MTDAVGK------------ADAVTMASPSTESRGAGNEHPERSDRCVGTTAPCLAEASTSTAV---
Coelacanth KANK3       ----------------------------------MAQFVQLNQKL--------PDLGGPFQYREEEENDKS--------LYSVETPYGFQLDLDFLK---
Frog Kank3             ----------------------------------MTQSIHLNQNL--------PDLGGPFLYRDQDENEKT--------AYSVETPYGFQLDLDFLK---
Turtle KANK3           ----------------------------------MAQPIHLNQNL--------PDLGGPFLYRDQDDGEKS--------SYSVETPYGFLLDLDFLK---
Aligator KANK3         ----------------------------------MAQPAHLNQNL--------PDLGGPFLYRDQDDGEKS--------SYSVETAYGFTLDLDFLK---
Eagle KANK3            ----------------------------------MAQPAPLNQNL--------PDLGGPFLYRDQDDGEKS--------SYSVETPYGFLLDLDFLK---
Chicken KANK3          ----------------------------------MAQPAHLNQNL--------PDLGGPFLYRDQDDSEKS--------SYSVETPYGFLLDLDFLK---
Goose KANK3            ----------------------------------MAQPAHLNQNL--------PDLGGPFLYRDQDDGEKS--------SYSVETPYGFLLDLDFLK---
Spotted gar Kank3      ----------------------------------MTQSVQLNQKL--------PDVGTPFLYSTQEEGDQGG-------SYSVQTPYGFQLDLDFLK---
Zebrafish Kank3        ----------------------------------MTQSVHITSKV--------PNLSAPFHCSAEEESDQSG-------AYSVQTPYGFELDLDFLK---
```

```
Cavefish Kank3        ----------------------------------MTQSVQINPKL--------PNLGAPFQCATQEEMDQSA-------SYSVQTPYGFQLDLDFLK---
Herring Kank3         ----------------------------------MTQSVQVNSKL--------PDLGAPFLYGSQEETDQAG-------SYSVQTPYGFQLDLDFLK---
Stickleback Kank3     ----------------------------------MTQSVQVNPKL--------PDLGSPFIYSAQEDADQSG-------SYSVQTPYGFQLDLDFLK---
Fugu Kank3            ----------------------------------MTQSVQMNPKL--------PDLGSPFMYPSQGETDQPA-------SYSVQTPYGFQLDLDFLK---
Tetradon Kank3        ----------------------------------MAQSVQMNPNL--------PDLASPFICSGRGEGDQPG-------SYSVQTPYGFQLDLDFLK---
Medaka Kank3          ----------------------------------MSQSVQVNPKM--------PDLGSKFLYSSQEEADQSS-------SYSVQTPYGFQLDLDFLK---
Tilapia Kank3         ----------------------------------MTQSVQVNPKL--------PDLGSPFHYPSQEEADQQA-------SYSVQTPYGFQLDLDFLK---
Amazon molly Kank3    ----------------------------------MTQSVQVNPKV--------PDIGSAFIYSSQEEADQSG-------SYSVQTPYGFQLDLDFLK---
Platyfish Kank3       ----------------------------------MTQSVQVNPKV--------PDLGSAFIYSGQEEADQSG-------SYSVQTPYGFQLDLDFLK---

                               110       120       130       140       150       160       170       180       190       200
                      ....|....|....|....|....|....|....|....|....|....|....|....|....|....|....|....|....|....|....|....|
Hydra                 -FTDTSCDSAK---VFKDVGVQ---KSLNYPV-----------------MKDIGINV-------------------------------------------
Trichoplax            AIID----------LMNQLTVN------------------------------------------------------------------------------
Aplasia               -FCR-DVEGGSTLRNLKRIQRA---KRKLRKS---MEIMLDEQEGIAADPNSAPPDV-------------------------------------------
Snail                 -FCR-DLESGS---TLRNLKRI---RRNKKKLRKSMELMLDEQEGRTFDPSSAPPDI-------------------------------------------
Fruitfly              -YCE-AL-------ALEKPSEEEQRRRDRRRS------RK---------SMEFMLGF-------------------------------------------
Hookworm              EYAVSDTENAH---RVRYYSAS-----PKMPR------K----------LQAVPPLP-------------------------------------------
C.elegance            -YAQ-NVSNG----FVSRQGTP---------------------------TNMTLRGN-------------------------------------------
Tunicate              -YCE-ELMSGQ---SFKRTSSF---RRPR--------------------SRTASQAS-------------------------------------------
Spider                -YCD-SLYNTA---QLDKLRQH---KRERRKE-----------------KKTVESYL-------------------------------------------
Lingula               -YCE-DSQSGTMLKQLKKIKRD---KKRLRKS-----------------MEVQLQGQ-------------------------------------------
Lancelet              -YCD-DISKGS---NIPKIVRP---RKPKSPG------GPAPMDFR---ANTLPRGFYLNSQQSNLPKHASVVPSSPISPLSVNTSLSSANNSIPETTTT
Medaka Kank4b         -YVE-DIEKGN---TIKRVPIQ---RRSKGPR-----------------ASTLPRNL-------------------------------------------
Amazon molly Kank4b   -YVD-DIEKGN---TIKRVPIQ---RRSKGSR-----------------ASTLPRHL-------------------------------------------
Platyfish Kank4b      -YVD-DIEKGN---TIKRVPIQ---RRSKGPR-----------------ASTLPRHL-------------------------------------------
Stickleback Kank4b    --------------QVDGVPGE---RRM------------------------------------------------------------------------
Fugu Kank4b           -YVD-DIEKGN---TIRRIPIH---RRSKGPR-----------------ASTLPRHF-------------------------------------------
Tetraon Kank4b        -QVD------------------------------------------------------------------------------------------------
Cave fish Kank4b      -YVD-DIEKGN---TIRRVPIQ---RRQRGRT-----------------TGSLSRNL-------------------------------------------
Medaka Kank4a         -YVD-DIEKGN---TIKRVHIH---RRIKGPAK----------------FSTLPRNF-------------------------------------------
Fugu Kank4a           -YVD-DIEKGN---TIKRVHIQ---RRIKGPS-K---------------FSTLPRNF-------------------------------------------
Tetradon Kank4a       -YVD-DIEKGN---TIKRVHIQ---RRIKGPP-K---------------FSTLPRNF-------------------------------------------
Stickleback Kank4a    -YVD-DIEKGN---TIKRVHIQ---RRVKSPA-K---------------FSTLPRNF-------------------------------------------
Tilapia Kank4a        -YVD-DIEKGN---TIKRVHIQ---RRVKGPPK----------------FSTLPRNF-------------------------------------------
Platyfish Kank4a      -YVD-DIEKGN---TIKRVHIQ---RRVKGPA-K---------------FSTLPRNF-------------------------------------------
Amazon Molly Kank4a   -YVD-DIEKGN---TIKRVHIQ---RRVKGPA-K---------------FSTLPRNF-------------------------------------------
Herring Kank4         -YVD-DIEKGN---TIKRVHIQ---RKNRGPK-----------------YSTLPRNF-------------------------------------------
Zebradish Kank4       -YVD-DIEKGN---TIKRVHIQ---RKNRGPK-----------------YSTLPRNF-------------------------------------------
Cave fish Kank4a      -YVD-DIEKGN---TIKRVHIQ---RKNRGPK-----------------YSTLPRNF-------------------------------------------
Mouse KANK4           -YVD-DIEKGH---TIKRIPIH---RRAKQAK-----------------FSTLPRNF-------------------------------------------
Rat KANK4             -YVD-DIEKGH---TIKRIPIH---RRAKQAK-----------------FSTLPRNF-------------------------------------------
Dog KANK4             -YVD-DIEKGN---TIRRIPIH---RRAKQAK-----------------FSTLPRNF-------------------------------------------
Human KANK4           -YVD-DIEKGN---TIKRIPIH---RRAKQAK-----------------FSTLPRNF-------------------------------------------
Eagle KANK4           -YVD-DIEKGN---TIRRVHIH---RKAKQPK-----------------FSTLPRNF-------------------------------------------
Chicken KANK4         -YVD-DIEKGN---TIRRVHIH---RKAKQPK-----------------FSTLPRNF-------------------------------------------
Goose KANK4           -YVD-DIEKGN---TIRRVHIH---RKAKQPK-----------------FSTLPRNF-------------------------------------------
Anole lizard KANK4    -YVD-DIEKGN---TIKRIQIH---RKTKYPK-----------------FSTLPRNF-------------------------------------------
Turtle KANK4          -YVD-DIEKGN---TIKRVHIH---RKAKQPK-----------------FSTLPRNF-------------------------------------------
Aligator KANK4        -YVD-DIEKGN---TIRRVHVH---RKAKQPK-----------------FSTLPRNF-------------------------------------------
Frog Kank2            -YVD-DIEKGH---TIKRVPVH---RRPR--------------------YGSLPRGP-------------------------------------------
Mouse KANK2           -YVD-DIEKGH---TLRRVAVQ---RRPR--------------------LGSLPRGP-------------------------------------------
Rat KANK2             -YVD-DIEKGH---TLRRVPVQ---RRPR--------------------LGSLPRGP-------------------------------------------
Dog KANK2             -YVD-DIEKGH---TLRRVAVQ---RRPR--------------------LGSLPRGP-------------------------------------------
Human KANK2           -YVD-DIEKGH---TLRRVAVQ---RRPR--------------------LSSLPRGP-------------------------------------------
Aligator KANK2        -YVD-DIEKGH---TLRRLPVR---RRPR-----------------------------------------------------------------------
Spotted gar Kank2     --------------RVCQLQMR---------------------------SLSEPGRS-------------------------------------------
```

```
Cave fish Kank2       -YVN-DIEKGN---TLKKVPVQ---RRPR--------------------YGSLPRGY-------------------------------------------
Herring Kank2         -YVN-DIEKGN---TIKRVPVQ---RRPR--------------------YGSLPRGY-------------------------------------------
Zebrafish Kank2       -YVN-DIEKGN---TIKKVPVQ---RRPR--------------------YGSLPRGY-------------------------------------------
Tetradon Kank2        -YVN-DIEKGN---TIKKVAVQ---RRPR--------------------YGSLPRGY-------------------------------------------
Fugu Kank2            -YVN-DIEKGN---TIKKVAVQ---RRPR--------------------YGSLPRGY-------------------------------------------
Medaka Kank2          -YVN-DIEKGN---TIKKVSIQ---RRPR--------------------YGSLPRGY-------------------------------------------
Sticklebakc Kank2     -YVN-DIEKGN---TIKKVPIQ---RRPR--------------------YGSLPRGY-------------------------------------------
Tilapia Kank2         -YVN-DIEKGN---TIKKVPVQ---RRPR--------------------YGSLPRGY-------------------------------------------
Amazon molly Kank2    -YVN-DIEKGN---TIKKVPIQ---RRPR--------------------YGSLPRGY-------------------------------------------
Platyfish Kank2       -YVN-DIEKGN---TIKKVPIQ---RRPR--------------------YGSLPRGY-------------------------------------------
Coelacanth KANK2      -YVD-DIEKGH---TIKKVPIH---RRPR--------------------YSSLPRGY-------------------------------------------
Anole lizard KANK2    -YVD-DIEKGH---TIKRIPVH---RRPR--------------------YSSLPRGY-------------------------------------------
Turtle KANK2          -YVD-DIEKGN---TIRRVPVH---RRPR--------------------YGSLPRGA-------------------------------------------
Tetradon Kank1b       --------------VPNQAAPQ---PRPRQRL--------------------------------------------------------------------
Medaka Kank1b         -----RFQKGA---TIKRLSLK---RRPRVV------------------LSDAEKTV-------------------------------------------
Amazon molly Kank1b   ---------------MKRLSLK---RRPRGV------------------LNDVEKSQ-------------------------------------------
Platyfish Kank1b      -NII----------TMKRLSLK---RRPRGV------------------LNDVEKTQ-------------------------------------------
Frog Kank4            -YVH-DIEKGN---TIKRIHIH---RRAKQSK-----------------FSTLPRNF-------------------------------------------
Zebrafish Kank1b      -YVD-DIERSD---TIRRLRFN---KRPGVAN-----------------AASEPQHP-------------------------------------------
Tilapia Kank1b        -YVD-DIERSD---TIRRLRFN---KRPGVAN-----------------AASEPQHP-------------------------------------------
Fugu Kank1b           -YVD-DIERSD---TIRRLRFN---KRPGVAN-----------------AASEPQHP-------------------------------------------
Cavefish Kank1b       -YVD-DIEQSD---TIRRLGFN---RRPR--------------------ADPAPEAQ-------------------------------------------
Spotted gar Kank4     -YVD-DIEKGN---TIKRVHIH---RRAKDPK-----------------FSTLPRNF-------------------------------------------
Coelacanth KANK4      -YVD-DIEKGN---TIKRVHIH---RKAKQPK-----------------YSTLPRNF-------------------------------------------
Frog Kank1            -YVE-DIQKGN---TIKKVSLL---KKRKLVP-----------------NLAEGRNP-------------------------------------------
Shark Kank1           -YVD-DIQRGN---TIKKVNIQ---RKPKVPK-----------------TLATTRNA-------------------------------------------
Coelacanth KANK1      -YVD-DIQRGN---TIKKLNIQ---RKSKVAK-----------------PIVTPKSA-------------------------------------------
Mouse KANK1           -YVD-DIQKGN---TIKKLNIQ---KRRKPSV-----------------PCPEVRAI-------------------------------------------
Rat KANK1             -YVD-DIQKGN---TIKKLNIQ---KRRKPSG-----------------PCAEVRAI-------------------------------------------
Dog KANK1             -YVD-DIQKGN---TIKKLNIP---KRRKPSV-----------------PCPESRAT-------------------------------------------
Human KANK1           -YVD-DIQKGN---TIKRLNIQ---KRRKPSV-----------------PCPEPRTT-------------------------------------------
Anole lizard KANK1    -YVE-DIQKGN---TIKKLNIQK--KRKVIPP-----------------FATVKNTT-------------------------------------------
Eagle KANK1           -YVD-DIQKGN---TIKKLNIR---KKRKAVP-----------------ASAGTKNS-------------------------------------------
Chicken KANK1         -YVD-DIQKGN---TIKKLNIQ---KKRKAVP-----------------ASTSTKNA-------------------------------------------
Goose KANK1           -YVD-DIQKGN---TIKKLNIQ---KKRKAIP-----------------ASTGTKNS-------------------------------------------
Turtle KANK1          -YVD-DIQKGN---TIKKLNIQ---KKRKLVP-----------------TFTGTKNS-------------------------------------------
Aligator KANK1        -YVD-DIQKGN---TIKKLNIQ---KKRKLVP-----------------TFPGTKNS-------------------------------------------
Spotted gar Kank1     -YVD-DIERGN---TLKKLNIQ---RKPKVAK-----------------PVATPRSG-------------------------------------------
Herring Kank1         -YVD-DIERGN---TIKKLNFQ---RKPKSAR-----------------PSTSSQHS-------------------------------------------
Zebrafish Kank1a      -YVD-DIERGN---TIKKLNIQ---RKPKVVR-----------------TVPPPRSS-------------------------------------------
Cave fish Kank1a      -YVD-DIERGN---TIKKLNIQ---RKPKVPR-------------------CVPSSQ-------------------------------------------
Stickleback Kank1a    -YVD-DIERGN---TIKKLSVH---RKPKGAT-----------------PSTSG----------------------------------------------
Medaka Kank1a         -YVE-DIERGN---TIKKLSIQ---RKPKVAR-----------------TPPAPRAS-------------------------------------------
Fugu Kank1a           ----------------------------------------------------------------------------------------------------
Tetradon Kank1a       ----------------------------------------------------------------------------------------------------
Tilapia Kank1a        ----------------------------------------------------------------------------------------------------
Amazon molly Kank1a   -YVD-DIERGN---TIKKLSIQ---RKPKAAR-----------------PLATPRGS-------------------------------------------
Mouse KANK3           -YVE-EIERGP---ASRRTPGPPHARRPRASRTGL--------------AGARSPGA-------------------------------------------
Rat KANK3             -YVE-EIERGP---ASRRAPGPPHARRPRASG------KGL--------AGARSPGA-------------------------------------------
Dog KANK3             -YVE-ELERGP---APRRAPGPPPARRPRAPR-----------------AGLAGARS-------------------------------------------
Human KANK3           -YIE-ELERGP---AARRAPGPPTSRRPRAPR-----------------PGLAGARS-------------------------------------------
Lamprey               -SCR-DAQSGTVAVEVRTVSVG---NCER--------------------YEDTRRSV-------------------------------------------
Coelacanth KANK3      -YVD-DIQSGQ---TLRKVNVQ---RKSKPAK-----------------PTTSLRSP-------------------------------------------
Frog Kank3            -YVD-DIQSGQ---TLKKVPVY---RKPRVPRQ----------------STSSLRSP-------------------------------------------
Turtle KANK3          -YVD-DIESGQ---TLKKVPKH---RKAKGPK------Q----------QPSSLRSP-------------------------------------------
Aligator KANK3        -YVD-DIQSGQ---TLKKLH-----RKAKGPKQ----------------QLSCLRSP-------------------------------------------
Eagle KANK3           -YVN-DIESGQ---TLKKVPLP---RRAKGARQ----------------PPSALRSP-------------------------------------------
```

```
Chicken KANK3        -YVD-DIESGQ---TLKKVPLP---RRAKGTRL----------------QPGTLRSP-------------------------------------------
Goose KANK3          -YVD-DIESGQ---TLKKVPLP---RRAKGAR--------------------------------------------------------------------
Spotted gar Kank3    -YVE-EIESGH---NFRRAPVNPR-RGPRSSK-----------------SGSLSRRS-------------------------------------------
Zebrafish Kank3      -YVE-EIESGH---SIRRANVH---PRRG--------------------SRSDRRNL-------------------------------------------
Cavefish Kank3       -YVE-EIESGQ---SIRRAHMSP--RRPSRS------------------DRGSQRSA-------------------------------------------
Herring Kank3        -YVE-EIESGH---TFRRTPVSSR-RGVRAGK-----------------LSQRSPGV-------------------------------------------
Stickleback Kank3    -YVE-EIESGH---NLRRAPLG---SRRSGRG-----------------ARLSQRGA-------------------------------------------
Fugu Kank3           -YVE-EIESGH---HLRRAPVSSR-RSSRGTK-----------------LSLRSPSV-------------------------------------------
Tetradon Kank3       -YVE-EIESGH---NLRRAPVGSR-RSSRGPR-----------------LSQRSPSV-------------------------------------------
Medaka Kank3         -YVE-EIESGH---NLRRAPVSS--RRPGRGLK----------------LSQRSASV-------------------------------------------
Tilapia Kank3        -YVE-EIESGH---NLRRAPISSR-RSGRGLK-----------------LSQRNPGV-------------------------------------------
Amazon molly Kank3   -YVE-EIESGH---NLRRAPLSS--RRPGRGLK----------------LSQRSPSV-------------------------------------------
Platyfish Kank3      -YVE-EIESGH---NLRRAPLSS--RRPGRGL------K----------LSQRSPSV-------------------------------------------

                             210       220       230       240       250       260       270       280       290       300
                     ....|....|....|....|....|....|....|....|....|....|....|....|....|....|....|....|....|....|....|....|
Hydra                -----------------------------------------------FLT---FL-DRACQTKTETND--------------------------------
Trichoplax           ------------------------------------------------------S-ETSCPNNYESND--------------------------------
Aplasia              ------------------------VHSTEAGRLMHMINYERSATHRILNQ---ID-TTVNTTIAGGGR-------------------------HMSSDSD
Snail                ------------------------VHSTEAGRLMHMINYERSATHHILSQ---ID-SSVNTTIANIDG-----------------------VRGRRYMSS
Fruitfly             ----------------------------------------ESLFGGDWQA----E-SRVQGKLSETSH--------ESEPDSPHPIGGGGSLHMSQSYTT
Hookworm             -----------------------------------------SASQFTYTH---NN-GNTMLAKTQGTS--------------------------------
C.elegance           -----------------------------------------GADRHLMSP----LLDNSIFSDSLDNL--------------------------------
Tunicate             -----------------------------------------GQQQVHYNT---RL-AGGPSNESLDSL--------------------------------
Spider               -----------------------------------------GLGNICSSQ---YS-EPNLTTVRQHNT-------------------------LPNDFYL
Lingula              ------------------------------------------QIETDYEP----R-QEMVGYDSGTNR-------------------------YLNDIDS
Lancelet             TATTTKQTVTDPRENASSVYGYNSSTRRPSLPGDLPIPFTSPNSNSVFTM----P-AALATQSNSGSSSSLSSQLSTQSSSGQFSPHSEMSGDTKHS---
Medaka Kank4b        -----------------------------------------NPSGCGYRP------SPWGSTGALGPR--------------------------------
Amazon molly Kank4b  -----------------------------------------NSSGSGYKP------SPWRSTGALDPR--------------------------------
Platyfish Kank4b     -----------------------------------------NSSGSGYKQ------NPWRSTGALDPR--------------------------------
Stickleback Kank4b   --------------------------------------------------------EGMTRTNPGGQM--------------------------------
Fugu Kank4b          -------------------------------------------TASGWGS----T-GALGSRQLEASC--------------------------------
Tetraon Kank4b       ---------------------------------------------------------------ANGSR--------------------------------
Cave fish Kank4b     -----------------------------------------SLPGYGCSR----P-SQWNSTGILFPR--------------------------------
Medaka Kank4a        -----------------------------------------SLPGHGVRPTLKDKDSAWPGTSTLGPK--------------------------------
Fugu Kank4a          -----------------------------------------SLPGHGVRPVPKEKDSTWSGTSTLGPK--------------------------------
Tetradon Kank4a      -----------------------------------------SLPGHGVRPVPKEKDSTWSGTSTLGPK--------------------------------
Stickleback Kank4a   -----------------------------------------SLPGHGARPPPKDKDSTWSGTSTLGPK--------------------------------
Tilapia Kank4a       -----------------------------------------SLPGHGVRPPPKEKDSTWSGTSTLGPK--------------------------------
Platyfish Kank4a     -----------------------------------------SLPGHGVRP--PPKEGAWSGTSTLGPK--------------------------------
Amazon Molly Kank4a  -----------------------------------------SLPGHGVRP--PSKEGAWSGTSTLGPK--------------------------------
Herring Kank4        -----------------------------------------SLPGHGARP---PPKDVWASGTSTLGP--------------------------------
Zebradish Kank4      -----------------------------------------SLPGHGARP---LAKDTWANTSTLGSK--------------------------------
Cave fish Kank4a     -----------------------------------------SLPGHGARP---AAKDTWANTSTLGSK--------------------------------
Mouse KANK4          -----------------------------------------SLPNSGDRT----Y-AVPPQQNWSPVV--------------------------------
Rat KANK4            -----------------------------------------SLPNSGDRS----H-AAPPQQNWSPVV--------------------------------
Dog KANK4            -----------------------------------------SLPDSRARP----H-AALSHQNWSPVV--------------------------------
Human KANK4          -----------------------------------------SLPDSGARP----P-AAPPLQNWSPVV--------------------------------
Eagle KANK4          -----------------------------------------SLPENGSRG----C-ASTPGKGWAATR--------------------------------
Chicken KANK4        -----------------------------------------SLPENGSHG----Y-AASTGPGWPAAT--------------------------------
Goose KANK4          -----------------------------------------SLPENGSRG---YA-AALLLASSMPAA--------------------------------
Anole lizard KANK4   -----------------------------------------SLPENGSRA---RNKNFIAQRKASLGA--------------------------------
Turtle KANK4         -----------------------------------------SLPENRSRA----Y-ASSPNKSWTATC--------------------------------
Aligator KANK4       -----------------------------------------SLPENGSRA----Y-AASSNKSWAAFA--------------------------------
Frog Kank2           -----------------------------------------GYTGSWWTS----T-ESLCSNASNDSR--------------------------------
Mouse KANK2          --------------------------------------------GSWWTS----T-ESLCSDASGDSR--------------------------------
Rat KANK2            --------------------------------------------GSWWTS----T-ESLCSDASGDSR--------------------------------
```

```
Dog KANK2              --------------------------------------------GSWWTS----T-ESLCSNASGDSR--------------------------------
Human KANK2            --------------------------------------------GSWWTS----T-ESLCSNASGDSR--------------------------------
Aligator KANK2         -----------------------------------------CASASWWPS----T-ESLCSTDSLASA--------------------------------
Spotted gar Kank2      -----------------------------------------SLGTGGFNF--------------------------------------------------
Cave fish Kank2        -----------------------------------------GYTGSWWTS----T-ESLCSNASVDSR--------------------------------
Herring Kank2          -----------------------------------------GYTGSWWTS----T-ESLCSNTSVDSR--------------------------------
Zebrafish Kank2        -----------------------------------------GYTGSWWTS----T-ESLCSNASMDSR--------------------------------
Tetradon Kank2         -----------------------------------------GYAGSWWTS----T-ESLCSNASMDSR--------------------------------
Fugu Kank2             -----------------------------------------GYTGSWWTS----T-ESLCSNTSIDSR--------------------------------
Medaka Kank2           -----------------------------------------GYTGSWWTS----T-ESLCSNASMDSR--------------------------------
Sticklebakc Kank2      -----------------------------------------GYTGSWWTS----T-ESLCSNTSMDSR--------------------------------
Tilapia Kank2          -----------------------------------------GYTGSWWTS----T-ESLCSNASMDSR--------------------------------
Amazon molly Kank2     -----------------------------------------GYTGSWWTS----T-ESLCSNASVESR--------------------------------
Platyfish Kank2        -----------------------------------------GYTGSWWTS----T-ESLCSNASVESR--------------------------------
Coelacanth KANK2       -----------------------------------------GYTGSWWTS----T-ESLCSNASMDSR--------------------------------
Anole lizard KANK2     -----------------------------------------GYTGSWWTS----T-ESLCSNVSMDSH--------------------------------
Turtle KANK2           --------------------------------------------GSWWTS----T-ESLCSNASLDSR--------------------------------
Tetradon Kank1b        --------------------------------------------------------ASFGGVSSPGSA--------------------------------
Medaka Kank1b          -------------------------------------------SSSQWHS----A-ESLSSSSSDDFR------------------------LLGMSSSA
Amazon molly Kank1b    -----------------------------------------SGASSGPVH---SA-ESLSLPGNDGTR-----------------------LLGMSSSAK
Platyfish Kank1b       -----------------------------------------SGSSCGPVH---SA-ESLSLPGNDGTR-----------------------LLGMSSSAK
Frog Kank4             -----------------------------------------SVPDNGSQS----Y-ISSSSRTWSPTS--------------------------------
Zebrafish Kank1b       -----------------------------------------INPTHRISS------ESLSSVSSDEAK---------------------KTSSSSSAVNS
Tilapia Kank1b         -----------------------------------------INPTHRISS------ESLSSVSSDEAK---------------------KTSSSSSAVNS
Fugu Kank1b            -----------------------------------------INPTHRISS------ESLSSVSSDEAK---------------------KTSSSSSAVNS
Cavefish Kank1b        ----------------------------------------SRVDPSQWAS----S-ESLSSVSSDEAR----------------NPSVTTTSSISSSINR
Spotted gar Kank4      -----------------------------------------SLPDHGSRS------APKDSWNTPGSY--------------------------------
Coelacanth KANK4      -----------------------------------------SIPNNGSRS---YE-SSFSSGNWTSSY--------------------------------
Frog Kank1             -----------------------------------------PSQEAGWPS----T-DSLSSSNSDDNK---------------------------HSPSL
Shark Kank1            -----------------------------------------GGQVSGWTS----T-ESLSSSNSEDNK--------------------------LSPSLF
Coelacanth KANK1       -----------------------------------------AGQFSGWTS----T-ESLSSSNSDENK-----------------------LSPSFLTTK
Mouse KANK1            -----------------------------------------PGHQGVWTS----T-ESLSSSNSDDSK------------------------QCPSFLLA
Rat KANK1              -----------------------------------------PGPQGVWTS----T-ESLSSSNSDDSK------------------------QCPSFLLA
Dog KANK1              -----------------------------------------TGQQGVWTS----T-ESLSSSNSDDNK------------------------QCPHFLLA
Human KANK1            -----------------------------------------SGQQGIWTS----T-ESLSSSNSDDNK------------------------QCPNFLIA
Anole lizard KANK1     -----------------------------------------PSQYSGWTS----N-ESLSSSNSDDNK----------------------HSSSFCAARS
Eagle KANK1            -----------------------------------------GGQCGGWTS----T-ESLSSSNSDENK--------------------------QSCLAA
Chicken KANK1          -----------------------------------------GGHCGDWTS----T-ESLSSSNSDENK--------------------------QSFLAA
Goose KANK1            -----------------------------------------GGHCSGWTS----T-ESLSSSNSDENK--------------------------QSFLAA
Turtle KANK1           -----------------------------------------SGQYSGWTS----T-ESLSSSNSDENK--------------------------QSSFAA
Aligator KANK1         -----------------------------------------SVQYSGWTS----T-ESLSSSNSDENK--------------------------QSFLAA
Spotted gar Kank1      -----------------------------------------GGQTSGWTS----T-ESLSSSNSDECK--------------------------QSPLLP
Herring Kank1          ------------------------------NNNSVSSSSGGGCGLAEWTS----T-DSLSSSNSDENK------------------QSQSPVFLNPGYLP
Zebrafish Kank1a       -----------------------------------------CSGHTEWTS----T-DSLSSSNSDECK--------------------------QSPVYF
Cave fish Kank1a       -----------------------------------------SGGQAEWTS----T-DSLSSSNSDESK--------------------------QSPVYF
Stickleback Kank1a     -----------------------------------------GGAPAEWTS----T-DSLSSSNSDDKQ--------------------------------
Medaka Kank1a          -------------------------------------------AQAEWTS----T-DSLSSSNSDDKQ--------------------------------
Fugu Kank1a            ----------------------------------------------------------------------------------------------------
Tetradon Kank1a        ----------------------------------------------------------------------------------------------------
Tilapia Kank1a         ----------------------------------------------------------------------------------------------------
Amazon molly Kank1a    --------------------------------------TAGGGARPEWTS----T-DSLSSSNSDGKQ-------------------------SPVFFTS
Mouse KANK3            -----------------------------------------------WTS----S-ESLASDDGGASG--------------------------------
Rat KANK3              -----------------------------------------------WTS----S-ESLASDDGGASG--------------------------------
Dog KANK3              -----------------------------------------PGA---WTS----S-ESLASDDGGASG--------------------------------
Human KANK3            -------------------------------------------PGAWTSS------ESLASDDGGAPG--------------------------------
Lamprey                -----------------------------------------GVNTAGVGA----A-IRLSRREAAVGS--------------------------------
Coelacanth KANK3       -----------------------------------------NSHTSGWTS----T-ESLSSTTSEDSK--------------------------NMPIFF
```

```
Frog Kank3            -----------------------------------------SCQTGGWTS----T-ESLTFSDDGRSS--------------------------NVFF
Turtle KANK3          -----------------------------------------SGHASGWTS----T-ESLASTASEDGR--------------------------NVLLLS
Aligator KANK3        -----------------------------------------GSHASGWTS----T-ESLASTASEDGR--------------------------TTLLLS
Eagle KANK3           -----------------------------------------SSHASAWTS----T-ESLASTASEEGR--------------------------------
Chicken KANK3         -----------------------------------------SSHPSGWTS----T-ESLASTLSEDGR-----------------------------SAV
Goose KANK3           --------------------------------------------------------QPLGTLLVDGGR--------------------------------
Spotted gar Kank3     --------------------------------------PGAGGHTSGWTS----T-ESLSSSASEEGR-----------------------------VPP
Zebrafish Kank3       ---------------------------------------STGGRTSGWTS----T-ESLSSTTSENGQ-------------------------------T
Cavefish Kank3        -----------------------------------------GGRASGWTS----T-ESLASTTSEDGR------------------------SAPPPVAR
Herring Kank3         -----------------------------------------GGRASGWTS----T-ESLSSSASDDGS----------------------------RVPP
Stickleback Kank3     -----------------------------------------GGRASGWTS----T-ESLASPASEDGS--------------------------------
Fugu Kank3            -----------------------------------------GGRTSGWTS----T-ESLSSPASEDGR--------------------------------
Tetradon Kank3        -----------------------------------------GGRTSGWTS----T-ESLSSPASEDGR--------------------------------
Medaka Kank3          -----------------------------------------GGRTSGWTS----T-ESLASPASEDGR-----------------------------APP
Tilapia Kank3         -----------------------------------------GGRTSGWTS----T-ESLSSPVSEDGR--------------------------------
Amazon molly Kank3    -----------------------------------------GGRTSGWTS----T-ESLSSPVSEDGR-----------------------------VPP
Platyfish Kank3       -----------------------------------------GGRTSGWTS----T-ESLSSPVSEDGR-----------------------------VPP

                               310       320       330       340       350       360       370       380       390       400
                      ....|....|....|....|....|....|....|....|....|....|....|....|....|....|....|....|....|....|....|....|
Hydra                 ----------------------------------------------------------------------------------------------------
Trichoplax            ----------------------------------------------------------------------------------------------------
Aplasia               EPMTPYSPVHGGRPPVDETPP-FSASGR------------------------------------------------------------------------
Snail                 DSDEPVTPFSSSSYRAAYSNDGAMTPPL------------------------------------------------------------------------
Fruitfly              TPCYRPRSSSVPRFTYGSIPPRSESPFG------------------------------------------------------------------------
Hookworm              ----MSPKPEEEKKMFSKQLTTVGVWTG------------------------------------------------------------------------
C.elegance            --MSDFDDVLAPHKEVEKKKTVQDIRSR------------------------------------------------------------------------
Tunicate              --------------------ASEGTCEG------------------------------------------------------------------------
Spider                NSESLKDAVTDFEDTWQRSLKKSSSSYA------------------------------------------------------------------------
Lingula               SVDTTLHTIDKVIKQTSYRTYSAGDSDF------------------------------------------------------------------------
Lancelet              --MGVQTPASLLSSGSSSRGSGASTPQPPAVSSAMLQGIREQIAASLKRIKVLEEQVKEIPVLQVKISVLKEEKRLLVMQLKSKNGEQNLKPEDTDNKRE
Medaka Kank4b         --SPPSDTHHHGYSLWAHSHRPPLFPTD------------------------------------------------------------------------
Amazon molly Kank4b   --SRPLDNHHHGCSSWTFDHRPPLSPTG------------------------------------------------------------------------
Platyfish Kank4b      --SRPFANHHHGCSSWTFDHRSLLYPTG------------------------------------------------------------------------
Stickleback Kank4b    ----------------------------------------------------------------------------------------------------
Fugu Kank4b           --------SPHGYASWSNGHRSHLSPTG------------------------------------------------------------------------
Tetraon Kank4b        ----------------------------------------------------------------------------------------------------
Cave fish Kank4b      ---PRLADLQQSYGNTAFQSSDGGSTAL------------------------------------------------------------------------
Medaka Kank4a         ----PKSRVTEVQQIFDFRGGEGGTSSC------------------------------------------------------------------------
Fugu Kank4a           ----PKSRVAEVQQIFDFRASE------------------------------------------------------------------------------
Tetradon Kank4a       ----PKSRVAEVQQIFDFRASERTTSTQ------------------------------------------------------------------------
Stickleback Kank4a    ----PKSRVTEVQQIYDFRASEGGTSSH------------------------------------------------------------------------
Tilapia Kank4a        ----PKSRVTEVQQIFDFRANEGVTSSL------------------------------------------------------------------------
Platyfish Kank4a      ----PKSRVTEVQQIFDFRPSEGAISSH------------------------------------------------------------------------
Amazon Molly Kank4a   ----PKSRVTEVQQIFDFRPSEGAISSH------------------------------------------------------------------------
Herring Kank4         ---RPKSRVTEVQQIFDFKPSDGGSPSL------------------------------------------------------------------------
Zebradish Kank4       ----PKSRVTEVQQLFEFRASDATGGSS------------------------------------------------------------------------
Cave fish Kank4a      ----PKSRVTEVQQLFEFRASDGSSSSG------------------------------------------------------------------------
Mouse KANK4           -----PRKISLGTQEPSQPLPLGDLPQA------------------------------------------------------------------------
Rat KANK4             -----PRKISLGTQEQSQSVPLGDLPQA------------------------------------------------------------------------
Dog KANK4             -----PRKVLLGTEERAQSLPPGEYPQA------------------------------------------------------------------------
Human KANK4           -----PREASLGTQEQNQSPPLGNAPQA------------------------------------------------------------------------
Eagle KANK4           SFPQRKASLGAEETPRPLPPDEAPLPVA------------------------------------------------------------------------
Chicken KANK4         --YRAQRKASLGAEEISRAPLPSEEPSY------------------------------------------------------------------------
Goose KANK4           --------------LLPGQPPAGDGCEL------------------------------------------------------------------------
Anole lizard KANK4    ------DESTHLITPTDSTPFSGISNEP------------------------------------------------------------------------
Turtle KANK4          --SFAQRKASLGAEETTCLITSYDSPQS------------------------------------------------------------------------
```

```
Aligator KANK4       HRKTSLGAEETIRLVTTNESPPFVTPTD------------------------------------------------------------------------
Frog Kank2           ------------HSSYSYCGRGFYPAYE------------------------------------------------------------------------
Mouse KANK2          ------------HSAYSYCGRGFYPQYG------------------------------------------------------------------------
Rat KANK2            ------------HSAYSYCGRGFYPQYG------------------------------------------------------------------------
Dog KANK2            ------------HSAYSYCGRGFYPQYG------------------------------------------------------------------------
Human KANK2          ------------HSAYSYCGRGFYPQYG------------------------------------------------------------------------
Aligator KANK2       ---------------LSYGP--------------------------------------------------------------------------------
Spotted gar Kank2    ----------------------------------------------------------------------------------------------------
Cave fish Kank2      ------------HSSYSYCAPGYHTAQR------------------------------------------------------------------------
Herring Kank2        ------------HSSFSYCAPGYHHPHH------------------------------------------------------------------------
Zebrafish Kank2      ------------HSSYSYCAPGFHTSQR------------------------------------------------------------------------
Tetradon Kank2       ------------HSSYSYCAPGYHTAQR------------------------------------------------------------------------
Fugu Kank2           ------------HSSYSYCAPGYHSTQR------------------------------------------------------------------------
Medaka Kank2         ------------HSSYSYCAPGYHTSQR------------------------------------------------------------------------
Sticklebakc Kank2    ------------HSSFSYCAPGYHTTHR------------------------------------------------------------------------
Tilapia Kank2        ------------HSSYSYCAPGYHTSQR------------------------------------------------------------------------
Amazon molly Kank2   ------------HSSYSYCAPGYHTSQR------------------------------------------------------------------------
Platyfish Kank2      ------------HSSYSYCAPGYHTLQR------------------------------------------------------------------------
Coelacanth KANK2     ------------YSSFSYCGRGFYPAYG------------------------------------------------------------------------
Anole lizard KANK2   ------------HSSYSYCGRSFYPQYP------------------------------------------------------------------------
Turtle KANK2         ------------HSSYSYCAPGFYPQCG------------------------------------------------------------------------
Tetradon Kank1b      ---------------SPFTGLGAYNQDS------------------------------------------------------------------------
Medaka Kank1b        RGRPPLPPSHISSQDSKIFNSEDPSPSR------------------------------------------------------------------------
Amazon molly Kank1b  GRPPLPPPHSSLLDDRRSKGEGPGNSQR------------------------------------------------------------------------
Platyfish Kank1b     GRPPLPPPHSSLLDDRRSKGGGPGNSQR------------------------------------------------------------------------
Frog Kank4           ---------------FKSDAQGNIAPAS------------------------------------------------------------------------
Zebrafish Kank1b     RRPPLPHSKPKTSSQRADVPQDLITCQR------------------------------------------------------------------------
Tilapia Kank1b       RRPPLPHSKPKTSSQRADVPQDLITCQR------------------------------------------------------------------------
Fugu Kank1b          RRPPLPHSKPKTSSQRADVPQDLITCQR------------------------------------------------------------------------
Cavefish Kank1b      RRPPLPPSHSLPKTTSATLESSQAPPAS------------------------------------------------------------------------
Spotted gar Kank4    -LPKNKSRALDVQQIFDFRAAESMTPES------------------------------------------------------------------------
Coelacanth KANK4     -LPAPKNKVVKTKDVLDFAAADSTTLAG------------------------------------------------------------------------
Frog Kank1           LTRRHTFSNTARPSSQEASPSFLTVPEN------------------------------------------------------------------------
Shark Kank1          IGARSQPAPPIRGGGIEASQAFLTVQEN------------------------------------------------------------------------
Coelacanth KANK1     SQPVPPSAHVKTFASFEASPTFLTVPDS------------------------------------------------------------------------
Mouse KANK1          RSHVTSTPIPRPPAPLETSPTFAVSENR------------------------------------------------------------------------
Rat KANK1            RSHVTSTPIPRPPAPLETSPTFAISENR------------------------------------------------------------------------
Dog KANK1            RSQVTSTPVPRPPAPLETSPTFLTIPES------------------------------------------------------------------------
Human KANK1          RSQVTSTPISKPPPPLETSLPFLTIPEN------------------------------------------------------------------------
Anole lizard KANK1   PAAAATSFSTRPSLSFEASPSFLTIPES------------------------------------------------------------------------
Eagle KANK1          RSQVTSSIAVRPSVTFEASPSYLTVPES------------------------------------------------------------------------
Chicken KANK1        RSQVVSSAAVRPLASLEASPSYLTVPES------------------------------------------------------------------------
Goose KANK1          RSQVTSSVAVRPLVSFEVSPSYLTVPES------------------------------------------------------------------------
Turtle KANK1         RNQVASSVTARPSVSFEASPTFLTIPES------------------------------------------------------------------------
Aligator KANK1       RSQVVSSATARPSVSFEASPAFLTIPES------------------------------------------------------------------------
Spotted gar Kank1    TRNQPNSPQVRTAVLHEASPAFLSVPEG------------------------------------------------------------------------
Herring Kank1        SLPPPPQPLTQSQTIRPRPVPIPANPVH------------------------------------------------------------------------
Zebrafish Kank1a     TSQNQVAPPGHTAQVHEVPQPFVNVPET------------------------------------------------------------------------
Cave fish Kank1a     TTRSQLASLSHIGQVHEPPQAFVNVLEA------------------------------------------------------------------------
Stickleback Kank1a   -SPVFFGGGPQVAAPLTPTLCRAACEAP------------------------------------------------------------------------
Medaka Kank1a        -SPIPFPPRPQDAAPLTCEAPVQQLPSG------------------------------------------------------------------------
Fugu Kank1a          ----------------------------------------------------------------------------------------------------
Tetradon Kank1a      ----------------------------------------------------------------------------------------------------
Tilapia Kank1a       ----------------------------------------------------------------------------------------------------
Amazon molly Kank1a  RHPDAAPLTPTLPRATGDVPLGQSYLAE------------------------------------------------------------------------
Mouse KANK3          -------------ALSPGAFPGLSLPPL------------------------------------------------------------------------
Rat KANK3            -------------ALSPGAFPGLSLPPL------------------------------------------------------------------------
```

```
Dog KANK3            -------------ALSPGAPSGLLLSPR------------------------------------------------------------------------
Human KANK3          -------------ILSQGAPSGLLMQPL------------------------------------------------------------------------
Lamprey              ---------------VDFSENFLVGAEV------------------------------------------------------------------------
Coelacanth KANK3     PRGRTQSSSLEVREPFGVQTSNPVSPAP------------------------------------------------------------------------
Frog Kank3           PRVRTQSNSSDNKEMLSFPKSNPVSPTP------------------------------------------------------------------------
Turtle KANK3         PRIRTQSGSSEMREPLSKQASNPVSPTP------------------------------------------------------------------------
Aligator KANK3       PHARTQSNSSETREALSKQVSNPVSPTA------------------------------------------------------------------------
Eagle KANK3          TTLLLSPRGRAPLEPLSKPTSHPVSPPP------------------------------------------------------------------------
Chicken KANK3        LLSPHGRAVPEPPGKQTSLPTSPPPPVR------------------------------------------------------------------------
Goose KANK3          ----------------------------------------------------------------------------------------------------
Spotted gar Kank3    VPPPRNRTISAPSESLPLSPVPLTSPPL------------------------------------------------------------------------
Zebrafish Kank3      NQPFLQSPITPPYKSQPLSPLPVISPSL------------------------------------------------------------------------
Cavefish Kank3       SHLISPSPKSQPLSPTSVCSPTLPSASK------------------------------------------------------------------------
Herring Kank3        PPPPRNRIGSAPAEVHIRSPVSVLSPPL------------------------------------------------------------------------
Stickleback Kank3    -------LGSAPCEGLRLSPVTLLGVPP------------------------------------------------------------------------
Fugu Kank3           --GPPPPPPPRNRLGSAPCEGLGLSPMA------------------------------------------------------------------------
Tetradon Kank3       -----APPPPPPRNRLGSAPCAKVPPPP------------------------------------------------------------------------
Medaka Kank3         PPPPRNRLGSAPCEVLSLSPVTLASFPS------------------------------------------------------------------------
Tilapia Kank3        ------ATVSLSPVTFLNVPPLSAGAKV------------------------------------------------------------------------
Amazon molly Kank3   PPPPRLRLGSAPCEVLSISPVTLLNVPP------------------------------------------------------------------------
Platyfish Kank3      PPPPRLRLGSAPCEVLSISPVTLLNVPP------------------------------------------------------------------------

                            410       420       430       440       450       460       470       480       490       500
                     ....|....|....|....|....|....|....|....|....|....|....|....|....|....|....|....|....|....|....|....|
Hydra                ---------------------------------------------------------------FSCQNDLIISSKNQELQTDEVILK-------------
Trichoplax           ----------------------------------------------------------------------------------------------------
Aplasia              -----------------------------------------------------TDSLSSLSSVSTVSSEAPMMHQAYLYQQQQAQHF-------------
Snail                ------------------------------------------------------SMSARTDSLTSLSSVSTMSSESHLMQQLYPQEF-------------
Fruitfly             ---------------------------TASSTCSSLRGGVESDAGIYHQRHRPAISPPETRAFLHEALDEVCSDFERTLERTSVKRR-------------
Hookworm             ------------------------------------------------------PLPPAKPCAECAPLRREVQELLKRLAVAEA----------------
C.elegance           ------------------------------------------------------MQGGYNSDYTTYRRGPPEIPRRAHFEQAKPPPL-------------
Tunicate             -------------------------------------------------------------NRQGVRADKTLMETRRRLEAFRSFRS-------------
Spider               ----------------------------------------------------VSDKRNGFKVVKDLATSETSAIMCGSMENKELYAT-------------
Lingula              ----------------------------------------------------------SPSPTSPTKFTSTMYTTNRALAGSNSSIS-------------
Lancelet             KQDGDKDGTKRLHRRSLSTGSFQMLEQQWKEQQAEQEKQKASKDAPAPSEKTVQPRSPSANGATNTYGKERILAKPRFVNQTQPIEDGPPNIKMEENDRQ
Medaka Kank4b        -----------------------------------------------------------------PRSLAELEAKIKEFDEQPLGEH-------------
Amazon molly Kank4b  -----------------------------------------------------------------LTSLTEMEARIKEFDEQPLGEH-------------
Platyfish Kank4b     ------------------------------------------------------------------LTSLTEMEARIKEFDEQPLGE-------------
Stickleback Kank4b   ----------------------------------------------------------------------------------------------------
Fugu Kank4b          -----------------------------------------------------------------LRTLSEMEARIREFDEQPLGEH-------------
Tetraon Kank4b       ----------------------------------------------------------------------------------------------------
Cave fish Kank4b     --------------------------------------------------------VRQVEPSYSSFTAAEMDATIQAFDEQPLGLH-------------
Medaka Kank4a        ------------------------------------------------------RGQTSQGIGFVPGKPREEIGAGTQGEDNAVSQS-------------
Fugu Kank4a          -------------------------------------------------------------SVTSTQSNRAAVSPAKPRDEAREGDG-------------
Tetradon Kank4a      ----------------------------------------------------------SNRAAVSPGGGFSLAKPRDEAQEGEVKTI-------------
Stickleback Kank4a   -----------------------------------------------------------IRAGHVSAKPRDELGAGAKDVEERTSGI-------------
Tilapia Kank4a       ---------------------------------------------------SSRVTTSQGTSYVSTKPKDEVGAGARGVEDKTGGIQ-------------
Platyfish Kank4a     ----------------------------------------------------SSRATTSQGTANVSTKSRDEPGTVYQGTEDKTAVI-------------
Amazon Molly Kank4a  ----------------------------------------------------SSRATTSQGTPNVSNKSRDEPGTVYQGTEDKTAVI-------------
Herring Kank4        --------------------------------------------------------SRIQGTSYSPPPRPAEEVKSRVYEEQPLSLQ-------------
Zebradish Kank4      ------------------------------------------------------SISGSSATSQSKISGSSYLPSPKVAEESQVQTS-------------
Cave fish Kank4a     ---------------------------------------------------------SGISTAGHSKVQNTGYLSSSKPADEPLVQA-------------
Mouse KANK4          ---------------------------------------------------------SVQGSELNYHRKALLAKDARQAEAGSLEDV-------------
Rat KANK4            ---------------------------------------------------------TVHGNELSYHRKALLAKDTRQVEAGSLEDA-------------
Dog KANK4            ---------------------------------------------------------SASGSEVSYHRKALLAETARHLEAAAPREA-------------
Human KANK4          ---------------------------------------------------------STSRSEVSYHRKALLAEATRQLEAAEPEDA-------------
Eagle KANK4          -------------------------------------------------------------DEPSYRGKALSAETRRPSEPGRQEGE-------------
```

```
Chicken KANK4          ------------------------------------------------------------------RRKALLAETRRHAELVRCEDE-------------
Goose KANK4            ---------------------------------------------------------------------CPLRSPSQPGDNESCPES-------------
Anole lizard KANK4     ----------------------------------------------------------------NYRRKALLIETTRQDPMKLAEEN-------------
Turtle KANK4           ----------------------------------------------------------LTAEEPSYRKKALLAETIRQAEVIHHEEE-------------
Aligator KANK4         --------------------------------------------------------------EQSYRRKALLAETIRQAEVMHPEED-------------
Frog Kank2             -----------------------------------------------------SRGGAQAPHNFNPRVEKTLLDASKKLEAGQSRPL-------------
Mouse KANK2            --------------------------------------------------------ALETRIGSNPRVERTLLDARRRLEDQAAAPS-------------
Rat KANK2              --------------------------------------------------------ALETRSGTNPRVERTLLDARRRLEDQAAAPS-------------
Dog KANK2              --------------------------------------------------------ALETRAGFNPRVERTLLDARRRLEDQAAAPT-------------
Human KANK2            --------------------------------------------------------ALETRGGFNPRVERTLLDARRRLEDQAATPT-------------
Aligator KANK2         ---------------------------------------------------------------RDARVESTLLGARRKLEGQA-----------------
Spotted gar Kank2      ----------------------------------------------------------------NARVERTLLDARKKLEEDQKDGR-------------
Cave fish Kank2        ------------------------------------------------------------PGFGAGRVEKTLLDARRKLEEEKDARR-------------
Herring Kank2          --------------------------------------------------ASSAAATMQRPSFSAARVERTLLDARRKLEEEKES---------------
Zebrafish Kank2        ------------------------------------------------------------PNFSTARVEKTLMDARRKLEEEKDGRR-------------
Tetradon Kank2         ------------------------------------------------------------PSFTTARVEKTLLDARRKLEEEKEGRR-------------
Fugu Kank2             ------------------------------------------------------------PSFSTARVEKTLLDARRKLEEEKEGRR-------------
Medaka Kank2           ------------------------------------------------------------PSFTAARVEKTLLDARRKLEEEKEGRR-------------
Sticklebakc Kank2      ------------------------------------------------------------PSFSSARVEKTLLDARRKLEEERESRR-------------
Tilapia Kank2          ------------------------------------------------------------PSFSTARVEKTLLDARRKLEEEKEGRR-------------
Amazon molly Kank2     ------------------------------------------------------------PSFSTARVEKTLLDARRKLEEEKEGRR-------------
Platyfish Kank2        ------------------------------------------------------------PSFSTARVEKTLLDARRKLEEEKEGRR-------------
Coelacanth KANK2       ----------------------------------------------------VENRPPLLTSGYNARVEKTLLDAKRRLEEDQADSA-------------
Anole lizard KANK2     ---------------------------------------------------------NTTPGSFNARVEKTLLDARKKLEDQADSGD-------------
Turtle KANK2           ------------------------------------------------------------TGAFNARVEKTLLDARKKLEDQAGDRA-------------
Tetradon Kank1b        ----------------------------------------------------------------------------KRCSDAGTEKH-------------
Medaka Kank1b          ---------------------------------------------------IPSGSKPQSAPRSSQSGVMEKTETSKLLNQDVVGQT-------------
Amazon molly Kank1b    ----------------------------------------------HTEPKPQQTERNAASQRPSLGVEQSVLETHKHISQSPPQPL-------------
Platyfish Kank1b       ----------------------------------------------HAEPKPQQTERNAASQIPSPGVEQTVLETHKNISQSPPQPL-------------
Frog Kank4             ----------------------------------------------------DSSLRLRYIHDLNYRKKENVCDTQKHIGDIVSEDI-------------
Zebrafish Kank1b       ----------------------------------------------NVEGKPTPTTQPPTPAKSNPVVEKTLMETCRRLEHEKGSLQ-------------
Tilapia Kank1b         ----------------------------------------------NVEGKPTPTTQPPTPAKSNPVVEKTLMETCRRLEHEKGSLQ-------------
Fugu Kank1b            ----------------------------------------------NVEGKPTPTTQPPTPAKSNPVVEKTLMETCRRLEHEKGSLQ-------------
Cavefish Kank1b        -------------------------------------------HRPSDGKPPPVACQPTSPTKPNPLVERTLVETRRRLEQENVLSQ-------------
Spotted gar Kank4      -----------------------------------------------------------------PRALGFSFSSVKAFDEQPLGFR-------------
Coelacanth KANK4       ---------------------------------------------------------SSNTCELNFKVGSTYQEIKGEGKEACYGEP-------------
Frog Kank1             ---------------------------------------------------KPLPPPSPQIPQHNFHVTKTLMETRRRLEQEKIIMQ-------------
Shark Kank1            ---------------------------------------------------KLLPPPSPRPPIHNFHVEKTLMETRRRLEQERLIMQ-------------
Coelacanth KANK1       ---------------------------------------------------KLLPPPSPRLPKHNFHVTKTLMETRRRLEQERLIMQ-------------
Mouse KANK1            ---------------------------------------------------QLLPPPSPQLPRHNLHVTKTLMETRRRLEQERVTMQ-------------
Rat KANK1              ---------------------------------------------------QLLPPPSPQLPRHNLHVTKTLMETRRRLEQERVTMQ-------------
Dog KANK1              ---------------------------------------------------RQLPPPSPQLPKHNLHVTKTLMETRRRLEQERVTMQ-------------
Human KANK1            ---------------------------------------------------RQLPPPSPQLPKHNLHVTKTLMETRRRLEQERATMQ-------------
Anole lizard KANK1     ---------------------------------------------------KQLFPPSPQLPRHNLHVTKTLMETRRRLEQEKMAMQ-------------
Eagle KANK1            ---------------------------------------------------KQLPPPSPQPPRHNLHVTKTLMETRRRLEQERMMQV-------------
Chicken KANK1          ---------------------------------------------------KQLPPPSPQLPRHNLHVTKTLMETRRRLEQERMMQV-------------
Goose KANK1            ---------------------------------------------------KQLPPPSPQLPRHNLHVTKTLMETRRRLEQERMMQV-------------
Turtle KANK1           ---------------------------------------------------RQLPPPSPQLPRHNLHVTKTLMETRRRLEQEKVIMQ-------------
Aligator KANK1         ---------------------------------------------------KQLPPPSPQLPRHNFHVTKTLMETRRRLEQERIMMQ-------------
Spotted gar Kank1      ---------------------------------------------------KLLPPPSPRLPRHNLHVEKTLMETRRRLEQERLLMQ-------------
Herring Kank1          ------------------------------------EAPQVYLSVLEAKQQQHLPPPSPRVPRHNPQVEKTLMETRRRLEQERLLAQ-------------
Zebrafish Kank1a       --------------------------------------------------KQLLPPPSPHLLRHNPQVEKTLMETRRRLEQERLLMQ-------------
Cave fish Kank1a       ----------------------------------------------KQQQQQLLPPPSPRLPRHNPQVEKTLMETRRRLEQERLLMQ-------------
Stickleback Kank1a     -----------------------------------------------QRPLLLPPPSPRFAPRHNPQVERTLMETRLRLEQERLLTQ-------------
Medaka Kank1a          ----------------------------------------------ATEQRLLPPPSPRFAPRHNPQVEKTLMETRRRLEQERLLLQ-------------
Fugu Kank1a            ------------------------------------------------------------------------METRLRLEQERLLMQ-------------
Tetradon Kank1a        ------------------------------------------------------------------------METRLRLEQERLLMQ-------------
```

```
Tilapia Kank1a         ------------------------------------------------------------------------METRRRLEQERALLQ-------------
Amazon molly Kank1a    ------------------------------------------------QKQLLPPPSPRFAPRHNPQVEKTLMETRRRLEQERLLVQ-------------
Mouse KANK3            ---------------------------------------------------------SPRSLSRNPRVEHTLLETSRRLEQAQARER-------------
Rat KANK3              ---------------------------------------------------------SPRSFSRNPRVEHTLLETSRRLEQAQAQER-------------
Dog KANK3              ------------------------------------------------------------VPARNPRVEHTLLETSRRLELAQAHER-------------
Human KANK3            ---------------------------------------------------------SPRAPVRNPRVEHTLRETSRRLELAQTHER-------------
Lamprey                ---------------------------------------------------------------------RSVSCDTEELENE------------------
Coelacanth KANK3       -------------------------------------------------TVRLLPPPSPKSLVRNPRVEKTLLETSRRLEEEQLSLE-------------
Frog Kank3             -------------------------------------------------VISLLPPPSPKSLLRNHRVEKTLEETSKRLEQEQLNLQ-------------
Turtle KANK3           -------------------------------------------------VINLLPPPAPKSLMRNPRVEKTLQETSRRLELEQLHLL-------------
Aligator KANK3         -------------------------------------------------VINLLPPPSPKSLLRNPRVEKTLLETSRRLEQEQLNLL-------------
Eagle KANK3            -------------------------------------------------PVRLLPPPTRKCLMRNPRVEKTLLETSRRLEQEQGHLQ-------------
Chicken KANK3          ----------------------------------------------------LLPPPARKYLARNPQVEKTLLETSRRLEQEQSRSQ-------------
Goose KANK3            ----------------------------------------------------------------------------------------------------
Spotted gar Kank3      ---------------------------------------------------SAGPRGPPPPPIRNPRVERTLLETSRRLEQEQSHLQ-------------
Zebrafish Kank3        ---------------------------------------------------PTFSKVPPPPPPRNPRVERTLLETSLRLQQEQSQFS-------------
Cavefish Kank3         --------------------------------------------------------IPPPPPPRNPRVERTLLETSRRLQQEQSFFP-------------
Herring Kank3          ---------------------------------------------------SAGAKVPPPPPARNPRVERTLLETSLRLQQEQSQLQ-------------
Stickleback Kank3      --------------------------------------------------LSAGAKVPPPPPQRNPRVERTLLETSRRLQHEQTHQH-------------
Fugu Kank3             --------------------------------------------------------------LRNPRVERTLLETSRRLQQEQTHQH-------------
Tetradon Kank3         -------------------------------------------------------------PQRNPRVERTLLETSRRLQQEQTHQH-------------
Medaka Kank3           ----------------------------------------------------ISAKVPPPPPQRNPRVERTLLETSRRLQQEQTHQH-------------
Tilapia Kank3          ---------------------------------------------------------PPPPPQRNPRVERTLLETSRRLQQEQTHQN-------------
Amazon molly Kank3     --------------------------------------------------LSAGAKVPPPPPQRNPRVERTLLETSRRLQQEQTHQH-------------
Platyfish Kank3        --------------------------------------------------LSAGAKVPPPPPQRNPRVERTLLETSRRLQQEQTHQH-------------

                                510       520       530       540       550       560       570       580       590       600
                       ....|....|....|....|....|....|....|....|....|....|....|....|....|....|....|....|....|....|....|....|
Hydra                  -----------------------------------------------------------------------------------------ESSVGDDVAEK
Trichoplax             ----------------------------------------------------------------------------------------------------
Aplasia                --------------------------------------------------------------------------------------QHSKTTKTSTEVIE
Snail                  --------------------------------------------------------------------------------------QYTVNHKVTSKIIE
Fruitfly               --------------------------------KSPYGAGSLSMDRNNNKLSLSISNGHGALKELKSHRLGNSTWDRHFDVADSCIGGNQSPTGTPRPFLR
Hookworm               ----------------------------------------------------------------------------------------------KPTVVS
C.elegance             ---------------------------------------------------------------------------------------------SRPINLG
Tunicate               --------------------------------------------------------------------------------------------KRTKAFGE
Spider                 -----------------------------------------------------------------------------------------TFPAVQSRSSS
Lingula                ---------------------------------------------------------------------------------------------------S
Lancelet               QANKDNITRPTNLENTTSSANVVRTVEPQTNVEKPADAESGFGDMESDTPEANPKVDLSPDGKCTCCPYGYHIDLDFLKYCDDISKGSNIPKIVRPRKPK
Medaka Kank4b          ---------------------------------------------------------------------------------------------IRPHLLR
Amazon molly Kank4b    ---------------------------------------------------------------------------------------------IRPHLLR
Platyfish Kank4b       --------------------------------------------------------------------------------------------HIRPHLLR
Stickleback Kank4b     ----------------------------------------------------------------------------------------------------
Fugu Kank4b            ---------------------------------------------------------------------------------------------IRPNLLR
Tetraon Kank4b         ----------------------------------------------------------------------------------------------------
Cave fish Kank4b       ---------------------------------------------------------------------------------------------VRPNLLR
Medaka Kank4a          ----------------------------------------------------------------------------------------------RPNLLR
Fugu Kank4a            ---------------------------------------------------------------------------------------RTIPGQARPNLLR
Tetradon Kank4a        ------------------------------------------------------------------------------------------LGQTRPNLLR
Stickleback Kank4a     --------------------------------------------------------------------------------------------QSRPNLLR
Tilapia Kank4a         -------------------------------------------------------------------------------------------IQSRPNLLR
Platyfish Kank4a       --------------------------------------------------------------------------------------------QSRPNLLR
Amazon Molly Kank4a    --------------------------------------------------------------------------------------------QSRPNLLR
Herring Kank4          ---------------------------------------------------------------------------------------------LRPNLLR
Zebradish Kank4        -----------------------------------------------------------------------------------SQNEQSIGLSVRPHLLR
Cave fish Kank4a       -----------------------------------------------------------------------------------SYKEQQLSLNVRPHLFR
Mouse KANK4            -------------------------------------------------------------------------------------------GSGRPQLLR
```

```
Rat KANK4               -------------------------------------------------------------------------------------------GSGRPQLLR
Dog KANK4               ---------------------------------------------------------------------------------------ETASGSGRPQLLR
Human KANK4             ---------------------------------------------------------------------------------------ELTFGSGRPQLLR
Eagle KANK4             -------------------------------------------------------------------------------------------LRGRPQLLR
Chicken KANK4           -------------------------------------------------------------------------------------------LRGRPQLLR
Goose KANK4             --------------------------------------------------------------------------------------------TFRPALRS
Anole lizard KANK4      -----------------------------------------------------------------------------------------GSCRGRPQLLR
Turtle KANK4            ----------------------------------------------------------------------------------------PGSCRGRPQLLR
Aligator KANK4          ----------------------------------------------------------------------------------------LASCRGRPQLLR
Frog Kank2              ----------------------------------------------------------------------------------------------------
Mouse KANK2             ----------------------------------------------------------------------------------------------------
Rat KANK2               ----------------------------------------------------------------------------------------------------
Dog KANK2               ----------------------------------------------------------------------------------------------------
Human KANK2             ----------------------------------------------------------------------------------------------------
Aligator KANK2          ----------------------------------------------------------------------------------------------------
Spotted gar Kank2       ------------------------------------------------------------------------------------------------RFSN
Cave fish Kank2         -------------------------------------------------------------------------------------------------FSN
Herring Kank2           -----------------------------------------------------------------------------------------------RRFSH
Zebrafish Kank2         -------------------------------------------------------------------------------------------------FSN
Tetradon Kank2          -------------------------------------------------------------------------------------------------YSN
Fugu Kank2              -------------------------------------------------------------------------------------------------YSN
Medaka Kank2            -------------------------------------------------------------------------------------------------FSN
Sticklebakc Kank2       -------------------------------------------------------------------------------------------------FST
Tilapia Kank2           -------------------------------------------------------------------------------------------------FSN
Amazon molly Kank2      -------------------------------------------------------------------------------------------------FSN
Platyfish Kank2         -------------------------------------------------------------------------------------------------FSN
Coelacanth KANK2        -------------------------------------------------------------------------------------------EDRRSRFAS
Anole lizard KANK2      --------------------------------------------------------------------------------------------GERSRVSG
Turtle KANK2            ---------------------------------------------------------------------------------------------------G
Tetradon Kank1b         ----------------------------------------------------------------------------------------------------
Medaka Kank1b           ----------------------------------------------------------------------------------------PPQPLPRRRLAS
Amazon molly Kank1b     ---------------------------------------------------------------------------------------------PRRRLAS
Platyfish Kank1b        ---------------------------------------------------------------------------------------------PRRRLAS
Frog Kank4              -----------------------------------------------------------------------------------------NRCADRPQFVR
Zebrafish Kank1b        --------------------------------------------------------------------------------------QAPPEPQPRRRLAS
Tilapia Kank1b          --------------------------------------------------------------------------------------QAPPEPQPRRRLAS
Fugu Kank1b             --------------------------------------------------------------------------------------QAPPEPQPRRRLAS
Cavefish Kank1b         --------------------------------------------------------------------------------------AVPQELCPRRRLAS
Spotted gar Kank4       ---------------------------------------------------------------------------------------------VRPQLLR
Coelacanth KANK4        ----------------------------------------------------------------------------------------SALSRGRPQLLR
Frog Kank1              -------------------------------------------------------------------------------------VASSYNDIRRPRLAS
Shark Kank1             ---------------------------------------------------------------------------------------STSGDARRPRFAS
Coelacanth KANK1        ---------------------------------------------------------------------------------------SGPGDVRRPRLAS
Mouse KANK1             ---------------------------------------------------------------------------------------MAPGDFRRPRLAS
Rat KANK1               ---------------------------------------------------------------------------------------MAPGEFRRPRLAS
Dog KANK1               ---------------------------------------------------------------------------------------VAPGEFRRPRLAS
Human KANK1             ---------------------------------------------------------------------------------------MTPGEFRRPRLAS
Anole lizard KANK1      ---------------------------------------------------------------------------------------ASPGDTRRPRLSS
Eagle KANK1             ----------------------------------------------------------------------------------------TPGDVRRPRLSS
Chicken KANK1           ----------------------------------------------------------------------------------------MPGDVRRPRLSS
Goose KANK1             ----------------------------------------------------------------------------------------TPGDVRRPRLSS
Turtle KANK1            ---------------------------------------------------------------------------------------VTPGDVRRPRLSS
Aligator KANK1          ---------------------------------------------------------------------------------------VTPGDVCRPRLSS
Spotted gar Kank1       ---------------------------------------------------------------------------------------PPSSEARRPRLAS
Herring Kank1           --------------------------------------------------------------------------------------PPPCDPPPRRRLAS
Zebrafish Kank1a        ---------------------------------------------------------------------------------------PPVSEPPRRRLAS
Cave fish Kank1a        ---------------------------------------------------------------------------------------PPASEPPRRRLAS
```

```
Stickleback Kank1a      ----------------------------------------------------------------------------------------------PPSLAS
Medaka Kank1a           --------------------------------------------------------------------------------------PHTEVLMPRRRLAS
Fugu Kank1a             --------------------------------------------------------------------------------------PHVDPPMPRRRLAS
Tetradon Kank1a         --------------------------------------------------------------------------------------PHVDPPLPRRRLAS
Tilapia Kank1a          --------------------------------------------------------------------------------------PLSEPPMPRRRLAS
Amazon molly Kank1a     --------------------------------------------------------------------------------------AHNEPLIPRRRLAS
Mouse KANK3             ----------------------------------------------------------------------------------------------------
Rat KANK3               -----------------------------------------------------------------------------------------ALSPARAATRS
Dog KANK3               ----------------------------------------------------------------------------------------------------
Human KANK3             ----------------------------------------------------------------------------------------------------
Lamprey                 ----------------------------------------------------------------------------------------------------
Coelacanth KANK3        ----------------------------------------------------------------------------------------NIGDVQRPRLAS
Frog Kank3              ------------------------------------------------------------------------------------GAEGQIRTCSSTSLSK
Turtle KANK3            -------------------------------------------------------------------------------------------SSGDAQMTG
Aligator KANK3          -------------------------------------------------------------------------------------------SSGNAQIPK
Eagle KANK3             -------------------------------------------------------------------------------------------DIGGPSRSG
Chicken KANK3           ----------------------------------------------------------------------------------------------------
Goose KANK3             ----------------------------------------------------------------------------------------------------
Spotted gar Kank3       -----------------------------------------------------------------------------------NGSGFQLAEPPRAGSKQ
Zebrafish Kank3         -------------------------------------------------------------------------------------------NGLGSNLAD
Cavefish Kank3          ----------------------------------------------------------------------------------------NGLGLPASDLSR
Herring Kank3           ----------------------------------------------------------------------------------NGTGTIQLADPPRGALRE
Stickleback Kank3       ----------------------------------------------------------------------------------------------------
Fugu Kank3              ------------------------------------------------------------------------------------------QNGGRFQLAD
Tetradon Kank3          ----------------------------------------------------------------------------------------------------
Medaka Kank3            ----------------------------------------------------------------------------------------------------
Tilapia Kank3           ----------------------------------------------------------------------------------------------------
Amazon molly Kank3      ----------------------------------------------------------------------------------------------------
Platyfish Kank3         ---------------------------------------------------------------------------------------------------Q

                               610       620       630       640       650       660       670       680       690       700
                        ....|....|....|....|....|....|....|....|....|....|....|....|....|....|....|....|....|....|....|....|
Hydra                   FYSNSFTQ-----------------------------------------------------------------VLKNYKNQFTQCHLTEYLD--------
Trichoplax              ----------------------------------------------------------------------------------------------------
Aplasia                 NPVLEMAT----------------------------------------------------------------AEMKYATNPVVEMGTAEMKPLPPPPPVK
Snail                   TPVIEMAT-----------------------------------------------------------------AEVKLKETPVIERGTAEVKPKETSVVE
Fruitfly                SNTLPLRQ------------------------------------------RPSPAEVQYESYVQATVAANAKSPVEQLESAKSPGAPPPPPPRRHHLLAT
Hookworm                IGTATEEV----------------------------------------------------------------QRVEAAVGEPTQC---------------
C.elegance              FSGSRSNT-----------------------------------------------------------------PTQRFNAPNGTGNGYSTALAKT-----
Tunicate                EPGLPPHN----------------------------------------------------------------YAVRNGVSSQTLHNMRRGSGS-------
Spider                  MSSVSSQS------------------------------------------------------------------TASILGPVPDASRNSCMSD-------
Lingula                 QTTVNSEM----------------------------------------------------------------SAVNGSPGPERYTTVTAQ----------
Lancelet                SPGGPAPMDFRANTLPRGFYLNSQQSNLPKHASVVPSSPISPLSVNTSLSSANNSIPETTTTTATTTKQTVTDPRENASSVYGYNSSTRRPSLPGDLPIP
Medaka Kank4b           ASSLPLTV----------------------------------------------------------------LLRQESECADDRSSIWSSREY-------
Amazon molly Kank4b     ASSLPLTV----------------------------------------------------------------LLRQESESTDDRSSVRSSRD--------
Platyfish Kank4b        ASSLPLTV----------------------------------------------------------------LLRQESESTDDRSSIQSSRD--------
Stickleback Kank4b      ----------------------------------------------------------------------------------------------------
Fugu Kank4b             ASSLPLTV----------------------------------------------------------------LLRQSSESSDDPSSLRGSRDHLEG----
Tetraon Kank4b          ----------------------------------------------------------------------------------------------------
Cave fish Kank4b        ASSLPLTV----------------------------------------------------------------LLRKRSESIEDPTSPNGSRDYLTQ----
Medaka Kank4a           ASSMPITL-----------------------------------------------------------------QQRKGSDSSSPDCTTGTPE--------
Fugu Kank4a             ASSMPITL-----------------------------------------------------------------QQRKGSDSSSPDRTVGTPE--------
Tetradon Kank4a         ASSMPITL-----------------------------------------------------------------QQRKGSDSSSPDRTVGTPE--------
Stickleback Kank4a      ASSMPITL-----------------------------------------------------------------QQRKASDSSSPDRVVGTPE--------
Tilapia Kank4a          ASSMPITL-----------------------------------------------------------------QQRKSSDSSSPDRTVGTPE--------
Platyfish Kank4a        ASSMPITL-----------------------------------------------------------------QQRKGSDSSSPDCTTGTPE--------
Amazon Molly Kank4a     ASSMPITL-----------------------------------------------------------------QQRKGSDSSSPDCTTGTPE--------
```

```
Herring Kank4        TSSMPGNV----------------------------------------------------------------PRRKGSDSSEDRSPVALGESQ-------
Zebradish Kank4      ASSMPVNV----------------------------------------------------------------PRRKGSDSTDEQSSQ-------------
Cave fish Kank4a     ASSMPVNV-----------------------------------------------------------------PQRRLSDSTDEQSPQ------------
Mouse KANK4          ASSMPATL------------------------------------------------------------------LQNQVPEEPSLTSGPST---------
Rat KANK4            ASSMPATL-----------------------------------------------------------------PQNQAPEVPNLTSGPSA----------
Dog KANK4            ASSMPATL-----------------------------------------------------------------LQNKASEDSSLNSGSPM----------
Human KANK4          ASSMPATL-----------------------------------------------------------------LHSRASEEPGLSLGPPA----------
Eagle KANK4          ASSMPAAL----------------------------------------------------------------PPGQPPPPPGDGKVPSPLRPPPQHCHD-
Chicken KANK4        ASSLPAAL---------------------------------------------------------------PPGQAPASDGCVPCPLRPS----------
Goose KANK4          PDGITSSL-----------------------------------------------------------------PQDAF----------------------
Anole lizard KANK4   ASSMPATL----------------------------------------------------------------PPNQNSEEEETPLTFLQS----------
Turtle KANK4         ASSMPAKL----------------------------------------------------------------LPSKSSEEESQCSKPSQPSALSQPC---
Aligator KANK4       ASSMPATL----------------------------------------------------------------PPTKNAEEESQCSEPSQLVLLPQPC---
Frog Kank2           --SLGCRS-------------------------------------------------------------------TSSLSTQSLSRFP------------
Mouse KANK2          SGGLGSLT-------------------------------------------------------------------PSAAGSTS-----------------
Rat KANK2            SGGLGSLT-------------------------------------------------------------------PSAAGSTS-----------------
Dog KANK2            --GLGSLT-------------------------------------------------------------------PSAAGSTS-----------------
Human KANK2          --GLGSLT-------------------------------------------------------------------PSAAGSTA-----------------
Aligator KANK2       ----------------------------------------------------------------------------------------------------
Spotted gar Kank2    LGSLHSSI----------------------------------------------------------------AGSTSSLASQGYSRAQNG----------
Cave fish Kank2      LGSMHGSM----------------------------------------------------------------AGSNTSLSSAHSFN--------------
Herring Kank2        LGSMHSSV----------------------------------------------------------------AGSTTSLSSAHSYNRSHGGGGGGG----
Zebrafish Kank2      LGSMHSSM----------------------------------------------------------------AGSNTSLSSAHS----------------
Tetradon Kank2       LGSMNSSV----------------------------------------------------------------AGSNTSLSSAHSF---------------
Fugu Kank2           LGSMNSSV----------------------------------------------------------------AGSNTSLSSAHSF---------------
Medaka Kank2         LGSMHSSV----------------------------------------------------------------AGSNTSISSAHSF---------------
Sticklebakc Kank2    LGSMHSSV----------------------------------------------------------------AGSNTSLSSAHSF---------------
Tilapia Kank2        LGSMHSSV----------------------------------------------------------------AGSNTSISSAHSF---------------
Amazon molly Kank2   LSSMNNSM----------------------------------------------------------------AGSNTSLSSAHSF---------------
Platyfish Kank2      LGSMNNSM----------------------------------------------------------------AGSNTSLSSAHSF---------------
Coelacanth KANK2     LGSLHGSV----------------------------------------------------------------SGSTSSLSGAGLSQSLSKMSSHS-----
Anole lizard KANK2   LGSLHSSV---------------------------------------------------------------SGSTSSLVGSQSLSRQTSYN---------
Turtle KANK2         PGGLGSLH--------------------------------------------------------------------SSVSGSTSSLVGGQPP--------
Tetradon Kank1b      -AGLSSSL--------------------------------------------------------------------------------------------
Medaka Kank1b        FGGVSSPG-----------------------------------------------------------------SVSPFTGLNAYNQSNNNHSSDADVHLG
Amazon molly Kank1b  FGGISPSG-----------------------------------------------------------------SHSPFTGMGAYNQNNNGNKPDDDMSAH
Platyfish Kank1b     FGGISPSG-----------------------------------------------------------------SHSPFTGLGAYNQNNNGNKPDDDMSAH
Frog Kank4           ASSMPAAL-------------------------------------------------------------------PQSTGHYEQNKY-------------
Zebrafish Kank1b     FGGVGSSG-----------------------------------------------------------------TLSPYTSWSALNQGAKMTVLDQPRER-
Tilapia Kank1b       FGGVGSSG-----------------------------------------------------------------TLSPYTSWSALNQGAKMTVLDQPRER-
Fugu Kank1b          FGGVGSSG-----------------------------------------------------------------TLSPYTSWSALNQGAKMTVLDQPRER-
Cavefish Kank1b      FGGVSSSG-----------------------------------------------------------------TLSPYTSWNALNQAQTQALSKRSVGDQ
Spotted gar Kank4    TSSLPVTV----------------------------------------------------------------LQRKNSEPSEDQAPPPIRSSNKPNGT--
Coelacanth KANK4     ASSMPVTV----------------------------------------------------------------LQRKDSEEQCSTPTDVQHS---------
Frog Kank1           FGGMGSTG-----------------------------------------------------------------SLSSVTGTTGHNISAQQLQ-----NGH
Shark Kank1          FGGMGSTS-----------------------------------------------------------------SLSSFTGSTGLNQVSPNSQ--QVHNGF
Coelacanth KANK1     LGAMGSTS-----------------------------------------------------------------SLSSFTGSSGHNHISPTSQQLQ--NGY
Mouse KANK1          FGGMGSTS-----------------------------------------------------------------SLPSFVGSANHSSAIHQLQ-----NGY
Rat KANK1            FGGMGSTS-----------------------------------------------------------------SLPSFMGSGNHSSAMHQLQ-----NGY
Dog KANK1            FGGMGSTS-----------------------------------------------------------------SLSSFLGSGNHNPAMHQLQ-----NGY
Human KANK1          FGGMGTTS-----------------------------------------------------------------SLPSFVGSGNHNPAKHQLQ-----NGY
Anole lizard KANK1   FGAMGSTG-----------------------------------------------------------------SLPSFVGSSGPNQVSHPFQ-----NGY
Eagle KANK1          FGGMGSTS-----------------------------------------------------------------SLPSFVGSSGYGHMSQQLQ-----NGY
Chicken KANK1        FGGMGSTS-----------------------------------------------------------------SLPSFVGSSGYGHLPQQLH-----NGY
Goose KANK1          FGGMGSTS-----------------------------------------------------------------SLPSFVGSGGYGHMSQQLQ-----NGY
Turtle KANK1         FGGLGSAS-----------------------------------------------------------------SLPSFVGSSGHSQMSQQLQ-----NGY
Aligator KANK1       FGGLGSAS-----------------------------------------------------------------SLPSFVGSSGHNQVSPQLQ-----NGC
```

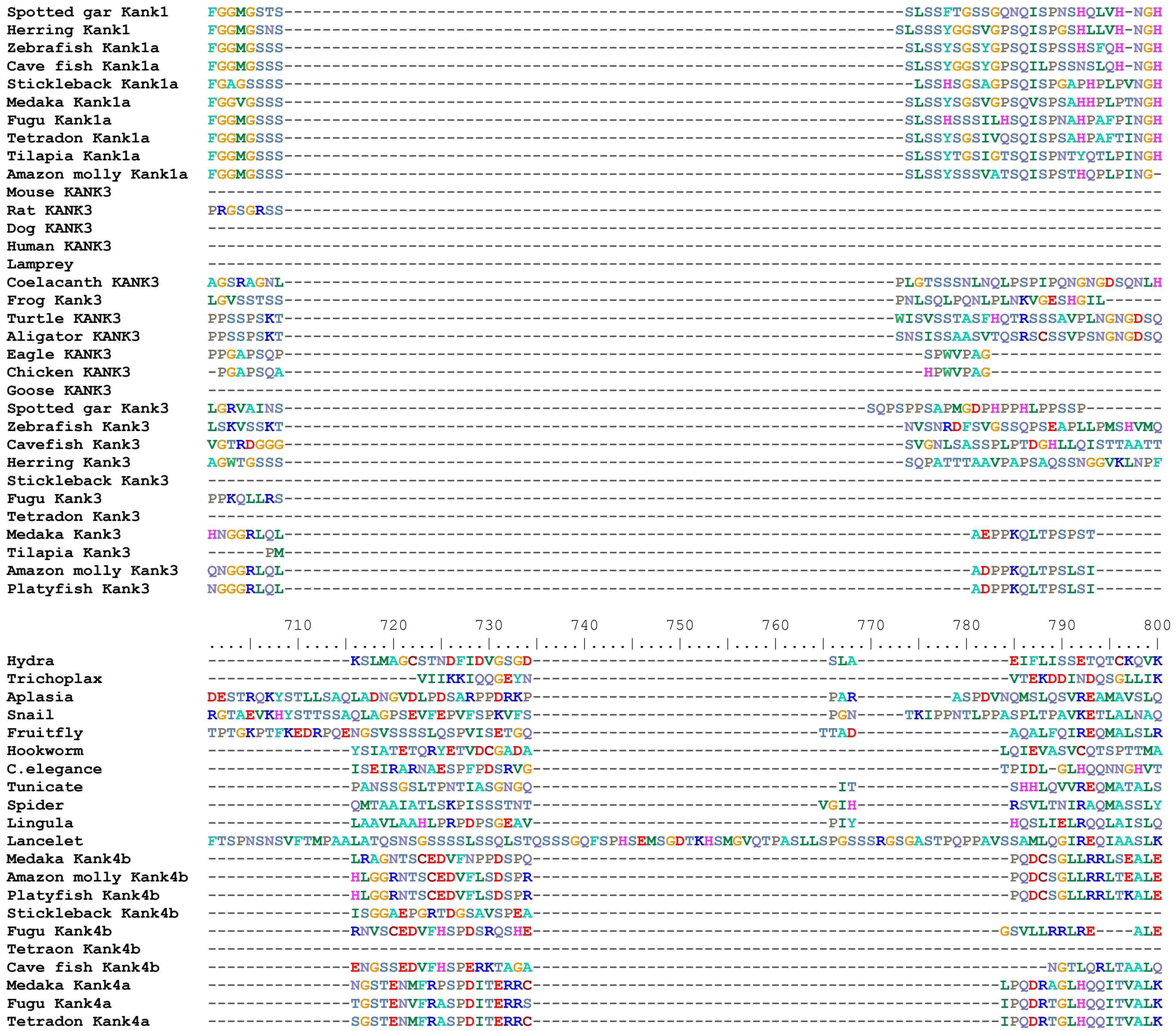
Spotted gar Kank1
Herring Kank1
Zebrafish Kank1a
Cave fish Kank1a
Stickleback Kank1a
Medaka Kank1a
Fugu Kank1a
Tetradon Kank1a
Tilapia Kank1a
Amazon molly Kank1a
Mouse KANK3
Rat KANK3
Dog KANK3
Human KANK3
Lamprey
Coelacanth KANK3
Frog Kank3
Turtle KANK3
Aligator KANK3
Eagle KANK3
Chicken KANK3
Goose KANK3
Spotted gar Kank3
Zebrafish Kank3
Cavefish Kank3
Herring Kank3
Stickleback Kank3
Fugu Kank3
Tetradon Kank3
Medaka Kank3
Tilapia Kank3
Amazon molly Kank3
Platyfish Kank3
710 720 730 740 750 760 770 780 790 800
Hydra
Trichoplax
Aplasia
Snail
Fruitfly
Hookworm
C.elegance
Tunicate
Spider
Lingula
Lancelet
Medaka Kank4b
Amazon molly Kank4b
Platyfish Kank4b
Stickleback Kank4b
Fugu Kank4b
Tetraon Kank4b
Cave fish Kank4b
Medaka Kank4a
Fugu Kank4a
Tetradon Kank4a

```
Stickleback Kank4a     ---------------SGSTENVFRASPDITERRC-------------------------------------------------IPQDRTGLHQQITVALK
Tilapia Kank4a         ---------------NGSTENMFRASPDVTERRC-------------------------------------------------VPQDRAGLHQQITVALK
Platyfish Kank4a       ---------------NGSTENMFRASPDILERRC-------------------------------------------------LPQDRAGLHQQITVALK
Amazon Molly Kank4a    ---------------NGSTENMFRASPDILERRC-------------------------------------------------LPQDRAGLHQQITVALK
Herring Kank4          ---------------KENGSYERLFRADEGGDRR-------------------------------GSV----------------PQDRASLHVQITNALK
Zebradish Kank4        ---------------SQNGSTEKLFRPADGGDRR-------------------------------GSI----------------PQDRASLHQQITAALK
Cave fish Kank4a       ---------------SQNGSTERLFRPVESSDRR-------------------------------GSV----------------PQDRASLHQQITAALK
Mouse KANK4            ---------------LLALPLLQDEGSVCDGAFD-------------------------------PAE---------------------GLMGFQASAQS
Rat KANK4              ---------------LLVLPPLQDEGSVRDGTFD-------------------------------PAE---------------------GLMGFQAAAQS
Dog KANK4              ---------------PPALPLLQSEGSVCDGTFG-------------------------------PAD----------------GFAGFQNSTPRATTQP
Human KANK4            ---------------PPALPPLQGEGSVCDGTFE-------------------------------PAE------------------GLAGFHSSSPRAST
Eagle KANK4            ---------------RSGSESAFRPAPRSPGSDG---------------------------------------------------AALSLAREASSEPGV
Chicken KANK4          ---------------DNGSCPESTFRPMPRSPDG-------------------------------IAS---------------LPSLMLSPGAELASQQQ
Goose KANK4            ---------------LQQQEQPGKGSPVREGWHG-------------------------------EVP--------------------------------
Anole lizard KANK4     ---------------CDAKSLENSFSPANESPES-------------------------------ESS----------------KGVSLTAPDGLMTVKQ
Turtle KANK4           ---------------DGKSFTEIPFSPAKELLDS-------------------------------HYS---------DSVTLHFPKEPRNVQEQIKVVFQ
Aligator KANK4         ---------------DGKSFTEIPFSPAKESLDS-------------------------------NCP---------DSLSLNLPKDTRIAQQQMEEAMP
Frog Kank2             ---------------STSSQQISSFTPLSSGMST-------------------------------PVS--------------PSPAQLQHVREQMAVALK
Mouse KANK2            ---------------SLAGVGLLPPTPRSSGLST-------------------------------PVA---------PS-----AGHLAHVREQMAGALR
Rat KANK2              ---------------SLAGVGLLPPTPRSSGLST-------------------------------PVA---------PS-----AGHLAHVREQMAGALR
Dog KANK2              ---------------SLVGVGLPPPTPRGSGLST-------------------------------PVP--------------PSAGHLAHVREQMAGALR
Human KANK2            ---------------SLVGVGLPPPTPRSSGLST-------------------------------PVP--------------PSAGHLAHVREQMAGALR
Aligator KANK2         ---------------GPSGLRNSRLSLAGSGLST-------------------------------PAT--------------PTPGHLQHVREQMAGALR
Spotted gar Kank2      ---------------AQGAGGGGQFTPMSSGLST-------------------------------PVS---------PT-----PAHLQHVREQMAVALR
Cave fish Kank2        ---------------RAQGGAGSSYTPASSGLST-------------------------------PVS---------PT-----PAHLQHVREQMALALR
Herring Kank2          ---------------GGGGGGGGSYTPMSSGLST-------------------------------PVS---------PT-----PAHLQHVREQMASALR
Zebrafish Kank2        ---------------FNRAQGGGSYTPMSSGLST-------------------------------PVS--------------PTPAHLQHVREQMAVALR
Tetradon Kank2         ---------------NRAHGGSGSFTPINSGLST-------------------------------PVT--------------PTPAHLQHVREQMATALR
Fugu Kank2             ---------------NRAHGGSGSFTPINSGLST-------------------------------PVT--------------PTPAHLQHVREQMASALR
Medaka Kank2           ---------------NRAQGGGGSVTNLSSGLST-------------------------------PVT--------------PTPAHLQHVREQMAAALR
Sticklebakc Kank2      ---------------NRAHGGGGSYTPMSSGLST-------------------------------PVT--------------PTPAHLQHVREQMATALR
Tilapia Kank2          ---------------NRSQGGGGSFTPMSSGLST-------------------------------PVT--------------PTPAHLQHVREQMAAALR
Amazon molly Kank2     ---------------NRAHGGGGTFTPMSSGLST-------------------------------PVT---------PT-----PAHLQHVREQMAAALR
Platyfish Kank2        ---------------NRAHGGGGTFTPMSSGLST-------------------------------PVT--------------PTPAHLQHVREQMAAALR
Coelacanth KANK2       ---------------NSQSGVSGQYTPMSSGLST-------------------------------PVS---------PT-----PAHLQHVREQMAVALR
Anole lizard KANK2     ---------------GSQLGASGQFTPIGSGLST-------------------------------PVS---------PT-----PGHLQHVREQMAVALK
Turtle KANK2           ---------------GRLNGSQAGFTPLSSGLST-------------------------------PVS--------------PSPGHLQHVREQMAVALK
Tetradon Kank1b        --------------GSRGSTGCLRLSPESSGRTT-------------------------------PVP---------NLG----PTQLQHVRDQMVVALQ
Medaka Kank1b          SSLG-----------SRGSTGCLQLSPQSSGRST-------------------------------PVS---------------GPMHLQHVRDQMVVALQ
Amazon molly Kank1b    LTSSLG---------SRGSTGCLKLSPQSSGRST-------------------------------PVT---------SLG----SMHLQHVRDQMVVALK
Platyfish Kank1b       LTSSLG---------SRGSTGCLTLSPQSSGRNT-------------------------------PVT---------SLG----SMHLQHVRDQMVVALK
Frog Kank4             ---------------LKFPQSVNSYSESTTQCLE--------------------------------------------------GSHHKGSEDQTAVALK
Zebrafish Kank1b       ---------------TLILGSALKISPSSSGRAS-------------------------------PAS-------------GVSPLHLQMVRDQMATALQ
Tilapia Kank1b         ---------------TLILGSALKISPSSSGRAS-------------------------------PAS-------------GVSPLHLQMVRDQMATALQ
Fugu Kank1b            ---------------TLILGSALKISPSSSGRAS-------------------------------PAS-------------GVSPLHLQMVRDQMATALQ
Cavefish Kank1b        SSQGQGA--------STMSSTSMRISPLSSGRAT-------------------------------PVT---------SVS----PLHLQLVRDQMVVALE
Spotted gar Kank4      ---------------SENSFWSLNASPSPDNKHQ-------------------------------LTA----------------QKEMSSLHQQFTNALQ
Coelacanth KANK4       ---------------NRTSCVDNIFNPANESISQ-------------------------------NSS---------KNFVLTKSEEGTNIKE-FQIALQ
Frog Kank1             QANGDYSALF-----ASSLGSSIRHSPLSSGVST-------------------------------PVT---------NVS----PMHLQHIREQMATALK
Shark Kank1            QTNGDYNPYF-----TSSMGSSIRHSPLSSGLTT-------------------------------PVT---------NVT----PTHLQHIREQMVVALR
Coelacanth KANK1       QANGEYNPYF-----TSSIGSSIRHSPMSSGMTT-------------------------------PVS---------NVS----PMHLQHIREQMVVALK
Mouse KANK1            QGNGDYNSYVPAAPTTSSMGSSVRHSPLSSGIST-------------------------------PVT---------NVS----PMHLQHIREQMAIALK
Rat KANK1              QGNGDYSSYVPAVPTTSSMGSSVRHSPLSSGIST-------------------------------PVT---------NVS----PMHLQHIREQMAIALK
Dog KANK1              QGNGDYGGYVPATATTSSMGSSIRHSPLSSGIST-------------------------------PVT---------NVS----PMHLQHIREQMAVALK
Human KANK1            QGNGDYGSYAPAAPTTSSMGSSIRHSPLSSGIST-------------------------------PVT---------NVS----PMHLQHIREQMAIALK
Anole lizard KANK1     QGNGDYSLHL-----ASSLGSSIRHSPLSSGIST-------------------------------PVT---------NVS----PVHLQHIREQMAIALK
Eagle KANK1            QGNGDYGACF-----STSLGSSIRHSPMSSGIST-------------------------------PVT---------NVS----PVHLQHIREQMAVALK
```

```
Chicken KANK1        QGNGDFGACF-----GSSLGSSIRHSPMSSGIST-------------------------------PVT---------NVS----PVHLQQIREQMAIALK
Goose KANK1          QGSGDYGACF-----SSSLGSSIRHSPMSSGIST-------------------------------PVT---------NVS----PVHLQHIREQMAVALK
Turtle KANK1         QGNGDYNVYL-----ASSLGSSIRHSPLSSGIST-------------------------------PVT---------NVS----PVHLQHIREQMAVALK
Aligator KANK1       QGNGDYNVCL-----GFSLNSSIRHSPMSSGIST-------------------------------PVT---------NVS----PVHLQHIREQMAVALK
Spotted gar Kank1    QANGEYNPYF-----TSSMGSSIRHSPMSSGMTT-------------------------------PVT---------NIS----PMHLQQIREQMVVALK
Herring Kank1        LPNGDYNPYF-----TPSMVSSMRHSPMSSGMTT-------------------------------PVT---------NVS----PAHLQHIRDQMVVALK
Zebrafish Kank1a     LGNGEYNPYF-----TSSMGSSIRHSPLSSGMTT-------------------------------PVT---------NVS----PLHLQHIREQMVVALK
Cave fish Kank1a     LANGEYNLYL-----TSSMGSSIRHSPLSSGMTT-------------------------------PVT---------NIS----PLHLQNIRDQMMVALK
Stickleback Kank1a   PPNGDYSLYY-----PSSMVSSIRHSPLSSGVAT-------------------------------PVT---------NVS----PLHLQQIREQMVVALR
Medaka Kank1a        LPNGDYGTYF-----PPSVGSSIRHSPLSSGVAT-------------------------------PIT---------NVS----PLHLQQIREQMVVALR
Fugu Kank1a          PHNGEYNPYY-----PASMGSSIRHSPLSSGMTT-------------------------------PVT---------NVS----PLHLHQIREQMVVALR
Tetradon Kank1a      PHNGEYNPYY-----PASMGSSIRHSPLSSGMAT-------------------------------PVT---------NVS----PLHLQQIREQMVVALR
Tilapia Kank1a       LPNGEYNPYY-----PPSRGSSIRHSPMSSGVAT-------------------------------PVT---------NVS----PLHLQQIREQMVVALK
Amazon molly Kank1a  LSNGEYNPYH-----SQSRGSSIRHSPMSSGVAT-------------------------------PVT---------NVS----PLHLQQIREQMVVALK
Mouse KANK3          ---------------ALSPARAVTRSPRGSGRSS-------------------------------PAP--------NPALASPGPAQLQLVREQMAAALR
Rat KANK3            ---------------PAPNPALASPSPVLASPG---------------------------------------------------PAQLQLVREQMAVALR
Dog KANK3            ---------------APSPGRAVPRSPRGSGRSS-------------------------------PAP------NPAPAPASPGPAQLQLVREQMAAALR
Human KANK3          ---------------APSPGRGVPRSPRGSGRSS-------------------------------PAP--------NLAPASPGPAQLQLVREQMAAALR
Lamprey              ---------------AEGNEGARAGSEVARGAEL--------------------------------------------------SRYIERVQRLLHEQQA
Coelacanth KANK3     ---------------SPSFTSSIRISPMNSGRST-------------------------------PVT---------SIT----PAQLQHVREQMVTALK
Frog Kank3           ---------------SPSFSGSVKISPVNSGRST-------------------------------PAM--------------ISSSHLHYVREQMAAALK
Turtle KANK3         ATP------------GPSLTGSMRMSPLNSGRST-------------------------------PAT---------NIS----PAHLQHVREQMAAALK
Aligator KANK3       ATL------------SPSLTGSMRMSPFSSGWTT-------------------------------PAT---------NLS----ATHLQHVREQMAAALK
Eagle KANK3          ---------------TEGQAGWGRASPGSSGRST-------------------------------PAA---------GLG----AAPLQHVREQMATALR
Chicken KANK3        ---------------SEGQPGSVRMSPGSSGRST-------------------------------PAA-------------GLGTAPLQHVREQMAAALR
Goose KANK3          -----------------------DPSPARDGRAA------------------------------------------------------------------
Spotted gar Kank3    ---------------SPSQVIWPRASPQNSGRST-------------------------------PASGGTSSTGLTQIP----PNQLQTVREQMAAALR
Zebrafish Kank3      ---------------NPLSGAWTRASPQTSGWST-------------------------------PAS----------NSGTMPPGQLQTVREQMAAALR
Cavefish Kank3       VQ-------------NPPSTGWNRLSPQNSGRST-------------------------------PAS-----NCGTSASSSIPPGQLQTVREQMAAALR
Herring Kank3        SSASP----------SPCPSGLARASPHTSGRST-------------------------------PAA----------ASAQMPPAQLQTVREQMASALR
Stickleback Kank3    --------------QNPVQSGWTKPSPQTSGRST-------------------------------PAV--------GSTMTPIPPSQLQTVREQMATALK
Fugu Kank3           ---------------QSPQPLQMKPSPQTSGLST-------------------------------PAA----GTTMTPIP----PSQLQTVREQMATALR
Tetradon Kank3       ---------------QNAQPLQAKPSPQTSGLST-------------------------------PAA------AMTPIP----PSQLQTVREQMATALK
Medaka Kank3         ---------------LVVQSSWIKPSPQTSGRST-------------------------------PAA----GATVTPVP----PSQLQTVREQMAIALK
Tilapia Kank3        ---------------QPLQSSWTKPSPQTSGRST-------------------------------PAA----GTTVTPIP----PSQLQTVREQMATALK
Amazon molly Kank3   ---------------PPLQNSWTKPSPQTSGRST-------------------------------PAA--------GTTVTPIPPSQLQTVREQMATALK
Platyfish Kank3      ---------------PPLQNSWIKPSPQTSGRST-------------------------------PAA----GTTVTPIP----PSQLQTVREQMATALK

                              810       820       830       840       850       860       870       880       890       900
                     ....|....|....|....|....|....|....|....|....|....|....|....|....|....|....|....|....|....|....|....|
Hydra                NDQAT-QCSINTLEKSLAVGCSYANL----KTASSQCLVDMKD-----------------FQCGDNFVKYKDASSNVD----------------------
Trichoplax           LITDI-NHSVHY------------------QNGHANINPSFQA----------------------NQTEYKDSQHLNH----------------------
Aplasia              RMREL-EEQVKAIP---ILQVRISVL----KEEKRLLNLQLKA----HKANSKPMTRTVGLNTDRVDWSDRTSEFSSY-------------SQFERNFSQ
Snail                RIKEL-EDQVKTIP---ILQVRISVL----KEEKRLLNLQLKA------SKSNSHPPMISIGVGDNRIEVDEKVEKLVYSPKQFELPKSFTDSTQSLRSP
Fruitfly             RLKDL-EEQVKVIP---DMELELNSL----RSEKQKLLRRNEE-------------------LMRSQRQTPSPPSPGL----------------------
Hookworm             EVGVAVEPNVADVD----LQ----------TDEQAKAHFGVNT-------------------DEVEKMPDVEKADQST----------------------
C.elegance           PSRFA-NNRYDDLP---PTPTPTFRR----APSQGDLPRGFST---------APSTGPGDFASLRRGREFALPPEQEH-----------------HSRRF
Tunicate             RLRDL-EEQVKTIP---VLQVKISVL----QQEKKHLLDELKE-----------------AENKVERLKSPLDDLDHN----------------------
Spider               RMREL-EEQVKSIP---ILEIKLSAL----KEEKNSLLNQLKL-----------------KESKLQNKKSVNSDGDLQ-------------KQLKRLENE
Lingula              KMRDL-EEQVKSVP---LLQTRMSVL----KEEKRLLELQLLA-------KQSKPTSTYSIGVGDYRVDVEEPRAPRR-------------PDMRSIGVG
Lancelet             RIKVL-EEQVKEIP---VLQVKISVL----KEEKRLLVMQLKS----KNGE-----QNLKPEDTDNKREKQDGDKDGT-------------KRLHRRSLS
Medaka Kank4b        RVGEL-EMEVRVIP---ELRAQICIL----QEERERLRLGLVP---------------------HLISSTENGTTKRF----------------------
Amazon molly Kank4b  RVGEL-EMEVRVVP---ELRAHICIL----QEERERLRLGLVH---------------------HIPPSTVNGTTDLH----------------------
Platyfish Kank4b     RVGEL-EMEVRVVP---ELRAQICIL----QEERERLRLGLVH---------------------HIPPSSVNGTTDLH----------------------
Stickleback Kank4b   ------EKTAKTKP--------------------------------------------------------------------------------------
Fugu Kank4b          RVREL-EMEIRVIP---ELKAQICIL----QEERQRLQPGLLS-------------QSVNGTTSLYDRNHNISQGDST----------------------
Tetraon Kank4b       ----------------------------------------------------------------------------------------------------
```

```
Cave fish Kank4b      RTAEL-EEEIRVIP---ELKAQICIL----QEEREMLLYKFQT---------------QISPSTKEPVDFSSGSPQSS----------------------
Medaka Kank4a         RVREL-EEQVKTIP---ELKAQICSL----REEREQLLNQLKV-----------------QTQTLVSTSSSAAAKSPD------------------AASA
Fugu Kank4a           RVREL-EEQVKTIP---ELKAQICSL----REEREKLLLQLQA-----------------HVCTSPSTVVPSSNTETL----------------------
Tetradon Kank4a       RVREL-EEQVKTIP---ELKAQIFSL----KEEREQLLLQLQA--------------------HVSTSPSAVDPGSYT----------------------
Stickleback Kank4a    RVREL-EEQVKTIP---ELKAQICSL----REEREKLLLQLHA-------------------HAQAQSKAQVSSLPST----------------------
Tilapia Kank4a        RVREL-EEQVKTIP---ELKAQICSL----REEREQLLLQLQA------------------QAKAQASKLSTTVPTGY----------------------
Platyfish Kank4a      RVREL-EEQVKTIP---ELKAQICSL----REEREKLLCQIQTQFQVQASTSSSAMATTYETGGISQRQRPRPSEGLNLSLAIQTVQGVASEHVPVNVEE
Amazon Molly Kank4a   RVREL-EEQVKTIP---ELKAQICSL----REEREKLLRQIQTQSQVQASTSSSAMATTYETGGNSQRQRPRPSEGLNLSLAIQPVRGVASEHVPVNVEE
Herring Kank4         RVREL-EEQPPTPEEETKTRVEESESA---EEEPEEEAAETQK----------------PQTSEIQTEALERGPEEIT---------------------E
Zebradish Kank4       RVREL-EEQVRTIP---ELKAQICSL----RTEREGLLQKILQ------------------HQSEQKRPLQLKEIEVL---------QADLQVSALPPST
Cave fish Kank4a      RVREL-EEQVRTIP---ELKSQICSL----REEKEKLLQRIQE-----QAERKAEPSEKSQPEDSTEHPVATQQQEKA-------------SAFSEDTVV
Mouse KANK4           VDREL-GELEPAIP---EQVWEGAEP----EEGDLKASSHLSQ---------------PGPSSAVQSVPMDLEEVEIE----------------------
Rat KANK4             TDRAV-EELEPAIP---EPVWEGAEP----EEGDIKAASHLSQ---------------PAPSSSVQNVSVDLEEVDIQ----------------------
Dog KANK4             KVREF-GDLVPGIP---ELVQEGTELPEGEDEEEAPNHLSLPS-------------PPFFSQDALVVLEDEEDEPKTR----------------------
Human KANK4           RIPELVQEGAE--P----------------PEGVVKVPNHLPL---------------PGPPFSFQNVLVVLEDKEDE----------------------
Eagle KANK4           SLGQK-EHPGGGGPGPRQTQPPRQSQ----HPAAPQLQAVMES-------------------GGEEGDAADAGGRSAS--------------------AG
Chicken KANK4         EWPGK-GGLVRDVP-----KERAQPLWQG-QHSAVQLQVVMES-------------------RGEEGDASDATNGSTL----------------------
Goose KANK4           ------KERAPAQP---PRQGQHAAV--------QQLQVVVES-------------------------GAEEGDAADM----------------------
Anole lizard KANK4    KINKL-------VP----------------ETERENTQLGLNQ----------------KHIVKERMRPSASWEGQPF----------------------
Turtle KANK4          KNEQPEGQQVKAIP---ELDPQPLEF----PRERMLIQSPHQS---------------QHFIAQELLLVTESGGEECY----------------------
Aligator KANK4        KNKQL-GEQAKTTP---ELDHQPEEH----PQEGKEIQSPQPS---------------QPSTVQEPLMVIESGEEECE----------------------
Frog Kank2            RLRQL-EEQVKMIP---VLQVKISVL----QEEKRQLSVQLKS-------------------QKYLGHPRGNIRGKDR----------------------
Mouse KANK2           KLRQL-EEQVKLIP---VLQVKLSVL----QEEKRQLTVQLKS-------------------QKFLGHPSGTRSRSEL----------------------
Rat KANK2             KLRQL-EEQVKLIP---VLQVKLSVL----QEEKRQLTVQLKS-------------------QKFLGHPSGTRSRSEL----------------------
Dog KANK2             KLRQL-EEQVKLIP---VLQVKLSVL----QEEKRQLTVQLKS-------------------QKFLGHPTGARSRSEL----------------------
Human KANK2           KLRQL-EEQVKLIP---VLQVKLSVL----QEEKRQLTVQLKS-------------------QKFLGHPTAGRGRSEL----------------------
Aligator KANK2        QLRQL-EEQVKLIP---VLQVKVAVL----QEEKRQLSTRLQS-----------------------RRGLGQPVGGPG----------------------
Spotted gar Kank2     KIREL-EEQVKMIP---VLQVKISVL----QEEKRQLSVQLKS-------------------QKFLGHTMGFGKGRSR----------------------
Cave fish Kank2       KIRDL-EEQVKTIP---VLQVKISVL----QEEKRQLSVQLKS-----------------------QKFLGVGRSRPR----------------------
Herring Kank2         KIREL-EEQVKTIP---VLQVKISVL----QEEKRQLSVQLKS-------------------QKFLGHTLGFGGGSSG--------------------GR
Zebrafish Kank2       KIREL-EEQVKTIP---VLQVKISVL----QEEKRQLSVQLKS-------------------QKFLGHTLGFNRSRPR----------------------
Tetradon Kank2        KIREL-EEQVKTIP---VLQVKISVL----QEEKRQLSVQLKS-------------------QKFLGHTLGFSRGRSR----------------------
Fugu Kank2            KIREL-EEQVKTIP---VLQVKISVL----QEEKRQLSVQLKS-------------------QKFLGHTLGFSRGRSR----------------------
Medaka Kank2          KIKEL-EEQVKTIP---VLQVKISVL----QEEKRQLSVQLKS-------------------QKFLGHTLGFSRSRPR----------------------
Sticklebakc Kank2     KIRDL-EEQVKTIP---VLQVKISVL----QEEKRQLSVQLKS-------------------QKFLGHSLSFNRVRPR----------------------
Tilapia Kank2         KIREL-EEQVKTIP---VLQVKISVL----QEEKRQLSVQLKS-------------------QKFLGHTLGFNRSRPR----------------------
Amazon molly Kank2    KIKEL-EEQVKTIP---VLQVKISVL----QEEKRQLSVQLKS-------------------QKFLGHTLGFSRGRNR----------------------
Platyfish Kank2       KIKEL-EEQVKTIP---VLQVKISVL----QEEKRQLSVQLKS-------------------QKFLGHTLGFSRGRNR----------------------
Coelacanth KANK2      KLRQL-EEQVKMIP---VLQVKISVL----QEEKRQLSVQLKS-------------------QKFLGHTTGFGKPKPR----------------------
Anole lizard KANK2    KLRLL-EEQVKMIP---VLQVKISVL----QEEKRQLSVQLKS-------------------QKFLGHPGGFCKGKSR----------------------
Turtle KANK2          KLRQL-EEQVKIIP---VLQVKISVL----QEEKRQLSVQLKS-------------------QKFLGHPGGFGKGKGR----------------------
Tetradon Kank1b       KLKEL-EEQVKIIP---VLQVKISVL----QEEKRLLACQLKT-----------QSDSEEFNGGLRDAPQSPPSEKGD----------------------
Medaka Kank1b         KLKEM-EEQVKMIP---VLQVKISVL----QEEKRQLYSQLKN---------------QNDNENINNLIWNRSCDVEM----------------------
Amazon molly Kank1b   KLKEL-EEQVKIIP---ILQVKISVL----QEEKRQLVSQLSD----------------SKSINDMTWRTCLGEDGID----------------------
Platyfish Kank1b      KLKEL-EEQVKIIP---ILQVKISVL----QEEKRQLVSQLSD----------------SKSLNDMTWKTCLGEDGID----------------------
Frog Kank4            RIKDL-EEQLQTVT---EHKKTISEL----EGENSKLNAQLKS-------------------ISELLTSNRMENENVD----------------------
Zebrafish Kank1b      RLKDL-EEQVKAIP---LLQVKISVL----QEEKRQLAALVKS--------------------QDEDQKSGSSIDQEY----------------------
Tilapia Kank1b        RLKDL-EEQVKAIP---LLQVKISVL----QEEKRQLAALVKS--------------------QDEDQKSGSSIDQEY----------------------
Fugu Kank1b           RLKDL-EEQVKAIP---LLQVKISVL----QEEKRQLAALVKS--------------------QDEDQKSGSSIDQEY----------------------
Cavefish Kank1b       RLKEL-EEQVKTIP---MLQIKISTL----QEEKRQLVALVKQ----QEGQSLSSTLDSDLQGLFRKRAHSTGSTLQQ------------------LQQH
Spotted gar Kank4     RVKEL-EEQARMIP---ELEEKYQLI----LKLPPQSAVATEE------------------KTLHSEHERRNGQSQCL----------------------
Coelacanth KANK4      RIKEL-EEKVKSIP---NLKKQILLL----KEEKNVLITQLQN----QSFA-----------TEFKDAQSLTLHKGSR----------------------
Frog Kank1            RLKEL-EEQVKTIP---ILQVKISVL----QEEKRQLMSEIKS----------QKLSNQKDTAGFRKRSYSAGNAAGF---------------------E
Shark Kank1           RLKEL-EEQVKAIP---ILQVKISVL----QEEKRQLASQLKS----------HKLSSQNVAPGFRKRSYSAGNADQL----------------------
Coelacanth KANK1      RLKEL-EEQVKTIP---ILQVKISVL----QEEKRQLVSQLKS----------EKISNQNCSFNFRKRSYSTGNVDQF-----------------EQLAQ
Mouse KANK1           RLKEL-EEQVRTIP---VLQVKISVL----QEEKRQLASQLKS----------QRASSQNEACGVRKRSYSAGNASQL----------------ELLARA
Rat KANK1             RLKEL-EEQVRTIP---VLQVKISVL----QEEKRQLASQLKN----------QRAASQNEACGVRKRSYSAGNASQL----------------ELLSRA
```

```
Dog KANK1              RLKEL-EEQVRTIP---VLQVKISVL----QEEKRQLASQLKN----------QRATAQNDACGVRKRSYSAGNASQL----------------EQLSRA
Human KANK1            RLKEL-EEQVRTIP---VLQVKISVL----QEEKRQLVSQLKN----------QRAASQINVCGVRKRSYSAGNASQL----------------EQLSRA
Anole lizard KANK1     RLKEL-EEQVKTIP---VLQVKISVL----QEEKRQIMAKLKN----------QRIVNQNDTGHFRKRSYSAGNAEHW-----------------KNLSP
Eagle KANK1            RLKEL-EEQVKTIP---VLQVKISVL----QEEKRHMMAELKN----------QRKATQNDTYGFRKRSYSAGNAEQW-----------------EHMSQ
Chicken KANK1          RLKEL-EEQVKTIP---VLQVKISVL----QEEKRHMMAELKS----------QRKSSQNDVYGFRKRSYSAGNAEQW-----------------EHLSQ
Goose KANK1            RLKEL-EEQVKTIP---VLQVKISVL----QEEKRHMMAELKS----------QRKPSQNETYGFRKRSYSAGNAEQW-----------------EHLSQ
Turtle KANK1           RLKEL-EEQVKTIP---VLQVKISVL----QEEKRQMMAQLKS----------QRIPNQNDTYGFRKRSYSAGNAEQW-----------------GHLSQ
Aligator KANK1         RLKEL-EEQVKTIP---VLQVKISVL----QEEKRQMVAELKS----------QRKPSQNETYGFRKRSYSAGNAEQW-----------------EHLSQ
Spotted gar Kank1      RLKEL-EEQVKTIP---VLQVKISVL----QEEKRQLAAQINN----------QKSTSQSIGHGFRKRSYSAGNAQQF---------------------D
Herring Kank1          RLKEL-EEQVKTIP---ILQVKIAVL----QEDKRQIQKNQKL-----------AGQQGAGAGGFRKRSYSVGSADQF--------------------EP
Zebrafish Kank1a       RLKEL-EEQVKTIP---ILQVKISVL----QEEKRQLVSQKNN----------SKVVGQGSGGCFRKRSFSVGSADQY----------------------
Cave fish Kank1a       RLKEL-EEQVKTIP---ILQVKISVL----QEEKRQLVSQLKN----------PKLAGQGPLGGFRKRSYSVGSADQY----------------------
Stickleback Kank1a     KLKEL-EEQVKTIP---ILQVKIAVL----QEEKRQLAAQSKS----------------QAAGSFRKRSFSVGSAAQM---------------------E
Medaka Kank1a          RLKEL-EEQVKTIP---VLQVKIAVL----QEEKRQLAAQSKS----------------PGPAGFRKRSFSVGSAAQM----------------------
Fugu Kank1a            RLKEL-EEQVKTIP---ILQVKIAVL----QEEKRQMAAQSKN----------------QGPGGFRKRSYSVGSADQM----------------------
Tetradon Kank1a        RLKEL-EEQVKTIP---ILQVKIAVL----QEEKRQMAAQSKN----------------QGPGGFRKRSYSVGSADQM----------------------
Tilapia Kank1a         RLKEL-EEQVKTIP---ILQVKIAVL----QEEKRQLAAQSKG----------------QGLGGFRKRSYSVGCADQM----------------------
Amazon molly Kank1a    RLKEL-EEQVKSIP---ILQVKIAVL----QEEKRQLAAQNKN----------------QGLGGFRKRSYSVGSADQM----------------------
Mouse KANK3            RLREL-EDQARALP---ELQEQVRAL----RAEKARLLAGRVQ---------------PEQEVEIEARPDKLAQLRRL----------------------
Rat KANK3              RLREL-EDQARALP---ELQEQVRAL----RAEKARLLAGRVQ---------------PEQDVEIEVRPDKLSQLRRL----------------------
Dog KANK3              RLREL-EEQARALP---ELQEQVRTL----RAEKARLLAGRGL-----------PEPD----AEAEARPDKLAQLRRL------------TERLATYERG
Human KANK3            RLREL-EDQARTLP---ELQEQVRAL----RAEKARLLAGRAQ---------------PEPDGEAETRPDKLAQLRRL----------------------
Lamprey                LLTDNYAELADAFA---EPATELGSL-------SSRLLTTVTA-------------------LSSLQRPAPTPARAGS----------------------
Coelacanth KANK3       QLKDL-EEQVKTIP---ALERKISML----QEEREKLSSELRK----------RKALDTSEAFSFRQRSYSAGHTGVV----------------------
Frog Kank3             QLKDL-EEQVKAIP---ILEMKISTL----ESEKKQLLADLEK----------------KQEIVPLERSFSAVNCNLN----------------------
Turtle KANK3           QLKEL-EEQVKTIP---VLEMKICKL----KREKEKLMAGLQE----QPLP----ETSEASVFSFPPRSLGAEVARAS----------------------
Aligator KANK3         QLKEL-EEQVKTIP---VLEMKISKL----KQEKEKLLAGLQE----------KASPETCETSAFTFPPQSPTASEEQ----------------------
Eagle KANK3            QLRDL-EEQVKTIP---LLEMQICEL----KREKAKLMEKLSA-----------------EPGEAFGHPSGSEAVAGC----------------------
Chicken KANK3          QLREL-EEQVKTIP---LLEMRICEL----QREKAKLMEKLSA------------------EPSKVSLDHPDGSEEQP----------------------
Goose KANK3            ----------------------------------------------------------------------------------------------------
Spotted gar Kank3      QLKEM-EERVKGVP---ALEREVAKL----RAEKEELQAALRE------------KVGQSKIAELRKLTERLGGREDR----------------------
Zebrafish Kank3        QLREM-EERVKGVP---VLEREVAML----RAEKERLSVELQN------------------------------KTKEL----------------------
Cavefish Kank3         QLKEM-EEKVKGVP---ALEREVTLL----RAEKDRLELALQQ-------------------KTEELAALAEAAASSS----------------------
Herring Kank3          QLKEM-EERVKGVP---VLEREVAKL----RAEKDQLLQVLKK----------KNEAPATMAAAVQTALAEPGTGGDQ----------------------
Stickleback Kank3      QLKEM-EERVKGVP---ALENEVAKL----RAEKDALLLALQE-----------------KKEAREVTQLKQKSTESF----------------------
Fugu Kank3             QLKEM-EERVKGVP---ALEKELASV----RAEKDVLLLALQE-------------------RLTEKFEGRDGKASSK----------------------
Tetradon Kank3         QLKEM-EERVKGVP---ALEKELAGL----RAEKDALLLALEK--------------------AQRRQPTSDSFTQTT----------------------
Medaka Kank3           QLKEM-EERVKGVP---ALEKEVANL----RAEKDMLLLALEE---------------KKAAPGFVQERRPSAESSTQ----------------------
Tilapia Kank3          QLKEM-EERVKGVP---ALEKEVAKL----RAEKDMLLLALQE-------------KKAAMDLAQQKQQFTETSTQTV----------------------
Amazon molly Kank3     QLKEM-EERVKGVP---ALEKELAKL----RAEKDMLLLALEE-------------KKAAMEASHQKQQSKESSTQTT----------------------
Platyfish Kank3        QLKEM-EERVKGLP---ALEKELAKL----RAEKDMLLLALEE------------------KKAAMEVSHQKQQSKES----------------------

                                910       920       930       940       950       960       970       980       990      1000
                       ....|....|....|....|....|....|....|....|....|....|....|....|....|....|....|....|....|....|....|....|
Hydra                  --------------LFMKDAWTSSRII-------------------------------------------------------------------------
Trichoplax             ------------------------------------------NGSIPSPMA-------------------------------------------------
Aplasia                NQRTPPATLPKPVRTVSVGVGDHSVVEPYLLQPSLPTGYVLWENETVTELR--------------------------STSMMEQESYRNRIPSLNLSFQQ
Snail                  LAKTPPATLPKPVKTTSTGVGDHSVIEPYLLQPELPTGYTIEDNVTHTEIH------------------------TTTVIERDRLLKSFIQPPSLNVSFH
Fruitfly               -KTASPVQFTPQRISPVSLESLGARMRSSSTSASPSPSVVRRDVSTQAVVR-------------------------------------------------
Hookworm               ----ATEFLEKSASVDQETMTTRTYYRNIPSQTDSPEVEEAREVVVPDSCA-------------------------------------------------
C.elegance             FSASPKLPRRGLQAGPSIDVESHFTYAQTQNGQKVLARTQSTSTSPPPQAP-------------------------------------------------
Tunicate               -----KLSPRKSLLSTSSGFSSSSSQSSISSPRSPPPTSPGLPRSLVVETK-------------------------------------------------
Spider                 GIMSPTLTKKCLLSRNNMDNLSSPVIASCIDAAMAKKSFLEKEIISHIKMC-------------------------------------------------
Lingula                NKSVTELYTLQPHLLESQTLEKEMHTQRLIREKETKVVVVGQEQRPTSPYS-------------------------------------------------
Lancelet               TGSFQMLEQQWKEQQAEQEKQKASKDAPAPSEKNSTAKKPKVPPPVPPRSFRSVSVGDTTSLSDIVCYNSALRTTTDVGTNTTVPSRDIGVGYSPRMRHV
Medaka Kank4b          ------------------------------ALTHRSIVDMKRPIKVRNSTS-------------------------------------------------
Amazon molly Kank4b    --------TSSQYGKGDVLKSQRESHVMFPRNHNV-----------------------------------------------------------------
```

```
Platyfish Kank4b      --------TSSPYGKGDVLKSQRESHVMFPRNHSV-----------------------------------------------------------------
Stickleback Kank4b    ----------------------------------------------------------------------------------------------------
Fugu Kank4b           ----------------------------------------------------------------------------------------------------
Tetraon Kank4b        ----------------------------------------------------------------------------------------------------
Cave fish Kank4b      ----SHFTSSAVVSKTAEIQNKANDDWMNREYDQLEENVKASSEQVDAIVI-------------------------------------------------
Medaka Kank4a         IHFKGPSAEQDFSLLTQVSGGSEAAETALEEKVTVKETATAVRQTQPPKKL-------------------------------------------------
Fugu Kank4a           -PSGSTQATGVSECLPTKEITGKEKISVLVKESDINSERVEICSTHKETGQ-------------------------------------------------
Tetradon Kank4a       ----ESQEHKPPERLSPSVSTQPTGVSECLPTKELPSLVEV-----------------------------------------------------------
Stickleback Kank4a    --------IASKHDSGTHTQPQGQRPSAGLNTSAHLQTSRLSEQELQTETL-------------------------------------------------
Tilapia Kank4a        ---------DPRTNNQSQGQRPSQEMKLKETESPLGQTVQKLPRDIS-----------------------------------------------------
Platyfish Kank4a      SHRETKRTKELPIQKDPSTNQKQSVPSEKADKQKETSKSLQDNAEERLSQDTEVVAKESEIIRESNASKEDQNGMLKLEEKLMMLEAKLTQASQELERTN
Amazon Molly Kank4a   SHRETKRTKELPIQRDPSTNQKQSVPSETADKQRETSKSLQDNAEERLSQDTEVVAKESEIIRESNASKEDQNGMLKLEEKLMVLEAKLTQASQELERTN
Herring Kank4         ALVSVPETETETRDETSESITEQADAEPAHQEETQKISAQEFEKPVEASRQ-------------------------------------------------
Zebradish Kank4       SGKEDALFLSKNKVADHEIVHEVPSDRSTEQLPEKPHFEVQLNVQPDSKIN-------------------------------------------------
Cave fish Kank4a      DHKSKPPASAEPTVEPQTTEGQDLVAQDLAEKETSKSAVSVQPVPTVMSVP-------------------------------------------------
Mouse KANK4           -HHMREAELVLTPGSATPSPPPLPSPILENDLSLDEIELSISEIPPPPPI--------------------------------------------------
Rat KANK4             -HHTRTADLALSPGSSTPSPPPLPSPIPENELSLEEIELNISEIPPPPPI--------------------------------------------------
Dog KANK4             -----EAEVVVSPGSPTPSPPPLPSPIPENEFPLEEIELNISEIPPPPPV--------------------------------------------------
Human KANK4           -HNAREAEVLFTPGSPTPSPPPLPSPIPENELLLEEIELNISEIPPPPPV--------------------------------------------------
Eagle KANK4           VEPASPAALQLAEESTNPGEGELSVSEMALDVVK------------------------------------------------------------------
Chicken KANK4         -VSASPGALQLADESTNHGKAGGSVSEIALNVLKEGEDRCAH----------------------------------------------------------
Goose KANK4           --------VPASPGAPQLAEGSVSPGRVEKSISEIAPSVLKEDEYGGARTQ-------------------------------------------------
Anole lizard KANK4    ----ISQELLVVAESGEEECDTVEMNIRPPPVQSGSSCSMTLEYKFENQIP-------------------------------------------------
Turtle KANK4          ----APETNKCPELVKDESISSHAPKFMPENENREEIKLNISEIMPCAPE--------------------------------------------------
Aligator KANK4        -----TPAMETFTQLVKDVSSSPKLFDENENHEEIELNINEVMPSISE----------------------------------------------------
Frog Kank2            ----GELYIEIPEEESKVEKEEGEDKVAGKEGPEKENKKPEGEIKNPKEVC-------------------------------------------------
Mouse KANK2           -------CLDLPEAPDDPA---------------------------------------------------------------------------------
Rat KANK2             -------CLDLPEAPDDPA---------------------------------------------------------------------------------
Dog KANK2             -------CLDLPEPLEDPV---------------------------------------------------------------------------------
Human KANK2           -------CLDLPDPPEDPV---------------------------------------------------------------------------------
Aligator KANK2        -----ELYLDLPEEEMAARGT-------------------------------------------------------------------------------
Spotted gar Kank2     ----GELYIDIPEEDSTGPVAAAARGGPLSPTTPEGSRQDSGCEIEDTVIV-------------------------------------------------
Cave fish Kank2       -----ELYIDIPEEEVGGATTKAATTTGLSPTTPDGSRQDSGCEIEDTVIV-------------------------------------------------
Herring Kank2         GRPRGELYIDIPEEDTTTAAGTKATATGVTAGADRASRGLSPTPQADGSRQ-------------------------------------------------
Zebrafish Kank2       ----GELYIDIPEEEAGNGAGAANKATGSLS-PTTPGSLQDSGCEIEETVI-------------------------------------------------
Tetradon Kank2        ----GELYIDIPEEEPDTTG--------------------------------------------------------------------------------
Fugu Kank2            ----GELYIDIPEEEVGPEA--------------------------------------------------------------------------------
Medaka Kank2          ----GELYIDIPEEEASTGAP-------------------------------------------------------------------------------
Sticklebakc Kank2     ----GELYIDIPEEEPED----------------------------------------------------------------------------------
Tilapia Kank2         ----GELYIDIPEEDMS-----------------------------------------------------------------------------------
Amazon molly Kank2    ----GELYIDIPEEEVGTAAKSTKTTE---SLSPTEVSKQDSGCEIEDTVI-------------------------------------------------
Platyfish Kank2       ----GELYIDIPEEEGPEG---------------------------------------------------------------------------------
Coelacanth KANK2      ----GELYIDIPEEDSDQSGSVFSPPLPGSSRSHSGTEIEGSMMQRSLIKS-------------------------------------------------
Anole lizard KANK2    ----GELYIDIPEEGTSGNTEEGKVRPDTGARLD------------------------------------------------------------------
Turtle KANK2          ----GELYIEIPEEEGGGGGAG------------------------------------------------------------------------------
Tetradon Kank1b       -------RVEVPPRKPSKENTFVYTD--------------------------------------------------------------------------
Medaka Kank1b         ----------TDSENDKGADEELADPGNFKEVRQLSEEMDALEKAIKEGPL-------------------------------------------------
Amazon molly Kank1b   -------FENKRHTEAESKGTNENVYTDFREFRELTEEMQALERTIKGGHM-------------------------------------------------
Platyfish Kank1b      -------FENKRHTETESEGTNEKVYTDFREFRELTEEMQALERTIKGGHM-------------------------------------------------
Frog Kank4            ------LNISESEAQPEAITGN------------------------------------------------------------------------------
Zebrafish Kank1b      ------------INKPSVEGWEHKTMSTLKDFQLLSVEMQLLERKIQDARI-------------------------------------------------
Tilapia Kank1b        ------------INKPSVEGWEHKTMSTLKDFQLLSVEMQLLERKIQDARI-------------------------------------------------
Fugu Kank1b           ------------INKPSVEGWEHKTMSTLKDFQLLSVEMQLLERKIQDARI-------------------------------------------------
Cavefish Kank1b       DVKMQKTKESKRESSGEVEGWEHGALSGLQEFKQLSAEMHLLEKKIQDARL-------------------------------------------------
Spotted gar Kank4     -------------NNTPASQLSAQCMSASADKLRDTQDTQKTVKALA-----------------------------------------------------
Coelacanth KANK4      -----ELDNSVNEGVYATNGYKVAKNQNIPSLDVTIPETSMHTKQNTNVMS-------------------------------------------------
Frog Kank1            PPSRVRSAGELHIDSDDEMETPEQNSQTLKEFRQLTAEMQALEKKIHDSSL-------------------------------------------------
```

```
Shark Kank1          ----EPLRGELHIDSEDEMESLEQSSLRMEEFRQLTAEVQALERKIQDNGD-------------------------------------------------
Coelacanth KANK1     IRRSGEQVGELHIDSEDEIESMEQSSQRVKEFRQLTAEMQALERKIQESNE-------------------------------------------------
Mouse KANK1          RRGGGELYIDYEEEEMESVEQSTQRIQ---EFRQLTADMQALERKIQDSSC-------------------------------------------------
Rat KANK1            RRGGGELYIDYEEEEMESVEQSTQRIR---EFRQLTADMQALEQKIQDSSC-------------------------------------------------
Dog KANK1            RRSGGELYIDYEEEEMESVEQSAQSIR---EFRQLAADMQALERKIQDSGC-------------------------------------------------
Human KANK1          RRSGGELYIDYEEEEMETVEQSTQRIK---EFRQLTADMQALEQKIQDSSC-------------------------------------------------
Anole lizard KANK1   ARGGGELYIDCEDDAGSIEQSS----ERIEEFRQLTAEMQALEKKIQDSSY-------------------------------------------------
Eagle KANK1          VRRGGELYIDCEEEVESVEQSS----QGIEEFRQLTAEMQALEKKIQDSNY-------------------------------------------------
Chicken KANK1        VRRGGELYIDCEDEMESAEQSS----RRVEEFRQLTAEMQALEKKIQDSNY-------------------------------------------------
Goose KANK1          GRRGGELYIDCEDEMDSMEQSS----QRIEEFRQLTAEMQALEKKIQDSNY-------------------------------------------------
Turtle KANK1         VRRGGELYIDCEEEVEQNSQRTE-------EFRQLTFEMQALERKIQDSNY-------------------------------------------------
Aligator KANK1       VRRGGELYIDCEEDVEHVEQSS----QRIEEFRQLTAEMQALEKKIQDSNY-------------------------------------------------
Spotted gar Kank1    QLSQLKKGSELHINSEDELENMEHSSQRIEEFRQLTAEMQALERKIQDNNY-------------------------------------------------
Herring Kank1        LAQLKQGSSELHIDSEDELEGMEQSSLRLQEFRQLTAEMQALEKKIQEPGA-----------------------------------------------EK
Zebrafish Kank1a     --EPLSQIKTGSELHIEEIDNTEQSTQRLQEFRQLTAEVEALERNIQDNGE-------------------------------------------------
Cave fish Kank1a     --EPVAQLKSGSELHIDEPENVEQSSQRLQEFRQLTAEVQALEKKIQDSNE-------------------------------------------------
Stickleback Kank1a   APGAERQETELHIMEPEAQRQDAQRLDCAAEFRRLAAEVQALDGNAADARE-------------------------------------------------
Medaka Kank1a        ---ENPAAYKEAELHIMEPEAQEQSAQQLEELRRLTAEVQALEKTTKNNRW-------------------------------------------------
Fugu Kank1a          --ENPGPVQKEPELHIREPEAQEQSSQCLEEFRRLAAEVQALEKTTRENKI-------------------------------------------------
Tetradon Kank1a      --ENPGPFPKEPELHIREPEAQEHSSQCLEEFRRLAAQVQALEKTSQESNM-------------------------------------------------
Tilapia Kank1a       --ENPGPIQKETELHITEPEAQEQSAQRLEEFRQLAAEVQALEKTSEDNAN-------------------------------------------------
Amazon molly Kank1a  --ENSGSIQKETELRIIEPEAQEQSAQRLEEFRRLAAEVQALEKTTKENNI-------------------------------------------------
Mouse KANK3          -----TERLATSDRGVRSRASPRAEDPDGLAARRSEGALQVLDPGSRTPDG-------------------------------------------------
Rat KANK3            -----TERLATSDRGVRSRASPRAEDPDGLAARRSEGALQVLDPASRTPDG-------------------------------------------------
Dog KANK3            VRARAGPWADGPDGPASRRGEGALEVPDAAGIPDAAPQTRDAGTQAVPETQ-------------------------------------------------
Human KANK3          -----TERLATSERGGRARASPRADSPDGLAAGRSEGALQVLDGEVGSLDG-------------------------------------------------
Lamprey              ----------------------------------------------------------------------------------------------------
Coelacanth KANK3     -DAKAKARQDHHGEFDSESEAVKIRTSKIVELRRLTEKLTNLERNPKCEKT-------------------------------------------------
Frog Kank3           -ESCSEQNIELPSETENELETSKLRTNKIAELKRLTEKLAVPERNVKMSRT-------------------------------------------------
Turtle KANK3         --------QEAPAELERELESVKARASKITELRRLTEKLSASDRNVRASKA-------------------------------------------------
Aligator KANK3       ----PPVSQEAQGELEGESEMGKARASKITELRKLTEKLSTSERNVRAGRT-------------------------------------------------
Eagle KANK3          -------EEPPRAGPESETEPAKGRASKVAELRKLTEKLAVPERSGRAWPG-------------------------------------------------
Chicken KANK3        -----------QAELEGEAEPGKGRASKIAELRKLTEKLAVPERGSRSVPG-------------------------------------------------
Goose KANK3          ----------------------------------------------------------------------------------------------------
Spotted gar Kank3    --NSEQVERTMVVERKSVGVGGDRPLEEGVFYYRKGTKEVAVGTEAVEACE-------------------------------------------------
Zebrafish Kank3      -----EAAIKLQVPTEAVEEQDKEQIKKIPTFVLETKSIAVGNDVPLENVI-------------------------------------------------
Cavefish Kank3       ---EPELKSSEVVKEPPPMRVERNSIAVGDDVPLEAVVVCNRQNVKDVSIE-------------------------------------------------
Herring Kank3        -GLPPLSPTHGRSTRVSELRKAIEKASKEEGSQQQQQQQQQTEKAVPAPVQ-------------------------------------------------
Stickleback Kank3    -TQTAEVLQTDPTTRGQPTTPVSPGLKGLGKSGELKRLTERQGVKDASEGT-------------------------------------------------
Fugu Kank3           ------------VLVKAPEKVREKKSVSVGEDVPMESIVFYYSQGLKDACE-------------------------------------------------
Tetradon Kank3       ----ETTKMHQGTQSRLTSPPSAVRGPGKAELGRLTEKFEGSRGRKDACEG-------------------------------------------------
Medaka Kank3         ---TTETQQMDQSQLTSPTTTGHKRFSKPGELKWLTEKFEVKEQKSPQIIS-------------------------------------------------
Tilapia Kank3        -----EILQTEHSRLTSPTSTGHKGFVKSGELKRLTEKDGVKDACEGTRVN-------------------------------------------------
Amazon molly Kank3   -----EILQSDYSRVTSPNSTGHKGLVKSGELKRLAEKFEGKELKSPPPPL-------------------------------------------------
Platyfish Kank3      ----STQTTELLQFLPHLAEKEKKSVAVGDDVPMTSVVFYHRNGVKDASVC-------------------------------------------------

                              1010      1020      1030      1040      1050      1060      1070      1080      1090      1100
                     ....|....|....|....|....|....|....|....|....|....|....|....|....|....|....|....|....|....|....|....|
Hydra                ----------------------------------------------------------------------------------------------------
Trichoplax           ----------------------------------------------------------------------------------------------------
Aplasia              PSSLEERESRFSSSMVSESSQQPHFTIKQIQRNAPKALTRSVGIGDGNVFEGSGLHVHEKEFRTVFIGQNDKVAKRNVGIECRVPTRDVGVSFMCDEE--
Snail                RTTSQESIDRETRLPSSSQLESPRFTINQIQRSTPKALTRTLGVGEGNVFEDSGLHVHEKELRTVIIGQSGAVAKRNVGIDCRVPTRDVGVSFMCDEE--
Fruitfly             ----------------------------------------------------------------------------------------------------
Hookworm             ----------------------------------------------------------------------------------------------------
C.elegance           --------------------------------ASAFEPVKPLSNGSPSPTHQVQK---------------------------------------------
Tunicate             -------------------------LDLRGPPLPPKRKYRSIAVGDSMPYQDIVCYSKSNLITHEVESDCQILRQTS-----------------------
Spider               ---------------------------DINSGVEKCKNMKSVGTSCSVLTRDIGIGYV------------------------------------------
```

```
Lingula                ---------------------------PMIKTMERKPITRSVGVGDGNVFEKDQMQIQEKELRTIIIDKEAKKQTRNVGISVRVPMRDVGVHYHCEAE--
Lancelet               GVGISNDLLQQELGIVENQKCNTAVVETSEVGVGVSVEQRAVAVGNHTELRDVGMTTDPEVVKKQKTKHATRSIGVIVDIPETKDVRTVGVGNGSVSEHQ
Medaka Kank4b          ----------------------------------------------------------------------------------------------------
Amazon molly Kank4b    ----------------------------------------------------------------------------------------------------
Platyfish Kank4b       ----------------------------------------------------------------------------------------------------
Stickleback Kank4b     ----------------------------------------------------------------------------------------------------
Fugu Kank4b            ----------------------------------------------------------------------------------------------------
Tetraon Kank4b         ----------------------------------------------------------------------------------------------------
Cave fish Kank4b       ----------------------------------------PFTTGTGRNLQGTKT---------------------------------------------
Medaka Kank4a          ----------------------------------------------------------------------------------------------------
Fugu Kank4a            ------------------ESSLPVGARKDASRI-------------------------------------------------------------------
Tetradon Kank4a        ----------------------------------------------------------------------------------------------------
Stickleback Kank4a     ----------------------------------------------------------------------------------------------------
Tilapia Kank4a         ----------------------------------------------------------------------------------------------------
Platyfish Kank4a       ALLKEQMEENKAKEDRILQLSEGMRVEICTTQVPVRSRRESIDAGTVTEKIDLSHQETETESPSKIDQGTDTDKILVEVIVP------------------
Amazon Molly Kank4a    ALLKEQMEENKVKEDRILQLSEGMRVEICTTQVPERPRRESIDAGTVTERIDLSHQETETESPAKIDQGTDTDKILVEVIVP------------------
Herring Kank4          -----------------GVEQQDKKAESPTEHFEEKKIEPSEGVSKEAAMPRVPLECTVSVVITEA----------------------------------
Zebradish Kank4        -----------KPVTDTESEKEQVPAPVSVPVILIDKAETPTDPDEEEELLQEPTLQEEASKSPSEQVKEQERSEILLEDKQPTLLEGIPASESE-----
Cave fish Kank4a       -----------------VILIEKVESPTDSEESEKQLEEFKHEAEIPESLSEQELEKQEGTQDT------------------------------------
Mouse KANK4            ----------------------------------------------------------------------------------------------------
Rat KANK4              ----------------------------------------------------------------------------------------------------
Dog KANK4              ----------------------------------------------------------------------------------------------------
Human KANK4            ----------------------------------------------------------------------------------------------------
Eagle KANK4            ----------------------------------------------------------------------------------------------------
Chicken KANK4          ----------------------------------------------------------------------------------------------------
Goose KANK4            ----------------------------------------------------------------------------------------------------
Anole lizard KANK4     -------------------------------------EDIELSICETEVLE-------------------------------------------------
Turtle KANK4           ----------------------------------------------------------------------------------------------------
Aligator KANK4         ----------------------------------------------------------------------------------------------------
Frog Kank2             ---------------------------------------------KKETVENNSLP--------------------------------------------
Mouse KANK2            ----------------------------------------------------------------------------------------------------
Rat KANK2              ----------------------------------------------------------------------------------------------------
Dog KANK2              ----------------------------------------------------------------------------------------------------
Human KANK2            ----------------------------------------------------------------------------------------------------
Aligator KANK2         ------------------------------------RELRSVAVGTEGG---------------------------------------------------
Spotted gar Kank2      --------------------------GGARQPAGGRKEVRSIGTEPEAGA--------------------------------------------------
Cave fish Kank2        ----------------------------GGGRPGNRKEVRTIGVGSDVAE--------------------------------------------------
Herring Kank2          ----------------DSGCEIEDTVIVGGARPGARKEVRTIGVGPEEKEGER-----------------------------------------------
Zebrafish Kank2        ---------------------------VAGARPGAKREVRTVGVGPEAE---------------------------------------------------
Tetradon Kank2         ----------------------------------------------------------------------------------------------------
Fugu Kank2             ----------------------------------------------------------------------------------------------------
Medaka Kank2           ----------------------------------------------------------------------------------------------------
Sticklebakc Kank2      ----------------------------------------------------------------------------------------------------
Tilapia Kank2          ----------------------------------------------------------------------------------------------------
Amazon molly Kank2     ---------------------------VGGARPDAKREVRTIGVGPEG----------------------------------------------------
Platyfish Kank2        ----------------------------------------------------------------------------------------------------
Coelacanth KANK2       ----------------------------------PKKEMRSISVGTGEEMMVRESRPS----------------------------ASVGLGQ-------
Anole lizard KANK2     -----------------------------------RKEVRSVGVGRSAEDREGNE---------------------------------------------
Turtle KANK2           ----------------------------------------------------------------------------------------------------
Tetradon Kank1b        ----------------------------------------------------------------------------------------------------
Medaka Kank1b          -----------------------QVWHGKGPISVQGKATKSVAVGTDTDMVPPKPSKVDKSVHTE-----------------------------------
Amazon molly Kank1b    -----------------------LSWQDKGHGLLHENTTKTIGIKTDIDMKMTLSKNSKENKSVHTD---------------------------------
Platyfish Kank1b       -----------------------LSWQEKGHSLLHKNTTNSIGIKTDIDMKMASSKTSKENKSVHTD---------------------------------
Frog Kank4             ----------------------------------------------------------------------------------------------------
Zebrafish Kank1b       -----------------------------ESSAFKMRDRESVDRGIDEAFCKDGTIGA------------------------------------------
Tilapia Kank1b         -----------------------------ESSAFKMRDRESVDRGIDEAFCKDGTIGA------------------------------------------
Fugu Kank1b            -----------------------------ESSAFKMRDRESVDRGIDEAFCKDGTIGA------------------------------------------
```

```
Cavefish Kank1b         --------------------------AAHPVAKARALIRRSVTVGENKNMCEDCRCTKSSFRDV------------------------------------
Spotted gar Kank4       ----------------------------------------------------------------------------------------------------
Coelacanth KANK4        --------------------------------------SKHLELNISENEMESSV---------------------------------------------
Frog Kank1              --------------------ESRCPDTQPREIKTKEKCSRSVAVGADENMNDIIIYHKSVKSS-----------------------QDASVGT-------
Shark Kank1             --------------------EKPGGVEPCVKHLAKAREFRSVAVSTDVNMNDFVIYHRSARSF-----------------------KDVAVGT-------
Coelacanth KANK1        -------------------VPVTSGFTQRTENPQKAKEYRSIAVGADENMNDIVIYHKSSRTC-----------------------KAIAVGT-------
Mouse KANK1             ----------------------EVASELRENGQCPSRECKSVAVGSDENMNDVVVYHRDLRPC-----------------------KDTAVGT-------
Rat KANK1               ----------------------EATPELRENGQCASRERKSVAVGSDENMNDIVVYHRDFRSR-----------------------KDTAVGT-------
Dog KANK1               ---------------------------------QPAPEGRSVAVGADDTMDSVVVYRRAAGSR-----------------------RDAAVGT-------
Human KANK1             ---------------------------EASSELRENGECRSVAVGAEENMNDIVVYHRGSRSC-----------------------KDAAVGT-------
Anole lizard KANK1      ----------------------DNPASMRVNRASVMKENRSVAVGTDESMDNIVVYNRALRAF-----------------------RDIAVGT-------
Eagle KANK1             ----------------------ESPANLRVNRESLTKEARSVAVGADENMNDVVIYNRSARQH-----------------------KEVAVGT-------
Chicken KANK1           -------------------------EVPSNLRESLTKESRSVAVGADENMNDVVIYNRSSRQH-----------------------REVAVGT-------
Goose KANK1             ----------------------ETPSNLRANRESLTKESRSVAVGADENINDVVIYNRSSRQR-----------------------REVAVGT-------
Turtle KANK1            ----------------------EIPSNLRTNRENITKESKSVAVGADENMNDVVIYNRSSRPC-----------------------MEVAVGT-------
Aligator KANK1          ----------------------EIPSNLRMNRDSLARESRSVAVGADENMNDVVIYNRSSRQC-----------------------REVAVGT-------
Spotted gar Kank1       --------------------DTQFNLTQKAEQKQNAKEQKSIAVGADENMNDVVIYRKSSKQY-----------------------RDVAVET-------
Herring Kank1           KIQEPGAEKNRRQQHSQMQNAAAAPAPAATLQRQSSREQKSIAVGTDENMNDVVICRRSPERS-----------------------KDASVGTE------
Zebrafish Kank1a        ---------------EIKHSLHQTELRSNQNVQLWSRESRSIGVGADENMNDVVVYRRSPGKN-----------------------KDVAVGT-------
Cave fish Kank1a        -------------EPERGLNQMQHNTNQNSLQRQWNRESKSIGVGADENMNDVVIYRRLPGRN-----------------------KDVCIGT-------
Stickleback Kank1a      -------------------------------------SQKSVGMVTDENMASLRADQRARGPRS----------------------RDAAVSTE------
Medaka Kank1a           -----------------------------TDGTPPRSQQGAVGVATDKSAKDLPDTPQSTFRDVGVSTE-------------------------------
Fugu Kank1a             ------------------------NEVHSHKVLQNQAHQKSIGIVTDENMNNLPVPHGKLTNG--------------------HCLRDAGVSTE------
Tetradon Kank1a         ------------------------DEVHSQKLLQNQTHQKSVGIVTDENMNSLPVLHGKLTSG--------------------LCFQDAGVSTE------
Tilapia Kank1a          -------------------------ENQPQNLTQNQAHQKSIGIVTDQNMNNLPATQRKPEKE--------------------RCFQDAGVSTE------
Amazon molly Kank1a     -------------------------AEVQPQNLQKQVLQKSIGIVTDENMDNLLGSLSKPQKD--------------------SCLQDVGVVTE------
Mouse KANK3             ------------------------EPRTRETGTEVVPETREVDAQAVPETGEAGVEV-------------------------------------------
Rat KANK3               ------------------------EPQTREAGTEVVPETREVDAQAVPETREAGVEV-------------------------------------------
Dog KANK3               ----------------------------------------DARAQAEPETQEAGVEA-------------------------------------------
Human KANK3             ------------------------TPQTREVAAEAVPETREAGAQAVPETREAGVEA-------------------------------------------
Lamprey                 ----------------------------------------------------------------------------------------------------
Coelacanth KANK3        --------------------DIAYIAENQIKASERKTTYMSVAVGEDINMNEAVFFYRSQRPC-----------------------KEVAVGS-------
Frog Kank3              -----------------------GASKITSPPKVMERRSKSIAVGEEMDMNDVVFYYRSQNTN-----------------------REVAVGC-------
Turtle KANK3            ---------------------------AARPAKAMEKACKSVGVGEGVNMNEAVFYYRSQKPC-----------------------HEIAVGQ-------
Aligator KANK3          ---------------------------GTRPARPSDQACKSVGVGEEMDMNDVVFYYRSQKLC-----------------------REVAVGQ-------
Eagle KANK3             -----------------------------RSPRAAERLCRSVAVGEDRAMTDAVFYYRSQREGGDMPDGR------------------------------
Chicken KANK3           -------------------------KGMAARPRAAERQCRSVAVGEERAMSDAVFYYRSQQEGGEAPE--------------------------------
Goose KANK3             ----------------------------------------------------------------------------------------------------
Spotted gar Kank3       -----------------------------------------KGVGTDARETRDAGAEA------------------------------------------
Zebrafish Kank3         --------------------------------VSNRQTLKDAAVGSIIDVCHAAVETEANAMCDEGIQT-------------------------------
Cavefish Kank3          ---------------------------------------ATVSVSHVGIETEVATMHDEEVQVG------------------------------------
Herring Kank3           --------------------------------------MASLAVGEDLPMSAGFFYYRQDMKDAAVDVRCEVTDKGVGTAPAGSACDKGVQV--------
Stickleback Kank3       ----------------------------------------QVKVCDKGSGMDAPLVQEEGVQV-------------------------------------
Fugu Kank3              --------------------------------------GTAVKVCEKGTRTEVPLLRDEGAQT-------------------------------------
Tetradon Kank3          ---------------------------------------PAVKLWEKGTGTEVPVVRDQGAQT-------------------------------------
Medaka Kank3            ---------------------------KLNPAEKAVVQKKTVAVGDDVPMSSIVFYYSHNVIDATVGTEVTVSEKCTGTDAPLVQEQEIQA---------
Tilapia Kank3           -------------------------------------------VCEKCTETEPPLVQEEGIQT-------------------------------------
Amazon molly Kank3      ---------------------------SKVLPHSAEKEKKSVAVGDDVPMTSVVFYHRNGVRDASVCTEVKVCDKTTGTEAAVVREQAIQV---------
Platyfish Kank3         ---------------------------------------MEVKVCDKTTETEAAVVHEQAIQV-------------------------------------

                                 1110      1120      1130      1140      1150      1160      1170      1180      1190      1200
                        ....|....|....|....|....|....|....|....|....|....|....|....|....|....|....|....|....|....|....|....|
Hydra                   ----------------------------------------------------------------------------------------------------
Trichoplax              ----------------------------------------------------------------------------------------------------
Aplasia                 ----------------------------------------------------------------------------------------------------
Snail                   ----------------------------------------------------------------------------------------------------
Fruitfly                ----------------------------------------------------------------------------------------------------
```

```
Hookworm               ----------------------------------------------------------------------------------------------------
C.elegance             ----------------------------------------------------------------------------------------------------
Tunicate               ----------------------------------------------------------------------------------------------------
Spider                 ----------------------------------------------------------------------------------------------------
Lingula                ----------------------------------------------------------------------------------------------------
Lancelet               KPPVETVSVYTNTPVINTIDKVTNTVAVNLKTVAVGNFSVKDVLCDRCADVKTKTVAVGVCSIKDTICDRCNNMESKTVGVGRCTVNDTVCDKCSNLKTR
Medaka Kank4b          ----------------------------------------------------------------------------------------------------
Amazon molly Kank4b    ----------------------------------------------------------------------------------------------------
Platyfish Kank4b       ----------------------------------------------------------------------------------------------------
Stickleback Kank4b     ----------------------------------------------------------------------------------------------------
Fugu Kank4b            ----------------------------------------------------------------------------------------------------
Tetraon Kank4b         ----------------------------------------------------------------------------------------------------
Cave fish Kank4b       ----------------------------------------------------------------------------------------------------
Medaka Kank4a          ----------------------------------------------------------------------------------------------------
Fugu Kank4a            ----------------------------------------------------------------------------------------------------
Tetradon Kank4a        ----------------------------------------------------------------------------------------------------
Stickleback Kank4a     ----------------------------------------------------------------------------------------------------
Tilapia Kank4a         ----------------------------------------------------------------------------------------------------
Platyfish Kank4a       ----------------------------------------------------------------------------------------------------
Amazon Molly Kank4a    ----------------------------------------------------------------------------------------------------
Herring Kank4          ----------------------------------------------------------------------------------------------------
Zebradish Kank4        ----------------------------------------------------------------------------------------------------
Cave fish Kank4a       ----------------------------------------------------------------------------------------------------
Mouse KANK4            ----------------------------------------------------------------------------------------------------
Rat KANK4              ----------------------------------------------------------------------------------------------------
Dog KANK4              ----------------------------------------------------------------------------------------------------
Human KANK4            ----------------------------------------------------------------------------------------------------
Eagle KANK4            ----------------------------------------------------------------------------------------------------
Chicken KANK4          ----------------------------------------------------------------------------------------------------
Goose KANK4            ----------------------------------------------------------------------------------------------------
Anole lizard KANK4     ----------------------------------------------------------------------------------------------------
Turtle KANK4           ----------------------------------------------------------------------------------------------------
Aligator KANK4         ----------------------------------------------------------------------------------------------------
Frog Kank2             ----------------------------------------------------------------------------------------------------
Mouse KANK2            ----------------------------------------------------------------------------------------------------
Rat KANK2              ----------------------------------------------------------------------------------------------------
Dog KANK2              ----------------------------------------------------------------------------------------------------
Human KANK2            ----------------------------------------------------------------------------------------------------
Aligator KANK2         ----------------------------------------------------------------------------------------------------
Spotted gar Kank2      ----------------------------------------------------------------------------------------------------
Cave fish Kank2        ----------------------------------------------------------------------------------------------------
Herring Kank2          ----------------------------------------------------------------------------------------------------
Zebrafish Kank2        ----------------------------------------------------------------------------------------------------
Tetradon Kank2         ----------------------------------------------------------------------------------------------------
Fugu Kank2             ----------------------------------------------------------------------------------------------------
Medaka Kank2           ----------------------------------------------------------------------------------------------------
Sticklebakc Kank2      ----------------------------------------------------------------------------------------------------
Tilapia Kank2          ----------------------------------------------------------------------------------------------------
Amazon molly Kank2     ----------------------------------------------------------------------------------------------------
Platyfish Kank2        ----------------------------------------------------------------------------------------------------
Coelacanth KANK2       ----------------------------------------------------------------------------------------------------
Anole lizard KANK2     ----------------------------------------------------------------------------------------------------
Turtle KANK2           ----------------------------------------------------------------------------------------------------
Tetradon Kank1b        ----------------------------------------------------------------------------------------------------
Medaka Kank1b          ----------------------------------------------------------------------------------------------------
Amazon molly Kank1b    ----------------------------------------------------------------------------------------------------
Platyfish Kank1b       ----------------------------------------------------------------------------------------------------
```

```
Frog Kank4               ----------------------------------------------------------------------------------------------------
Zebrafish Kank1b         ----------------------------------------------------------------------------------------------------
Tilapia Kank1b           ----------------------------------------------------------------------------------------------------
Fugu Kank1b              ----------------------------------------------------------------------------------------------------
Cavefish Kank1b          ----------------------------------------------------------------------------------------------------
Spotted gar Kank4        ----------------------------------------------------------------------------------------------------
Coelacanth KANK4         ----------------------------------------------------------------------------------------------------
Frog Kank1               ----------------------------------------------------------------------------------------------------
Shark Kank1              ----------------------------------------------------------------------------------------------------
Coelacanth KANK1         ----------------------------------------------------------------------------------------------------
Mouse KANK1              ----------------------------------------------------------------------------------------------------
Rat KANK1                ----------------------------------------------------------------------------------------------------
Dog KANK1                ----------------------------------------------------------------------------------------------------
Human KANK1              ----------------------------------------------------------------------------------------------------
Anole lizard KANK1       ----------------------------------------------------------------------------------------------------
Eagle KANK1              ----------------------------------------------------------------------------------------------------
Chicken KANK1            ----------------------------------------------------------------------------------------------------
Goose KANK1              ----------------------------------------------------------------------------------------------------
Turtle KANK1             ----------------------------------------------------------------------------------------------------
Aligator KANK1           ----------------------------------------------------------------------------------------------------
Spotted gar Kank1        ----------------------------------------------------------------------------------------------------
Herring Kank1            ----------------------------------------------------------------------------------------------------
Zebrafish Kank1a         ----------------------------------------------------------------------------------------------------
Cave fish Kank1a         ----------------------------------------------------------------------------------------------------
Stickleback Kank1a       ----------------------------------------------------------------------------------------------------
Medaka Kank1a            ----------------------------------------------------------------------------------------------------
Fugu Kank1a              ----------------------------------------------------------------------------------------------------
Tetradon Kank1a          ----------------------------------------------------------------------------------------------------
Tilapia Kank1a           ----------------------------------------------------------------------------------------------------
Amazon molly Kank1a      ----------------------------------------------------------------------------------------------------
Mouse KANK3              ----------------------------------------------------------------------------------------------------
Rat KANK3                ----------------------------------------------------------------------------------------------------
Dog KANK3                ----------------------------------------------------------------------------------------------------
Human KANK3              ----------------------------------------------------------------------------------------------------
Lamprey                  ----------------------------------------------------------------------------------------------------
Coelacanth KANK3         ----------------------------------------------------------------------------------------------------
Frog Kank3               ----------------------------------------------------------------------------------------------------
Turtle KANK3             ----------------------------------------------------------------------------------------------------
Aligator KANK3           ----------------------------------------------------------------------------------------------------
Eagle KANK3              ----------------------------------------------------------------------------------------------------
Chicken KANK3            ----------------------------------------------------------------------------------------------------
Goose KANK3              ----------------------------------------------------------------------------------------------------
Spotted gar Kank3        ----------------------------------------------------------------------------------------------------
Zebrafish Kank3          ----------------------------------------------------------------------------------------------------
Cavefish Kank3           ----------------------------------------------------------------------------------------------------
Herring Kank3            ----------------------------------------------------------------------------------------------------
Stickleback Kank3        ----------------------------------------------------------------------------------------------------
Fugu Kank3               ----------------------------------------------------------------------------------------------------
Tetradon Kank3           ----------------------------------------------------------------------------------------------------
Medaka Kank3             ----------------------------------------------------------------------------------------------------
Tilapia Kank3            ----------------------------------------------------------------------------------------------------
Amazon molly Kank3       ----------------------------------------------------------------------------------------------------
Platyfish Kank3          ----------------------------------------------------------------------------------------------------

                                  1210      1220      1230      1240      1250      1260      1270      1280      1290      1300
                         ....|....|....|....|....|....|....|....|....|....|....|....|....|....|....|....|....|....|....|....|
Hydra                    -------------------------------------------ETKSTYSGCE-----------------------------------------------
```

```
Trichoplax             -------------------------------------------TNGRRDSNGN-----------------------------------------------
Aplasia                -------------------------------------------KPETRTIGVN-----------------------------------------------
Snail                  -------------------------------------------KPATRTIGVN-----------------------------------------------
Fruitfly               -------------------------------------------PPATRDVSVGHQ---------------------------------------------
Hookworm               -------------------------------------------TAASSDVEC------------------------------------------------
C.elegance             -------------------------------------------PIVTRSIAVS-----------------------------------------------
Tunicate               -------------------------------------------PPETVDFSGLVEPC-------------------------------------------
Spider                 -------------------------------------------PNKSKSIGVGN----------------------------------------------
Lingula                -------------------------------------------KPSQRDVGVSVG---------------------------------------------
Lancelet               SIGCTTLANWNQKSVSVLAKVEQTDAGTGDDSVLEETVDSGKDQTETKDIGVGEDSIFPLEEAKPPVLIQEEKTVNVVSFATAARPPLEIVESSSSDEVE
Medaka Kank4b          -------------------------------------------DDNTQPMHEW------------------------------------------------
Amazon molly Kank4b    -------------------------------------------TSKINPANDW-----------------------------------------------
Platyfish Kank4b       -------------------------------------------TSKINPVNDW-----------------------------------------------
Stickleback Kank4b     ----------------------------------------------------------------------------------------------------
Fugu Kank4b            ------------------------------------------------ATHEW-----------------------------------------------
Tetraon Kank4b         -------------------------------------------EEAGRQIGSG-----------------------------------------------
Cave fish Kank4b       -------------------------------------------SRDNKDLSDH-----------------------------------------------
Medaka Kank4a          -------------------------------------------DKQKETFEHA-----------------------------------------------
Fugu Kank4a            ----------------------------------------------------------------------------------------------------
Tetradon Kank4a        -------------------------------------------RQDASRIHHA-----------------------------------------------
Stickleback Kank4a     -------------------------------------------ESTQDDAVQK-----------------------------------------------
Tilapia Kank4a         -------------------------------------------GEELLSITVA-----------------------------------------------
Platyfish Kank4a       -------------------------------------------KPRARSIDRG-----------------------------------------------
Amazon Molly Kank4a    -------------------------------------------KPRARIIDRG-----------------------------------------------
Herring Kank4          -------------------------------------------EEESEEADLY-----------------------------------------------
Zebradish Kank4        -------------------------------------------CTSTQEVTMT-----------------------------------------------
Cave fish Kank4a       -------------------------------------------SMEDKQLELS-----------------------------------------------
Mouse KANK4            -------------------------------------------EVDVRSIGIR-----------------------------------------------
Rat KANK4              -------------------------------------------EVDVRSVGVR-----------------------------------------------
Dog KANK4              -------------------------------------------EIDVRSIGIR-----------------------------------------------
Human KANK4            -------------------------------------------EVDMRSIGIR-----------------------------------------------
Eagle KANK4            -------------------------------------------KAEEGSVRAG-----------------------------------------------
Chicken KANK4          -------------------------------------------AQEKKEQGGD-----------------------------------------------
Goose KANK4            -------------------------------------------EDEERGGEAGE----------------------------------------------
Anole lizard KANK4     -------------------------------------------EGKVVSSGIR-----------------------------------------------
Turtle KANK4           -------------------------------------------KTEVRSVWAR-----------------------------------------------
Aligator KANK4         -------------------------------------------ENEDRNVWAR-----------------------------------------------
Frog Kank2             -------------------------------------------GKRMCSVGVW-----------------------------------------------
Mouse KANK2            -------------------------------------------ALETRSVGTW-----------------------------------------------
Rat KANK2              -------------------------------------------VLETRSVGTW-----------------------------------------------
Dog KANK2              -------------------------------------------TLETRSVGTW-----------------------------------------------
Human KANK2            -------------------------------------------ALETRSVGTW-----------------------------------------------
Aligator KANK2         -------------------------------------------TSGQRSVGVG-----------------------------------------------
Spotted gar Kank2      -------------------------------------------KREVRHIGVE-----------------------------------------------
Cave fish Kank2        -------------------------------------------NKRTRQVGVG-----------------------------------------------
Herring Kank2          -------------------------------------------ERCYRQVGVG-----------------------------------------------
Zebrafish Kank2        -------------------------------------------ERGYRQVGVG-----------------------------------------------
Tetradon Kank2         -------------------------------------------PSRTRGVGVG-----------------------------------------------
Fugu Kank2             -------------------------------------------VRGSRQVGVG-----------------------------------------------
Medaka Kank2           -------------------------------------------TQNNCDVGVW-----------------------------------------------
Sticklebakc Kank2      -------------------------------------------PRGSRHVGVG-----------------------------------------------
Tilapia Kank2          -------------------------------------------TGKSRHVGVW-----------------------------------------------
Amazon molly Kank2     -------------------------------------------SRGSRDVGVW-----------------------------------------------
Platyfish Kank2        -------------------------------------------SRGSRDVGVW-----------------------------------------------
Coelacanth KANK2       -------------------------------------------TREVKEVGIS-----------------------------------------------
Anole lizard KANK2     -------------------------------------------ETERCDVGVA-----------------------------------------------
Turtle KANK2           -------------------------------------------VTSPRSVGVG-----------------------------------------------
```

```
Tetradon Kank1b      -------------------------------------------KAEVRSVATE-----------------------------------------------
Medaka Kank1b        -------------------------------------------QVETRSIATE-----------------------------------------------
Amazon molly Kank1b  -------------------------------------------RVETRSIATE-----------------------------------------------
Platyfish Kank1b     -------------------------------------------QVETRSIATE-----------------------------------------------
Frog Kank4           -------------------------------------------GGNDGDLGCTS----------------------------------------------
Zebrafish Kank1b     -------------------------------------------KLETQSVAVG-----------------------------------------------
Tilapia Kank1b       -------------------------------------------KLETQSVAVG-----------------------------------------------
Fugu Kank1b          -------------------------------------------KLETQSVAVG-----------------------------------------------
Cavefish Kank1b      -------------------------------------------AVEKRSTAVG-----------------------------------------------
Spotted gar Kank4    -------------------------------------------SPSVQNVGVG-----------------------------------------------
Coelacanth KANK4     -------------------------------------------RAEVGSTAVT-----------------------------------------------
Frog Kank1           -------------------------------------------EIQVKNLGVG-----------------------------------------------
Shark Kank1          -------------------------------------------VSEQRDVSVG-----------------------------------------------
Coelacanth KANK1     -------------------------------------------AVEVRNAAVG-----------------------------------------------
Mouse KANK1          -------------------------------------------VTETRNVGIS-----------------------------------------------
Rat KANK1            -------------------------------------------VTETRNFGIS-----------------------------------------------
Dog KANK1            -------------------------------------------ATEMRSCAIS-----------------------------------------------
Human KANK1          -------------------------------------------LVEMRNCGVS-----------------------------------------------
Anole lizard KANK1   -------------------------------------------ELVTKDFVAG-----------------------------------------------
Eagle KANK1          -------------------------------------------EKEMRESGVG-----------------------------------------------
Chicken KANK1        -------------------------------------------EKEMRESGVG-----------------------------------------------
Goose KANK1          -------------------------------------------EKEMRESGVG-----------------------------------------------
Turtle KANK1         -------------------------------------------ETETRDSGVG-----------------------------------------------
Aligator KANK1       -------------------------------------------EKQMRDSGVG-----------------------------------------------
Spotted gar Kank1    -------------------------------------------EVETRSTGVG-----------------------------------------------
Herring Kank1        -------------------------------------------QRETRSAAVG-----------------------------------------------
Zebrafish Kank1a     -------------------------------------------EQEVRSTGVG-----------------------------------------------
Cave fish Kank1a     -------------------------------------------ENKTRSTGVG-----------------------------------------------
Stickleback Kank1a   -------------------------------------------RRETRSAAVG-----------------------------------------------
Medaka Kank1a        -------------------------------------------RLQTQSLSVG-----------------------------------------------
Fugu Kank1a          -------------------------------------------RPDTRSVALA-----------------------------------------------
Tetradon Kank1a      -------------------------------------------RPESSSVAVG-----------------------------------------------
Tilapia Kank1a       -------------------------------------------QLEIRSTAVG-----------------------------------------------
Amazon molly Kank1a  -------------------------------------------RLETRSAGVV-----------------------------------------------
Mouse KANK3          -------------------------------------------VPETVEVDTW-----------------------------------------------
Rat KANK3            -------------------------------------------VPETVEVDTW-----------------------------------------------
Dog KANK3            -------------------------------------------APETLESDAW-----------------------------------------------
Human KANK3          -------------------------------------------APETVEADAW-----------------------------------------------
Lamprey              ------------------------------------------------EVARG-----------------------------------------------
Coelacanth KANK3     -------------------------------------------EIETKNVGIW-----------------------------------------------
Frog Kank3           -------------------------------------------ERETKDAEVW-----------------------------------------------
Turtle KANK3         -------------------------------------------ETETKDAAVW-----------------------------------------------
Aligator KANK3       -------------------------------------------ETETRDAAVW-----------------------------------------------
Eagle KANK3          -------------------------------------------VRERRDAAVW-----------------------------------------------
Chicken KANK3        -------------------------------------------GRERRDVAVW-----------------------------------------------
Goose KANK3          ----------------------------------------------------------------------------------------------------
Spotted gar Kank3    -------------------------------------------RAEVEDASVW-----------------------------------------------
Zebrafish Kank3      -------------------------------------------TVDTEDVSVW-----------------------------------------------
Cavefish Kank3       -------------------------------------------PVQTEEVAVW-----------------------------------------------
Herring Kank3        -------------------------------------------EPSTEEASVW-----------------------------------------------
Stickleback Kank3    -------------------------------------------SAETAEAEVW-----------------------------------------------
Fugu Kank3           -------------------------------------------SVDTADAEVW-----------------------------------------------
Tetradon Kank3       -------------------------------------------RVDTTEAEVW-----------------------------------------------
Medaka Kank3         -------------------------------------------QVKTEEVAVW-----------------------------------------------
Tilapia Kank3        -------------------------------------------RVETEEAEVW-----------------------------------------------
Amazon molly Kank3   -------------------------------------------RAGTKEAEVW-----------------------------------------------
Platyfish Kank3      -------------------------------------------RAGTKEAEVW-----------------------------------------------
```

```
                      1310      1320      1330      1340      1350      1360      1370      1380      1390      1400
                      ....|....|....|....|....|....|....|....|....|....|....|....|....|....|....|....|....|....|....|....|
Hydra                 ------------------------------------VQTCNIGVSD------------------------------------------------------
Trichoplax            ------------------------------------CSDRDIEQ--------------------------------------------------------
Aplasia               ------------------------------------VNYDTSGVHTSLDFKGETELRMA-----------------------------------------
Snail                 ------------------------------------VSYDTSGILTSLDFKGETELRMA-----------------------------------------
Fruitfly              ------------------------------------PTTRNVGVQP------------------------------------------------------
Hookworm              ------------------------------------QTETVITADS------------------------------------------------------
C.elegance            -------------------------------------TGPLPAPKP------------------------------------------------------
Tunicate              ------------------------------------LIDAEFGVFP----PPPKEYKDC-----------------------------------------
Spider                ------------------------------------EASFIYSLYS----SNEKKYDQLT----------------------------------------
Lingula               ------------------------------------TRDTFTSTTTYETFRATTILQSLF----------------------------------------
Lancelet              DEDDMIPPLQQASIIKTTEPQVAEVQPVRHIVRETVVRETVVTVTPQ---DGYEELDGEVKHKEEEEKESLEAVDGFENKHALSPGSSPAISNLRPLRSC
Medaka Kank4b         ------------------------------------KSSTDLDE--------------------------------------------------------
Amazon molly Kank4b   ------------------------------------KTSTDLDE--------------------------------------------------------
Platyfish Kank4b      ------------------------------------KTSTDLDE--------------------------------------------------------
Stickleback Kank4b    ----------------------------------------------------------------------------------------------------
Fugu Kank4b           ------------------------------------RTSTDLDE--------------------------------------------------------
Tetraon Kank4b        ----------------------------------------------------------------------------------------------------
Cave fish Kank4b      ------------------------------------------GDRS------------------------------------------------------
Medaka Kank4a         ------------------------------------EQNQPQGVQENE----------------------------------------------------
Fugu Kank4a           ------------------------------------HDDAVKGGKS------------------------------------------------------
Tetradon Kank4a       ------------------------------------VKEGKR----------------------------------------------------------
Stickleback Kank4a    ------------------------------------LSQDIAGQELTSLREALKDAE-------------------------------------------
Tilapia Kank4a        ------------------------------------LKETETAAKE----GKPKD---------------------------------------------
Platyfish Kank4a      ------------------------------------IATSKLDTHD-----QQTEMEAE-----------------------------------------
Amazon Molly Kank4a   ------------------------------------IATSKLDTHD-----QQTEMETE-----------------------------------------
Herring Kank4         ------------------------------------EMEGKNADQVEHPIEDSQ----------------------------------------------
Zebradish Kank4       ------------------------------------VQRPSVIIIEENTTAQPAEQQ-------------------------------------------
Cave fish Kank4a      ------------------------------------EQQADTSEYTASQGKTEKAKAAEGSGDAGDKLTSTTQEQE------------------------
Mouse KANK4           ------------------------------------VTEESLGLLETD----------------------------------------------------
Rat KANK4             ------------------------------------VTEESLGLVETA----------------------------------------------------
Dog KANK4             ------------------------------------VTEESLGLPAVD----------------------------------------------------
Human KANK4           ------------------------------------VTEESLGLARVD----------------------------------------------------
Eagle KANK4           ------------------------------------EEKESCGPQPRDAGE-------------------------------------------------
Chicken KANK4         ------------------------------------AGE-------------------------------------------------------------
Goose KANK4           ----------------------------------------------------------------------------------------------------
Anole lizard KANK4    ------------------------------------QMEESSNLECLPSEE-------------------------------------------------
Turtle KANK4          ------------------------------------ANEENVDPEPLLCDTVE-----------------------------------------------
Aligator KANK4        ------------------------------------ENGEFLGLES----SVCSMVE-------------------------------------------
Frog Kank2            ------------------------------------VRETDFAPLPIRQLEADKQ---------------------------------------------
Mouse KANK2           ------------------------------------VRERDLGI--------------------------------------------------------
Rat KANK2             ------------------------------------VRERDLGI--------------------------------------------------------
Dog KANK2             ------------------------------------VRERDLGV--------------------------------------------------------
Human KANK2           ------------------------------------VRERDLGM--------------------------------------------------------
Aligator KANK2        ------------------------------------VPEPEMMELGAGAGE-------------------------------------------------
Spotted gar Kank2     ------------------------------------VREQDLGV--------------------------------------------------------
Cave fish Kank2       ------------------------------------VREEDLGLLP------------------------------------------------------
Herring Kank2         ------------------------------------VREQDLGLLP------------------------------------------------------
Zebrafish Kank2       ------------------------------------VREQDLGLLP------------------------------------------------------
Tetradon Kank2        ------------------------------------VREEDLGLLP------------------------------------------------------
Fugu Kank2            ------------------------------------VREEDLGLLP------------------------------------------------------
Medaka Kank2          ------------------------------------VQEKDLGLLP------------------------------------------------------
Sticklebakc Kank2     ------------------------------------VREEDLGLLP------------------------------------------------------
Tilapia Kank2         ------------------------------------VREKDLGLLP------------------------------------------------------
Amazon molly Kank2    ------------------------------------VREKDLGLLP------------------------------------------------------
```

```
Platyfish Kank2        ------------------------------------VREKDLGLRP------------------------------------------------------
Coelacanth KANK2       ------------------------------------VKEHDLGLSSV---ATQAEVEHQ-----------------------------------------
Anole lizard KANK2     ------------------------------------VKEEELGLPS-------PEEERQ-----------------------------------------
Turtle KANK2           ------------------------------------VSEEELAPEGQ-----------------------------------------------------
Tetradon Kank1b        ------------------------------------VSEIHLGIYT----EREAEIDAQ-----------------------------------------
Medaka Kank1b          ------------------------------------VSEVNLGIYA----EQEAELIAQ-----------------------------------------
Amazon molly Kank1b    ------------------------------------VTEVSLGIHT----KHEAELEAQ-----------------------------------------
Platyfish Kank1b       ------------------------------------VTEVSLGIHT----KHEAELEAQ-----------------------------------------
Frog Kank4             ------------------------------------VSHSIFLAIKQQITEFIEHFDDS-----------------------------------------
Zebrafish Kank1b       ------------------------------------VTETMLGVLS-------DKDEMQ-----------------------------------------
Tilapia Kank1b         ------------------------------------VTETMLGVLS-------DKDEMQ-----------------------------------------
Fugu Kank1b            ------------------------------------VTETMLGVLS-------DKDEMQ-----------------------------------------
Cavefish Kank1b        ------------------------------------VTEAMLGTVP----DSELELEMQ-----------------------------------------
Spotted gar Kank4      ------------------------------------VTEKDLGLTS----EQVSSDTG------------------------------------------
Coelacanth KANK4       ------------------------------------NTENELCLESSTLTE-------------------------------------------------
Frog Kank1             ------------------------------------VTEEMLGVTS----EAEREIELQ-----------------------------------------
Shark Kank1            ------------------------------------VTEALLGVST----EADQEIELQ-----------------------------------------
Coelacanth KANK1       ------------------------------------VTETLLGVTT----EADREIELQ-----------------------------------------
Mouse KANK1            ------------------------------------VTEAMLGVIT----EADKEIELQ-----------------------------------------
Rat KANK1              ------------------------------------VTEAMLGVIT----EADKEIELQ-----------------------------------------
Dog KANK1              ------------------------------------VTEAMLGVTT----EADKEIELQ-----------------------------------------
Human KANK1            ------------------------------------VTEAMLGVMT----EADKEIELQ-----------------------------------------
Anole lizard KANK1     ------------------------------------VTEGMLGLST----EAEREIDLQ-----------------------------------------
Eagle KANK1            ------------------------------------VTEAMLGLST----EVEKEIELQ-----------------------------------------
Chicken KANK1          ------------------------------------VTEAMLGLST----EVEKEIELQ-----------------------------------------
Goose KANK1            ------------------------------------VTEAMLGLST----EVEKEIELQ-----------------------------------------
Turtle KANK1           ------------------------------------VTEVMLGITT----EAEKEIELQ-----------------------------------------
Aligator KANK1         ------------------------------------VTEGMLGVTT----EAEKEIELQ-----------------------------------------
Spotted gar Kank1      ------------------------------------VTEALLGVST----EAETEIEMQ-----------------------------------------
Herring Kank1          ------------------------------------VTEAMLGMST----EAEAEIELQ-----------------------------------------
Zebrafish Kank1a       ------------------------------------VTEAMLGLSS----EAETEIEMQ-----------------------------------------
Cave fish Kank1a       ------------------------------------VTEAMLGMTT----EAEAEIELQ-----------------------------------------
Stickleback Kank1a     ------------------------------------VTEAMLGTSS----EAEREMELQ-----------------------------------------
Medaka Kank1a          ------------------------------------VTEAMLGVTS----EAEKEIELQ-----------------------------------------
Fugu Kank1a            ------------------------------------VTEAMLGVTS----EAEKEVELQ-----------------------------------------
Tetradon Kank1a        ------------------------------------VTEAMLGVTS----EAEKEIELQ-----------------------------------------
Tilapia Kank1a         ------------------------------------VTEAMLGMSS----EAEKEIELQ-----------------------------------------
Amazon molly Kank1a    ------------------------------------VTEAMLGVTS----EADKEIELQ-----------------------------------------
Mouse KANK3            ------------------------------------VTEELLGLPE----AAERELELL-----------------------------------------
Rat KANK3              ------------------------------------VTEALLGLPE----AAERELELL-----------------------------------------
Dog KANK3              ------------------------------------VTEALLGLPE----AAERELELL-----------------------------------------
Human KANK3            ------------------------------------VTEALLGLPA----AAERELELL-----------------------------------------
Lamprey                ----------------------------------------------------------------------------------------------------
Coelacanth KANK3       ------------------------------------VMESLLGVTS----DAEKEMELL-----------------------------------------
Frog Kank3             ------------------------------------VIESLLGMTC----EAEKEIELL-----------------------------------------
Turtle KANK3           ------------------------------------VVESLLGLPT----EAEKEMELL-----------------------------------------
Aligator KANK3         ------------------------------------VMESLLGLST----ESEKEIELL-----------------------------------------
Eagle KANK3            ------------------------------------VLESSLGLTS----EAERELELL-----------------------------------------
Chicken KANK3          ------------------------------------VLESSLGLAS----ETETELELL-----------------------------------------
Goose KANK3            ----------------------------------------------------------------------------------------------------
Spotted gar Kank3      ------------------------------------VMESLLGLRT----EAEREIETL-----------------------------------------
Zebrafish Kank3        ------------------------------------VIESFLGLQS----EAEREIDNL-----------------------------------------
Cavefish Kank3         ------------------------------------VMESLLGLSS----ETEQEISNL-----------------------------------------
Herring Kank3          ------------------------------------VMESLLGLTS----ETEREIDTL-----------------------------------------
Stickleback Kank3      ------------------------------------VIESLLGLTG----EAQREIDTL-----------------------------------------
Fugu Kank3             ------------------------------------VMESLLG---------------------------------------------------------
Tetradon Kank3         ------------------------------------VMESLLGVTS----EAQREMDAL-----------------------------------------
```

```
Medaka Kank3             ------------------------------------VVESLLGLST----DAQREIGAL-----------------------------------------
Tilapia Kank3            ------------------------------------VMESLLGLTT----EAQREIDTL-----------------------------------------
Amazon molly Kank3       ------------------------------------VMESLLGLTT----EAQREIDTL-----------------------------------------
Platyfish Kank3          ------------------------------------VMESLLGLTT----EAQREIDTL-----------------------------------------

                               1410      1420      1430      1440      1450      1460      1470      1480      1490      1500
                         ....|....|....|....|....|....|....|....|....|....|....|....|....|....|....|....|....|....|....|....|
Hydra                    ----------------------------------------------------------------------------------------CSIFDVFCDRCS
Trichoplax               ----------------------------------------------------------------------------------------QSNNSNKINQDE
Aplasia                  ----------------------------------------------------------------------------------------LRGVLQRNVRSV
Snail                    ----------------------------------------------------------------------------------------LRGVLQRSVHSV
Fruitfly                 ----------------------------------------------------------------------------------------SETVYSKEELDK
Hookworm                 ------------------------------------------------------------------------------------------NVESIASDDV
C.elegance               ----------------------------------------------------------------------------------------CGECAGLRKELF
Tunicate                 ----------------------------------------------------------------------------------------GSETNSIETINS
Spider                   ----------------------------------------------------------------------------------------SISSESLESDSE
Lingula                  ----------------------------------------------------------------------------------------QLQHISAPEIRN
Lancelet                 LKGKHSTKKVNIKKEIQFVGVNGNGHDESSSDDSSSEESSEDETDDEDSSSSESESSDEGGYNCELGKVIKKGKEDQGKKDVKTISSSEIICEDIKETDR
Medaka Kank4b            ----------------------------------------------------------------------------------------LLTVTSLQAKVA
Amazon molly Kank4b      ----------------------------------------------------------------------------------------LLTVTSLQAKVA
Platyfish Kank4b         ----------------------------------------------------------------------------------------LLTVTSLQAKVA
Stickleback Kank4b       ----------------------------------------------------------------------------------------------------
Fugu Kank4b              ----------------------------------------------------------------------------------------LLTVTSLQAKVA
Tetraon Kank4b           ----------------------------------------------------------------------------------------------------
Cave fish Kank4b         ----------------------------------------------------------------------------------------KSLTETLQRKVV
Medaka Kank4a            ----------------------------------------------------------------------------------------TCNTKKLQEKLM
Fugu Kank4a              ----------------------------------------------------------------------------------------EDSKQKLQEKLS
Tetradon Kank4a          ----------------------------------------------------------------------------------------EDSKQKLQEKLS
Stickleback Kank4a       ----------------------------------------------------------------------------------------TSKIQNLQEKLM
Tilapia Kank4a           ----------------------------------------------------------------------------------------PDDMQKLQEKLT
Platyfish Kank4a         ----------------------------------------------------------------------------------------VIVAIQPRPRAN
Amazon Molly Kank4a      ----------------------------------------------------------------------------------------VIEAIQPRPRAN
Herring Kank4            ----------------------------------------------------------------------------------------SLVVQTLQAKLQ
Zebradish Kank4          ----------------------------------------------------------------------------------------LVSNLELEAKLK
Cave fish Kank4a         ----------------------------------------------------------------------------------------SLAIQELQVKIK
Mouse KANK4              ----------------------------------------------------------------------------------------TSSISSLKNQVL
Rat KANK4                ----------------------------------------------------------------------------------------TSSISSLKSQVL
Dog KANK4                ----------------------------------------------------------------------------------------PGSISSLKQQLS
Human KANK4              ----------------------------------------------------------------------------------------PGSISSLKQQVS
Eagle KANK4              ----------------------------------------------------------------------------------------PGGVAALKQQVA
Chicken KANK4            ----------------------------------------------------------------------------------------PGGIAALKQHIA
Goose KANK4              ----------------------------------------------------------------------------------------PGGIAALKQQIA
Anole lizard KANK4       ----------------------------------------------------------------------------------------PSNIIALKQHIA
Turtle KANK4             ----------------------------------------------------------------------------------------PSRITALKQQIT
Aligator KANK4           ----------------------------------------------------------------------------------------PSTVTALKLQIA
Frog Kank2               ----------------------------------------------------------------------------------------KMLVQALSAKIA
Mouse KANK2              ----------------------------------------------------------------------------------------PDGEAALVAKVA
Rat KANK2                ----------------------------------------------------------------------------------------PDGEAALVAKVA
Dog KANK2                ----------------------------------------------------------------------------------------PDGEAALAAKVA
Human KANK2              ----------------------------------------------------------------------------------------PDGEAALAAKVA
Aligator KANK2           ----------------------------------------------------------------------------------------GDTVRALSARVA
Spotted gar Kank2        ----------------------------------------------------------------------------------------QSESEAWKTKAS
Cave fish Kank2          ------------------------------------------------------------------------------------------ETAALKTKVE
Herring Kank2            ------------------------------------------------------------------------------------------ETESLKTQVS
Zebrafish Kank2          ------------------------------------------------------------------------------------------ETEALKTKVG
Tetradon Kank2           ------------------------------------------------------------------------------------------ETEVLKSQVG
Fugu Kank2               ------------------------------------------------------------------------------------------ETEVLKSQVG
```

```
Medaka Kank2          ------------------------------------------------------------------------------------------ETEALRDQVG
Sticklebakc Kank2     ------------------------------------------------------------------------------------------ETKALRNQVG
Tilapia Kank2         ------------------------------------------------------------------------------------------ETEVLKNQVG
Amazon molly Kank2    ------------------------------------------------------------------------------------------EAEVLKDQVG
Platyfish Kank2       ------------------------------------------------------------------------------------------EAEVLKDQVG
Coelacanth KANK2      ----------------------------------------------------------------------------------------RQTIEALAAKIS
Anole lizard KANK2    ----------------------------------------------------------------------------------------RQAVQVLSTKIA
Turtle KANK2          ----------------------------------------------------------------------------------------RQAALALSAKVA
Tetradon Kank1b       ----------------------------------------------------------------------------------------QLIIGALKDRIS
Medaka Kank1b         ----------------------------------------------------------------------------------------QLLNGALKERIC
Amazon molly Kank1b   ----------------------------------------------------------------------------------------QLLNAALKERIS
Platyfish Kank1b      ----------------------------------------------------------------------------------------QLLNAALKERIS
Frog Kank4            ----------------------------------------------------------------------------------------TRDCHNLREILE
Zebrafish Kank1b      ----------------------------------------------------------------------------------------QQTIQNLTYQVQ
Tilapia Kank1b        ----------------------------------------------------------------------------------------QQTIQNLTYQVQ
Fugu Kank1b           ----------------------------------------------------------------------------------------QQTIQNLTYQVQ
Cavefish Kank1b       ----------------------------------------------------------------------------------------QQTIHVLSDRVQ
Spotted gar Kank4     ----------------------------------------------------------------------------------------PLTIIALRTKLT
Coelacanth KANK4      ----------------------------------------------------------------------------------------PFTVITLKAKIT
Frog Kank1            ----------------------------------------------------------------------------------------QQTIEALKDKIY
Shark Kank1           ----------------------------------------------------------------------------------------QQTIDALKDKVY
Coelacanth KANK1      ----------------------------------------------------------------------------------------QQTIDSLKEKIY
Mouse KANK1           ----------------------------------------------------------------------------------------QQTIEALKEKIY
Rat KANK1             ----------------------------------------------------------------------------------------QQTIEALKEKIY
Dog KANK1             ----------------------------------------------------------------------------------------QQTIEALKEKIY
Human KANK1           ----------------------------------------------------------------------------------------QQTIESLKEKIY
Anole lizard KANK1    ----------------------------------------------------------------------------------------HQTIEALKDKIY
Eagle KANK1           ----------------------------------------------------------------------------------------QQTIEALKEKIY
Chicken KANK1         ----------------------------------------------------------------------------------------QQTIEALKEKIY
Goose KANK1           ----------------------------------------------------------------------------------------QQTIEALKEKIY
Turtle KANK1          ----------------------------------------------------------------------------------------QQTIEALKGKIY
Aligator KANK1        ----------------------------------------------------------------------------------------QQTIEALKEKIY
Spotted gar Kank1     ----------------------------------------------------------------------------------------HQAIDALKDKIY
Herring Kank1         ----------------------------------------------------------------------------------------HQTIETLKEKIY
Zebrafish Kank1a      ----------------------------------------------------------------------------------------HQTIEALKEKIY
Cave fish Kank1a      ----------------------------------------------------------------------------------------HQTIESLKEKIY
Stickleback Kank1a    ----------------------------------------------------------------------------------------QQTIESLKEKIY
Medaka Kank1a         ----------------------------------------------------------------------------------------QQTIDALKDKIY
Fugu Kank1a           ----------------------------------------------------------------------------------------HQAIEALKEKIY
Tetradon Kank1a       ----------------------------------------------------------------------------------------HQAIEALKDKIY
Tilapia Kank1a        ----------------------------------------------------------------------------------------QQTIDSLKEKIY
Amazon molly Kank1a   ----------------------------------------------------------------------------------------QQTIEALKEKIY
Mouse KANK3           ----------------------------------------------------------------------------------------RTSLEHQRGVSE
Rat KANK3             ----------------------------------------------------------------------------------------RTSLEHQRGVSE
Dog KANK3             ----------------------------------------------------------------------------------------RASLEHQRGVSE
Human KANK3           ----------------------------------------------------------------------------------------RASLEHQRGVSE
Lamprey               --------------------------------------------------------------------------------------------AELSRYIE
Coelacanth KANK3      ----------------------------------------------------------------------------------------QHTINHQKEAIC
Frog Kank3            ----------------------------------------------------------------------------------------QHTVDHQRGVIG
Turtle KANK3          ----------------------------------------------------------------------------------------QHTIGHQREVIA
Aligator KANK3        ----------------------------------------------------------------------------------------QHTVSHQKEVIN
Eagle KANK3           ----------------------------------------------------------------------------------------QQTVGHQKEVIA
Chicken KANK3         ----------------------------------------------------------------------------------------QQTVGHQKDVIA
Goose KANK3           ----------------------------------------------------------------------------------------------------
Spotted gar Kank3     ----------------------------------------------------------------------------------------QDTIKFQQETIQ
Zebrafish Kank3       ----------------------------------------------------------------------------------------QHTITFQQESIQ
Cavefish Kank3        ----------------------------------------------------------------------------------------QQTITLQQESIQ
```

```
Herring Kank3            ----------------------------------------------------------------------------------------QDTVKLQQESIR
Stickleback Kank3        ----------------------------------------------------------------------------------------QDTIKFQQESIV
Fugu Kank3               -----------------------------------------------------------------------------------------------QESIA
Tetradon Kank3           ----------------------------------------------------------------------------------------QDTVRFQQESIV
Medaka Kank3             ----------------------------------------------------------------------------------------QDTVKLQQESIA
Tilapia Kank3            ----------------------------------------------------------------------------------------QDTIKFQQESIV
Amazon molly Kank3       ----------------------------------------------------------------------------------------QDTIKFQQESIV
Platyfish Kank3          ----------------------------------------------------------------------------------------QDTIKFQQESIV

                                  1510      1520      1530      1540      1550      1560      1570      1580      1590      1600
                         ....|....|....|....|....|....|....|....|....|....|....|....|....|....|....|....|....|....|....|....|
Hydra                    NLNLQNV---GVGEYVNNL-----------------LCCVDDKICG------------------------------------------------------
Trichoplax               VLNERTH-------------------RHSLSKEPSIDIPNADEHIM------------------------------------------------------
Aplasia                  ATNCNFKVPTVDCGTYMDL-------KTGISVGCGDEEHRTDVEIR------------------------------------------------------
Snail                    GTNCNFKVTTIDAATLTET-------GSGVSVGCGDEEMRIDVEVR------------------------------------------------------
Fruitfly                 KVEQALT--NAQKQQLRKL-------ISVGTQMYVPQRDQKDSGVQ------------------------------------------------------
Hookworm                 IGDTPVP---ECARCRQRE-------ETFTRNVGVGACAVSDKVCI------------------------------------------------------
C.elegance               LAVEKIPPPPPESCHVSTS-------TVLLEFADQSVGSEPIKHCH------------------------------------------------------
Tunicate                 YSQTKNELNTSATQTISPL-------TRTIGTGEHAFNAMVDKSCG------------------------------------------------------
Spider                   SLNGSFD----HSRLFLQQ-------RNLKSLTTSPIKTDSPNVVL------------------------------------------------------
Lingula                  IISTMLHRDVASKSTMTDVSFKSGVDKEVLTDKVHTVDEASSSDTI------------------------------------------------------
Lancelet                 LVSRELKETHELSEELQEA-------CGVLEKHVTTPKSVPPRELMMAMNTIQHEWMKAGAQKTSDVQVVRDYLAAFEQMATGVLGKIVNLADGNGNTAL
Medaka Kank4b            ALEQKLH---ETSLELQKV-------TGLLREQQAESGTKDKKIEN------------------------------------------------------
Amazon molly Kank4b      ALEQKLH---DTEQDLQRT-------AGLLKEQQQESRRKDEKIEY------------------------------------------------------
Platyfish Kank4b         ALEQKLH---DTEQDLQRT-------VGLLKEQQQESRRKDEKIEY------------------------------------------------------
Stickleback Kank4b       ----------------------------------------------------------------------------------------------------
Fugu Kank4b              ALEQKLH---ETDLELQRA-------LAQLREQKESREPGVKIEDL------------------------------------------------------
Tetraon Kank4b           ----------------------------------------------------------------------------------------------------
Cave fish Kank4b         TLEQKLH---ELESDLDKT-------RDQLKQQVLESRIKDERIKE------------------------------------------------------
Medaka Kank4a            TLEAKLT---QASQELERT-------NNLLKQQIQENKTKEERILQ------------------------------------------------------
Fugu Kank4a              TLEAKLA---QASQELDRT-------TGLLKEQLQENKLKEEKILQ------------------------------------------------------
Tetradon Kank4a          TLEAKLA---RATQELDRT-------SVLLKEQLQENKLKEEKILQ------------------------------------------------------
Stickleback Kank4a       ILEAKLT---QASQDLERT-------NTLLKEQMDENKVKEERILQ------------------------------------------------------
Tilapia Kank4a           ILEAKLI---KASQELERT-------NSLLKQQVEENKLKEERILQ------------------------------------------------------
Platyfish Kank4a         SMERGTITENIVTQDQTTETPAAERVNQVTETEGETVTDHPQRPRA------------------------------------------------------
Amazon Molly Kank4a      SMERGTITESIVTQDQTTETPAAERVNQVTETEGETVTDHPQRPRA------------------------------------------------------
Herring Kank4            ALEEKLN---LSSLELKQT-------NALLREQVEENRVKDERIQE------------------------------------------------------
Zebradish Kank4          TLEESLS---KASCELEKT-------NVLLREEMDKNRQKDERIQE------------------------------------------------------
Cave fish Kank4a         TLEESLS---QASCELEKT-------NSLLREQIEENKLKDLTIQE------------------------------------------------------
Mouse KANK4              ALEDKLS---GRTEELARV-------RAALEQQEEETKAREQRIQE------------------------------------------------------
Rat KANK4                DLEDKLS---GRTEELAQV-------RAALEQQEEETKAREQRIQE------------------------------------------------------
Dog KANK4                ALEGELS---GRTQELAQV-------RAALQQQEDESKARGQRIQE------------------------------------------------------
Human KANK4              ALEGELS---GRTEELAQV-------RTALQQQEEEIKAREQRIRE------------------------------------------------------
Eagle KANK4              ALEEQLG---KKKEELERI-------KAVLEQQDHEIKEKEKSIKL------------------------------------------------------
Chicken KANK4            ALEEQLD---RKKEELEQI-------RAVLKQQDHKIKEKEKSIQL------------------------------------------------------
Goose KANK4              TLEEQLS---KKKEELEQI-------RAVLEQQDHEIKEKEKSIKL------------------------------------------------------
Anole lizard KANK4       ILEEQLN---NKTEELAQI-------RVVLKEQDKEIKAKEKSIEM------------------------------------------------------
Turtle KANK4             VLEEQLN---SKIEELEQI-------KVVLKQQDSEIKAKERSIKI------------------------------------------------------
Aligator KANK4           VLEEQLS---TKTQELEHV-------RLVLKQQDDEIKEKERNIKT------------------------------------------------------
Frog Kank2               VLEKQLG---RALIEVQNA-------NRKLEEAERAGRAEEAAKRE------------------------------------------------------
Mouse KANK2              VLETQLK---KALQELRAA-------QTQQVDLQPQAWPPPDTQVR------------------------------------------------------
Rat KANK2                VLETQLK---KALQELRAA-------QTQQVDLQPQAWPPPDTQVR------------------------------------------------------
Dog KANK2                VLETQLK---KALQELQAA-------QARQADPQPQPQPQPQPRPP------------------------------------------------------
Human KANK2              VLETQLK---KALQELQAA-------QARQADPQPQAWPPPDSPVR------------------------------------------------------
Aligator KANK2           LLERQLQ---KALGELQDA-------RQRLGQGAG-----------------------------------------------------------------
Spotted gar Kank2        LLETQLR---KTLQELHTA-------HRKLEALGKETAEKGQDAKP------------------------------------------------------
Cave fish Kank2          ELEVQLK---RTVQELQSA-------QKKVETVQKERTSGAGPP--------------------------------------------------------
```

```
Herring Kank2          RLEAQLK---RAMQEIQSS-------QEQVETAQRERERQAQLQRQ------------------------------------------------------
Zebrafish Kank2        LLEVQLR---KTMQELQSA-----------QQQVEAAQKERQTVCP------------------------------------------------------
Tetradon Kank2         QLEGQLR---RTMQELQAA-------QQQVTAVQKGPRAE------------------------------------------------------------
Fugu Kank2             QLEGELK---RTMQELQAV-------QQQVTAVQNAP---------------------------------------------------------------
Medaka Kank2           QLEGKLK-------------------RTLEELQTAQQQSATVQETL------------------------------------------------------
Sticklebakc Kank2      QLEGQLK-------------------RTVQELRDAQKAPQADRPVM------------------------------------------------------
Tilapia Kank2          QLEGQLK---RTMQELQAA-------QQQVATVQKAP---------------------------------------------------------------
Amazon molly Kank2     QLEGKLK---RTLQELQAA---------QLQKAP------------------------------------------------------------------
Platyfish Kank2        QLEGKLT---RTLQELQAA------QLQKAPQAEHPPQQREQRTVG------------------------------------------------------
Coelacanth KANK2       VLEKQLK---KALQELQVA-------HQKLEQQAKEITEREKATKP------------------------------------------------------
Anole lizard KANK2     VLETQLK------RALQEL-------QAAHQKLEHQSREQEDKEMS------------------------------------------------------
Turtle KANK2           VLEKQLQ---KALRELQGA-------QQKLAGRPGWQQQEVPVKAD------------------------------------------------------
Tetradon Kank1b        HLEAELK-ESALQAEMSRL-------KSELKAAG--VRTRADKSSH------------------------------------------------------
Medaka Kank1b          HLEAELK-ESAFQIELTRL-------KLELHVAG--ARNRADKACL------------------------------------------------------
Amazon molly Kank1b    QLETELK-ESALQTELTRL-------KLELQAAG--ARNRVDKSCF------------------------------------------------------
Platyfish Kank1b       QLETELR-ESALQTELTRL-------KLELQAAG--ARNRVDKSCF------------------------------------------------------
Frog Kank4             RQKEEMN---AKDLHIIEL-------KNTIDTLEEMIRTRQKDDV-------------------------------------------------------
Zebrafish Kank1b       ILEAELK-EAMLKSEMGRM-------RSELREAEARNRTYKSSTAQ------------------------------------------------------
Tilapia Kank1b         ILEAELK-EAMLKSEMGRM-------RSELREAEARNRTYKSSTAQ------------------------------------------------------
Fugu Kank1b            ILEAELK-EAMLKSEMGRM-------RSELREAEARNRTYKSSTAQ------------------------------------------------------
Cavefish Kank1b        TLEAELK-EALLNAEMCRL-------KDELREAGSHNRADKSSSAQ------------------------------------------------------
Spotted gar Kank4      VVEKQLN---DTTKELDKV-------NSLLKEQMKENVRKEERIKI------------------------------------------------------
Coelacanth KANK4       VLENQLN---DARKELEKN-------SDTHKQQNEEHKAKNINPHQ------------------------------------------------------
Frog Kank1             RLETDLR-ETTYAMEMTKL-------KLEIKAAG--SRKNADKAIM------------------------------------------------------
Shark Kank1            RLEAQVK-ETMHQAEMSKL-------KFELQAAG--SWKKADKCVT------------------------------------------------------
Coelacanth KANK1       RLEVQLK-ETTHDMEMTKL-------KLELQAAG--SRKKTDKGLM------------------------------------------------------
Mouse KANK1            RLEVQLK-ETTHDREMTKL-------KQELQAAG--SRKKVDKATM------------------------------------------------------
Rat KANK1              RLEVQLK-ETTHDQEMTKL-------KQELQAAG--SRKKVDKAIM------------------------------------------------------
Dog KANK1              RLEVQLK-ETTHDREMTKL-------KQELQAAG--SRKKVDKAVM------------------------------------------------------
Human KANK1            RLEVQLR-ETTHDREMTKL-------KQELQAAG--SRKKVDKATM------------------------------------------------------
Anole lizard KANK1     RLEVQLR-ETTHDREMTKL-------KHELQAAG--SRKKLDKALM------------------------------------------------------
Eagle KANK1            RLEVQLK-ETTHDREMTKL-------KQELQAAG--SRKKLDKAVM------------------------------------------------------
Chicken KANK1          RLEVQLK-ETTHDREMTKL-------KQELQAAG--SRKKVDKAMM------------------------------------------------------
Goose KANK1            RLEVQLK-ETTHDREMTKL-------KQELQAAG--SRKKVDKATM------------------------------------------------------
Turtle KANK1           RLEVQLK-ETTHDREMTKL-------KQELQAAG--SRKKVDKALM------------------------------------------------------
Aligator KANK1         RLEVQLK-ETTHDREMTKL-------KQELQAAG--SRKKLDKAVM------------------------------------------------------
Spotted gar Kank1      RLEVQLK-ETTHEMEMGKL-------KLELQAAG--SRKKADKGSM------------------------------------------------------
Herring Kank1          RLEVQLK-ETTHQMEMCKL-------KLELQAAG--NRKKSDKACM------------------------------------------------------
Zebrafish Kank1a       RLEVQLK-ETTHQMEMGKL-------KLQLQVAG--SRKKADKGLM------------------------------------------------------
Cave fish Kank1a       RLEVQLK-ETTHEMEMGKL-------KLELQVAG--SRKKVDKGSM------------------------------------------------------
Stickleback Kank1a     RMEVQLK-ETTHQLEMGKL-------KLELQAAGGSGRKRVDKGST------------------------------------------------------
Medaka Kank1a          RLEVQLK-ETTHQTEMAKL-------KLELQAAGGSTKKRADKGLM------------------------------------------------------
Fugu Kank1a            RLEVQLK-ETTHEMEMGKL-------KLELQAAGGSNKKKADKGFM------------------------------------------------------
Tetradon Kank1a        RLEVQLK-ETTHEMEMGKL-------KLELQAAGGSNKKKADKGFM------------------------------------------------------
Tilapia Kank1a         RLEVQLK-ETTHQMEMGKL-------KLELQAAGGSNKKKADKGLM------------------------------------------------------
Amazon molly Kank1a    RLEVQLK-ETTHEMEMGKL-------KLELQAAGGSSKKKADKGLM------------------------------------------------------
Mouse KANK3            LLRGRLR-------ELEEA--------HEAAVTRPQSRDVAAQTTL------------------------------------------------------
Rat KANK3              LLRGRLR-------ELEEA-------HEAIVTRS------------------------------------------------------------------
Dog KANK3              LLRCRLR-------ELEEA-------REAAEAEAAAAAEAQRQPQP------------------------------------------------------
Human KANK3            LLRGRLR-------ELEEA-------REAAEEAAAGARAQLREATT------------------------------------------------------
Lamprey                RVQRLLH---EQQALLTDN-------YAELADAFAEPATELGSLSS------------------------------------------------------
Coelacanth KANK3       MLEGHLK---EATEELEKL-------RVEVCSRR--PRKMVNKEVM------------------------------------------------------
Frog Kank3             LLEGHLK---EAANELEEL-------RVAVYSRR--QKDMADKEVM------------------------------------------------------
Turtle KANK3           MMEGHLK---DATKELEEL-------RVEVCSRR--PRTLIDKEIM------------------------------------------------------
Aligator KANK3         MMEGHLK---EATKELEEL-------RVEVCSRR--PGKLIDKEIM------------------------------------------------------
Eagle KANK3            LMEGHLR---EATRELEEL-------RLEVCARR--PRGRVDKEVT------------------------------------------------------
Chicken KANK3          LMEGHLR---EATRELEEL-------RMEVCARQ--PRSRADKEVT------------------------------------------------------
```

```
Goose KANK3           ----------------------------------------------------------------------------------------------------
Spotted gar Kank3     VLESSLS---QVDKEMEAL---------RVQDDERKSKVAVEKGTQ------------------------------------------------------
Zebrafish Kank3       VLETRLT---QVNQDLETL---------RAQEIERTSKIMLDKETT------------------------------------------------------
Cavefish Kank3        VLETRLS---QANQHLEEL---------KAKEKERTSKISIERGTL------------------------------------------------------
Herring Kank3         VLEERLS---RADQDLEEL---------RAREHEHRSRVTLDRAVQ------------------------------------------------------
Stickleback Kank3     ALEVRLS-----------------------------EKMEAHKVAV------------------------------------------------------
Fugu Kank3            ALEKRLS---AADKDLLTL-------KAQMQEKN--SKETFEKGVL------------------------------------------------------
Tetradon Kank3        ALEKRLS---EADKDLLAL-------KAQLEERN--SKDTSEKGVS------------------------------------------------------
Medaka Kank3          ALEDRLS---VANKDLGVL-------RAQMVEKT--SKVTFDKGVL------------------------------------------------------
Tilapia Kank3         ALEDRLS---VADTDLGVL-------RAKMEGNI--SKITFEKGVL------------------------------------------------------
Amazon molly Kank3    ALESRLT---AADKDIDNL-------RAQIDERT--SKVTAEKGAL------------------------------------------------------
Platyfish Kank3       ALESRLT---VADKDLDNL-------RAWIDERT--SKVMAEKGAL------------------------------------------------------

                            1610      1620      1630      1640      1650      1660      1670      1680      1690      1700
                      ....|....|....|....|....|....|....|....|....|....|....|....|....|....|....|....|....|....|....|....|
Hydra                 -----------------------------------------------------------------------------------CFKPTCISVAISNDSV-
Trichoplax            ----------------------------------------------------------------------------------------------------
Aplasia               -------------------------------------------------------------------MPVVRKNVYISAKP--ETGHKCVSTEKGWAMD-
Snail                 ---------------------------------------------------------------------PATVKKSQGVTVKPECLPKCVSTEKDWVLD-
Fruitfly              ---------------------------------------------------------------------------TTPERPK-LKYHVGVTVKPATREN-
Hookworm              -----------------------------------------------------------------------------------ECDKATPDEVDENDAP-
C.elegance            -----------------------------------------------------------------------------------VALGTEPTSTREVGTV-
Tunicate              -------------------------------------------------------------------------------GSAVEKRDIGVEASVLQNTK-
Spider                --------------------------------------------------------------------------KDSSTSTDAICLNKSVNTELFLPKF-
Lingula               ---------------------------------------------------------------------------DVEVRPIKEYRSVYVDAEPSTANKA
Lancelet              HYSVSHSNFDIVEILLETGQCEVNKQNKAGYTAIMLASLAKLQGDRQMEVVKKLFQAGDVNIRATQAGQTALMLAVSHGRQ--EIVKMLIEAGAEVNLQ-
Medaka Kank4b         ----------------------------------------------------------------------------------------LINNPGMWVCA-
Amazon molly Kank4b   ----------------------------------------------------------------------------------------LIRNPGVWVRA-
Platyfish Kank4b      ----------------------------------------------------------------------------------------LIRNPGVWVRA-
Stickleback Kank4b    ----------------------------------------------------------------------------------------------------
Fugu Kank4b           -----------------------------------------------------------------------------------------VRKPGTWVQA-
Tetraon Kank4b        ----------------------------------------------------------------------------------------------------
Cave fish Kank4b      ----------------------------------------------------------------------------------------------------
Medaka Kank4a         ------------------------------------------------------------------------------LNASQAHDDSKAKQIPQTTEK-
Fugu Kank4a           --------------------------------------------------------------------------LSEGVREEETETESLHTADQGTDTD-
Tetradon Kank4a       ------------------------------------------------------------------------------AEMVTAKNDSGTKEILPTKSE-
Stickleback Kank4a    -----------------------------------------------------------------------------------LSDGVTAEACTPEAQN-
Tilapia Kank4a        ---------------------------------------------------------------------------LRTVTERIDFTHQETETESPGTVD-
Platyfish Kank4a      -----------------------------------------------------------------------SSIDQGTETERVDTVDRITETEAAQRSD-
Amazon Molly Kank4a   -----------------------------------------------------------------------SSIDQGTETERVDTVDRVTETEVAQRSD-
Herring Kank4         -------------------------------------------------------------------------LSERAEELAETAKRDTVENNVSAESQ-
Zebradish Kank4       --------------------------------------------------------------------------------LTDQVKEQILQGPIETEPQ-
Cave fish Kank4a      -----------------------------------------------------------------------------LSDKVMESFAASIQQETTPPPE-
Mouse KANK4           ----------------------------------------------------------------------------------LECTVAHLEEKLSQERA-
Rat KANK4             ----------------------------------------------------------------------------------LECTVAHLEEKLSQEKA-
Dog KANK4             ----------------------------------------------------------------------------------LECTVARLAGKLSHENT-
Human KANK4           ----------------------------------------------------------------------------------LEFTVAQLEGQFHQENA-
Eagle KANK4           ---------------------------------------------------------------------------LASSKA--QLEEKLCRENAKEATL-
Chicken KANK4         -----------------------------------------------------------------------------LASSKAQLEGQLCRENTREDAV-
Goose KANK4           ---------------------------------------------------------------------------LASSKA--QLEEQLCRENTQVATA-
Anole lizard KANK4    -----------------------------------------------------------------------------LASSKAQLEEKLCGESTKEIRP-
Turtle KANK4          -----------------------------------------------------------------------------LASSKAQLEEKLCQENTKETEP-
Aligator KANK4        ---------------------------------------------------------------------------LASSKA--QLEEKFCRENTQETKP-
Frog Kank2            -----------------------------------------------------------------------------------KKEEVRADAVKVARVE-
Mouse KANK2           -----------------------------------------------------------------------------------VDTVRVVEGPREVEVA-
Rat KANK2             -----------------------------------------------------------------------------------VDTVRVVEGPREVEVA-
Dog KANK2             -----------------------------------------------------------------------------------PDSPIRVDTVRVVEGP-
```

```
Human KANK2            -----------------------------------------------------------------------------------VDTVRVVEGPREVEVV-
Aligator KANK2         ----------------------------------------------------------------------------------------------------
Spotted gar Kank2      -----------------------------------------------------------------------------------SMADAVMMTSIGWQEL-
Cave fish Kank2        -----------------------------------------------------------------------------------QADHAVRATSLGWQGQ-
Herring Kank2          --------------------------------------------------------------------------------P--QAEHPVKATSLGWQEQ-
Zebrafish Kank2        -----------------------------------------------------------------------------------QVDHAVRATSLGWQDQ-
Tetradon Kank2         --------------------------------------------------------------------------------------HPVMATSTGWQEP-
Fugu Kank2             -----------------------------------------------------------------------------------QAEHPVMATNTGWQEP-
Medaka Kank2           -----------------------------------------------------------------------------------QVDHPVMATSIGWQEP-
Sticklebakc Kank2      ------------------------------------------------------------------------------------------ATSVGWREP-
Tilapia Kank2          -----------------------------------------------------------------------------------QAEHPVIATSLGWQEP-
Amazon molly Kank2     -----------------------------------------------------------------------------------QAEHPVMATSIGWQEP-
Platyfish Kank2        ------------------------------------------------------------------------------IQVYTLEKPTVVEVGTLLRAE-
Coelacanth KANK2       -----------------------------------------------------------------------------------STADAIIMTCVGWQEL-
Anole lizard KANK2     ------------------------------------------------------------------------------------TGDVGHPTEREVREE-
Turtle KANK2           -----------------------------------------------------------------------------------TVKVVQVQREPEIAAS-
Tetradon Kank1b        ------------------------------------------------------------------------------ARP--STAGASSEARPTTASQ-
Medaka Kank1b          ------------------------------------------------------------------------------ARP--STASIGTEIKPFTKSQ-
Amazon molly Kank1b    ------------------------------------------------------------------------------AKP--HTASIGTTTRLSTQSQ-
Platyfish Kank1b       ------------------------------------------------------------------------------AKP--HTASVGTTARPFTQSQ-
Frog Kank4             -----------------------------------------------------------------------------------EYKDIAVNTEQPPKDE-
Zebrafish Kank1b       ------------------------------------------------------------------------------------PLVKCTATQTGSQNH-
Tilapia Kank1b         ------------------------------------------------------------------------------------PLVKCTATQTGSQNH-
Fugu Kank1b            ------------------------------------------------------------------------------------PLVKCTATQTGSQNH-
Cavefish Kank1b        ----------------------------------------------------------------------------------PKMQSSAFQTTTQTRSV-
Spotted gar Kank4      ------------------------------------------------------------------------------LTEEAEARANLKEAKVLQRAA-
Coelacanth KANK4       ---------------------------------------------------------------------------LPGQKIRMDSKPAVQEEKCILVDK-
Frog Kank1             ------------------------------------------------------------------------------AQP--LMFSRTTEASVEMKDQ-
Shark Kank1            ------------------------------------------------------------------------------ARP--AVCSVSVQAAVQTKSQ-
Coelacanth KANK1       ------------------------------------------------------------------------------VQP--LVSSRAVEAVVQMRDQ-
Mouse KANK1            ------------------------------------------------------------------------------AQP--LVFSKLVEALVPTRDQ-
Rat KANK1              ------------------------------------------------------------------------------AQP--LVFSKLVEALVPTREQ-
Dog KANK1              ------------------------------------------------------------------------------AQP--LVGDKMVEAVVPTRDQ-
Human KANK1            ------------------------------------------------------------------------------AQP--LVFSKVVEAVVQTRDQ-
Anole lizard KANK1     ------------------------------------------------------------------------------AQP--LLFSRAVEAVVQTRDQ-
Eagle KANK1            ------------------------------------------------------------------------------AQP--RVISRMVEAVIQTRDQ-
Chicken KANK1          ------------------------------------------------------------------------------AQP--LVVSRMVEAVVQMRDQ-
Goose KANK1            ------------------------------------------------------------------------------AQP--LVVSRTVEAVVPMRDQ-
Turtle KANK1           ------------------------------------------------------------------------------AQP--LVFSRMVEAVVQTRDQ-
Aligator KANK1         ------------------------------------------------------------------------------TQP--LVFSRMVEAVVQTRDQ-
Spotted gar Kank1      ------------------------------------------------------------------------------ARP--AVYSTSVEAKVQMQSQ-
Herring Kank1          ------------------------------------------------------------------------------ARP--EMYSTSVEARVSTHSL-
Zebrafish Kank1a       ------------------------------------------------------------------------------ARP--EMYSTSVEARVSTHTQ-
Cave fish Kank1a       ------------------------------------------------------------------------------ARP--EMYSTSVEAKVSTQSQ-
Stickleback Kank1a     ------------------------------------------------------------------------------ARP--ETYDACVEARVQTRSV-
Medaka Kank1a          ------------------------------------------------------------------------------VRP--EVYSVSVEAKVQLHSR-
Fugu Kank1a            ------------------------------------------------------------------------------VRP--ETYNVYVEAKVQMRTQ-
Tetradon Kank1a        ------------------------------------------------------------------------------VRP--EMYSVFVEAKVQMRTQ-
Tilapia Kank1a         ------------------------------------------------------------------------------VRP--EMYSVCVEAKVQTRSQ-
Amazon molly Kank1a    ------------------------------------------------------------------------------VRP--EMYNASVEAKVQMRSQ-
Mouse KANK3            -----------------------------------------------------------------------------------GCTEKTTQTELPVENQ-
Rat KANK3              -----------------------------------------------------------------------------------QSREVATQTTLGYTEK-
Dog KANK3              ---------------------------------------------------------------------------------QPQPQPREAATQTQWG---
Human KANK3            -----------------------------------------------------------------------------------QTPWSCAEKAAQTESP-
Lamprey                ----------------------------------------------------------------------------------------------------
Coelacanth KANK3       ------------------------------------------------------------------------------IQP--HTANAFVEAIISVANQ-
Frog Kank3             ------------------------------------------------------------------------------AHP--QTLETASEAVVSMLNQ-
```

```
Turtle KANK3          ------------------------------------------------------------------------------AKP--QMVEAQAEAAVPMQSQ-
Aligator KANK3        ------------------------------------------------------------------------------AKP--QLAEAYVEAVVPMQNQ-
Eagle KANK3           ------------------------------------------------------------------------------AKP--QVAEALVEAAVVTQNR-
Chicken KANK3         ------------------------------------------------------------------------------ARP--QVAEALVETQPGVCDA-
Goose KANK3           ----------------------------------------------------------------------------------------------------
Spotted gar Kank3     ------------------------------------------------------------------------------ARP--EMASVLVEAVVSLTST-
Zebrafish Kank3       ------------------------------------------------------------------------------AKP--ETVSIRLETCPEVCSV-
Cavefish Kank3        ----------------------------------------------------------------------------------------------------
Herring Kank3         ------------------------------------------------------------------------------VRT--QTAHVGTDALPAAPPK-
Stickleback Kank3     ----------------------------------------------------------------------------------------LKKRQMTIAEY-
Fugu Kank3            ------------------------------------------------------------------------------AKA--DTADAQVETATPHFKH-
Tetradon Kank3        ----------------------------------------------------------------------------------------------------
Medaka Kank3          ------------------------------------------------------------------------------VTP--DVKNVQLETLTSALKH-
Tilapia Kank3         ----------------------------------------------------------------------------------------------------
Amazon molly Kank3    ------------------------------------------------------------------------------AKP--DTRNAQVETLAPVLKH-
Platyfish Kank3       ------------------------------------------------------------------------------AKP--DMRNAQVETLGPVLKH-

                              1710      1720      1730      1740      1750      1760      1770      1780      1790      1800
                      ....|....|....|....|....|....|....|....|....|....|....|....|....|....|....|....|....|....|....|....|
Hydra                 -----------ND -------------------------------------------VFCDHCKNLKTSS--TGVGDDKIDDVF-----------------
Trichoplax            ----------------------------------------------------------------------------------------------------
Aplasia               -----------VG-----------------------TNTAPVDKYHKASMTDKQRCSFAATNTEVTHTR---QANNQTEVKMF---LAMDQLRHAASNTQ
Snail                 ----------------------------------ASTNTFMIDQYHKACMTDKQKCIMAATNTEPLPYR---HSSIQTDLNVF-----------------
Fruitfly              -----------FT-------------------------SCKPDVRHVGSSDDRLDDVLCEKCAVSKRSV---ACGPETPAEKV-----------------
Hookworm              -----------GF--------------------KPEKDELRTKEFDLARAAAVKKLLTSEQKQPFVRGT---AVSRSARLERN-----------------
C.elegance            --------------------------------------PVTVRSLHIDTQTDPAASTVDAACSPVLFETQEEAVKVTVAASSS-----------------
Tunicate              -----------LT----------STVGLILTNDFASGNHVESSTVGTSTPRVTRS-DIATSCEGLLKTV---DIGVSAGAKLT-----------------
Spider                -----------IL------------------------NNSTFQNKSTLISPSVRTKAVQAVEKKAVRNI---GLQISGSVNVE-----------------
Lingula               TGINFISKMDFGTNTVTVQKKNSATSTTPKLSFPAETQTISVPYISMGTETDMKIFLATEQIRNQSTNT---DVHSTVTQAVGTRDDDSDFYLRNFGTNT
Lancelet              -----------DE----------------------DGSTALMCASEHGHLEIVKLLLAQPGCDATITDN---DDSTALSIAMEAGHRDIGVLLYAQVNMG
Medaka Kank4b         -----------ER-------------------------------------------VMVEQDGDETVVR---SMEPDYSKGCS-----------------
Amazon molly Kank4b   ------------------------------------------------------ERVVVDQEGDETVVR---SIEPERIPASS-----------------
Platyfish Kank4b      ----------------------------------------------------ER--VVVDQEGDETIVR---SIEPERIPASS-----------------
Stickleback Kank4b    ----------------------------------------------------------------------------------------------------
Fugu Kank4b           ---------------------------------------------------------------ERVVIE---QVGDQAVVRSV-----------------
Tetraon Kank4b        ------------------------------------------------------------------------AAGPERT---------------------
Cave fish Kank4b      ------------------------------------------------------------------LNK---NVERNSAENVW-----------------
Medaka Kank4a         ------------------------------------AEPGKVDDEKNQSPMTSSGPPKKDQVRSVSQVE---APSPTVKLTEE-----------------
Fugu Kank4a           -----------RI-----------------------CIEVHPPKERTGSIDHGTETIKTDTCEQPQPQS---KNQPQAQIKGP-----------------
Tetradon Kank4a       -----------IS-------------------------QIVDTQPLTIETEDKGPKEDGETLRETIKEA---VIGEKTVVPVR-----------------
Stickleback Kank4a    -----------RP----------------------RRESIDTGTETERVDFTHQETETEIKGVPVHLLQ---NPGPKPRVEDS-----------------
Tilapia Kank4a        -------------------------------------QGTDTDKICIERPRASSVDRGTETERVGTVDR---VTETEVAQRAD-----------------
Platyfish Kank4a      -----------QQTETEIERQQDNNLSRDAQAEGRVGESETTVRGDIGHPVKSTAESEKTKKVVQIESE---NVAVEVAANIF-----------------
Amazon Molly Kank4a   -----------QQTETEIERRQDNNLSGDAQAEGRVGESETTESEKTKKVVQIESENVAVEVAAKIYVT---DVEGIVVQTLD-----------------
Herring Kank4         ------------------------------------PQPVLTSDASVCTDSERIPVSEKAVCTDEEAQP---SNGPKETDLTC-----------------
Zebradish Kank4       ------------------------------------PEPVVKCDASVSTDSKSVLEKGISTEPQPIEDP---KVTESQCSSTQ-----------------
Cave fish Kank4a      --------------------------------------PRPLPQLIVTCDVSVSTDSKSVSEKAVLTDA---ELQPDDGPKET-----------------
Mouse KANK4           ------------------------------------------SEAPDRTDATVNTDPLQELTPRESHDK---NIGVNLLNTPD-----------------
Rat KANK4             ------------------------------------SDALDRTDAMVNTDPLRE--LIPRESQDKNIGV---NLLSTPVPECW-----------------
Dog KANK4             -------------------------------------RDSQGQTDAMVNTDPLNGLLTRESCDKSIEVN---LLGSTGSESWR-----------------
Human KANK4           -------------------------------------KDTQGQTDVMVNTDPVHGLLTRESCDKGIEVN---LLGSMESESWG-----------------
Eagle KANK4           -----------PS------------------------TRSSGALQCYDAAVNTD--LSQVAGAKQAHDK---GVNVNIPVSTK-----------------
Chicken KANK4         -------------------------------------SSMQRGSMLQCNDVAVNTELSYVNGSKQVHDK---GINVNIPLSTK-----------------
Goose KANK4           -----------PS-----------------------GRRSSTLQYYDAAVNTE---PSHVNGSKEAHDK---GVNVNIPLSTK-----------------
Anole lizard KANK4    --------------------------------------YTTAKNVLQYHDAAVNTELVHEKIAKEAYDK---GVNVRLTVPME-----------------
Turtle KANK4          --------------------------------SKQDGNVLQYHDAAVNTE------LMQANTFKEAYDK---GVNVNIPVATE-----------------
Aligator KANK4        -----------CE---------------------QDGNALPYHDAAVNTE------LIGTDRLKESQDK---GVNVNISVSTK-----------------
```

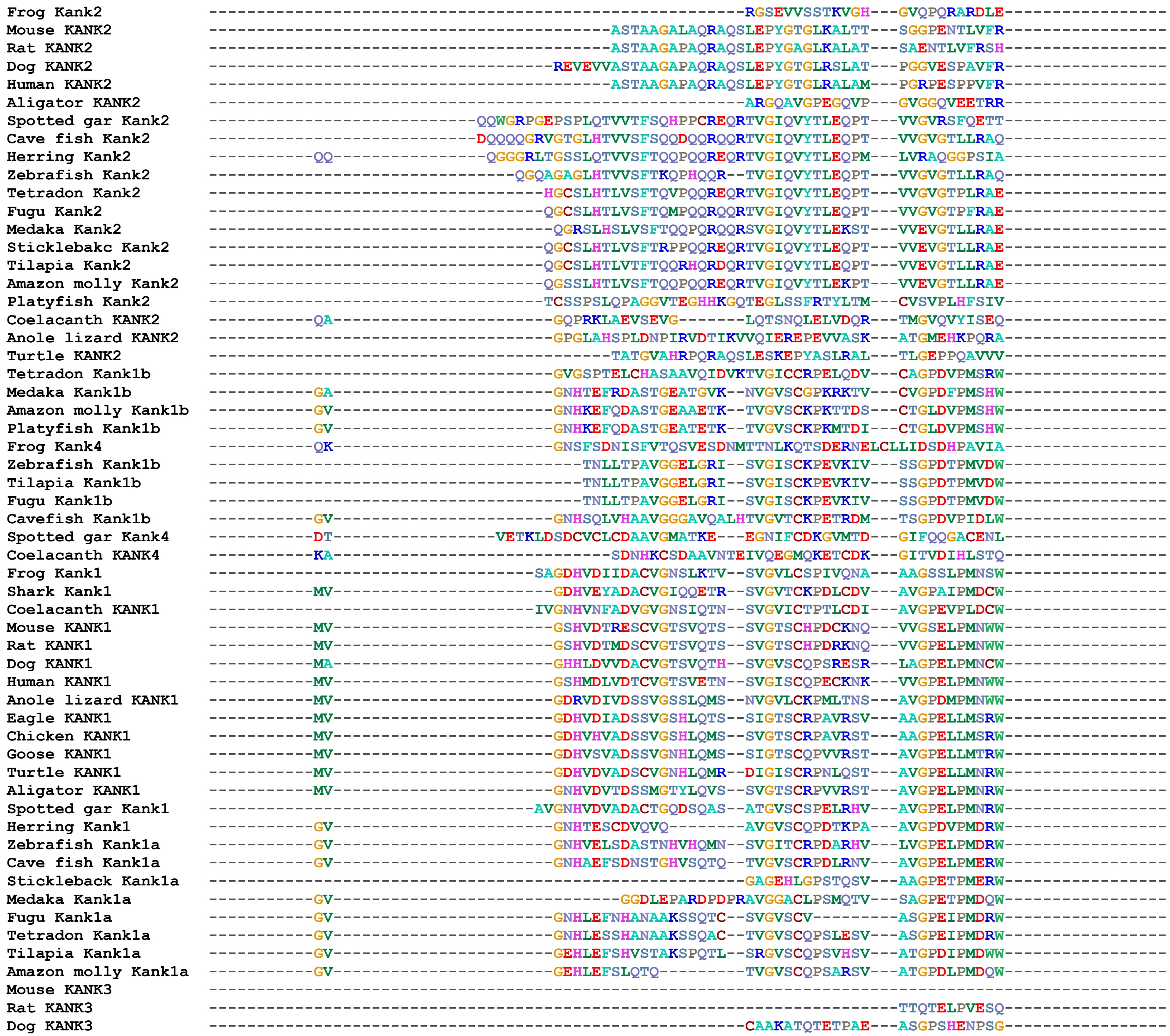

Frog Kank2 RGSEVVSSTKVGH---GVQPQRARDLE
Mouse KANK2 ASTAAGALAQRAQSLEPYGTGLKALTT---SGGPENTLVFR
Rat KANK2 ASTAAGAPAQRAQSLEPYGAGLKALAT---SAENTLVFRSH
Dog KANK2 REVEVVASTAAGAPAQRAQSLEPYGTGLRSLAT---PGGVESPAVFR
Human KANK2 ASTAAGAPAQRAQSLEPYGTGLRALAM---PGRPESPPVFR
Aligator KANK2 ARGQAVGPEGQVP---GVGGQVEETRR
Spotted gar Kank2 QQWGRPGEPSPLQTVVTFSQHPPCREQRTVGIQVYTLEQPT---VVGVRSFQETT
Cave fish Kank2 DQQQQGRVGTGLHTVVSFSQQDQQRQQRTVGIQVYTLEQPT---VVGVGTLLRAQ
Herring Kank2 QQ QGGGRLTGSSLQTVVSFTQQPQQREQRTVGIQVYTLEQPM---LVRAQGGPSIA
Zebrafish Kank2 QGQAGAGLHTVVSFTKQPHQQR--TVGIQVYTLEQPT---VVGVGTLLRAQ
Tetradon Kank2 HGCSLHTLVSFTQVPQQREQRTVGIQVYTLEQPT---VVGVGTPLRAE
Fugu Kank2 QGCSLHTLVSFTQMPQQRQQRTVGIQVYTLEQPT---VVGVGTPFRAE
Medaka Kank2 QGRSLHSLVSFTQQPQRQQRSVGIQVYTLEKST---VVEVGTLLRAE
Sticklebakc Kank2 QGCSLHTLVSFTRPPQQREQRTVGIQVYTLEQPT---VVEVGTLLRAE
Tilapia Kank2 QGCSLHTLVTFTQQRHQRDQRTVGIQVYTLEQPT---VVEVGTLLRAE
Amazon molly Kank2 QGSSLHTLVSFTQQPQQREQRTVGIQVYTLEKPT---VVEVGTLLRAE
Platyfish Kank2 TCSSPSLQPAGGVTEGHHKGQTEGLSSFRTYLTM---CVSVPLHFSIV
Coelacanth KANK2 QA GQPRKLAEVSEVG LQTSNQLELVDQR---TMGVQVYISEQ
Anole lizard KANK2 GPGLAHSPLDNPIRVDTIKVVQIEREPEVVASK---ATGMEHKPQRA
Turtle KANK2 TATGVAHRPQRAQSLESKEPYASLRAL---TLGEPPQAVVV
Tetradon Kank1b GVGSPTELCHASAAVQIDVKTVGICCRPELQDV---CAGPDVPMSRW
Medaka Kank1b GA GNHTEFRDASTGEATGVK--NVGVSCGPKRKTV---CVGPDFPMSHW
Amazon molly Kank1b GV GNHKEFQDASTGEAAETK--TVGVSCKPKTTDS---CTGLDVPMSHW
Platyfish Kank1b GV GNHKEFQDASTGEATETK--TVGVSCKPKMTDI---CTGLDVPMSHW
Frog Kank4 QK GNSFSDNISFVTQSVESDNMTTNLKQTSDERNELCLLIDSDHPAVIA
Zebrafish Kank1b TNLLTPAVGGELGRI--SVGISCKPEVKIV---SSGPDTPMVDW
Tilapia Kank1b TNLLTPAVGGELGRI--SVGISCKPEVKIV---SSGPDTPMVDW
Fugu Kank1b TNLLTPAVGGELGRI--SVGISCKPEVKIV---SSGPDTPMVDW
Cavefish Kank1b GV GNHSQLVHAAVGGGAVQALHTVGVTCKPETRDM---TSGPDVPIDLW
Spotted gar Kank4 DT VETKLDSDCVCLCDAAVGMATKE---EGNIFCDKGVMTD---GIFQQGACENL
Coelacanth KANK4 KA SDNHKCSDAAVNTEIVQEGMQKETCDK---GITVDIHLSTQ
Frog Kank1 SAGDHVDIIDACVGNSLKTV--SVGVLCSPIVQNA---AAGSSLPMNSW
Shark Kank1 MV GDHVEYADACVGIQQETR--SVGVTCKPDLCDV---AVGPAIPMDCW
Coelacanth KANK1 IVGNHVNFADVGVGNSIQTN--SVGVICTPTLCDI---AVGPEVPLDCW
Mouse KANK1 MV GSHVDTRESCVGTSVQTS--SVGTSCHPDCKNQ---VVGSELPMNWW
Rat KANK1 MV GSHVDTMDSCVGTSVQTS--SVGTSCHPDRKNQ---VVGPELPMNWW
Dog KANK1 MA GHHLDVVDACVGTSVQTH--SVGVSCQPSRESR---LAGPELPMNCW
Human KANK1 MV GSHMDLVDTCVGTSVETN--SVGISCQPECKNK---VVGPELPMNWW
Anole lizard KANK1 MV GDRVDIVDSSVGSSLQMS--NVGVLCKPMLTNS---AVGPDMPMNWW
Eagle KANK1 MV GDHVDIADSSVGSHLQTS--SIGTSCRPAVRSV---AAGPELLMSRW
Chicken KANK1 MV GDHVHVADSSVGSHLQMS--SVGTSCRPAVRST---AAGPELLMSRW
Goose KANK1 MV GDHVSVADSSVGNHLQMS--SIGTSCQPVVRST---AVGPELLMTRW
Turtle KANK1 MV GDHVDVADSCVGNHLQMR--DIGISCRPNLQST---AVGPELLMNRW
Aligator KANK1 MV GNHVDVTDSSMGTYLQVS--SVGTSCRPVVRST---AVGPELPMNRW
Spotted gar Kank1 AVGNHVDVADACTGQDSQAS--ATGVSCSPELRHV---AVGPELPMNRW
Herring Kank1 GV GNHTESCDVQVQ AVGVSCQPDTKPA---AVGPDVPMDRW
Zebrafish Kank1a GV GNHVELSDASTNHVHQMN--SVGITCRPDARHV---LVGPELPMDRW
Cave fish Kank1a GV GNHAEFSDNSTGHVSQTQ--TVGVSCRPDLRNV---AVGPELPMERW
Stickleback Kank1a GAGEHLGPSTQSV---AAGPETPMERW
Medaka Kank1a GV GGDLEPARDPDPRAVGGACLPSMQTV---SAGPETPMDQW
Fugu Kank1a GV GNHLEFNHANAAKSSQTC--SVGVSCV ASGPEIPMDRW
Tetradon Kank1a GV GNHLESSHANAAKSSQAC--TVGVSCQPSLESV---ASGPEIPMDRW
Tilapia Kank1a GV GEHLEFSHVSTAKSPQTL--SRGVSCQPSVHSV---ATGPDIPMDWW
Amazon molly Kank1a GV GEHLEFSLQTQ TVGVSCQPSARSV---ATGPDLPMDQW
Mouse KANK3
Rat KANK3 TTQTELPVESQ
Dog KANK3 CAAKATQTETPAE---ASGPSHENPSG

```
Human KANK3            ------------------------------------------------------------------------AEAPSLTQESS-----------------
Lamprey                --------------------------------------------------------RLLTTVTALSSLQ---RPAPTPPATQA-----------------
Coelacanth KANK3       -----------SV-----------------------GNHLEMTETGVQCMPQMT--SIGIGCRSEVKEV---AVGPESTQVTE-----------------
Frog Kank3             ----------------------------------ASGEHLKMVDAGVNCCSELS--CIAVSCVSEAKEV---AVGTDQMLGYQ-----------------
Turtle KANK3           -----------GV-----------------------GSHLEMIDKGVDCSPEML--CIGVNCNLESREV---AVGPDSPALYE-----------------
Aligator KANK3         -----------AV-----------------------GSPLEMIDEGVDCSPHML--CIGINYDLKGKEV---AVGPDSAATCE-----------------
Eagle KANK3            -----------AA-----------------------GDPLETAEAGVECCPPTA--CVGVGCRPDGRDV---AVGPDSAGRCK-----------------
Chicken KANK3          ------------------------------------------------------------GCRQPPPGS---PAAPTPQRAAV-----------------
Goose KANK3            ----------------------------------------------------------------------------------------------------
Spotted gar Kank3      ------------------------------------------------------------------------AAGGTLEAPEH-----------------
Zebrafish Kank3        ---------------------------------------------------------------GVLFPD---VTDPEYDLKKD-----------------
Cavefish Kank3         -------------------------------------------------------------VKPETTDA---LVSTEVCLTSS-----------------
Herring Kank3          -----------------------------------------------------MLHSVGVSCRPEVAEA---GVGGEQAPAPS-----------------
Stickleback Kank3      ------------------------------------------------------------------------KVSPEEEVVSM-----------------
Fugu Kank3             ---------------------------------------------------------ASVLCRSEVSDA---CVGGSLTGNQI-----------------
Tetradon Kank3         -------------------------------------------------------------AQPDLVDA---QVETETPPL-------------------
Medaka Kank3           ---------------------------------------------------------AAIQCRPEVTDI---CVGKETDQGEQ-----------------
Tilapia Kank3          -------------------------------------------------------------AKPDMTNA---QVETLTSALKA-----------------
Amazon molly Kank3     ---------------------------------------------------------TATTCHPEVTHA---CVGEDLGADHT-----------------
Platyfish Kank3        -----------TA----------------------------------------------TMCRPEVTHV---CVGEDLGTDQT-----------------

                                1810      1820      1830      1840      1850      1860      1870      1880      1890      1900
                       ....|....|....|....|....|....|....|....|....|....|....|....|....|....|....|....|....|....|....|....|
Hydra                  ----------------------------------------------------------------------------------------------------
Trichoplax             ----------------------------------------------------------------------------------------------------
Aplasia                KIATYSTGVATDVKQMYTWCQTTEKVTDLFRKEEEQKTVITTTEVKSVPKKVLMVDIGVGDGLVEDPYMFSETVEIIEPVTTTLTTEEKIVQRGASQANA
Snail                  -------------------------------------------------MSSAQIKHAVTNTEQPTTYSTAMCTDLVIGLDSATNTESIPRSNVAVNTTE
Fruitfly               ----------------------------------------------------------------------------------------------------
Hookworm               ----------------------------------------------------------------------------------------------------
C.elegance             ----------------------------------------------------------------------------------------------------
Tunicate               ----------------------------------------------------------------------------------------------------
Spider                 ----------------------------------------------------------------------------------------------------
Lingula                EKVQTRSKGVGDGRAMVRENTEGQVVQQQQQVKEVSKQQSYQPQQQPTPKSSQDRVVTETITTYTSQVPSVVRRVKNTHLDKETLETQRRSLLGLDDAAT
Lancelet               KTAPGGGKGRRLSPSTAKKPKVPPPVPPRSFRSVSVGDTTSLSDIVCYNSALRTTTDVGTNTTVPSRDIGVGYSPRMRHVGVGISNDLLQQELGIV---E
Medaka Kank4b          ----------------------------------------------------------------------------------------------------
Amazon molly Kank4b    ----------------------------------------------------------------------------------------------------
Platyfish Kank4b       ----------------------------------------------------------------------------------------------------
Stickleback Kank4b     ----------------------------------------------------------------------------------------------------
Fugu Kank4b            ----------------------------------------------------------------------------------------------------
Tetraon Kank4b         ----------------------------------------------------------------------------------------------------
Cave fish Kank4b       ----------------------------------------------------------------------------------------------------
Medaka Kank4a          ----------------------------------------------------------------------------------------------------
Fugu Kank4a            ----------------------------------------------------------------------------------------------------
Tetradon Kank4a        ----------------------------------------------------------------------------------------------------
Stickleback Kank4a     ----------------------------------------------------------------------------------------------------
Tilapia Kank4a         ----------------------------------------------------------------------------------------------------
Platyfish Kank4a       ---------------------------------------------VTDVKGIVIQTLDSEVGKKTKVCPSPTSGTDKREVTSAQDHSKPKELQLSTGIEI
Amazon Molly Kank4a    ----------------------------------------------------------SEVGNKTKVCPSPASGTDKREATSAQDDSKPKELQLSTGVEI
Herring Kank4          --------------------------------------------------------SSTQTTSVEAKDMEVLAQVLTSEKEVGVEIITCDQAVETEPQMD
Zebradish Kank4        ----------------------------------------------------------------------------------------------------
Cave fish Kank4a       ----------------------------------------------------------------------------------------------------
Mouse KANK4            ----------------------------------------------------------------------------------------------------
Rat KANK4              ----------------------------------------------------------------------------------------------------
Dog KANK4              ----------------------------------------------------------------------------------------------------
Human KANK4            ----------------------------------------------------------------------------------------------------
Eagle KANK4            ----------------------------------------------------------------------------------------------------
Chicken KANK4          ----------------------------------------------------------------------------------------------------
```

```
Goose KANK4              ----------------------------------------------------------------------------------------------------
Anole lizard KANK4       ----------------------------------------------------------------------------------------------------
Turtle KANK4             ----------------------------------------------------------------------------------------------------
Aligator KANK4           ----------------------------------------------------------------------------------------------------
Frog Kank2               ----------------------------------------------------------------------------------------------------
Mouse KANK2              ----------------------------------------------------------------------------------------------------
Rat KANK2                ----------------------------------------------------------------------------------------------------
Dog KANK2                ----------------------------------------------------------------------------------------------------
Human KANK2              ----------------------------------------------------------------------------------------------------
Aligator KANK2           ----------------------------------------------------------------------------------------------------
Spotted gar Kank2        ----------------------------------------------------------------------------------------------------
Cave fish Kank2          ----------------------------------------------------------------------------------------------------
Herring Kank2            ----------------------------------------------------------------------------------------------------
Zebrafish Kank2          ----------------------------------------------------------------------------------------------------
Tetradon Kank2           ----------------------------------------------------------------------------------------------------
Fugu Kank2               ----------------------------------------------------------------------------------------------------
Medaka Kank2             ----------------------------------------------------------------------------------------------------
Sticklebakc Kank2        ----------------------------------------------------------------------------------------------------
Tilapia Kank2            ----------------------------------------------------------------------------------------------------
Amazon molly Kank2       ----------------------------------------------------------------------------------------------------
Platyfish Kank2          ----------------------------------------------------------------------------------------------------
Coelacanth KANK2         ----------------------------------------------------------------------------------------------------
Anole lizard KANK2       ----------------------------------------------------------------------------------------------------
Turtle KANK2             ----------------------------------------------------------------------------------------------------
Tetradon Kank1b          -----------------------------------------------EVRERVEVMEKNVGNDIL-----------MITRAVGIETKSRDAETNTEIPVE
Medaka Kank1b            ---------------------------------------------------------------------EIRERVENKDAAVGIQIFTNTLGVGTEMKVR
Amazon molly Kank1b      ---------------------------------------------------------------------EIRERVETSDKAVGIQVFTSTVGVGTEIKLS
Platyfish Kank1b         ---------------------------------------------------------------------EIRERVETSEKAIGIQVSTSTVGVGTDIKLS
Frog Kank4               ----------------------------------------------------------------------------------------------------
Zebrafish Kank1b         -----------------------------------------------EVRRRVERKDQCVGSKCA----------KTQSQCVGTTVCMCDATTITLETMK
Tilapia Kank1b           -----------------------------------------------EVRRRVERKDQCVGSKCA----------KTQSQCVGTTVCMCDATTITLETMK
Fugu Kank1b              -----------------------------------------------EVRRRVERKDQCVGSKCA----------KTQSQCVGTTVCMCDATTITLETMK
Cavefish Kank1b          -----------------------------------------------EVRKKVQRRDQCVGKDYVP----------TCSQCVGTAVIVRDTGMVTLELME
Spotted gar Kank4        ----------------------------------------------------------------------------------------------------
Coelacanth KANK4         ----------------------------------------------------------------------------------------------------
Frog Kank1               -----------------------------------------------IVKQKVEMCDTGTGRDLD-----------LCDKCVGNDQVLFEDALNIREPID
Shark Kank1              -----------------------------------------------VVHEKVEVQEKNVGGHVV-----------MCDKTVTCGVNVCFSDMGVN---T
Coelacanth KANK1         -----------------------------------------------VVHEKTEMQDKCIGQHIE-----------VCNKNVGVELCTCEVGINTEESIE
Mouse KANK1              -----------------------------------------------VVKERVAMHDQCVGRSVE-----------TCDRSVGVEVSVCETGSNTEASGS
Rat KANK1                -----------------------------------------------VVKERVGTHDRCVGRSVE-----------TCNRSVGVEVSVCETGSNTEASGS
Dog KANK1                -----------------------------------------------IVKERVDVRDQCTSRSVE-----------MCDKSVGVETSVCETGSNTEESVN
Human KANK1              -----------------------------------------------IVKERVEMHDRCAGRSVE-----------MCDKSVSVEVSVCETGSNTEESVN
Anole lizard KANK1       -----------------------------------------------IVKDREDMRDQCIGSSVE-----------TCNQCVGTEISICETGVNTEESVD
Eagle KANK1              -----------------------------------------------LVRERAEVRDQGAGRSVE-----------LRNKAVGAETSVCEMGINTEEPAG
Chicken KANK1            -----------------------------------------------LVRERAEMRDQCTGRCVE-----------LLDKKVGVETSVCETGVNTEESVG
Goose KANK1              -----------------------------------------------LVRERAEVRDQCTGRCVE-----------LYDKQVGVETSVCETGVNTEESVG
Turtle KANK1             -----------------------------------------------LVKERADMRDQCTGRSTE-----------MCDKNVGTEMSVCETGINTEESVD
Aligator KANK1           -----------------------------------------------LVKERPELCDQCTGRSIE-----------KHDKCVGIEMSVCETGINTEESVD
Spotted gar Kank1        -----------------------------------------------TVQERVQTQDKCTGRCVE-----------MCSKNVGVELSVYETGVNTEEIVG
Herring Kank1            -----------------------------------------------VVQERKEVMDQCVGRQVT-----------MSDKSVGLQLSMCEMGINTEETVE
Zebrafish Kank1a         -----------------------------------------------IVQERTEVQDQCVGRHVV-----------MCSQSVGVELSVCEMGINTELTAE
Cave fish Kank1a         -----------------------------------------------IVQERVEVQDQCVGKQVI-----------LCHQKVGVELSTCEMGVNTELTVE
Stickleback Kank1a       -----------------------------------------------VVGVRVEVRDQCVGREAG--------------------PSVREAGVNTEESVD
Medaka Kank1a            -----------------------------------------------VVRRRPPVHDKCVGREVE-----------TCHQLVGAEQSVCEVGVNTEADPG
Fugu Kank1a              -----------------------------------------------SVHELVEVKDQCVGRRME-----------TRDQQVGVEQNVCEVGVNTEESMD
Tetradon Kank1a          -----------------------------------------------VVHELVEVKDQCVGRRVE-----------TCDQLVGVEVNACEAGVNTEESMG
Tilapia Kank1a           -----------------------------------------------VVHEQMEVSDKCVGRHVE-----------TCHQQVGVELSVCEVGVNTEESVD
```

```
Amazon molly Kank1a   -----------------------------------------------VVHQRVEVRDQCVGRRVE-----------TRHQQVGMEPNISEAGVNTEESLD
Mouse KANK3           ----------------------------------------------------------------------------------------------------
Rat KANK3             ----------------------------------------------------------------------------------------------------
Dog KANK3             ----------------------------------------------------------------------------------------------------
Human KANK3           ----------------------------------------------------------------------------------------------------
Lamprey               ----------------------------------------------------------------------------------------------------
Coelacanth KANK3      ----------------------------------------------------------------------------------------------------
Frog Kank3            ----------------------------------------------------------------------------------------------------
Turtle KANK3          ----------------------------------------------------------------------------------------------------
Aligator KANK3        ----------------------------------------------------------------------------------------------------
Eagle KANK3           ----------------------------------------------------------------------------------------------------
Chicken KANK3         ----------------------------------------------------------------------------------------------------
Goose KANK3           ----------------------------------------------------------------------------------------------------
Spotted gar Kank3     ----------------------------------------------------------------------------------------------------
Zebrafish Kank3       ----------------------------------------------------------------------------------------------------
Cavefish Kank3        ----------------------------------------------------------------------------------------------------
Herring Kank3         ----------------------------------------------------------------------------------------------------
Stickleback Kank3     ----------------------------------------------------------------------------------------------------
Fugu Kank3            ----------------------------------------------------------------------------------------------------
Tetradon Kank3        ----------------------------------------------------------------------------------------------------
Medaka Kank3          ----------------------------------------------------------------------------------------------------
Tilapia Kank3         ----------------------------------------------------------------------------------------------------
Amazon molly Kank3    ----------------------------------------------------------------------------------------------------
Platyfish Kank3       ----------------------------------------------------------------------------------------------------

                               1910      1920      1930      1940      1950      1960      1970      1980      1990      2000
                      ....|....|....|....|....|....|....|....|....|....|....|....|....|....|....|....|....|....|....|....|
Hydra                 ----------------------------------------------------------------------------------------------------
Trichoplax            ----------------------------------------------------------------------------------------------------
Aplasia               FLAQSGSSSSSTQQRTSSSDDGSRYSTYEVSLRSGSTAPSSSYSTSSTQEKVSSYGASTASRSSVTDQTSTSQVSTPTKVGEEGFSETVVEHYIITDEGK
Snail                 KVSQLFQQATVEKKAPELKSILKKPQHVDKSVGDNVADEEPYSFSEITEVITPITIVSTAKLENVQKAATETNKYISQSNSVGRSGSDERDVHISSYELS
Fruitfly              ------------KPAPQMPGRSNTFSLGENETVSVVRKTIGCQTPVTLVLS------------------------------------------------S
Hookworm              ----------------------------------------------------------------------------------------------------
C.elegance            ----------------------------------------------------------------------------------------------------
Tunicate              ---------------------DQCIGTQPTSTSDVAVSCELPTHPTVEVRTLATNTDAGNGRWCVYNDMK------------------------DVACGN
Spider                ----------------------------------------------------------------------------------------------------
Lingula               QQLQDTSLRDLVSSSVTRSYKSKATGGQEKSDHMSGLHIGELTKGKKMPEDQTETFEEWVEITPVYQERVIEARRKGGEMTSSTSERTRDFSSSSSSSSA
Lancelet              NQKCNTAVVETSEVGVGVSVEQRAVAVGNHTELRDVGMTTEPEVVKKQKTKHATRSIGVIVDIPETKDVR-----------------TVGVGNGSVLEQQ
Medaka Kank4b         ----------------------------------------------------------------------------------------------------
Amazon molly Kank4b   ----------------------------------------------------------------------------------------------------
Platyfish Kank4b      ----------------------------------------------------------------------------------------------------
Stickleback Kank4b    ----------------------------------------------------------------------------------------------------
Fugu Kank4b           ----------------------------------------------------------------------------------------------------
Tetraon Kank4b        ----------------------------------------------------------------------------------------------------
Cave fish Kank4b      ----------------------------------------------------------------------------------------------------
Medaka Kank4a         ----------------------------------------------------------------------------------------------------
Fugu Kank4a           ----------------------------------------------------------------------------------------------------
Tetradon Kank4a       ----------------------------------------------------------------------------------------------------
Stickleback Kank4a    ----------------------------------------------------------------------------------------------------
Tilapia Kank4a        ----------------------------------------------------------------------------------------------------
Platyfish Kank4a      VEPKTTEMETAESKDKEMLCESVKENIIPDTVVVIAENSAVKSVAPIRPQRGRKPPAEQMQPTPPQPQLAPMLTTRESSESESEKITTKVQTAPGLEADL
Amazon Molly Kank4a   IEPKKIEMETAENKDKEIVCESVKENVVPDTVIVIAENSAGKAVAPVRPQRGRKPSAEQMQPTPPQPQLAPMLTTRESSETESDKITTKVQTAPRLEAEL
Herring Kank4         MVSGTEDRNEGLEEVVEQENVLFEAVDLESTSSENVVSEVKVLEEKAVEDTVGESLEAG------------------------------------SETEE
Zebradish Kank4       ---------------------TNIVEARDIEVLAQVTTAEKIVGVEVVMCD--------------------------------------QSIETDVLDNL
Cave fish Kank4a      -------------DLISSSTQTTIIQTRDIEVLAQVMTAEKVVGVEVSTSD-------------------------------------------------
Mouse KANK4           ----------------------------------------------------------------------------------------------------
Rat KANK4             ----------------------------------------------------------------------------------------------------
```

```
Dog KANK4              ----------------------------------------------------------------------------------------------------
Human KANK4            ----------------------------------------------------------------------------------------------------
Eagle KANK4            -----------------------SVGCSIRRADNVNVQVMEVGARRDQ----------------------------------------------------
Chicken KANK4          -----------------------SIGCGIRRADNGNLLAAELSAQG------------------------------------------------------
Goose KANK4            -----------------------SIGCGVHRADNMNSRAVEVSAQGEQGWA-------------------------------------------------
Anole lizard KANK4     -----------------------SVECDVCEADNKNLQVKEMHRKLFHVPD------------------------------------------------E
Turtle KANK4           -----------------------SVGCGTYGVDNVNLQAKELRRVPVHTDQ-------------------------------------------------
Aligator KANK4         -----------------------SVGCGVDNMNLEVEKLSNM----------------------------------------------------------
Frog Kank2             ----------------------------------------------------------------------------------------------------
Mouse KANK2            ----------------------------------------------------------------------------------------------------
Rat KANK2              ----------------------------------------------------------------------------------------------------
Dog KANK2              ----------------------------------------------------------------------------------------------------
Human KANK2            ----------------------------------------------------------------------------------------------------
Aligator KANK2         ----------------------------------------------------------------------------------------------------
Spotted gar Kank2      -------------------------TTPRKDQAEFVQQPSWGEGGGTHAEL-------------------------------------TGLVTKDPNGQQ
Cave fish Kank2        -------------------------ECSSPTRPLSAVLTEGGAHRGHAEPL--------------------------------------------SGEDS
Herring Kank2          --SAPSGTVLEGPLRGQAEALSPEEGPRELPIAISSKQVREVLLKSEVSSSVPVTSPVAVMETKCHQVTP------------------------LNQGSN
Zebrafish Kank2        -----------------------------------------------------------------------------------------------GCTHP
Tetradon Kank2         ----------------------------------------------------------------------------------------------------
Fugu Kank2             ----------------------------------------------------------------------------------------------------
Medaka Kank2           ----------------------------------------------------------------------------------------------------
Sticklebakc Kank2      ----------------------------------------------------------------------------------------------------
Tilapia Kank2          ----------------------------------------------------------------------------------------------------
Amazon molly Kank2     ----------------------------------------------------------------------------------------------------
Platyfish Kank2        ----------------------------------------------------------------------------------------------------
Coelacanth KANK2       ----------------------PTSGTKDYKEVPVKVDQLELIQTQKETKG-------------------------------------------------
Anole lizard KANK2     ----------------------------------------------------------------------------------------------------
Turtle KANK2           ----------------------------------------------------------------------------------------------------
Tetradon Kank1b        DLAPRK------------EKKYSSVACGDCSVHLAVPEGKS----------------------------------------------------QTVSTST
Medaka Kank1b          DAGSNTEMPAENNWFKKEKVKCLSVGCGDFSINDSAVKDMVSKSVTTDL---IRGVDLGIMASPDTSSQR------------------TNTVSNLVSRFT
Amazon molly Kank1b    DAESNTDLPLEKLVFQKEKVKCHSVACGAYSVGLTICESKEMISQGITTDS-IRRVDQGILASPQMASQR------------------TNTVSSSVSRFT
Platyfish Kank1b       DAESNTELPLEKLVFQKEKVKCHSVACGAYSVDLTICESKEMTSQGITTDS-IRRVDQGILASPQTASQR-----------------TNTVSSSVSRFTN
Frog Kank4             ----------------------------------------------------------------------------------------------------
Zebrafish Kank1b       NRR---------------NISYRTVGCGDYMVNADLRPIKSFVSQGTVTDP-IRSVDLGVMVIPQTVSQR-----------------TSTAFCTISRSTS
Tilapia Kank1b         NRR---------------NISYRTVGCGDYMVNADLRPIKSFVSQGTVTDP-IRSVDLGVMVIPQTVSQR-----------------TSTAFCTISRSTS
Fugu Kank1b            NRR---------------NISYRTVGCGDYMVNADLRPIKSFVSQGTVTDP-IRSVDLGVMVIPQTVSQR-----------------TSTAFCTISRSTS
Cavefish Kank1b        TLSKRK-------------VSSRTVGCGDCTIDMNVNVVKPLVSKGMVTDP-VKGIDLGITVNPQTMSQR-----------------TNTTISTVSRCTG
Spotted gar Kank4      ----------------------------------------------------------------------------------------------------
Coelacanth KANK4       -----------------------SVGCGDCTVDI-FSPVKEVHSMGVQTDC-------------------------------------------------
Frog Kank1             QQDGTGDLKT---------KELRSVGCGDSSVDVIVKPVMESLSCGVNTEF-VNKVDSSVTASPCTADQQ------------------TNTEMDTANQYT
Shark Kank1            DETVEDPDISGGPHSALRAKDVQSVGCGDCLVDVVVFPAKELASSSTNTVA-VSTAELAVMAAPQTTTQQ------------------THTQIDTSCKFT
Coelacanth KANK1       NLELFKRKAE--------PKESRSVGCGDCLVNVIVCLPKELVSCSTNTDI-LSKLDSAVMAVPQTTAKE------------------SNTVLETTNQFT
Mouse KANK1            DLTLLKTNLN--------LKDVRSIGCGDCSVDVIVCFPKECTSRSMNTEA-VGQEEAAVMAVPHTTDQH------------------TSTNLERVDQCT
Rat KANK1              DLTLLKTNLN--------LKDVRSIGCGDCSVDVIVCLPKECTSRSMNTEA-VGQGEAAVMAVPHTTDQH------------------TSTNLERVDQGT
Dog KANK1              DLSLLKTNLN--------LKEVRSIGCGDCSVDVTVCPPKECTSRSVNTEA-ISQVDAAVTAVPQTASQH------------------TSTVLGQVNQAT
Human KANK1            DLTLLKTNLN--------LKEVRSIGCGDCSVDVTVCSPKECASRGVNTEA-VSQVEAAVMAVPRTADQD------------------TSTDLEQVHQFT
Anole lizard KANK1     RLSLCKTELK--------IKEVRSIGCGAC------FPVNETISCSTNTET-SCKSDSGVMAVPCTATQG------------------TNTVLEKTDQLT
Eagle KANK1            VPSPRQTARS--------VGAVRSVGCGDCSVDVMVCTPKERVSRETATEA-VPRVEATVMAVPSTASRQ------------------TSTALEMVSQCT
Chicken KANK1          DLSLCRTARE--------VRETRSIGCGDCSVDVVVCAPKECVSRQTATET-VCRAEATVMAVPSTATQQ------------------TSTAMEMVSRCT
Goose KANK1            NLSLCRTAQE--------VRETRTVGCGDCSVDVIVHSPKECVSRETATEA-VSRAEATVMAVPSTASQH------------------TSTALETVSRCT
Turtle KANK1           NLSLCKTEAE--------IKEVRSIGCGDCSVNVTVCPPKECVSRSTNTEV-VCKVECAVMAVPQTATQQ------------------TSTVLAMVNQFT
Aligator KANK1         SLSLCKTEQE--------IKEVRSIGCGDCSVNVIVCPPKECMSRDTNTEA-ICKVEATVMAVPHTATQQ------------------TGTVLEVVNQFT
Spotted gar Kank1      NLDLYKTRLE-------MAKKFRSVGCGECSVNVTVCPEKDLVSFGTNTDF-VNMVESGIMAVRHTTSQY------------------TNTAVDTVSTST
Herring Kank1          SLGLCKAEAE-------QAKEVRSIGCGDCSVNVTVSPIKDLVSLGTVTDA-VSREDQGVMVFPETLSQY------------------CETDMLGVSRST
Zebrafish Kank1a       GLGLCKREAE-------QAKEFRSIGCGDCSVDVMVRPIKEMATRSTGTDE-VHRTDFGVMVLPICASQY------------------TNIEIETIDKLT
Cave fish Kank1a       NLGLCKTEPE-------QAKEYRSIGCGDCSVDVLVSPIKELFSQGTATDL-VRKLDSAVMVSPTMHCQH-----------------TNTEVKTSVGRCT
Stickleback Kank1a     RLALCRPMAE-------LSRESRSVGCGDCSVDVVVRPAKTLLSRGTDPER-VGTADSAVTAAPESATRQ------------------TNTETEVASKST
```

```
Medaka Kank1a            GRT---------------WAESRSVGCGDGSVEV---PAKTFVSRGTEADA-VRRTDSAVMAAPLSATQQ------------------TNTETQVAAKST
Fugu Kank1a              SLVLRKAKLS---------KESRSVGCGDCSVDVIVSPVKALVSRGTDPDL-IGKVDLGVMAAPESANQQ------------------TSTDLEVASKST
Tetradon Kank1a          SLALSKAKFS---------KESRSVGCGDCSVDVTVGPIKVLGSRGTNTDL-ISKVDSSVTAAPESANQQ------------------TNTDLEVVNKST
Tilapia Kank1a           SLTLSKPAVE-------ASKEQRSVGCGDCSVDVIVSPIKALVSQGTETDR-VSQVDSGVTAAPELLTQE------------------TSTEAEVAHKYT
Amazon molly Kank1a      GPGFCRTAAE-------PIKQSRSVGCGDCSVDVNVTPVKTFVSRGTDPDV-VGTIDSAVMAAPDSASQQ------------------TGTDTEYESRST
Mouse KANK3              ----------------------------------------------------------------------------------------------------
Rat KANK3                ----------------------------------------------------------------------------------------------------
Dog KANK3                ----------------------------------------------------------------------------------------------------
Human KANK3              ----------------------------------------------------------------------------------------------------
Lamprey                  ----------------------------------------------------------------------------------------------------
Coelacanth KANK3         ----------------------------------------------------------------------------------------------------
Frog Kank3               ----------------------------------------------------------------------------------------------------
Turtle KANK3             ----------------------------------------------------------------------------------------------------
Aligator KANK3           ----------------------------------------------------------------------------------------------------
Eagle KANK3              ----------------------------------------------------------------------------------------------------
Chicken KANK3            ----------------------------------------------------------------------------------------------------
Goose KANK3              ----------------------------------------------------------------------------------------------------
Spotted gar Kank3        ----------------------------------------------------------------------------------------------------
Zebrafish Kank3          ----------------------------------------------------------------------------------------------------
Cavefish Kank3           ----------------------------------------------------------------------------------------------------
Herring Kank3            ----------------------------------------------------------------------------------------------------
Stickleback Kank3        ----------------------------------------------------------------------------------------------------
Fugu Kank3               ----------------------------------------------------------------------------------------------------
Tetradon Kank3           ----------------------------------------------------------------------------------------------------
Medaka Kank3             ----------------------------------------------------------------------------------------------------
Tilapia Kank3            ----------------------------------------------------------------------------------------------------
Amazon molly Kank3       ----------------------------------------------------------------------------------------------------
Platyfish Kank3          ----------------------------------------------------------------------------------------------------

                                  2010      2020      2030      2040      2050      2060      2070      2080      2090      2100
                         ....|....|....|....|....|....|....|....|....|....|....|....|....|....|....|....|....|....|....|....|
Hydra                    ---------------------CDKCNNLKTLSCGVGDCTIDNLLCDRCSNKTFFD-IGVSED---SVNYSCCTSCTQKSVRSIGI-GEGNINDTF-----
Trichoplax               ---------------------RNGHADNAAKTISIADIQEENLNTD----------------------------------------GEKDSQNKY-----
Aplasia                  KLISKERTRSTTSGGTNRTVQQFSDGNVVGGGEGGGGGSSGTDGMWSSSLSQSQS-SSSSSYDSRSYNGSAGGGSLTYGSVSGGVSGGVTGTAGDAGGYQ
Snail                    RRSSAPVTLVGKQSESLETNTMTMNSSFKQVSPRRSDDSFQETVTEHYIITKDGK-KLISEEKTTTSSRGGHSSSVNHYTGNASIEGRSSLTDAS-----
Fruitfly                 GSQTTTAVVRSTGAQVNPQQQHSGVQCATEQVSRQTDTKGLQRLTRSQTKAEQVS-ESVPRPTTRHTASNTDKVEEAEPAKVRTSHSACNTEEVR-----
Hookworm                 ------------------KGDDLEYIAEKNKTVSTSPSAPATTPS----GETADA-LKSKVVLKKEVPPPPSNLPDPIPARI------------------
C.elegance               -------------AAAADCEECLKRQDVFTRNVGVGACSISDKICVSCDNQVVEA-------DENEAPGFKTDTKDDQKQRDFEI-AKANAVKKL-----
Tunicate                 ERVCIPNASVAVGTELQTCDQCTDTHVATRTTSTIADIRKPSTDA------VQQT-ADVATYNSSTDCDDIIPIVTSSTTNTQCV-TTQNVSTQY-----
Spider                   ----------------REVEKAFNQNQILKHSVAVGDFNILDTIC----ALCLKA-KTSVGVGDSDINISFCDRCRSVKTVSTSC-GEASIYDVC-----
Lingula                  TGMTVSLDSTDLGPKQEIIPHHTIYGGKRIIDSSVGQGYKPSVATYTSMRSANRSPSPTETSYRITESRERSGGGTVTGTVEGGDSGVTHTTETY-----
Lancelet                 KPPVETASVSTNTPVVNTIDKVTNTVAVNLKTVAVGNFSVKDVLCDRCADVKTKT-VAVGVC---SIKDTICDRCNNMESKTVGV-GRCTINDTV-----
Medaka Kank4b            -----------------LALRHPPQKMSSRGGVEPADGTADSMST-------------------------------------------------------
Amazon molly Kank4b      -----------------VITRTVPSQAQRHQDSVMSDGVTNTFII-------------------------------------------------------
Platyfish Kank4b         -----------------IISRTVPSHAQRHQDLIMSDGGTDTFII-------------------------------------------------------
Stickleback Kank4b       ----------------------------------------------------------------------------------------------------
Fugu Kank4b              -----------------------------GKSAGHGEGAGATVV--------------------------------------------------------
Tetraon Kank4b           ----------------------------------------------------------------------------------------------------
Cave fish Kank4b         -----------------VRAEGTEEHSPENKTSEHAKVRKPEA------SVQVQE-SGQCGLEEGLKPHHASKAPAKSHTE-------------------
Medaka Kank4a            SKVKLPKAQSKTDAQKPSSLSELPSRPKSIQTQSQSQASTSRRDSKEIESQQPQKGVGETQSLRRSSNEAAQRRGSSEAQALRRGSGEAQAARRG-----
Fugu Kank4a              -------------PPHEDTQKKHPAPAPAQKPPQRGESQARTTRQG---SCDTQT-QTQGSRKTSSEAKPAHKDGSDAQSLRRAS-GETAQRRGS-----
Tetradon Kank4a          ------------------PQRGKKPPAADQAEPSTPQQPAPVRPRRGSSEAQSQQ-AQPSTKGQPQVQTQGPPQLEGTQVPHPGP---------------
Stickleback Kank4a       --------SLAERPSAESSGKQTKPQPRSETQGAPPGASVKKRPK------SIQA-QTRRHSKELKASQPPGGVPGESQTRPTGQ-VSHEAQPSS-----
Tilapia Kank4a           -----------QQTETEMERRHDNNPARSVEAESQLEARSPEQLSETQTQTVSPG-SSEVQGSRRSSSEAQAPQKDASETQSLRR-GSSEAAQRR-----
Platyfish Kank4a         SAQPSEPRENDQMPSVPQVQDSSQAEKLPDKLIEKQYNVQPQGESQSVSAISSEI-QVKSKPIQPHSKSQPASRRDSKEQKAPQR-GSSVSHPQSRESGE
Amazon Molly Kank4a      SAQPSEPQENDQMPSVPQVQDSSQAEKLPDELIKKESNVQPQGESQSVSSSSSEI-QVKTKPIQPHTKSQHASRRDSKEQKAPQR-GSSVSHPPSRETGE
Herring Kank4            KKVLVERVLEENGVKESDVIQSAVTEPAPAESEAVANAKSEDVAKEPAVTKETVGSPSSSEPALKSPKEGQPGQEAQSQSRRSSSEGSGSGSASS-----
```

```
Zebradish Kank4       EGNNSLEVLEMLENTDSIKNKEQECDIKDSELSQITDTKVSENVAESDTEEYVMVESAMVETLASEIEVTEPTVQKTESAESLGIESTVTEKERE-----
Cave fish Kank4a      ---QAVETDVPISPTDNAQEDTDEQRDDARYSIDKEDTVEVQISESIMIESDVAEGYVIVEKTEGELVETVAEENTTVRLSTVEYKLPESVNVER-----
Mouse KANK4           --PECRAPRAEKNGFPWVQNNHKQSYPSPEEPVLPPQLSLPRGPE----QILASS-LCSCLSMELRIEEEGSEQEGGQEEGAGGL-SRAAGESSW-----
Rat KANK4             ------ASRAEKNGFPWVQTNHKQSYPSPEEPALPPQLSLPRGPEQILASSLCSC-LSMELRIEEEVSEQERDPDGGAGSLSREAGGSPWSRRES-----
Dog KANK4             -------ARGEGNGLLWGQAGHKPGDRSPPKLVPPPQLSLPQGPE----MVLPTS-LHSCLSTELRIEEAGSEQEGSPQVGAEGL-GRAAGGSSW-----
Human KANK4           -------HRGEENGLLWGPDGHKQGNQSPAERVLLPQLSLPQGPE----QVLTSS-VHSFLSTELRIEEAGTEQEGGPQGGTRGAGGFLWGSDRK-----
Eagle KANK4           ----GLADAGTGAGHFGEAESEAGLHASCFTQLKIDEHLSDDNQNH---RNNYDT-QSLAAHAVTAESYRGTRIGSNASENKAGF-GEDKPRGGL-----
Chicken KANK4         ---------------WADVGTSGGGDGHFAKLSETEAGLEPGLHAPGFTELKIDEQLSDDNQ---NQSYRTKRTGSSADENEAGF-GEDKTEDGM-----
Goose KANK4           ------GGGTEGPGHFAELTETEDDLEPGLHAPCLTELKIDEQFSDDNQNQRNNY-DTRSLTAHAATAASYRATKTDSNTGKTKA--------------
Anole lizard KANK4    MSNIPVCIQEKDSPSFQRQKEMKQDNDSSTLELSYNELKIDEHTA----NDKSEK-KAYNNVSRGESSLSVGTTDSTTGKNEHNFQGDIRQDSTE-----
Turtle KANK4          ------------GLAAVHTGVREEGAASLAKQKGTGIDTEPPVFTSCFTELKIDEQINDDCQHQRNSYDRQSCAASSLIGESCSRTGRADSNGNK-----
Aligator KANK4        ----PIHTDQGLADVHAGIRKKGTAFLDKQKRTGTDIEPAVHTSCFTELQINEHI-RDDCQSQRSSYDTESCVAPAPTGESYSGT--------------
Frog Kank2            -----------------WRGAAKATDGEEERVYKSKEVVEKPLLS------------------------PSGPAPKFHLVKKISITGHCSTEE-------
Mouse KANK2           -------------------SHEVVETMCPLPTATTGNVHTAKKIS--------ITERSCTGAPRMTEPSSVNPRPAAASVVQPEN--------------
Rat KANK2             ----EVVETMCPLPTASTSNMHTAKKISITERNCTGAARMADPPP---------SPAEPSPSSPYPAAEPENPAPAAQDTTDREL--------------
Dog KANK2             -------------------THEVVETAFPTATASTGNVHLVKKISITERSCDGTAELPPAPAESPSSPPGVEATSLAQPEKSAGGSAARAAAD-------
Human KANK2           --SQEVVETMCPVPAAATSNVHMVKKISITERSCDGAAGLPEVPA--------------------ESSSSPPGSEVASLTQPEKSTGRVPTQEPTH----
Aligator KANK2        ----------------KVLGVGEQTPGPGGQRLGVGSAGDQAQGA----GERAEVVRVVQVPRGPEIAASTAAGPAAQPQHARGL--------------
Spotted gar Kank2     GGTEAELTVTHRREEPQETSFPVHSSNQLQKKSVEGPVSVPGHYPNQGFLQEVNE-QVKATEDQDGTGKSNPTSPTAIHGKDQDLPYSVETPYGY-----
Cave fish Kank2       PRELPIAISSKQVREVLRSEVSMSVPVSSSALVMETKCNQITSLCLRLKEEETHQQSPTELIQSQEDAAKPAPQIRALKNFFTEYFGLFNTVLKL-----
Herring Kank2         EEEACQHTTVEVLQAEESTKPASPQSTLRSIMKRKADGEPGSPTT----KKNLQF-IGVNGGYESTSSEESSSESSEDESDTSEY-HEAPEKLPE-----
Zebrafish Kank2       AQLEASHRRYGESPAAGDSPHEFPIAISSKQVREVLRSEVSKSVPVTNQATPMETKCNQVTAVCQRLKEGEINQQPAEEAIQVDPANPASPQSNL-----
Tetradon Kank2        --------------------ICSPPAATEGHRGGQAQGPVTRCVC--------------CIPKRRTHNRTHRRRPSTRRM-------------------
Fugu Kank2            --------------------ICSPPAAAEGRHRGLAEGLDSASKTTYTPVEFPLA-VSSKQVRDVLKSELSTSVPIAAPAIARGRRTTASTSRFY-----
Medaka Kank2          ---SCSSPSPRPASGVTESQHKEKTEGSLTQKTSSGAANRSQLQTGLSTSVPIAN---------AAVAVATGNQINLVQSEEEETSSSVQSQENS-----
Sticklebakc Kank2     ----------------ICSSPSLLPACGAVEGHHRGQAEVEALKCYFCLADAPVE-LPIAVSSKQVRDVLKSELSTSVPVANPAFAV-------------
Tilapia Kank2         ---TCSSPPIQPAAGGVEDHHRGRTEVITENACCSAFSLTPDAPVELPIAVSSKQ-------SRLSTSVPVANPAIAVASDDRIVHNKEEANESN-----
Amazon molly Kank2    ---TCSSPSLQPAGGVTEGHHKGQTEAPVELPIAVSSKQVRDVLKSGLSTSVPVSNPAIVVASGDQIGLVQTKEESPEVFSKSASSPQSSLRSIM-----
Platyfish Kank2       ------------------------SKVPVELPIAVSSKQVRDVLK-----------SGLSTSVPVSNPAIVVASGDQIGLVQTKE--------------
Coelacanth KANK2      ----SASPAVPVNLQAPVPQRTEPVESSTVGAPKNEGAYLDQVPVFKTLFTSKTE-VPYILYKNPEAVETVFPIQADHCTEVLHVVKKISLTTET-----
Anole lizard KANK2    -----------------QTLESKEPYASLRALAESEEVKMPVCATYHSSEVMETTFPVHAGSATPSSTFHCVKKISIAGTEEDGN--------------
Turtle KANK2          ---------ETAFPIHAAAPHAAPIHGVKKISIAAEPGGEETGPG------------------GGASPAKINPAQTGIGASTHGL--------------
Tetradon Kank1b       NTAHALIRDASMNTVLHTRNRHSNTSQAMTRTVSVGNLAQDMKRT-------------PESCRGCASALKQIQAQASKVTRDIGV-GFININDSF-----
Medaka Kank1b         NTRQAFINDSSTNTLLSTQDKHTNTTHTLTRTVSVGS-RVRDFMC----TTEYRS-VGVNTVSLHESISRDTPETVIKITRDSGV-GFTSIYENF-----
Amazon molly Kank1b   NTSQTFSNDSSTNTLLSKHDKHTNTSHTFTRTVSVGNG-VGGIIC----TTETRT-VGVNTT-NLECYSKLPPDTVNKATRDCGV-GLTNIYENF-----
Platyfish Kank1b      TSQTFSNDSSTNTLLLSKHDKHTNTSHTFTRTISVGSG-VGGIIC----TTETRT-VGVNTANS-ECYSKLPPDTVNKATRDCGV-GLTNIYDNF-----
Frog Kank4            ---------------GPEAQSHITSNIDTEQRLDNSDMNITEAMP-SCITVETEH-LDAHISQSHNLNNPESVVCITEDQTPTDA--------------
Zebrafish Kank1b      TLQACVSEASTNTKHTLTRERHTNTANTVTRSLAIGDGKVCDRVA----AVQMRT-VGTTAY---LDGCTSAPDVPNVKTCDIGV-GMANINENI-----
Tilapia Kank1b        TLQACVSEASTNTKHTLTRERHTNTANTVTRSLAIGDGKVCDRVA----AVQMRT-VGTTAY---LDGCTSAPDVPNVKTCDIGV-GMANINENI-----
Fugu Kank1b           TLQACVSEASTNTKHTLTRERHTNTANTVTRSLAIGDGKVCDRVA----AVQMRT-VGTTAY---LDGCTSAPDVPNVKTCDIGV-GMANINENI-----
Cavefish Kank1b       TSQAHVSESSTNTTNMPTRERHTNTASVVTRTLAIGDGKVNDQSP----ALKVRS-IAVGTTGY-LKESTIAPSVPKVMTRDTGV-GLANVNENY-----
Spotted gar Kank4     ---------------TETQPKDLRSSKNQTDCVSTNEVHVSVKVLT---EDKSVE-AVVSTCDKAVETAQLVTACPNTAQEDPPI-DCTHNKDGV-----
Coelacanth KANK4      -DDTTTDSSMSSGADRCDDKAKEAVIVVTDKAVDIDQTLLLKSSIHKLSSSKETT-VVTQDPISKSNVQSLINDNDSESITFAQTEGSRLVLEES-----
Frog Kank1            NTDTLCCIDSSTNTALSTYSKQTNTETVEMRSVAIGDGKVKEVNS-----------LPKTRSIGVSTSDSDFESSTP-KTKDFGV-GHINIMDNF-----
Shark Kank1           NTEQVAHMESSTNTLASSCDKETSTAIIEMRTVAVGDGRVKDVST----ALKTRS-VGVGT----TATSDIDLEKSSVKTKDSGI-GQINVNESY-----
Coelacanth KANK1      STETAILTDSSTNTVTSAFDKQTNTIIVETRTVAVGDGRVKDFST----SPKTRS-IGVGPM---ASVSIGIEKPTAVKTKDCGV-GLISISENY-----
Mouse KANK1           NTEAATLVESCTNTLLSTMDKQTSTQTVEMRTVAIGEGRVRDINP----STKTRS-VGVGTV---LSGNSEFDRPCAVKTKESGV-GQINIQDNY-----
Rat KANK1             NTEVTTLVESCTNTLLSTLDKQTSTQNVEMRTVAIGEGRVRDINP----STKTRS-VGVGTV---LSGSSDFDKPCAVKTKESGV-GQINIHDNY-----
Dog KANK1             NTEVATLVESCTNTSVSTLDKQTSTQMVETRTVAIGEGRVKDVNS----SAKMRS-VGVGTV---LSGSAGFDRPSAVKTKESGV-GQININDNY-----
Human KANK1           NTETATLIESCTNTCLSTLDKQTSTQTVETRTVAVGEGRVKDINS----STKTRS-IGVGTL---LSGHSGFDRPSAVKTKESGV-GQININDNY-----
Anole lizard KANK1    NTEVATVTDSSTNTFLSTYDKQTNTVIVEMRTVAVGDGRVKDVNA----AAKTRS-IGVGTL---LSTTSSFDRSSLTKTKDCGV-GQININENY-----
Eagle KANK1           GTEMAALVDCETNTALSSCDKQTNTESVETRSVAVGDGRVKDIHA----SAKTRS-VGVGTL---LSSHPGFEKPSAVKTKDCGV-GQINVHENY-----
Chicken KANK1         NTEAPVAVDCGTNTVPSSRDQQTSTVSVEMRTVAVGDGRVKDIHA----SAKMRS-VGVGTL---LSSHPGFEKLSVIKTKDCGV-GQININENY-----
Goose KANK1           STEVLVLADCSTNTVPSGHDKQTNTVSVETRTVAVGDGRVKDIHA----SAKTRS-VGVGTL---YSSHPGFEKPSAIKTKDCGV-GQININENY-----
Turtle KANK1          NTESASLLDSSTNTILNTYDKQTNTVNVEMRTVAVGDGRVKDINT----SAKTRS-VGVGTL---LSSDSSFDKPSVIKTKDCGV-GQINIHENY-----
Aligator KANK1        STETVSLMDSSTNTVWSAYDKQTNTVSVETRTVAVGDGRVKDINA----SVKTRS-VGVGTL---LSSYSSSEKPSVVKTKDCGI-GQININENY-----
Spotted gar Kank1     NTETAVFMDTCTNTTLKTQDKQTNTVPTETRTVAVGDGRVKEVQM----TVKTRS-VGVGTT---VRSESELEKPSVVKTKDCGV-GQAGIHENF-----
```

```
Herring Kank1        STEQIVLVDSCTDTQLCTIDKHTSTASTETRTVAVGDGLVKDVQA----TVKTRS-IAVGTAA--LGDTESERLSAAAKTKDSGV-GQASMHDNF-----
Zebrafish Kank1a     NTDKLVLTDTSTNTQSCSKDKQVGTEPLETRTIAVGDGLVKDAPA----TLKTRS-VSVGTT---SSDDAFLDKLPSCKTKETGI-GHTSIHENF-----
Cave fish Kank1a     NTESVILIDSCTNTQSNSKDKQTSTVPLETRTVAIGEGLVKDIQA----TVKTRS-IAVGT----TASEDTTEKLASSKTKESGV-GHASVHENF-----
Stickleback Kank1a   NTERSVRTDSSTDTGLLTCEKHTNTVGTDTRTVAAGEGLVRDAHP----AARGRT-VAVGTG---ADAGSILGGPAAARTRECGV-GPVSVQENF-----
Medaka Kank1a        NTNTPALTDSSTHMHLSTCNRHTNTPPTETRTVAVGEGLVKDALS----GAKMRS-VAVGTI------TDAPAKPASAKTKECGV-GPLSVRDNF-----
Fugu Kank1a          NTKTAVLTDSFTDMTFITCDKQTSTAAIETRTVAAGEGLVKDVNS----SAKARS-VAVGTTA--DMESFLGKSSCSAKTKESGV-GAVSIHDNF-----
Tetradon Kank1a      NTKTVVLTDCFTDMTFITCDKHTNTAVTETRTVAAGEGLVKDVTS----ATKMRS-IAVGTTA--SVDAFLGKSSCHTKNKECGV-GAVSIHDNF-----
Tilapia Kank1a       NTKHAVLSDSFTHMESVTSDKQTNTVVTETRTVAAGEGLVKDVHS----TAKMRS-IAVGTT---SDVELLPGKLSSTKTKECGV-GPVSVHDNF-----
Amazon molly Kank1a  NTRTAEVTDSCTDMERTSCDKQTNTAPPETRTVAAGEGLVKDAPT----TAKTRS-VAVGTT---SDVGSLPSKPNPTRTKECGV-GPVSIHDNF-----
Mouse KANK3          ------------------------------PRPTAGDEMAP-----------------------------------------------------------
Rat KANK3            ------------------------------PRPTAGDEMAP-----------------------------------------------------------
Dog KANK3            --------------------------------PTDGDRAVAP----------------------------------------------------------
Human KANK3          -----------------------PGSMDGDRAVAP-----------------------------------------------------------------
Lamprey              ---------------------------ATQAATPAAEGEIAD-------PTDTIR-RGPSESASDDVFETEGQAAPPVAEAEAEQRGTGHVNAHF-----
Coelacanth KANK3     --------------------KGSQTDPEKLGEVCVSSSIQKVDVNSREEKSVFFD-QDMVKVEKEVEPGEKKRHVSDWEVRTSGK---------------
Frog Kank3           -----EKSTQANLEKNTEVQSSMSSTIIEAISMPRSDLILKSKCK---MTLLAQDEVDVAKNNQDITKHVEKCDKTEESITVAGKVTALNLTTEG-----
Turtle KANK3         ----------------EKDTQADIEAGIDAAMEETDQGVAGDKSK----QTPNGA-GEGGNQVDVAESGVASGTKTAMPQGGTGT-VEVEQGSRL-----
Aligator KANK3       -----------------EKATQANKSQFRGGEHCAKCCSSGRSRSTGSADKSTQRQKGAGETGNQVNVTEPGAAGGSKAAGAPGEKGPSE----------
Eagle KANK3          ----------------DKGSQTDVGGTVLAGETEPGAGHAPSGPS-----------ARGTGAMGGTCQGSPVPRAATPETTHSGR---------------
Chicken KANK3        ------------------PEAIGAAVGPAALSAEVEDGSQEPAQD-------------------------------------------------------
Goose KANK3          ----------------------------------------------------------------------------------------------------
Spotted gar Kank3    ------------------------QINRDTQTESRGEAREAIPVT-----------STGSQWEKQDGPETGGEQKAPVAVLK------------------
Zebrafish Kank3      --------------------------------------------------------------QSIQTDPEETSKEQPVELISTGV---------------
Cavefish Kank3       ----------------------------IGVGVSDSDFPVSKTDQ----SMQTDS-VECSEEKTPVTRVSTGVQCENLLST-------------------
Herring Kank3        -----------------------QQSVQTDEEVEVKKEEVAKVET-------------VSTGSQWESPEEEDEEEGEEEPQQVQKRGQVTIAEAV-----
Stickleback Kank3    ----------------------AEKQEGDQGEKTSANAKTASIMA-------------------------------------------------------
Fugu Kank3           --------------------QQSTQTDAPIKTAACDPAVLVSTGSQWENLSEDNTEHKVLKKRQLIIAEYKVSPEELVSTAEKKE---------------
Tetradon Kank3       ----------------------------------------------------------------------------------------------------
Medaka Kank3         ---------------AIQTDAMTAPEPAPVEQVSIGCQWENMTED--------------------KAEEQKLTKKRQLTINEYKV---------------
Tilapia Kank3        ----------------------------------------------------------------------------------------------------
Amazon molly Kank3   -------------------EQGTQTDATPEPEPEPEQTPVER--------------VSVGCQWENLRGENPEEEKVAVPKK-----RQLTIAEYK-----
Platyfish Kank3      -------------------EQGTQTDATPEPEPEPAPMERFSVGC------------------QWENEEKTGEQEEETRTSDTKT---------------

                              2110      2120      2130      2140      2150      2160      2170      2180      2190      2200
                     ....|....|....|....|....|....|....|....|....|....|....|....|....|....|....|....|....|....|....|....|
Hydra                ---------------------------------------------------------------------------CSKCIANEDKFVFDIDILKLQQPAL
Trichoplax           ----------------------------------------------------------------------------------------------------
Aplasia              TSSSTTSYSGYSSGGSNLASGLTSGQQISSSSLLSGDSSVRESTGGYQTSMSSSSYSSSSRGVGGGQQQISSSLLSGDSGVGSGGFQQTSMSSSGYSSSS
Snail                -------------IIHSNNDISTTEPKSSLDGHNTHKHSGSSKVEKIHINLMTNP------------------YRTLENLSEVSALVDSQSDEIDSNSVS
Fruitfly             ------------------------------------KRDVGCGDIVKPHISIACA----------------------------ANYCDSCKEAIQDLARG
Hookworm             ----------------------------------------------------------------------------------------------------
C.elegance           -------------LTNEQKSNFQRGTAVSKSARYDRKRSVDDLDYIVDKKNATSP--------------ATSSQEAPPTVPDASESPLTTKVVLKKEVFI
Tunicate             ---------------------------------LTDKVDEATSTDDLVKQKHSTQ---------------------------IDDYVDKVQSLLEEQQSL
Spider               -----------------------------DKGSVHQKENAAVGDFDVNDLSCEQC---------------------------------QNNSIQRNEMQS
Lingula              --------FISKRGSGDGKTGTSGGGGGGRSSSRVEKVISSSGPLEDSSAGGGFTKRTEYNVTSSGHVGSSGRIGAGGGGGGSGGYTRKTERIISSSTSS
Lancelet             -------------CDKCSNLKTRSIGCTTLANWNQKSVSVLAKVEQTDAGTGDDS----------VLEETVDSGKDQTETKDIGVGEDSIFPLEEVKPPV
Medaka Kank4b        --------------------------------------------------------------------------------AAVLHHIRRIKNLLDQQWEC
Amazon molly Kank4b  ------------------------------------------------------------------------------------RHIKRIKSLLDQQWEC
Platyfish Kank4b     ------------------------------------------------------------------------------------RHIKRIKSLLDQQWEC
Stickleback Kank4b   ----------------------------------------------------------------------------------------------------
Fugu Kank4b          ------------------------------------------------------------------------------------HHIRKLKRLLDQQWEC
Tetraon Kank4b       ----------------------------------------------------------------------------------------------------
Cave fish Kank4b     ----------------------------------------------------------------------------------MEHHVKRVKELLHEQWEC
Medaka Kank4a        ------------------------------------------------------------------------SSDSPTSPAALGQVVTRLTGLLGEQWAQ
Fugu Kank4a          ------------------------------SEAQASRRDSGDGQTPRRGSGESPT-----------------------SPAALGQVVTRLTGLLGEQWAQ
Tetradon Kank4a      -----------------------------SLSHNEDVAEKLQGEPGSGESPTSPA--------------------------ALGQVVTRLTGLLGEQWAQ
Stickleback Kank4a   -----------------------QGSRRSSAETQAPQKMPGETQSGSGESPTSPA--------------------------ALGQVVTRLTGLLGEQWAQ
```

```
Tilapia Kank4a       ----------------------------GSSEAQGSRRDPGEAQPPRRGSSESPT-----------------------SPAALGQVVTRITGLLGEQWAH
Platyfish Kank4a     TKTRQIRHGSGDAQSHSQSLRRSSTEAPSSQKDARETQSLRRGSSEAAQRRGSSEAKALRRESGESQTARRGSGESPTSPAALGQVVTRITGLLGEQWAQ
Amazon Molly Kank4a  TKTRQIRHGSGDTQSHSQSLRRSSTEAPSSQKDARETQSLRRGSSEAAHRRGSSEAKALRRESGESQTARRGSGESPTSPAALGQVVTRITGLLGEQWAQ
Herring Kank4        ---------PAANVVTRIQGLLNEQWSSLGSGSPEPSQSAPPTPPPPSKFSSIQKGEGSSSSSLTAVRYRHKGSGSGSASSPAANVVTRIQGLLNEQWSS
Zebradish Kank4      --------------------------EGSSGLQTQQEPKETQGQDPHPQSQRASE--------------------ATASPAAIGQVVNRIQGLFNEQWAS
Cave fish Kank4a     -----------------VVIETSTGDTSTGESQQGQDQKQEQGQDTQPQPQTQPQ----------------RSTETATSPAAIGQVVNRIQGLLNEQWAS
Mouse KANK4          ------------------------STRESAPVIREEATSELPGAERPGRPASSPQ------------------------DATIGQYVKKIQELLHEQWNC
Rat KANK4            -----------------------------APVIREEASSQLPGAERPGRPASSPQ------------------------DATIGQYVKKIQELLHEQWNC
Dog KANK4            -----------------------SSDRKTPPAGREEASSEPLGKERPGRPPSSPP------------------------DATIGQYVKKIQELLQEQWSC
Human KANK4          ----------------------------TPPAGREETSSNLPGKEHPGRPPSSPT------------------------DATIGQYVKKIQELLQEQWNC
Eagle KANK4          ------------------------------------------------------------------EEEGPPDHEDLPAVDPIGQYVKKIQELLQEQWLC
Chicken KANK4        ------------------------------------------GEEGLPDPKDFPA------------------------VDPIGQYVKKIQELLQEQWMC
Goose KANK4          -------------------------------GFDEDKTEDGMEDDGHPDHKDFPA------------------------VDPIGQYVKKIQELLQEQWMC
Anole lizard KANK4   -----------------------------------------EGNNSEPKKVTTDP--------------------------SVSQYVKKIQDLLQEQWMC
Turtle KANK4         -------------------------PGGNKPVFAGDVKQDLIEEENHTEQQDSSA------------------------DDPIGQYVKKIQELLQEQWMC
Aligator KANK4       ---------------------------DLKKGPAEDVKQVLMEEERHTDQQDSSD------------------------ADPISQYVKKIQELLQEQWMC
Frog Kank2           ----------------------------------------------------------------------------------------------------
Mouse KANK2          ----------------------------------------------------------------------------------------------------
Rat KANK2            ----------------------------------------------------------------------------------------------------
Dog KANK2            ----------------------------------------------------------------------------------------------------
Human KANK2          ----------------------------------------------------------------------------------------------------
Aligator KANK2       ----------------------------------------------------------------------------------------------------
Spotted gar Kank2    ---------------------------RLDLDFLKYVNDIEKGNTIKKVPIHPIC-------------------------------SKEVREVLQKSEVS
Cave fish Kank2      ---------LLRFLMFLHKLINHCSFAYVNFCDLHYRYESTTTEESSSESSEAES--------------------------DVSEYHEATEKLPDSTAAE
Herring Kank2        -----------------------SGARPPHPEATGPPATAAAQEPPFPEPPQQLS---------------TAQPTANEPPVPQSTSQSPVTDASLQQTVS
Zebrafish Kank2      --------------RSIMKRKADGEPGSPYTKKNLQFLGVNGGYESTSSEESSSE--------------------SSEDESDASEYHEATEKLPESATPQ
Tetradon Kank2       ----------------------------------------------------------------------------------------------------
Fugu Kank2           ----------------------------------------------------------------------------------------------------
Medaka Kank2         ----------------------------------------------------------------------------------------------------
Sticklebakc Kank2    ----------------------------------------------------------------------------------------------------
Tilapia Kank2        ----------------------------------------------------------------------------------------------------
Amazon molly Kank2   ------------------KRKAEGEPGSPSTKKNLQFIGVNGGYESTSSDDSSSE--------------------SSDDGSDSSEYHEAREKLPESTNQL
Platyfish Kank2      ----------------------------------------------------------------------------------------------------
Coelacanth KANK2     -------------------------------------------------------------------------------------YEDKLKGVDDPAVLV
Anole lizard KANK2   ----------------------------------------------------------------------------------------GNEDLTESQRLL
Turtle KANK2         -----------------------------------------------------------------------------------------WRQVIRMGWVL
Tetradon Kank1b      -------------LVGLKTRNMASGPSHLADPIRMRSVGVGEGRIRDVGLSSSHP----------------ADKLQPRWDPELNHYIGKMHKLLGQEDCV
Medaka Kank1b        -------------LVGLKTRNMASGPSHLPDPVKTKSVGVGDGRVQEFTVTSSTS---------------GHTTQQCQRGHELNHYIDKLHRLLSEHGDL
Amazon molly Kank1b  -------------LVGLKTRNMASGPSHIPDPIKTRSIGVGDGRIRDFSMSSCAP------------KPKFQQPSQFQWDHELNHYIEKMHRLLKDHGDL
Platyfish Kank1b     -------------LVGLKTRNMASGPSHIPDPIKTRSIGVGDGRIRDFSTSSCAP------------KPKSQQFSQFQWDHELNHYIEKMHRLLKENGDL
Frog Kank4           ---------------------------------------------------------------------------------SVGQYVKRIQDLLQEQWTC
Zebrafish Kank1b     -------------LIGLHTRNIACGPSRLPDPVKTRSIGVEVGDGRIRDPEGQMF---------------------VQNEPGLDHYIDRMQKLLKEQQDL
Tilapia Kank1b       -------------LIGLHTRNIACGPSRLPDPVKTRSIGVEVGDGRIRDPEGQMF---------------------VQNEPGLDHYIDRMQKLLKEQQDL
Fugu Kank1b          -------------LIGLHTRNIACGPSRLPDPVKTRSIGVEVGDGRIRDPEGQMF---------------------VQNEPGLDHYIDRMQKLLKEQQDL
Cavefish Kank1b      -------------LVGLRTRNIACGPSRLPDPIKTRSIGVEVGDGRIRDVNGHIQ----------VLAHPLQPHLCAESEPGLDHYIDRMQKLLKEQQCL
Spotted gar Kank4    ----------------------GCVAEQMQEWQKEGTDESGSKALHQQETDESTS-----------------------SQAALGHYVTRIQELLHEQWAC
Coelacanth KANK4     ----------------------GQSEQSECDSMNVNTKAFQDDLGNKEQVKDETQ----------------------QYPAIVGQYVNKIQVLLQEQWAC
Frog Kank1           -------------LIGMKSRSIACGPSE-ALPLGPDTRSIAVGDEPFQEQECIPL---------------INKEPTPDTPAVIDQYIEQVQKLLQEQQML
Shark Kank1          -------------LVGLKTRHIGCGTVSPTL-ANGNTKSVGVGDEPACVAEGGQP-------------------AVQDPAPQLHHYIELVQKLLQEQQTL
Coelacanth KANK1     -------------LIGLKTRNIACGPTNLPN-MPANTRSVGVGDKLISVASQEAK---------------------SEATVGVDHYIERVQKLLQEQQML
Mouse KANK1          -------------LVGLKMRTIACGPPQLTVGLMGSRRSVGVGNEPVGELPEESP----------------QPRVASGMVTGLDHYIERVQRLLAEQQTL
Rat KANK1            -------------LVGLKMRTIACGPPQLTVGLIGSRRSVGVGNEPVGDLLEDPL----------------QPQVPSGMMTGLDHYIERVQKLLAEQQTL
Dog KANK1            -------------LVGLKMRTIACGPPQLTLGLAASRRSVGVGDEPVGEFMESPQ-----------------LQAPPGMMTGLDHYIERVQKLLAEQQML
Human KANK1          -------------LVGLKMRTIACGPPQLTVGLTASRRSVGVGDDPVGESLENPQ-----------------PQAPLGMMTGLDHYIERIQKLLAEQQTL
Anole lizard KANK1   -------------LVGLKMRNIACGPPPVSV-APSSTRSIGVGDESVYESENFVL---------------DCPLPLPEMKTGLDHYIERVQKLVQEQQML
Eagle KANK1          -------------LVGLKMRSIACGPPPLPV-VPAGTRSIGVGGESVCEPVSGLL---------------ESPLPPPELRTGLDHYIERVQKLLQEQQML
Chicken KANK1        -------------LVGLKMRSIACGPPPLVV-VPTSTRSIGVGGDPVCESVSSLL---------------ESPLPPPELRTGLDHYIERVQKLLQEQQML
```

```
Goose KANK1           -------------LVGLKMRSIACGPPPLVV-VPTGTRSIGVGGEPVCESVSGLL---------------ENPLPPPELRTGLDHYIERVQKLLQEQQML
Turtle KANK1          -------------LVGLKMRNIACGPPPLSV-VSASTRSIGVGDEPVYEPVSILV---------------ESPLPPPEMRTGLDHYIERVQKLLQEQQML
Aligator KANK1        -------------LVGLKMRNIACGPPPPSV-VPASTRSVGVGDEPVYQSVSALV---------------ESSLPPPEMRTGIDHYIERVQKLLQEQQML
Spotted gar Kank1     -------------LVGLKTRTISCGPSH-QITDSAKTRSIGVGDESISEAGQIQQ-------------------SQLGTAAGLDHYIEQVQKLLQEQQML
Herring Kank1         -------------LVGLKTRTIACGPSLPQLPNLSQVGESPAPTLIQVPPLACPL------------------PDQPATGAGLDHYIERVQKLLQEQQML
Zebrafish Kank1a      -------------LVGQKTRNIACGPSQSPTPTHVKSNFEESVLTEGPNQSQAGA-------------------------------------GVGLDHYIERVQKLLQEQQML
Cave fish Kank1a      -------------LVGLKTRNIACGPSQPCNQASEKSTTVERMVTPEKSLQLQAS----------------------------------AGCGVSLDHYIEKVQKLLQEQQML
Stickleback Kank1a    -------------LVGLKTRNIASGPSQPSGSEGAVRVE--------------------------------------------DHYIERVQKLLQEQQML
Medaka Kank1a         -------------LIGLKTRSIACGPASSVELSQSEAAAPLAA------------------------------------AVGLDHYIERVQKLLQEQQML
Fugu Kank1a           -------------LVGRNTRNIACGPSQPAGSPTSKEATEVKASPAPAQAQECPG----------------------------------------LDHYIGRVQKLLQEQQML
Tetradon Kank1a       -------------LVGRNTRNIACGPSQPNGSLTSKETTEVKASPAPAQAQE---------------------------GAGLDHYIERVQKLLQEQQML
Tilapia Kank1a        -------------LVGLKTRNIACGPSQPAESVASIGTQETAEVKPALAAVHADP-----------------ATLQAHGGVSLDHYIERVQKLLQEQQML
Amazon molly Kank1a   -------------LIGLKTRNIACGPSQPRDAAPSKEEKTEAAAAAP-----------------------------PPGGVGLDHYIERVQKLLQEQQML
Mouse KANK3           ----------------------------------------------------------------------------------------------------
Rat KANK3             ----------------------------------------------------------------------------------------------------
Dog KANK3             ----------------------------------------------------------------------------------------------------
Human KANK3           ----------------------------------------------------------------------------------------------------
Lamprey               ----------------------------------------------------------------------------------------------------
Coelacanth KANK3      ----------------------------------------------------------------------------------------TVEVITVHKRIT
Frog Kank3            ----------------------------------------------------------------------------------------------GDEHCL
Turtle KANK3          ----------------------------------------------------------------------------------------------------
Aligator KANK3        ----------------------------------------------------------------------------------------------------
Eagle KANK3           ----------------------------------------------------------------------------------------------------
Chicken KANK3         ----------------------------------------------------------------------------------------------------
Goose KANK3           ----------------------------------------------------------------------------------------------------
Spotted gar Kank3     ----------------------------------------------------------------------------------------------------
Zebrafish Kank3       ------------------------------------------------------------------------------------------------QWEC
Cavefish Kank3        ----------------------------------------------------------------------------------------------------
Herring Kank3         ------------------------------------------------------------------------------MGSGAGRRV-------------
Stickleback Kank3     ----------------------------------------------------------------------------------------------------
Fugu Kank3            ----------------------------------------------------------------------------------------------------
Tetradon Kank3        ----------------------------------------------------------------------------------------------------
Medaka Kank3          ----------------------------------------------------------------------------------------------------
Tilapia Kank3         ----------------------------------------------------------------------------------------------------
Amazon molly Kank3    ----------------------------------------------------------------------------------------------------
Platyfish Kank3       ----------------------------------------------------------------------------------------------------

                            2210      2220      2230      2240      2250      2260      2270      2280      2290      2300
                      ....|....|....|....|....|....|....|....|....|....|....|....|....|....|....|....|....|....|....|....|
Hydra                 CHYCG---------------------------------------------------------------------------------NKVDLNDTNLDES-
Trichoplax            ----------------------------------------------------------------------------------------------------
Aplasia               GGVSGGVSGGVSGGVSGGVSGGVGGGQQQISSSSSSVVSGGDVSSTRVEKSYSTVVTNPYRTVENLAAASSLVSGSDSLDGGSSSSSSSSSRLMSKTSSE
Snail                 ISKTSSD-------------------------------------------------------------------------------GNLYSERTSLNET-
Fruitfly              FSKAS---------------------------------------------------------------------------------PTATRTGGAVRRS-
Hookworm              --------------------------------------------------------------------------------------PRPKISKYALPAE-
C.elegance            EPPKQ---------------------------------------------------------------------------------MLPDPIPARIPRP-
Tunicate              LAENY-------------------EGFNDVIN-------------------------------------------------------KEEKTEEKKVSRF-
Spider                VGINT---------------------------------------------------------------------------------TIDSSSHLNVSSG-
Lingula               GGSGGVSSTVSSSSGGGGSGMAGSGSRSSER-------------------------------------------------------TVTSTVHHDLPSN-
Lancelet              LIQEE--------------KTVNVVSFATAAR------------------------------------------------------PPLEIVESSSSDE-
Medaka Kank4b         VCAEG-----------------KPGDKGRAL-------------------------------------------------------KHLDPKVNSLQEE-
Amazon molly Kank4b   LCGEF-------------------GEKGKPP-------------------------------------------------------KHPDSKVNSLQQE-
Platyfish Kank4b      VCGQF-------------------GEKGKPL-------------------------------------------------------KHPDPKVNSLQQE-
Stickleback Kank4b    --------------------------------------------------------------------------------------QPPGEQ--------
Fugu Kank4b           LCANI---------------------------------------------------------------------------------PSGNPKVRSLQQE-
Tetraon Kank4b        ----------------------------------------------------------------------------------------------------
Cave fish Kank4b      LCR-------------------------------------------------------------------------------------------------
```

```
Medaka Kank4a         LKPAG---------------------------------------------------------------------------------KPGPSKMSSIQSQ-
Fugu Kank4a           LGSSS--------------------GAQQTAG------------------------------------------------------QQGPSKMSSIQSQ-
Tetradon Kank4a       LGSSS-------------------GAQQPASQ---------------------Q--------------------------------ESPASKMSSIQSQ-
Stickleback Kank4a    LGSST--------------------TAGKAAG------------------------------------------------------KPGPSKMSSIQSQ-
Tilapia Kank4a        LGSSS-------------------GAQQTAS-------------------------------------------------------QQGTSKMSSIQSQ-
Platyfish Kank4a      LGSSSGAQQTAGQQESPIAPKQTATKRAEAGK---------------------GTPAKPAGKGAPAATTGKAAG------------KSSASKMSSIQSQ-
Amazon Molly Kank4a   LGSSSGAQQTVGQQESPIAPKQTATKRAEAGK---------------------GTPAKPAGKGAPAATTGKAAG------------KSSASKMSSIQSQ-
Herring Kank4         LGSGS-------------------PEPSQSAPPK----------------------------------------------------PPQPSKFSSIQSQ-
Zebradish Kank4       LGSGG------------------QDAKGESSQ------------------------------------------------------KPHSSKISSIQSH-
Cave fish Kank4a      LGSGS------------------QEGKGESSQ------------------------------------------------------KQPVSKISSIQSH-
Mouse KANK4           LEHGY-------------------PELASAI-------------------------------------------------------KQPASKLSSIQNQ-
Rat KANK4             LEHGY-------------------PELASAI-------------------------------------------------------KQPASKLSSIQNQ-
Dog KANK4             LEHGY-------------------PELASAI-------------------------------------------------------KQPASKLSSIQSQ-
Human KANK4           LEHGY-------------------PELASAI-------------------------------------------------------KQPASKLSSIQSQ-
Eagle KANK4           LEHGY-------------------PELASAI-------------------------------------------------------KQPASKLSSIQNQ-
Chicken KANK4         LEHGY-------------------PELASAI-------------------------------------------------------KQPASKLSSIQNQ-
Goose KANK4           LEHGY-------------------PELASAI-------------------------------------------------------KQPASKLSSIQNQ-
Anole lizard KANK4    LEHGY-------------------PELASAI-------------------------------------------------------KQPASKLSSIQNQ-
Turtle KANK4          LEHGY-------------------PELASAI-------------------------------------------------------KHPASKLSSIQNQ-
Aligator KANK4        LEHGY-------------------PELASAI-------------------------------------------------------KQPASKLSSIQNQ-
Frog Kank2            --------------------------------------------------------------------------------------PATEGNTGGILAQ-
Mouse KANK2           --------------------------------------------------------------------------------------PVPAAQDTTDKKP-
Rat KANK2             --------------------------------------------------------------------------------------TRPVASQDSQAAE-
Dog KANK2             --------------------------------------------------------------------------------------GEPTRQAASCQSQ-
Human KANK2           --------------------------------------------------------------------------------------REPTRQAASQESE-
Aligator KANK2        ---------------------------------------------------------------------------------------EPRDGPDLLEMA-
Spotted gar Kank2     SSVPV---------------------------------------------------------------------------------CSPAVVMETVSAT-
Cave fish Kank2       QPETS---------------------------------------------------------------------------------STSVPSVPSVPTE-
Herring Kank2         LS------------------------------------------------------------------------------------PATAVATGHCATQ-
Zebrafish Kank2       SLVSSCI-------------------------------------------------------------------------------PQLASETPATQTA-
Tetradon Kank2        --------------------------------------------------------------------------------------EPPQAFPPRFRSE-
Fugu Kank2            --------------------------------------------------------------------------------------ARAASSP-------
Medaka Kank2          --------------------------------------------------------------------------------------SKPGSLSFLASSP-
Sticklebakc Kank2     --------------------------------------------------------------------------------------GTSGNQMGLLHAK-
Tilapia Kank2         --------------------------------------------------------------------------------------QSQEGLPKTASSP-
Amazon molly Kank2    QRVTVNKT------------------------------------------------------------------------------SQPQEESTSVPQQ-
Platyfish Kank2       --------------------------------------------------------------------------------------ESPEIFSKSASSP-
Coelacanth KANK2      PAVSS------------------SEEVSEVLQ------------------------------------------------------KPSQMIVLSASSA-
Anole lizard KANK2    VRA-----------------------------------------------------------------------------------EHPSSHPLQTEQV-
Turtle KANK2          --------------------------------------------------------------------------------------GQPRGFRNGGPGP-
Tetradon Kank1b       QEKVYYFLVQKCH-------------------------------------------------------------------------SPPQCSPDSSDCD-
Medaka Kank1b         LTGVG--------VGNSTSKTVQSGNTMSEEG------------------------------------------------------KEKHKRFCSEPTE-
Amazon molly Kank1b   LTGDQSD-------------------------------------------------------------------------------HYPSSKPLSNIKS-
Platyfish Kank1b      LTGDQSD-------------------------------------------------------------------------------HYPSSKPLSNFKS-
Frog Kank4            LEHGY-------------------PDLANAI-------------------------------------------------------RQPASKLSSIQGQ-
Zebrafish Kank1b      LTDSH-----------------------------------------------------------------------------------------------
Tilapia Kank1b        LTDSH-----------------------------------------------------------------------------------------------
Fugu Kank1b           LTDSH-----------------------------------------------------------------------------------------------
Cavefish Kank1b       LTDGHF--------------------------------------------------------------------------------EAREEQEVTIQIQ-
Spotted gar Kank4     LENGH-------------------PELASTL-------------------------------------------------------RQPASKISSIQNQ-
Coelacanth KANK4      LEHGY-------------------PELANAL-------------------------------------------------------KQPASKISSIQNQ-
Frog Kank1            LAENY-------------------TELAETF-------------------------------------------------------GEPYSQIGTLNSQ-
Shark Kank1           LAENY-------------------TELADAF-------------------------------------------------------GEPGSHIGSLNCQ-
Coelacanth KANK1      LAQNY-------------------SELADAF-------------------------------------------------------GQPNSQIGSLNCQ-
Mouse KANK1           LAENY-------------------SELAEAF-------------------------------------------------------GEPHSQIGSLNSQ-
Rat KANK1             LAENY-------------------SELAEAF-------------------------------------------------------GEPHSQIGSLNSQ-
Dog KANK1             LAENY-------------------SELAEAF-------------------------------------------------------GEPHSQIGSLNSQ-
```

```
Human KANK1            LAENY-------------------SELAEAF-------------------------------------------------------GEPHSQMGSLNSQ-
Anole lizard KANK1     LAENY-------------------AELADAF-------------------------------------------------------GEPHSQIGSLNSQ-
Eagle KANK1            LAENY-------------------SELAEAF-------------------------------------------------------GEPHSQIGSLNSQ-
Chicken KANK1          LAENY-------------------SELAEAF-------------------------------------------------------GEPHSQIGSLNSQ-
Goose KANK1            LAENY-------------------SELAEAF-------------------------------------------------------GEPHSQIGSLNSQ-
Turtle KANK1           LAENY-------------------SELAEAF-------------------------------------------------------GEPHSQIGSLNSQ-
Aligator KANK1         LAENY-------------------SELAEAF-------------------------------------------------------GEPHSQIGSLNSQ-
Spotted gar Kank1      LAENY-------------------SELADAF-------------------------------------------------------GQPHSQIGSLNSQ-
Herring Kank1          LAENY-------------------SELADAF-------------------------------------------------------GQPHTQFGSINSE-
Zebrafish Kank1a       LAQNY-------------------SELADAFG------------------------------------------------------PPQQSQFSSINSE-
Cave fish Kank1a       LAENY-------------------SELADAFG------------------------------------------------------PPQQSQFSNINSE-
Stickleback Kank1a     LAENY-------------------SELADAF-------------------------------------------------------GQPQSQFGSINCQ-
Medaka Kank1a          LAENY-------------------SELADAF-------------------------------------------------------GQPQSQLGSLNSQ-
Fugu Kank1a            LAENY-------------------SELADAF-------------------------------------------------------GQPQSQFGSINSQ-
Tetradon Kank1a        LAENY-------------------SELADAF-------------------------------------------------------GQPQSQFGSINSQ-
Tilapia Kank1a         LAENY-------------------TELAEAF-------------------------------------------------------GQPQSQFGVINSQ-
Amazon molly Kank1a    LAENY-------------------SELADAF-------------------------------------------------------GQPQSQFGSINSQ-
Mouse KANK3            ----------------------------------------------------------------------------------------------------
Rat KANK3              ----------------------------------------------------------------------------------------------------
Dog KANK3              ----------------------------------------------------------------------------------------------------
Human KANK3            ----------------------------------------------------------------------------------------------------
Lamprey                ----------------------------------------------------------------------------------------------------
Coelacanth KANK3       M-------------------------------------------------------------------------------------RSPSSDVTENHCL-
Frog Kank3             IIQNS---------------------------------------------------------------------------------GEQPEDNDTQKGS-
Turtle KANK3           --------------------------------------------------------------------------------------IPQDSRKGVHECA-
Aligator KANK3         --------------------------------------------------------------------------------------KKQSPCLSLKDSK-
Eagle KANK3            --------------------------------------------------------------------------------------DPSAAQDAGAAPSP
Chicken KANK3          --------------------------------------------------------------------------------------GRAAPSPA------
Goose KANK3            --------------------------------------------------------------------------------------ASPA----------
Spotted gar Kank3      ----------------------------------------------------------------------------------------------------
Zebrafish Kank3        LDDTE---------------------------------------------------------------------------------PQDSEEQTTSAET-
Cavefish Kank3         --------------------------------------------------------------------------------------DPQDNQEQSISAEG
Herring Kank3          --------------------------------------------------------------------------------------GEDVVAAPATPSP-
Stickleback Kank3      ----------------------------------------------------------------------------------------------------
Fugu Kank3             --------------------------------------------------------------------------------------EDVAATTTACSAR-
Tetradon Kank3         --------------------------------------------------------------------------------------KHASAQSGAEVAA-
Medaka Kank3           --------------------------------------------------------------------------------------SAEEEIVGVVKNK-
Tilapia Kank3          ----------------------------------------------------------------------------------------------------
Amazon molly Kank3     --------------------------------------------------------------------------------------VSPEEEVVVAEEE-
Platyfish Kank3        ----------------------------------------------------------------------------------------------------

                                2310      2320      2330      2340      2350      2360      2370      2380      2390      2400
                       ....|....|....|....|....|....|....|....|....|....|....|....|....|....|....|....|....|....|....|....|
Hydra                  ----------------------------------------------------------------------------------------------------
Trichoplax             ----------------------------------------------------------------------------------------------------
Aplasia                GNLYKENKKNMSRSEA-VQSMETIYDMRRAGSVEDFMPDDIRKYMSIDPSTSKDSGFGEDLLQRFSEKSSSSSTSSSSAAPSSDKARSMKSMKVSKTVTT
Snail                  ------------------KSVDTIFDMRRAGSVEDFMPDDIKKYMSIETSLTKDSGFGEDLQSRFSGNLVSSSEGLSSGGASRSVKLSKTITTTSTSKSG
Fruitfly               -----------------TSADSRIPRPKHLSSPSPVRKEFMRQNTYTLATPSPTPSPQVERKARLSSLQEKPPS--------------------------
Hookworm               ----------------------------SAETPDEEEEAVDR----------------------------------------------------------
C.elegance             ----------------KISKWAVTSEEEAAETPDEQEEAQDR----------------------------------------------------------
Tunicate               -----------INPAF-AQKDNLVVEEKLKEQTSAAISASFHYMQSTPETDESSEEEDDEEDGYIPLHEPSIVD--------------------------
Spider                 -----------------IHICDKCSATIHSVAKDLATNTVDSK---------------------------------------------------------
Lingula                ------------RKSR-TEKRVTVSSTGHSGGTSRMGGGDGRTFKTQKIVISPTGEMSEDMGFQSQVSNAEQLMSDFHGRIMGSAMQGMTSGTGGDRNVT
Lancelet               -----------V-----EDEDDMIPPLQQASIIKTTEPQVAE--------VQPVRHIVRETVVRETVVTVTPQDGYEELDGEVKHKEEEEKESLEAVDGF
Medaka Kank4b          -----------M-----MELVDILLSHYQQNGDASKSTIKTDTCAQK-----------------------------------------------------
Amazon molly Kank4b    ---------------M-MELVDVLMSGHKHPEDSDGQKIQNRASKSALESDGSTQT--------------------------------------------
Platyfish Kank4b       ---------------M-MELVDVLMSGHKHHEDSDGQKIQDRASKSTLESDGSTQT--------------------------------------------
```

```
Stickleback Kank4b     ----------------------------------------------------------------------------------------------------
Fugu Kank4b            ---------------M-MGLVDIITSYYTQHSDGEEPQLR------------------------------------------------------------
Tetraon Kank4b         ----------------------------------------------------------------------------------------------------
Cave fish Kank4b       ----------------------------------------------------------------------------------------------------
Medaka Kank4a          ----------------LVSSLSVLSAFYSPSQKGAAASKHPWTPSSTCPPPRVPLPAISS----------------------------------------
Fugu Kank4a            ---------------L-VSSLSVLSAFYSPGQKAAGAGKNPP--------FSLLSRSLRLPLSSD-----------------------------------
Tetradon Kank4a        ---------------L-VSSLSVLSAFYSPGQKATSASKHPPFSLSSLSLHLPPSSD-------------------------------------------
Stickleback Kank4a     ---------------L-VSSLSVLSAFYSPGQKAAAASKQRD--------QGRTNTPPPPSPPSLFLFI-------------------------------
Tilapia Kank4a         ---------------L-VSSLSVLSAFYSPSQKAAAASKQQEQASPLPLALSSS----------------------------------------------
Platyfish Kank4a       ---------------L-VSSLSVLSAFYSPGQKTAATSKQQ-----------------------------------------------------------
Amazon Molly Kank4a    ---------------L-VSSLSVLSAFYSPGQKAAATSKQQ-----------------------------------------------------------
Herring Kank4          ---------------L-VSSLSVLSAFYSPGQKEKAAAAASR----------------------------------------------------------
Zebradish Kank4        ---------------L-RGSLSALSAFYSPVQKGGAAR--------------------------------------------------------------
Cave fish Kank4a       ---------------L-RGSLSALSAFYSPVQKGGAAK--------------------------------------------------------------
Mouse KANK4            ---------------L-LSSLNLLLSAYSAQAPEPEPKETPAPPPSTPPPPPPPPPEISP----------------------------------------
Rat KANK4              ---------------L-LSSLNLLLSAYSAQAPEPKESPVPPSSTPPPPPEISP----------------------------------------------
Dog KANK4              -----------L-----LSSLNLLLSAYSAHAPPQKGPPAPSASPPMEISP-------------------------------------------------
Human KANK4            -----------L-----LSSLNLLLSAYSAQAHPPKEPPASSSSPPVEISP-------------------------------------------------
Eagle KANK4            -----------L-----VNSLNSLLSAYSTQGPADKENSNTH--------YQQLEISP------------------------------------------
Chicken KANK4          -----------L-----VNSLNSLLSAYSSQRPADKENSNTHYQQLETSS--------------------------------------------------
Goose KANK4            -----------L-----VNSLNSLLSAYSTQRPADKENSNTHYQQLEISP--------------------------------------------------
Anole lizard KANK4     ---------------L-VNSLNLLLSAYSTQTPTDKENSNTQYQQLEISP--------------------------------------------------
Turtle KANK4           -----------I-----VNSLNLLLSAYSTQAPSDKENSNTQYQQLEISP--------------------------------------------------
Aligator KANK4         -----------L-----VNSLNLLLSAYSTQASPDKENSNAQYQQLDLSP--------------------------------------------------
Frog Kank2             -----------------TATWSQMNVSGAAAGREGDG---------------------------------------------------------------
Mouse KANK2            --------------------------TRPAAASQDSQAADG-----------------------------------------------------------
Rat KANK2              ----------------------------GAGGA-------------------------------------------------------------------
Dog KANK2              -------------------------EVGGAGEP-------------------------------------------------------------------
Human KANK2            ---------------------------EAGGTAPPLSSPPGGP---------------------------------------------------------
Aligator KANK2         -----------------LPRLVLAPAHGHAHGAHKISIIETGAA--------------------------------------------------------
Spotted gar Kank2      -------------QLV-SNQLAPLCQRLKEGDMTQQTPHETAPAQEDNSSQPASP---------------------------------------------
Cave fish Kank2        --------------TI-APQHRTVPTPQSLSQSPASDSTDTQHCAAGQSSPTATPPQDR-----------------------------------------
Herring Kank2          -----------I-----VATASVPPLASGSSSGQGDASPQQTASHGLVPDTQRPAVQP------------------------------------------
Zebrafish Kank2        -----------------QHSTAQIPTNHTPAAQTTSQSHTTDATTQQHVTQSPAAEADVQHCVSQSSVTTTPPP--------------------------
Tetradon Kank2         ---------------------------LDPCPSSPASSP-------------------------------------------------------------
Fugu Kank2             ----------------------------------------------------------------------------------------------------
Medaka Kank2           ----------------------------------------------------------------------------------------------------
Sticklebakc Kank2      -----------------EDETSVQTRPEAVQSQEGPATASSP----------------------------------------------------------
Tilapia Kank2          ----------------------------------------------------------------------------------------------------
Amazon molly Kank2     ---------------T-AIKMPAITHLQDTKLPENAHQSPA-----------------------------------------------------------
Platyfish Kank2        ----------------------------------------------------------------------------------------------------
Coelacanth KANK2       -----------------PVAVEMGGTVLSASAQQEGPPSPEANTCSSSVASEQGSALSTRDAPQPASP--------------------------------
Anole lizard KANK2     -----------------ARCLSQEPDLNVQTEGEDQASF-------------------------------------------------------------
Turtle KANK2           --------------------------VSDPGLSARLPAASA-----------------------------------------------------------
Tetradon Kank1b        -----------INLETLAARDSEVKRMIQKLEQQTSSVLQGEYVACTCAFALLVLNLTELRKWLPPAWQVQQSEGFHLQCLNKMWCRAPTRFADKPAK--
Medaka Kank1b          -----------LIRSE-SCHLDFVAAKLEMLPTPQPLDEDCQSTTNEAEMCHRSNSEVKRMIQLLEQQASSAALQDKP----------------------
Amazon molly Kank1b    -----------------MTEGEAVIHRFTSHPTDLLSPSQPLNVSKEHSEISQQDDRSAS----------------------------------------
Platyfish Kank1b       -----------------MTEGEAVIHRFTSHPTDLLSPSQPLNVSKEHPEVSQQDGNASEVKKMIKMLEQQAASAVQDRSAN------------------
Frog Kank4             -----------L-----VNSLNLLSSAYSAQAAPAKGHSKMKNQHRAEAVP-------------------------------------------------
Zebrafish Kank1b       ----------------------------------------------------------------------------------------------------
Tilapia Kank1b         ----------------------------------------------------------------------------------------------------
Fugu Kank1b            ----------------------------------------------------------------------------------------------------
Cavefish Kank1b        ------------------DHRDTQHGVFGYGSPSKNNISEHKMRHPTRSFNDSASD--------------------------------------------
Spotted gar Kank4      -----------L-----VSSLNVLSSMYSSQVHKEKTVSLNSGSKSTRCSP-------------------------------------------------
Coelacanth KANK4       -----------L-----MSSLNVLSSVYSTQTSSEEKNVGKSSPELVRSP--------------------------------------------------
Frog Kank1             ---------------L-ISTLSSFNSVMKYGSSEELHSSGFKRHSAELDPSAGSNLDCFPESQFISQYANGNTQTINLNQLPEATQCEVQNVPKSAGKPS
Shark Kank1            -----------L-----INTLSSISSAMKYGSTEEIRSGDAVDLRPDSAEIDTGISCEHSLSTKSTSLVTTSQSAIATRVEQVTTVTTIESCETLLQQDN
```

```
Coelacanth KANK1      ---------------L-MNTLSSMNSVIKYTSTEELRNLDVPRENEDHSMRKDANCEPTTLNHSTSACMTSTTQLMMPNQEVKTTQVEKVASKTSVAEVA
Mouse KANK1           -----------L-----ISTLSSINSVMKSASTEELRNSDFQKASLGKTTGNHLEYTCKCGGLRSGGLLNVQPSQPEVEAETAEGKHSRGHEQFPMQGST
Rat KANK1             -----------L-----ISTLSSINSVMKSASTEELRNPDFQKSSLGKIAGNHLECTCKCGGLRSGGLVNVQTSQPEVETETTEGKRGCEQFLTQGNTLP
Dog KANK1             -----------L-----ISTLSSINSVMKSASTEELRNPDFPKMSLGKVT-GSLEHTCRCGGLPAGAPFSSQASRHEQAVGPTEGRPPGGQDAFPPQEST
Human KANK1           -----------L-----ISTLSSINSVMKSASTEELRNPDFQKTSLGKITGNYLGYTCKCGGLQSGSPLSSQTSQPEQEVGTSEGKPISSLDAFPTQEGT
Anole lizard KANK1    -----------I-----ISTLSSINNVMKYASTEELRNLDVPKQCIDRAAETEDNLPNTPHGSSTSSSLKLKLGHEIGTISEEKTAVQGTGQDWKPSPSQ
Eagle KANK1           -----------L-----ISTLTSINSVMKYTSTEELRSLDLQKQCMERSTTSGATLEYIPHGQLANAHLTSNLRTLKLEQDVVPAQEERKTPLVEAVRGR
Chicken KANK1         -----------L-----ISTLTSINSVMKYASTEELRSLDLQKQCMEKSTASGATLEYIPHGQLANTHLTSNLRALKLEQDAVPAQEEKTTPLVEAARGR
Goose KANK1           -----------L-----ISTLTSINSVMKYASTEELRSLDLQKQCVERSTVSAGATLEYIPHGQLANAHLTSNLRALKLEQDIVPAQEERKTPLVEAARG
Turtle KANK1          -----------L-----INTLSSINSVMKYASTEELRNLDVQKQCLERSATPDANLEYIPHGQLASACLTSSSRTLKFEQEAGTIQEEKMIATVEKGWGK
Aligator KANK1        -----------L-----ISTLSSINSVMKYASTEELRSLDLAKQQCMDKSAITDAPLEYIPHGQLANAYLTSSSQTLKFEQDVGTVHEEKITVIDTGLGG
Spotted gar Kank1     ---------------L-MNTLSSINSVMKYSSTEELRTMEIFKLCPDSASAITDAGQSSNVSGACTGVNSTAHSDIRTQESEAQLREPLPSSATIDGLSK
Herring Kank1         -----------L-----VNTLSSINSVMKYGSVEELSAADIIKLAPDGSVSGDQQVVSRVSSQSLTLRSEVIQTESMSSAVTVTDETRQAEQKKRMDQLM
Zebrafish Kank1a      -----------L-----VTTLSSINSVWKYGSLEELCNLDLLRPQENSNHTVKETQMVSAVHSHSSSLRSEVTRIESVCTDDKHEQSTAAQKSRMDQQMS
Cave fish Kank1a      ---------------L-VTTLSSINSVMKYGSIEDLHALDLHKLQTDSGNVRTDTQIASSQSVGVRTEVTRTEKVCVEDPVTHEQSAAAQKSRMDQQMST
Stickleback Kank1a    -----------L-----ASTLSSINSVMKCGSAEDVLQLQRC------DSLSQASGQQLCAGGRSQVEAPPADRAAQQQVSAAEQKSRMDLQMSTALHGT
Medaka Kank1a         -----------L-----VSTLSSINSVMKYGSAEDIRKLQADPTPEGALLLSALQTDPSAVQTAADASPQQQISAAEQKRCMDLQMSTALHGQPSD----
Fugu Kank1a           -----------L-----VSTLSSINTVMKSGSTEDILKLQSDSVNLNKENVQNYSYSEYCALIFSYYMSQLRSEKTTSEPNSAEPPKNSPAQQQFSASTA
Tetradon Kank1a       -----------L-----VSTLSSINTVMRYGSSEDILKLQSDPASLKGKVRNEVHFICKKAVGLGIMIVSGSNTASTLLICKGLKEASVDEFQADTTNAV
Tilapia Kank1a        -----------L-----VSTLSSINSVMKYGSAEDILKLQAESKEESSQQLSSVRAECVEKSPDLTSADTPANTPSQQQISAAEQKSRMDLQMSTALHG-
Amazon molly Kank1a   -----------L-----VSTLSSINSVMKYGSAENILALHSDSCRDRSLMLPLPDAEAAERPENSPQQISAAEQKSRMDLQMSTALHGQPGS--------
Mouse KANK3           ----------------------------------------------------------------------------------------------------
Rat KANK3             ----------------------------------------------------------------------------------------------------
Dog KANK3             ----------------------------------------------------------------------------------------------------
Human KANK3           ----------------------------------------------------------------------------------------------------
Lamprey               -----------LSPVQ-VASVSGTSQCSTLASPADAGSSSV-----------------------------------------------------------
Coelacanth KANK3      -----------VIGNI-SSTLQGALNKKETDNGRDHTPVLSPP---------------------------------------------------------
Frog Kank3            -----------------VAEEDTSMANFGN----------------------------------------------------------------------
Turtle KANK3          -----------S------------ATPERPLEKDSNAPLSPG----------------------------------------------------------
Aligator KANK3        -----------------TGMQECVPTALEVTWENDSRAPLSTG---------------------------------------------------------
Eagle KANK3           A---------------------------------------------------------------------------------------------------
Chicken KANK3         ----------------------------------------------------------------------------------------------------
Goose KANK3           ----------------------------------------------------------------------------------------------------
Spotted gar Kank3     ----------------------------------------------------------------------------------------------------
Zebrafish Kank3       ----------------------------------------------------------------------------------------------------
Cavefish Kank3        ----------------------------------------------------------------------------------------------------
Herring Kank3         ----------------------------------------------------------------------------------------------------
Stickleback Kank3     ----------------------------------------------------------------------------------------------------
Fugu Kank3            ----------------------------------------------------------------------------------------------------
Tetradon Kank3        ----------------------------------------------------------------------------------------------------
Medaka Kank3          ----------------------------EEGSSEETSASESK----------------------------------------------------------
Tilapia Kank3         ----------------------------------------------------------------------------------------------------
Amazon molly Kank3    ----------------------------KTGEQEEETRTSDPK---------------------------------------------------------
Platyfish Kank3       ----------------------------------------------------------------------------------------------------

                               2410      2420      2430      2440      2450      2460      2470      2480      2490      2500
                      ....|....|....|....|....|....|....|....|....|....|....|....|....|....|....|....|....|....|....|....|
Hydra                 -------------------------------------------------------LQAMRDSMHNFAS-GMK-----RSTVSRSLNLES----C--DDS-
Trichoplax            ----------------------------------------------------------------------------------------------------
Aplasia               KRTTRSGDKVHVQETKTVEGPGGKTSTTVTETEDGKPVSSTRLQESESGSRTASSSVEAPASSQMTSL-SSSSTSSSSSSSALSSSFSSSSRQA--SGT-
Snail                 D------------------------------------------------------TVIVQETKTVEGP-DGKSVTTVTETTKDGLKIVQQYDNA--LQGP
Fruitfly              -------------------------------------------------------VSSFQASEPLPES-QSF--IRQPQKDSLDLSLLGEDELI-VR---
Hookworm              -------------------------------------------------------LTPLRSELRSLGR-WPR--------SNTQVPYSP-----------
C.elegance            -------------------------------------------------------LTPLRSDMRQLGNRWNRNQVTTASNTTPHLPYSG-------ATS-
Tunicate              -------------------------------------------------------PADLKSILKTPDT---------PVGEKKFLRF-------------
Spider                -------------------------------------------------------IPKLKLNIRSSEV------PSETKKASFRTKFTKTQKHT--TKS-
Lingula               TTVEESPDGVTTIRTEKSSYSSSSSSNRMGGDGGLNITGGGMGREQHAML-----SGLMDSGISMSEG-TGL---PAETVSTKSFSYSG-------TGGL
```

```
Lancelet              ENKHALSPGSSPAISN---------------------------------------LRPLRSCLKGKHS-TKK------VNIKKEIQFVG----V--NGN-
Medaka Kank4b         -------------------------------------------------------SETLRSEVPNERL-TAKGNLSDSDCKIQSLHARR-----------
Amazon molly Kank4b   -------------------------------------------------------LKKLQSEDANEGV-QMS--TKGSVVGQDNVKLCGEASVV--PRG-
Platyfish Kank4b      -------------------------------------------------------LKKLQSVDANEGV-QMS--TKGSVIGQDNGKLCGEASVV--PRG-
Stickleback Kank4b    --------------------------------------------------------------MERRSG----------------------------SGI-
Fugu Kank4b           -------------------------------------------------------DGRARSSKHRHSG-------GDNDGKNAVGQYRG-------HQS-
Tetraon Kank4b        ------------------------------------------------------------------------------------------------DGG-
Cave fish Kank4b      -------------------------------------------------------KESSGRVLSSEHL-PPR----VCSIQEQLVTLVS----L--LSL-
Medaka Kank4a         -------------------------------------------------------AGRLKSIMKKNGA-ADK---QGNKGAKKNLKFVG----V--NGG-
Fugu Kank4a           -------------------------------------------------------SPGLKSIMKKEGA-ADK---QGNKRAKKNLKFVG----V--NGG-
Tetradon Kank4a       -------------------------------------------------------SPGLKSIMKKDGV-ADK---QGNKRAKKNLKFVG----V--NGG-
Stickleback Kank4a    -------------------------------------------------------HLCLKSIMKKNGV-ADK---QGNKGAKKNLKFVG----V--NGG-
Tilapia Kank4a        -------------------------------------------------------SLGLKSIMKKNGA-AGK---QGNKGAKKNLKFVG----V--NGG-
Platyfish Kank4a      -------------------------------------------------------EQGLKSIMKKNGV-ADK---QGSKGAKKNLKFVG----V--NGG-
Amazon Molly Kank4a   -------------------------------------------------------EQGLKSIMKKNGV-ADK---QGSKGAKKNLKFVG----V--NGG-
Herring Kank4         -------------------------------------------------------QSGLKSIMKKDGC-PQK---PGNGAAKKNLKFVG----V--NGG-
Zebradish Kank4       -------------------------------------------------------QSGLKSIMKKNDC-PDK---QGNGGAKKNLKFVG----V--NGG-
Cave fish Kank4a      -------------------------------------------------------QSGLKSIMKKNDY------PDKQGGVKKNLKFVG----V--NGG-
Mouse KANK4           -------------------------------------------------------STSLKSIMKKKDY-GFR---AGGNGTKKNLQFVG----V--NGG-
Rat KANK4             -------------------------------------------------------STSLKSIMKKKDY-GFR---AGGNGTKKNLQFVG----V--NGG-
Dog KANK4             -------------------------------------------------------STSLKSIMKKKDY-GFR---AGGNGTKKNLQFVG----V--NGG-
Human KANK4           -------------------------------------------------------STSLKSIMKKKDY-GFR---AGGNGTKKNLQFVG----V--NGG-
Eagle KANK4           -------------------------------------------------------TTSLKSIMKKKGY-GFH---AGGNGTKKNLQFVG----V--NGG-
Chicken KANK4         -------------------------------------------------------TTSLKSIMKKKGY-GSH---AGGNGTKKNLQFVG----V--NGG-
Goose KANK4           -------------------------------------------------------TTSLKSIMKKKGY-GFH---AGGNGTKKNLQFVG----V--NGG-
Anole lizard KANK4    -------------------------------------------------------STSLKSIMKKKET-GYH---AGGNGTKKNLQFVG----V--NGG-
Turtle KANK4          -------------------------------------------------------TTSLKSIMKKKGY-GFH---AGGNGTKKNLQFVG----V--NGG-
Aligator KANK4        -------------------------------------------------------TTSLKSIMKKKGH-GFH---AGGNGTKKNLQFVG----V--NGG-
Frog Kank2            -------------------------------------------------------TDPTKEQDKGKRN-------AQEDPQPKPAQFPR----L--NGE-
Mouse KANK2           -------------------------------------------------------AGRASLATKRKED------PADPEVNQRNLQFVG----V--NGG-
Rat KANK2             -------------------------------------------------------SLGVQSALKRKEV------PADPDVHQRNLQFVG----I--NGG-
Dog KANK2             -------------------------------------------------------QVGVRSIMKRKEE------PADPAARRRSLQFVG----V--NGG-
Human KANK2           -------------------------------------------------------PAGVRSIMKRKEE------VADPTAHRRSLQFVG----V--NGG-
Aligator KANK2        -------------------------------------------------------QAEARASARTIGE-------DPEPPGGSSYHLGG----I--NGG-
Spotted gar Kank2     -------------------------------------------------------QSSLKSIMKRKDS-EAC---TDSPNTKKNLQFIG----V--NGG-
Cave fish Kank2       -------------------------------------------------------VQPIPATDSTQHT-PPH--STATPPEPSSVQSAATCTVL--EQT-
Herring Kank2         -------------------------------------------------------ASTSPPASEQTAP-QAK--PTDTSATQSDAQELT-----------
Zebrafish Kank2       -------------------------------------------------------EGDVQCVFQGCNP-----PSTATSLEQNSVQLSSATQQSKATDSN
Tetradon Kank2        -------------------------------------------------------PPSLRSIMKRKAE-----GEPGSPSTKKNLQFIG----V--NGG-
Fugu Kank2            -------------------------------------------------------SSTLRSIMKRKAE-----GEPGSPTTKKNLQFIG----V--NGG-
Medaka Kank2          -------------------------------------------------------QSCLRSIMKRKAE-----GEPGSPSTKKNLQFIG----V--NGG-
Sticklebakc Kank2     -------------------------------------------------------QSSLRSIMKRKAE-----GEPGSPSTKKNLQFIG----V--NGG-
Tilapia Kank2         -------------------------------------------------------QLSLRSIMKRKAE-----GEPGSPSTKKNLQFIG----V--NGGI
Amazon molly Kank2    -------------------------------------------------------TDSERKCASHTST-TNSTSTPPAKDSASPVDQIADKHTV--TQE-
Platyfish Kank2       -------------------------------------------------------QSSLRSIMKRKAE-----GEPGSPSTKKNLQFIG----V--NGG-
Coelacanth KANK2      -------------------------------------------------------ETSLKSIMKRKDD-TAF----LDPASRKNLQFVG----V--NGG-
Anole lizard KANK2    -------------------------------------------------------TAGIRSIMKRKEE------HTEIVASKKSLHFVG----I--NGG-
Turtle KANK2          -------------------------------------------------------QGGVRSGTKRQEE-------APELVGKKSLQFVG----I--NGG-
Tetradon Kank1b       -------------------------------------------------------SSLLPSGMKKQKG------DQGCSSTRKNMKLLR----L--NPP-
Medaka Kank1b         -------------------------------------------------------TNMCKSVIKKPSG------DQSCSSNRKNMKFVE----A-ATGL-
Amazon molly Kank1b   -------------------------------------------------------AAKPWSAKKKQGA------AQCHSSNRKSMKFMK----V--SAG-
Platyfish Kank1b      -------------------------------------------------------ASKPWSAMKKQGT------DQCHSSNRKSMKFMK----V--SAG-
Frog Kank4            -------------------------------------------------------RSSLKSIMKKKNI-NSR-MGSSEVQAKKNLQFVG----V--NGG-
Zebrafish Kank1b      -------------------------------------------------------TGSKPDLILQSQF--------------------------------
Tilapia Kank1b        -------------------------------------------------------TGSKPDLILQSQF--------------------------------
Fugu Kank1b           -------------------------------------------------------TGSKPDLILQSQF--------------------------------
Cavefish Kank1b       -------------------------------------------------------LSSPRSIIKRQDI-SPR------AECRRTVKCLA--------AG-
```

```
Spotted gar Kank4      -------------------------------------------------------PSALKSIMKKKDC-SVR---LGSSGAKKNLKFVG----V--NGG-
Coelacanth KANK4      -------------------------------------------------------TTMLKSIMKKKNC------NPEDNGTKKNLQFVG----I--NGG-
Frog Kank1             ACIPASPAAEGETECVSASG-----------------------------------QTTLKSIMKRKDE------KKESTNTKKNLQFVG----I--NGG-
Shark Kank1            LTPQTDSPSQPATSTICAVSPSS--------------------------------TGILKSIMKKKDS-PKK---HETNGTKKNLQFVG----V--NGG-
Coelacanth KANK1      DSKTFSPVEDMTSQLNVIDQPMASLYVSSCS------------------------PGALKSIMKKKDG------RQDSNGTKKNLQFVG----I--NGG-
Mouse KANK1            LPPVNLTDDQIATGLYVCPNN----------------------------------ENTLKSIMKKSDG------NKDSNGAKKNLQFIG----I--NGG-
Rat KANK1              PVNLTDDQIATGLYVCPNN------------------------------------ENTLKSIMKKSDG------NKDSNGAKKNLQFIG----V--NGG-
Dog KANK1              LSPVNLTDDQIAAGLYVCTNS----------------------------------ENTLKSIMKKKDG------NRDSNGAKKNLQFVG----I--NGG-
Human KANK1            LSPVNLTDDQIAAGLYACTNN----------------------------------ESTLKSIMKKKDG------NKDSNGAKKNLQFVG----I--NGG-
Anole lizard KANK1     DKTMSPINLTDDQLASGLYVCTS--------------------------------DSMLKSIMKKKDG-------KKEENAKKNLQFVG----I--NGG-
Eagle KANK1            KSFSSQDKTLTPINLTDDQLASGLYVCTNS-------------------------ENTLKSIMKKRDG------KKDMSNTKKNLQFVG----I--NGG-
Chicken KANK1          KSFSSQDKTLAAINLTDDQLASGLYVCSNS-------------------------ENTLKSIMKKRDG------KKDLSNTKKNLQFVG----I--NGG-
Goose KANK1            RKSFSPQDKTLAAINLTDDQLASGLYVCSNN------------------------ENTLKSIMKKRDG------KKDLSNTKKNLQFVG----I--NGG-
Turtle KANK1           KSLSSQDNAIPPINLTDDQLASGLYVCANN-------------------------ESTLKSIMKKRDG------EKDLNGAKKNLQFVG----I--NGG-
Aligator KANK1         RTSSSEDDAVPPLNLTDDQLASGLYVCTNN-------------------------ESTLKSIMKKRDG------KKDSNGTKKNLQFVG----I--NGG-
Spotted gar Kank1      DSTSSALRQSRTSQMTLMDQQMSSALHGQSCS-----------------------SNTLKSIMKKKDG------RQGSNSTKKNLQFVG----V--NGG-
Herring Kank1          STALHGS------------------------------------------------QNTLKSIMKKKDG------RQGSNGTKKNLQFVG----V--NGG-
Zebrafish Kank1a       TALHGS-------------------------------------------------HSTLKSIMKKDGH-------QGHNGTKKNLQFVG----V--NGG-
Cave fish Kank1a       ALHGG--------------------------------------------------QSTLKSIMKKKDG------RQGTNGTKKNLQFVG----V--NGG-
Stickleback Kank1a     AAATTGGGGGRCLIP----------------------------------------QSTLKSIMKKKDG------RPDSNGTKKNLQFVG----V--NGG-
Medaka Kank1a          -------------------------------------------------------QSTLKSIMKKKDG------RPESNGTKKNLQFVG----V--NGG-
Fugu Kank1a            QKSSMDLQMSTALHGQPSS------------------------------------QSALKSIMKKKDG------KADANSTKKNLQFVG----V--NGG-
Tetradon Kank1a        LVLPDELSRGTHILPSS--------------------------------------QSTLKSIMKKKDG------KPDSNGTKKNLQFVG----V--NGG-
Tilapia Kank1a         -------------------------------------------------------QSTLKSIMKKKDG------RADSNGTKKNLQFVG----V--NGG-
Amazon molly Kank1a    -------------------------------------------------------QNTLKSIMKKKDG------RPDASGTKKNLQFVG----V--NGG-
Mouse KANK3            -------------------------------------------------------VGILKSIMKKKDG-IPG---AQSSQGPKSLQFVG----V-LNGE-
Rat KANK3              -------------------------------------------------------AGILKSIMKKKDG-TPG---AQSSQGPKSLQFVG----V-LNGE-
Dog KANK3              -------------------------------------------------------AGILKSIMKRRDG-TPG---AQPSPGPKSLQFVG----V-LNGE-
Human KANK3            -------------------------------------------------------AGILKSIMKKRDG----TPGAQPSSGPKSLQFVG----V-LNGE-
Lamprey                -------------------------------------------------------SPTLKSIMKKKDG-AGSAGGDPSSGTKKSLQFVG----V--NGG-
Coelacanth KANK3       -------------------------------------------------------VGALKSIMKKDGT-S----NKETNRGKKSLQFVG----V-LNGE-
Frog Kank3             -------------------------------------------------------RGALKSIMKNKDG-ASK---SQINREKKSLQFVG----I-LNGE-
Turtle KANK3           -------------------------------------------------------AGALKSIMKKRDG-FPK---SEAEGGRKSLQFVG----V--LNG-
Aligator KANK3         -------------------------------------------------------TGSLKSIMKKRDG-LPK---NEAEGGRKSLQFVG----V-LNGE-
Eagle KANK3            -------------------------------------------------------SGALKSIMKKRDG-PPR---SEADGSKKSLQFVG----V-LNGE-
Chicken KANK3          -------------------------------------------------------ARALKSIMKKRDG-PPR---SEAEGSKKSLQFVG----V-LNGE-
Goose KANK3            -------------------------------------------------------AGALKSIMKKRDG-APR---SEAEGSKKSLQFVG----V-LNGE-
Spotted gar Kank3      -------------------------------------------------------RSVLKSIMKRKDG-S----TQREPGSRKSLQFVG----V-LNGG-
Zebrafish Kank3        -------------------------------------------------------AGPLKSIMKRKDG-SGA--GAASSSGKKSLQFVG----I-LNGG-
Cavefish Kank3         -------------------------------------------------------TGPLKSIMKRKDG-SGS--GVACSGGKKSLKFVG----I-LNGG-
Herring Kank3          -------------------------------------------------------MGTLKSIMKRKDG-SSS--SQTRNTGKKSLQFVG----I-LNGG-
Stickleback Kank3      -------------------------------------------------------AGMLKSIMKRKDG-------NNSGEKKKSLQFVG----I-LNGG-
Fugu Kank3             -------------------------------------------------------TGILKSIMKRKDG-SSS--DENRTSSKKSLQFVG----I-LNGGA
Tetradon Kank3         -------------------------------------------------------AGVLKSIMKRKDG-SSA--EQSRTSSKKSLQFVG----I-LNGG-
Medaka Kank3           -------------------------------------------------------TGMLKSIMKRKDG-STS--SDSNSAGKKSLQFVG----I-LNGG-
Tilapia Kank3          -------------------------------------------------------TGLLKSIMKRKDG-NNA--GENRSSGKKSLQFVG----I-LNGG-
Amazon molly Kank3     -------------------------------------------------------TGMLKSIMKRKDG------SNSSENRKKSLQFVG----I-LNGG-
Platyfish Kank3        -------------------------------------------------------AGMLKSIMKRKDG------SNSSENRKKSLQFVG----I-LNGG-

                                2510      2520      2530      2540      2550      2560      2570      2580      2590      2600
                       ....|....|....|....|....|....|....|....|....|....|....|....|....|....|....|....|....|....|....|....|
Hydra                  ---------------------------FYKSKD-----DLE------------------------------------SPEDDYRAIMSDEEDDANGRGKA
Trichoplax             ---------------------------YDIATS-----SGL------------------------------------EKEMCNDSVTIANLSDHKDNVQA
Aplasia                ----------DSNLISSERDASALSGSFSSSSGGVGSSSMS------------------------------------YSETTISTVGSSSGVGGSSSEQQ
Snail                  HSNFFSSNGATENEMSSSTYSSS----YSSSRT-----DDS-------------------------------DVSVEQDTDSYTQISTDSDMTTSSGNYG
Fruitfly               ---------------------------EEKRVSISWSRDAS------------------------------------PAPLMTSSGTTKSESPEQEPAMS
Hookworm               ---------------------------YKTGSE-----SDE------------------------------------DSDDSEGSYDTHEDGVDHEES--
```

```
C.elegance            ---------------------------QEDVSD--------------------------------------------SDAESDCSAHSDATFEVNDDVFD
Tunicate              ---------------------------AEGVKS-----DDS------------------------------------SSEEDYSSSDEEEEQSTSADETK
Spider                ---------------------------IHVTSS-----DDA------------------------------------VVDSVVNRFDSDDLVTKRNELNE
Lingula               REALNMYKNIQDNERESMSDGSGRVTSFQSSSTTTSSSHESPVKEGVTRIAVTHAPETTGVREVKSVVETDIDADITTGETTTTSSSSSSTSASRQTSTS
Lancelet              -G-------------------------HDESSS-----DDS------------------------------------SSEESSEDETDDEDSSSSESESS
Medaka Kank4b         ---------------------------VDAAQT-----DAK------------------------------------SVQTGMAAKQQPEKPVTPVERKD
Amazon molly Kank4b   ---------------------------MEATQV-----DVH------------------------------------PAKRRVQTDKSKQTCPEGHATPV
Platyfish Kank4b      ---------------------------MEATQV-----DVH------------------------------------PAKRRIQTDESKQTCPEGHATPV
Stickleback Kank4b    -----------------------------TTRG-------------------------------------------------------------------
Fugu Kank4b           ---------------------------EERESSVGPGAMAA------------------------------------AQVEANRSGEEAQGQIRSGGAGP
Tetraon Kank4b        --------------------------------------------------------------------------------AKPKEAEAPERTAKLKPHPP
Cave fish Kank4b      ---------------------------YVSPAG-----ELQ------------------------------------AQQPAQTSKLEDPISVLHKSRVE
Medaka Kank4a         ---------------------------YETTSS-----EES------------------------------------SGDEKSKVEVEEVPADKGDASAD
Fugu Kank4a           ---------------------------YESTSS-----EES------------------------------------SGEEKPKAEVEEENSSEPEEETE
Tetradon Kank4a       ---------------------------YESTSS-----EES------------------------------------SGEDKPKAAVEDEDSSGAEEEKK
Stickleback Kank4a    ---------------------------YESTSS-----EES------------------------------------SGDEKSKVEVEEEKGSSSPEEKD
Tilapia Kank4a        ---------------------------YETTSS-----EES------------------------------------SGDEKSNVEVEEEDSSEPEVEKE
Platyfish Kank4a      ---------------------------YETTSS-----EDS------------------------------------SADEKSRAEVEEEDKSEVVVEKE
Amazon Molly Kank4a   ---------------------------YETTSS-----EDS------------------------------------SGDEKSRVEVEEEDKSEVVVEKE
Herring Kank4         ---------------------------YESTSS-----EES------------------------------------SGAENQEEQEQEQEDSSEPEEDK
Zebradish Kank4       ---------------------------YESTSS-----EDS------------------------------------SGEENQDAVEEVESSEPEVEQGE
Cave fish Kank4a      ---------------------------YESTSS-----EES------------------------------------SGEENEDEGKEEEEEVDSSEPEE
Mouse KANK4           ---------------------------YETTSS-----EET------------------------------------SGEDSSPEDLSDSETEKKQDCSE
Rat KANK4             ---------------------------YETTSS-----EDT------------------------------------SGEDSSPEDLSDSETEKKQDCSE
Dog KANK4             ---------------------------YETTSS-----EET------------------------------------SGEDSSPEDLSDSEAEKKCDGPE
Human KANK4           ---------------------------YETTSS-----EET------------------------------------SGEDSTPEDLSDSEAEKKCDGPD
Eagle KANK4           ---------------------------YETTSS-----EDT------------------------------------SCEDSPSDGDVESETERRAEDLE
Chicken KANK4         ---------------------------YETTSS-----EDT------------------------------------SCEDSPSDGDMESDTERGAGGLE
Goose KANK4           ---------------------------YETTSS-----EDT------------------------------------SCEDSPSEGDAESETERRAGGLE
Anole lizard KANK4    ---------------------------YETTSS-----EDS------------------------------------SCEETSSEGNSDSETERKCDNEE
Turtle KANK4          ---------------------------YETTSS-----EDT------------------------------------SCEDSSSEGNSDSEMERKYDTLE
Aligator KANK4        ---------------------------YETTSS-----EDT------------------------------------SCEDSSSEGNSDCETERKYESLA
Frog Kank2            ---------------------------MDSASS-----EDS-----------------------------------GTMENPSDSESTESEYHEASEALA
Mouse KANK2           ---------------------------YESPSE-----DSS------------------------------------TAENSEHESTENEGPEPPARVLS
Rat KANK2             ---------------------------YESSSE-----DSS------------------------------------TAENSEHESTENEAPEPPVRVLS
Dog KANK2             ---------------------------YESSSE-----DSS------------------------------------TAENFSDNESTENEAPEPEERVP
Human KANK2           ---------------------------YESSSE-----DSS------------------------------------TAENISDNDSTENEAPEPRERVP
Aligator KANK2        ---------------------------YESASS-----DSS------------------------------------TAENSEAESTESEYHEASEGLPA
Spotted gar Kank2     ---------------------------YESTSS-----DSS------------------------------------SESSDSSSESEYHEANERLPPTQ
Cave fish Kank2       ---------------------------LNQPKT-----TDS------------------------------------SSQQVSHQSEAFSPPPQQPTAPP
Herring Kank2         ---------------------------TQTTSP-----TPQ------------------------------------QQASQSKTTDLTPQQGASKSPAT
Zebrafish Kank2       VQPD-----------------------LAQTDV-----SDF------------------------------------AAQQTTTQTQFTSAKPQQEASQS
Tetradon Kank2        ---------------------------YESTSS-----DDS------------------------------------SSQSSDEGSDSSEYHEPPATDCP
Fugu Kank2            ---------------------------YESTSS-----DDS------------------------------------SSESSDEGSDSSEYHEAREKLPE
Medaka Kank2          ---------------------------YESSSS-----DES------------------------------------SSESSDEGSDSSEYHEAREKLPA
Sticklebakc Kank2     ---------------------------YESTSS-----DDS------------------------------------SSESSDEGSDSSEYHEAREKLPA
Tilapia Kank2         S--------------------------YESTSS-----EDS------------------------------------SSESSDEGSDSSEYHEAREKLPE
Amazon molly Kank2    ---------------------------ITSTSP--------------------------------------------NSESAPIAIKCSSTCITKSAEVN
Platyfish Kank2       ---------------------------YESTSS-----DDS------------------------------------SSESSDDGSDSSEYHEEITSTSP
Coelacanth KANK2      ---------------------------YESTSS------DT------------------------------------SSEDSSSENESSESEYHEANEGP
Anole lizard KANK2    ---------------------------YESTSS-----EDS------------------------------------STAENVSDNESTESEYHEASEGL
Turtle KANK2          ---------------------------YESTSS-----GSS------------------------------------TAENSSDNESTESEYHEASEGPP
Tetradon Kank1b       ---------------------------YKPYAV-----EKA------------------------------------SSEEEEKTERRRERSKVDLPRRP
Medaka Kank1b         --------------------------------------DFM------------------------------------LVQEQQERGENETFGKFSPQGHQ
Amazon molly Kank1b   ---------------------------LEPTSA-----YEH--------------------------------------EEREGRGSDTFRKVSQEGWQA
Platyfish Kank1b      ---------------------------LKPTSA-------F------------------------------------EHEEREGRGSDTFGKVSQDGWQA
Frog Kank4            ---------------------------YETTSS-----EDS------------------------------------SSSEEFCGTGKNDVSDSGEAQAS
```

```
Zebrafish Kank1b       ---------------------------Y----------SDE-----------------------------------------------------------
Tilapia Kank1b         ---------------------------Y----------SDE-----------------------------------------------------------
Fugu Kank1b            ---------------------------Y----------SDE-----------------------------------------------------------
Cavefish Kank1b        ---------------------------FERVSV---------------------------------------------SEDSFSEGADSEDEENRKRKME
Spotted gar Kank4      ---------------------------YETTSS-----EES------------------------------------SGEDEENDSSEEREDGGVEAAQS
Coelacanth KANK4       ---------------------------YETTSS-----EDS------------------------------------SGEESSSDDNSGCDSVEKCDSSV
Frog Kank1             ---------------------------YETTSS-----DES------------------------------------SSDDSSSSESDDECEEEESFNEE
Shark Kank1            ---------------------------YETTSS-----DDS------------------------------------SSDESSSSESEEELEEPDSCKDE
Coelacanth KANK1       ---------------------------YETTSS-----DDS------------------------------------SSEESSSSETDDECDGNGYNVEE
Mouse KANK1            ---------------------------YETTSS-----DES------------------------------------SSDGSSSSESDDECDTIGYPPEE
Rat KANK1              ---------------------------YETTSS-----DDS------------------------------------SSGGSSSSESDDECDTNGYPPEE
Dog KANK1              ---------------------------YETTSS-----DDS------------------------------------SSDESSSSESDDECDVIEFPPEE
Human KANK1            ---------------------------YETTSS-----DDS------------------------------------SSDESSSSESDDECDVIEYPLEE
Anole lizard KANK1     ---------------------------YETTSS-----DDS------------------------------------SSEESSSSESDDECVENEYGQIE
Eagle KANK1            ---------------------------YETTSS-----DDS------------------------------------SSEESSSSDSEEDCEGHEYPHDR
Chicken KANK1          ---------------------------YETTSS-----DDS------------------------------------SSEESSSSDSEEECEGHAYPHDH
Goose KANK1            ---------------------------YETTSS-----DDS------------------------------------SSEESSSSDSEEESEGHAYPHGH
Turtle KANK1           ---------------------------YETTSS-----DES------------------------------------SSEESSSSESDDECEGNEYSLHK
Aligator KANK1         ---------------------------YETTSS-----DES------------------------------------SSEESSSESDDECEGNEYSHKEE
Spotted gar Kank1      ---------------------------YETTSS-----DES------------------------------------SSEESSTSESEDECEGNEYPSEE
Herring Kank1          ---------------------------YETTSS-----DDS------------------------------------SSEESSSSGSDDEEDEEEEEVEG
Zebrafish Kank1a       ---------------------------YETTSS-----DDS------------------------------------SSEESSSSGSEDEGDDKNKTVNV
Cave fish Kank1a       ---------------------------YETTSS-----DDS------------------------------------SSEESSSDEGSEEDEEEQERVGD
Stickleback Kank1a     ---------------------------YETTSS-----DDS------------------------------------SSEDSSSSGSDEEEDEDEKAFEG
Medaka Kank1a          ---------------------------YETTSS-----DES------------------------------------SSEDSSSSGSDDEEEEEEEKAGG
Fugu Kank1a            ---------------------------YETTSS-----DDS------------------------------------SSEDSSSSGSDEEEEEEEKEWED
Tetradon Kank1a        ---------------------------YETTSS-----DDS------------------------------------SSEDSSSTVSNSGFFQ-------
Tilapia Kank1a         ---------------------------YETTSS-----DDS------------------------------------SSEDSSSSGSDEEEEDEEEGYGG
Amazon molly Kank1a    ---------------------------YETTSS-----DES------------------------------------SSEDSSSSGSEEEEGDEEKEEEY
Mouse KANK3            ---------------------------YESSSS-----EDG------------------------------------NSDDEDGVAEHPRSSSSGSDDSS
Rat KANK3              ---------------------------YESSSS-----EDA------------------------------------SNSDDEDDVAELPRSSSSGSGDD
Dog KANK3              ---------------------------YESSSS-----EDD------------------------------------SDSDSEDGAAEPPRSSSSGDDSG
Human KANK3            ---------------------------YESSSS-----EDA------------------------------------SDSDGDSENGGAEPPGSSSGSGD
Lamprey                ---------------------------YETTSS-----EES------------------------------------SSESSSSGSEDEDDDDDDDEEEE
Coelacanth KANK3       ---------------------------YETTSS-----EES------------------------------------SENEENSSDKVSGDSSDTDVENF
Frog Kank3             ---------------------------YESTSS-----EES------------------------------------SDNEGDSSSDKVSEDSSDSEALN
Turtle KANK3           -E-------------------------YESTSS-----EEE------------------------------------EEDSEDSSSEKISPTSSDSDAED
Aligator KANK3         ---------------------------YESTSS-----EEE------------------------------------GGDSSSEKISPNSSDSDAEGGTD
Eagle KANK3            ---------------------------YESTSS-----EEE------------------------------------EEEEEGDSSSEKASADSSNSEEQ
Chicken KANK3          ---------------------------YESTSS-----EEE------------------------------------EEEEEEEEEGDSSSEKASADSSD
Goose KANK3            ---------------------------YESTSS-----EEE------------------------------------EEDGDSSSEKASADSSDSEEQGG
Spotted gar Kank3      ---------------------------YESTSS-----EEE------------------------------------EDGEEEEGTSSGGSGGGGSDSSE
Zebrafish Kank3        ---------------------------YESTSS-----EEE------------------------------------EEEEESSSDGSEAGACSDSSVEE
Cavefish Kank3         ---------------------------YESSSS-----EEE------------------------------------EEEEEDEEEGEEESADGACSDSS
Herring Kank3          ---------------------------YESTSS-----EEE------------------------------------DEEEDEEGSSSDGSGAGESSSSE
Stickleback Kank3      ---------------------------YESTSR--------------------------------------------TSSGESGEGECLDSTEEDEAALE
Fugu Kank3             SPR------------------------YESTSS-----EED------------------------------------EEEDGSSSDESGEGECLDSTEDE
Tetradon Kank3         ---------------------------YESTSS--------------------------------------------EKEEEEEDGSSSDESGDGECLDS
Medaka Kank3           ---------------------------YESTSS-----EEE------------------------------------EEEEEDGSSSGESGEGELLDSTE
Tilapia Kank3          ---------------------------YESTSS-----EEE------------------------------------EEEEEEDGSSSGESVEGECLDST
Amazon molly Kank3     ---------------------------YESTSS-----EEE------------------------------------EEDEEEEVGSSSGESGEGECLDS
Platyfish Kank3        ---------------------------YESTSS-----EEE------------------------------------EEEEEEEEEDGSSSGESGEGECL

                                2610      2620      2630      2640      2650      2660      2670      2680      2690      2700
                       ....|....|....|....|....|....|....|....|....|....|....|....|....|....|....|....|....|....|....|....|
Hydra                  DV--------------------------------------------------------------------------------------------------
Trichoplax             PM--------------------------------------------DLPQDQIIINGNSD---------------------------------------S
```

```
Aplasia                 QQEQEMMSYGSLDRRTGQLKSIMKHTKAELEAASGKKGITFAESVTGGTGSSSDDDSDSTASFEEGSYDSHQGAVVYRCQDDEAIAQGLPGAQMFDQ--N
Snail                   SLDRATGKLKSIMKQSKSEAERRDKKTGIRFAESVTGGTGS-----SSEEDDDTSDSDSTTSFDEGSYDSQQGEVVYKCKDDEVIAQGRPGAQMFDQ--N
Fruitfly                SS--------------------SDSSPPIDVQIAQGARKKS-----STPLKSSQSEGSQV-------------------TVIEQPATKAQLHQLAQADSE
Hookworm                ----------------------------------------------------------------------------------------------------
C.elegance              AA--------------------------------------------------------------------------------------------------
Tunicate                EE-------------LKELMREIEKENDVKVKEEDMKNMVC-----DVGKEVVDQHKENS--------KKEEISLVAAMAKTVKTSVIEERIVGDGAEDE
Spider                  NK----------------------------AKSADDTGSFS-----ECSENLPNFLNLPV---------------------------------------P
Lingula                 STNGANGTIRSIMKKSSYKSSKGSSKKGISFSDDVTGGYAS-----SSGEEESEPESDES--FDEGSYDGRHMTITYTCKDDEKIADGAPGTGMFDQ--N
Lancelet                DE---------GGYNC--------ELGKVIKKGKEDQGKKD-----VKTISSSEIICEDI------------------KETDRL----------VSR--E
Medaka Kank4b           GQ------------------------RDGGAVTAGADGKEK-----KPPRQQKEGNSGSG----------------------------------------
Amazon molly Kank4b     GA----------------------------ISGQTDGGTVLLEAEGPEKQSKTLEDRTGT---------------------------------------N
Platyfish Kank4b        GA----------------------------ISGQTDGGTVLLEAEGPENKSKTLEDRTGS---------------------------------------N
Stickleback Kank4b      ----------------------------------------------------------------------------------------------------
Fugu Kank4b             QR-----------------------TDGGAKPEKADGPEKTAKLKPHPSREQMEGSSASG--------------------------------------CP
Tetraon Kank4b          RE--------------------------------------------QMEGSSASGCPSRG----------------------------------------
Cave fish Kank4b        SP-----------------------MQVDARERSGDGSDAV-----EYSTQPAELPEEES---------------------------------------D
Medaka Kank4a           QK-----------------------EVERGLMDPEEGHELL-----EEEEGFLGGGKLRL-------------------------------------GRK
Fugu Kank4a             KK--------------TESQP-----VEKPVEEAEAQQKEE-----DVPAAEGGASGAEQ------------------QSERGLMDPEVR---------H
Tetradon Kank4a         RK-------------------AEPQPAEKPEKEAEAQQKEE-----DVPAAEEGASAAEQ------------------RSDRGLL--------------D
Stickleback Kank4a      AE--------------------------VSTEGGGAVAAET-----EGERGLLDIEGSQE------------------LPEEKAE---------------
Tilapia Kank4a          PE-------------------------KEPEKQPEEGAEAQ-----RKDAEVPAEGGSAV------------------AQEKETERGLLDPESGQELLEE
Platyfish Kank4a        TD--------------------KESEESVGILVADAEVAAA-----APAPSAAAAEEEEG-----------------AVGEDKEVERGLLDLEGGHELLQ
Amazon Molly Kank4a     TD--------------------KESEGSVGILGADVEVAVA-----AAAPAAAAAEEEEG-----------------AVGEEKEVERGLLDLEGGHELLQ
Herring Kank4           DK----------------------EKEQGHEAGAEAQEEADSAGAQGEEEEEEEAGGPEG-----------------------------ATEPRGAEAFM
Zebradish Kank4         ED-----------------------SGAAQEEAAAAGEQSEGVQAEGTEGSEEPDVCQAS--------------------------------------MS
Cave fish Kank4a        QG-------------EEKENGAAQEEEEEDEKEAAATGEQADEAQAKGSECSEEPKDPEA-------------------------------------IID
Mouse KANK4             SR-----------------------EDRDLHPSCEAGQGVP-----EGTRNSGHTSDRGE---------------------------------------E
Rat KANK4               PR-----------------------EDRDMHLSCEAGQGVS-----KGTGNSGHMSEPRK---------------------------------------G
Dog KANK4               HR--------------------------RGKEAHNTCKAGQ-----GIPEGISSTGQESG------------------------------------PGGD
Human KANK4             HK-----------------------HVKDAHLTCEAGQGIP-----EGTCHAAQESGPGE---------------------------------------E
Eagle KANK4             PT--------------------------QAEAGGESEGSSS-----GTSSQERREDGDLQ------------------EPSAEAAPCAQ----------H
Chicken KANK4           PT-----------------------------QEQGGGGSEG-----ASFVTSVQETREDD---------------------------------------D
Goose KANK4             PT--------------------------QEEGGGESTGASS-----GTSSQERREDDDLQ------------------EPVAEAT--------------P
Anole lizard KANK4      HR-----------------------WREDGDEGDENASGTSQCVKHEDTDGKELEETLRG---------------------------------------S
Turtle KANK4            HR--------------------------QVKAARENKHASS-----GVSECTRHEDNEPQ------------------ELEEITM--------------A
Aligator KANK4          HR--------------------------QVRAAGDRDRASP-----GVSQCTRHKDEELQ------------------EREEPAM--------------A
Frog Kank2              GA---------------------QAELHSLAAGNSDTPKEP-----EREQPAPTAIVSPP---------------------------------------K
Mouse KANK2             PA-------------------ECPQLRPPGAAVATTSLEGP-----QLSQESQRVPAPEV-------------------------------------ASG
Rat KANK2               TA-------------------EGPQLRPLGPAVGKTSQDER-----QLSQESQRVPEAKV-------------------------------------APG
Dog KANK2               SV------------------VEAPQLRPSGTAAAKTSQEEC-----QLPRESQHVPVAEA-------------------------------------ASG
Human KANK2             SV------------------AEAPQLRPAGTAAAKTSRQEC-----QLSRESQHIPTAEG-------------------------------------ASG
Aligator KANK2          GG----------------------------AETPKATEEEA-----AAGAALSTTQGSND----------------------------------------
Spotted gar Kank2       DA--------------------PRAHSEEAAPGPLQEATATAFPPAPSEGAAAEQDRSTT--------------------------------------QE
Cave fish Kank2         ST-------------------------TVPAHTGASQSQTT-----DLTTQQEAIQSEAQ---------------ISLQSSDMSVQQGAAPPPPDAHAAN
Herring Kank2           DS------------------------SSQQHTSQSTATEPA-----TGNVCSASSANQES----------------------------------------
Zebrafish Kank2         KT-----------------------ADLTAQKGATHSTDGSAKQDIAISSTTKPAADTAT---------------------------------------P
Tetradon Kank2          PA-------------------AGGAPNPASTAAADVAKEQS-----ETTIRAEQKPAAES---------------------------------------S
Fugu Kank2              ST---------------------LQHQQLTHNRLSDGTGVPQQAAATQPSPHQPPAVDMG---------------------------------------Q
Medaka Kank2            VK-----------------------TSDATKQQDTAQSEASAASSQQDSKPAAERVPNNS---------------------------------------G
Sticklebakc Kank2       AA------------------TRHQQTPEITTPRPTDRSETT-----VPGSRSEPTPPDAT---------------------------------------A
Tilapia Kank2           ST--------------------VQHQQTIHNKDSKQEHLVSLKSVITPSSGTASSQNANQ---------------------------------------Q
Amazon molly Kank2      PL--------------------------SQTSTTSCQPESK-----AAPESVKHESSKPV----------------------------------------
Platyfish Kank2         NT----------------ESAPITIKCSSICITKSAEVNALSQMSTTSCQPESKAAPESV---------------------------------------K
Coelacanth KANK2        PA---------------------------GHAGQPAEQKPQ-----EMETSDTNNNEKED------------------SEGEKPTLVTAAAGESASSEEA
Anole lizard KANK2      PA-----------------------AQAAIAEPAKPSGTAATHPRIATEGGEIPTPAEGQ---------------------------------------S
Turtle KANK2            AG-------------------------QTETPRPPATAPQR-----VPAESDTATAGEPP---------------------------------------R
Tetradon Kank1b         KC-------------------------ARDHKRSQSNVSQL-----SSCFHSESANFLSV---------------------------------------T
```

```
Medaka Kank1b          AT---------------------------------KGKEGF-----SKRLKDTTKLHKQQ----------------------------------------
Amazon molly Kank1b    KK--------------------------------------------GKDGSNKGSKGSLK---------------------------------------L
Platyfish Kank1b       KK--------------------------------------------GKDGSNKGSKGSLK--------------------------------------LP
Frog Kank4             SD-------------------------DKIETVLMPVSEVK-----EIAQEDPEPMVPSQ---------------------------------------Q
Zebrafish Kank1b       ---------------------------------------------------------------------------------------------------Q
Tilapia Kank1b         ---------------------------------------------------------------------------------------------------Q
Fugu Kank1b            ---------------------------------------------------------------------------------------------------Q
Cavefish Kank1b        KQ--------------------------------------------LEESSRTSMRASKM---------------------------------------E
Spotted gar Kank4      TN--------------------------SEESERTGGHSQV-----TIWSHCGLNAFPGD---------------------------------------D
Coelacanth KANK4       EN----------------------HLQAASNGSEAVSKTIQ-----GKRVPDTEPNELDV------------------NATDGPQ--------------Q
Frog Kank1             SG-----------------------ELYHGVASVIPGSPSN-----LEEKQLNEKEPEKV---------------------------------------E
Shark Kank1            FE---------------------------GNSEESSEGPVV-----RQPETEKGVSGESE------------------ELDKEVDKV------------E
Coelacanth KANK1       DG-----------------------HVEQMAGELDGYSNKGILPVSESVEEMKEKEAETG---------------------------------------E
Mouse KANK1            EE-------------------------EEEEKDHDTRGMAEGHHAVNIEGFKSARVEDEV-----------------QVPECEPEKE------------E
Rat KANK1              EE------------------EEEEEEEDHDTRGMAEGRHAV-----NIEGFQSARVEDEV-----------------QVPECEPEKE------------E
Dog KANK1              EE-------------------------EEEEEEEDTRGVAEGQHAVNMEGFKSAGAEDEM-----------------QVPECEPEKV------------E
Human KANK1            EE----------------------EEEDEDTRGMAEGHHAV-----NIEGLKSARVEDEM-----------------QVQECEPEKV------------E
Anole lizard KANK1     EN--------------------GSLLNAEERHGLNREEKSA-----RVEGVVQDQEPEPE-------------------------------------KVE
Eagle KANK1            HT---------------------EEGQPAPRAAPEVCAMGA-----EKDSTPPECEAEEV---------------------------------------E
Chicken KANK1          VT----------------------DGEHPVLGPPETSAAGT-----AKDGPPLECEAEEV---------------------------------------E
Goose KANK1            GA---------------------EEEEDPALSAPETGAVGA-----EKDRPPLECEAEEV---------------------------------------E
Turtle KANK1           ME-----------------------GGHHELNAEDVGCVGV-----DEEVQAQESEPEKV---------------------------------------E
Aligator KANK1         GS---------------------------SLPNMEEGHSKI-----NIEDSCSVQVEEES-----------------QVQENEPEKL------------E
Spotted gar Kank1      AT-------------------------RSGEGHGKKPREFCNEGAMEVKRECEEREAEKV---------------------------------------E
Herring Kank1          KQ-----------------------AQENVGEEKTGGEEFCNEGAVEVGKRAELSGSEKQ---------------------------------------E
Zebrafish Kank1a       RE---------------------------RRKEEFRNNEAV-----DEDRDTEDLGQDKR----------------------------------------
Cave fish Kank1a       VK--------------------------EKRKEEFNNEGAV-----DVERDVEDMGQDKQ---------------------------------------E
Stickleback Kank1a     EE----------EYENVEARSVSEEFKPVGFREAGDEREVT-----EPCVSTRDPTNNKS--------------------------------------ID
Medaka Kank1a          KG----------------------------EEFHTEGGEDE-----DKEEEEESCEKKRE----------------------------------------
Fugu Kank1a            KE------------------------ELKAVEGKSTGEEFQSEGVENVDKKDENECSEKQ---------------------------------------G
Tetradon Kank1a        ----------------------------------------------------------------------------------------------------
Tilapia Kank1a         KE------------------------EHQKVEGESMREEFQPEGAEDVDKKDDEESSENR---------------------------------------E
Amazon molly Kank1a    QE----------------------------VGGRRIGEEFQ-----NEGAEDEDKKDEEE---------------------------------------G
Mouse KANK3            GG----------------------SDAGTPGPHNDKDAGDC-----ELETHPELTAGREG----------------------------------------
Rat KANK3              SS-------------------------GGSDAGTPGPHNDQ-----DAGDCELEMHPEQK---------------------------------------T
Dog KANK3              RD-----------------------------VGDDSGGDVL-----DPEREPEPEAEPQP---------------------------------------G
Human KANK3            DS----------------------------GGGSDSGTPGP-----PSGGDIRDPEPEAE--------------------------------------AE
Lamprey                EE-------------EEDDEGLGEVIHMQPNGGAAGRSEAE-----RQDGDAQEVGREEP------------------VAEAKQ---------------E
Coelacanth KANK3       IE---------------------TSEEELNVNMEDSDSDFENLQHVTTTGKMGKAERKEE------------------HEEQGMDLG------------E
Frog Kank3             YE-----------------DTSEEEMNVNMQDTDSDIEDCAVEEKRKEEKVKKRKDREEQ---------------------------------------P
Turtle KANK3           DA---------NTWDEEIGANVGETASSVEVPEPGTEQRKR-----NGEQGTESAPALDP---------T-----------------------------E
Aligator KANK3         SS-----------------------DEDVNVGENESDLENPEVDTEQRKREEQHEHREEH------------------IPAPEPP--------------E
Eagle KANK3            GD-----------------------TETSDEEAEEGEREPG-----QEREGAGVTLPEPP---------------------------------------E
Chicken KANK3          SE-----------------EPGGTDTSEEEEEDAVDGDGQSELCGQQEGEGGSMAPPEPP---------------------------------------E
Goose KANK3            TD--------------------------TSEEEEEDAGGGQ-----SEAGEQQEHEGANV------------------APPEPT---------------E
Spotted gar Kank3      GE---------------------------DGALIDTSEDER-----NVNLDDSDSEGEEP------------------AAEREEGQQS-----------A
Zebrafish Kank3        AA------------------ALEDTSDEEMNINADESDSDE-----NMPAGTEELKDQAE---------------------------------------E
Cavefish Kank3         EE-------------------------EAAAALEDTSDEER-----NINLEDSDSDENVQ------------------AVTEEGKEQTE----------E
Herring Kank3          EE------------------------EEAALQETSDEERNINLDESDSDEGQTGSGGQQE---------------------------------------T
Stickleback Kank3      DE---------------------------------TSEEER-----NVNLDESDTDEETL----------------------------------RDTSYS
Fugu Kank3             AA----------------------------LEEEETSEEER-----NINLDESDTDEETL------------------RDATYSSD-------------V
Tetradon Kank3         TE-------------------------DEAALDEETSEEER-----NVNLDESDTDEDTP---------------------------------SAYSSVA
Medaka Kank3           ED--------------------------EAPLEEETSEEER-----NVNLDESDTDEEML------------------QTANYSPD-------------A
Tilapia Kank3          EE-------------------------DEAALEEETSEEER-----NVNLDESDTDEETL------------------RAANYSSD-------------G
Amazon molly Kank3     TE------------------------EDEAALEEETSEEER-----NINLDESDTDEETL-------------------------------RAPSYSSDA
Platyfish Kank3        DS----------------------TEEDEAALEEETSEEER-----NVNLDESDTDEETL------------------RAPNYSSD-------------A
```

```
                          2710      2720      2730      2740      2750      2760      2770      2780      2790      2800
                      ....|....|....|....|....|....|....|....|....|....|....|....|....|....|....|....|....|....|....|....|
Hydra                 ---------------IESCKMLHAHFSG---ENQLK-QPAV--LQYMSIIQALWFKTVKRKRANAAIVKSYIDLFQQRVPELLETIMNMSDDEGNTALHF
Trichoplax            LEI----RDFINQDFKENFQKLDDHLNE---INILS-SSKL--DEILNQLRKQWFEVVLADNLQIAMLKQWIEETKISSSKALSVIIDMQDLNGNTLLHH
Aplasia               IRE----TYELSGEVREACKILATYLMD---STTIQ-TKQL--NASLNVIQQEWFQVSSHKLSTPHQVEDFLSSVNEISKRLLEYIVNMTDANGNTAIHY
Snail                 IRE----TYELSEEVREACTVLAAYLID---STDIQ-TKQL--NACQNIVQQEWFRVSSMKLSSAHQVEDFLSSVNEISKSLLKYIVNMTDSNGNTAIHY
Fruitfly              PRK----KTELSQSLKDALKIINDSLLKKLGAKPIS-SQSL--KSAKATIQDHWFTNSSTGKSDPHQVEDYLDYFESLSIPLLEYCVNLSDSNGNTAMHY
Hookworm              -------PYEISSPMREAMESLNSHLIQ---PGTIT-PETA--DWALKYVQHEWLKCAARKGSQAGAVDILIEQLRELSPALLKVVVNITDQNGNTALHY
C.elegance            -------PFEVSDRLREALENFNSSILE---PGSVA-EETV--EWSAKLVRHVWMTTAGNSSSRYENVDRFVAELGNLGPQILHMVVNFCDQNGNTALHY
Tunicate              VFQDENKKYQFTSELLDACDILEMHMRV---PKSVN-HSQL--ENATSTVQQEWFRVAAQPNSNVKTLDDFVTDLADTSLPVLRKVVNMTDNSGNTVLHY
Spider                PRK----KTELNKEVRAACKVLNDYLLK---PDRGG-EKKT--LSSLNIIQNEWFKVANQKNSDPHVIEDYLDAFEEFSKLLLERVVNLSDSNGNTALHY
Lingula               IRE----TYELSEEMQASCELLSRHLED---PNTIP-TEQV--TAAATIIQQEWFKAAGQKLSSPYMVEDFLSCFNEYSKELLERIVNMTDTNGNTALHY
Lancelet              LKE----THELSEELQEACGVLEKHVTT---PKSVP-PREL--MMAMNTIQQEWMKAGAQKTSDVQVVRDYLAAFEQMATGVLGKIVNLADGNGNTALHY
Medaka Kank4b         ARA----REANDADFVSACHFVKAHMGN--MDNP---SEDM--RKALVTLFQHWFTTAAEEASEACNVAACVRGVKKETPSLLAFLVNLPDDNGNTVLHY
Amazon molly Kank4b   MKG----RDILSADFMTACHFVKDHMDI--TENP---NDDM--RKALVTMFQHWFGTAAEEASEPSRVAAYLKEVKSEAPPLLAFLVNLADDNGNTVLHY
Platyfish Kank4b      TKG----RDILSADFMSACHFVKDRMDI--TENP---NDDM--RQALVTMFQHWFGAAAEEASETSRVAAYLKEVKSEAPPLLAFLVNLADDNGNTVLHY
Stickleback Kank4b    -------RETVNLDFLAACHLLKDHMDH--IDNP---NEDM--RKALVVLFQHWFSAAAEETSEAGRVSAYLSDVKKTTPSLLAFLINLADDNGNTALHY
Fugu Kank4b           SRG----REIVSSDFMTACQFLNDHMDN--MDNP---NNEM--RKALIVLFQRWFSVAAEETSMVGDLAAYLREVRKSTPTLLAFLVNLADDNGNTVLHY
Tetraon Kank4b        -------RETVSSDFMTACRFLNDHMDN--MDNP---NNEM--RKALIVLFQRWFSVAAEETSVPGDLAAYLREVRKATPTLLAFLVNLADDNGNTVLHY
Cave fish Kank4b      L------KTFPPDQIIESIVFIKEQKTREEDPTEET-TRE---EEEEEAMKQEPRQTCEAECPDDPPVKDESEQVETCSGVETQELSE--DGRGETEI--
Medaka Kank4a         VSELFAQIYKVDKGFIDACVYVKDRMEE--VSSP---DKEM--RQVLVVLYQEWFRVSSQKDSQADTVRLYLRQVGRTTPTLLPYVVNLTDGNGNMALHY
Fugu Kank4a           LRE---TREKVDKRFIDACVYVKDRMEE--VSSP---DKEM--RQVLVALYQEWFRVSSQKDSQADTVRSYLRQVSLTTPTLLPYVVNLTDGNGNMALHY
Tetradon Kank4a       PEGSSELLEEQAEGFIDACVYVKDRMEE--VSSP---DKEMVRRQVLVALYQEWFRVSSQKDSQADTVRSYLRQVSLTTPTLLPYVVNLTDGNGNMALHY
Stickleback Kank4a    -------GEKVNQGFIDACLYVKDRMEE--VSSP---DKEM--RQVLVVLYQEWFKISSQKESQADTVRLYLRQVGFTTPTLLPYVVNLTDGNGNMALHY
Tilapia Kank4a        QAE----GGKVDKGFIDACLYVKDRMEE--VSSP---DKEM--RQVLVVLYQEWFRVSSQKDSQADTVRLYLRQVGMTTPTLLPYVVNLTDGNGNMALHY
Platyfish Kank4a      VEE--AEREKVDKGFIDACVYVKDRMEE--VSSP---DKEM--RQVLVVLYQEWFKVSSQKDSQADTVRLYLRQVGLTTPTLLPYVVNLTDGNGNMALHY
Amazon Molly Kank4a   VEE--AEREKVDKGFIDACVYVKDRMEE--VSSP---DKEM--RQVLVVLYQEWFRVSSQKDSQADTVRLYLRQVGLTTPTLLPYVVNLTDGNGNMALHY
Herring Kank4         SSPEQPVSEVVDKDFMAACYYLKDRRNE--VDNP---NKEM--RQVLMILYQEWFRVSSQKDSQAEAVTLYLREVGNATPTLLSYIVNLADGNGNTALHY
Zebradish Kank4       PQEEQPVSELVDKNFMAACHFLKDRMAE--VSAP---NKEM--RQVLMMLYQEWFRVSSQKDSQAETVTLYLREVGFHTPTLLRYIVNLADGNGNMALHY
Cave fish Kank4a      PQEEQPVSEFVDKNFMAACHYLKDRMAE--VSTP---NKDM--RQVLMVLYQEWFRVSSQKESQEETVTLYLREIGNQTPTLLRYIVNLADGNGNMALHY
Mouse KANK4           VSHLRAERYKPSEEFLNACQTLSQHLPE---TGDTT-KQLL--RQSLNTISQEWFRVSSRKLSSPEAVAAYLLEVQPHSPYLLKLLVNLADRSGNTALHY
Rat KANK4             VSHMKAERYKPSEEFLNACQALSQHLPE---AGATN-KQLL--RQSLNTISQEWFRVSSRKLSSPEAVAACLLEVQPQSPHLLKLLVNLADRSGNTALHY
Dog KANK4             LPHPKAERYKPSEEFINACRALSQHLPE---TGATT-EQLL--RQSLNTIRQEWFRVSSRKSSSPAVVAAYLRGVQPHSPHFLKLLVNLADGNGNTALHY
Human KANK4           VPHSKAERYKPSEEFLNACRALSQHLPE---TGTTT-DQLL--RQSLNTISQEWFRVSSRKSSSPAVVASYLHEVQPHSPHFLKLLVNLADHNGNTALHY
Eagle KANK4           KAD----RCKPSEDFLADCQLLSKHLSE---IRTTS-DKHL--RHVLSTVCQEWFRVSSRKSSSPEVVGAYLKALGAIQPQLLAMVVNLADRNGNTALHY
Chicken KANK4         LQEPGAETCKPSEDFLAACQLLSQHLSE---IRATS-DQHL--RHVLSTICQEWFQVSSRKSSSPEVVTAYLQALGTIQLQLLEAVVNMADRNGNTALHY
Goose KANK4           GTEHKADRCKPSEDFLADCQLLSQHLSE---IRTTS-DEHL--RHVLSTVCQEWFRVSSRKSSSPEAVAAYLQVLGAIQPQLLEAVVNLADRNGNTALHY
Anole lizard KANK4    MQPHKAERFKPSADFLKGCQILSKHIPE---IRNTK-DKLL--RHVLSTISQEWFRISSRKSSSPEIVAAYLREVEVIHPQLQNIVVNLADGNGNTALHY
Turtle KANK4          SIQPKTDRCKPSEDFLDGCLLLSKHLSE---IRTTN-DKHL--RHILNTVCQEWFRVSTRKSSSPEVVAAYLKEFKAIHPQLLKMIINLADGNGNTALHY
Aligator KANK4        STQHKTDRYKPSEDFLNGCRLLRKHLSE---IRTTG-DKHL--RHVFNSVCQEWFRVSSRKSSSPEIVAVYLREFQAIDPQLLKRIVNLADGNGNXALHY
Frog Kank2            PTP----RVELSNVLISGCVALQKSLDE---GTALP-EMEK--SAAHSVILQEWMKILRQKDVDPAVIRHHLTVFRAMSPRLLEVIINMADAKGNTAVHY
Mouse KANK2           PDPEEEIRMDLSPDLISACLALEKYLEN---PNALT-EREL--KVAYTTVLQEWLRLACRSDAHPELVRRHLVTFRAMSARLLDYVVNIADSNGNTALHY
Rat KANK2             PDPEEEIRMDLSPDLISACLALEKYLEN---PNALT-EREL--KVAYTTVLQEWLRLACRSDAHPELVRRHLVTFRAMSARLLDYVVNIADSNGNTALHY
Dog KANK2             ANAEEEIRMELSPDLISACLALEKYLDN---PNALT-EREL--KVAYTTVLQEWLRLACRSDAHPELVRRHLVTFRAMSVRLLDYVVNIADSNGNTALHY
Human KANK2           SNTEEEIRMELSPDLISACLALEKYLDN---PNALT-EREL--KVAYTTVLQEWLRLACRSDAHPELVRRHLVTFRAMSARLLDYVVNIADSNGNTALHY
Aligator KANK2        -------GLGLGQELLSACTILKRYLET---PNASM-DTKT--RDAYGVVQQDWLGLACGQAVEATAVSQRLDTYRAFSPRVLATALNMTDGHGNTALHH
Spotted gar Kank2     TQPEDSTRLELSDTLMSACTVLQKALGE---TNAFS-QQEA--RTAYTTVLQEWLRVSCHKAADAEVVRRYMVAFQAISPQLLEFIINMADGNGNTTLHY
Cave fish Kank2       VAAQQEIRGELSESLMTALQTLQKALGE---PNAFS-QQGA--RTAYTAVLQEWLKVSCHKSADTAVVRSYMDAFASVSPELLEFVVNMADGNGNTALHY
Herring Kank2         -------KLELSESLITALHTLQKALGE---PNAFS-QQEA--RTAYTTVLQEWLRVSCHKGADTAVVRAYMDAFASVSPQLLEFVVNMADGNGNTALHY
Zebrafish Kank2       PTNKQTDSLELSGGLMSALHILQKALSE---PNAFS-HQDA--RTAYTSVLQEWLRVSCHKAADTAVVKAHMDAFASISPQLLEFVINMADGNGNTALHY
Tetradon Kank2        DAPAKPVRVELSDSLVSALHVLQKALGE---PNAFV-HQAA--RAAYTAVLQEW-RVSCHKAADTAVVQAHMSAFASISPQLLEFVINMADGNGNTALHY
Fugu Kank2            SEKANPVRVELSDGLMSALHILQKALGD---PNAFS-HQEA--RAAYTTVLQEWLRVSCHKAADTAVVQAYMNAFASISPQLLEFVINMADGNGNTALHY
Medaka Kank2          AASSNQVRAELSKNLMSALQTLRKALGE---PNAFS-QQAA--REAYTTVLQEWLRVSCHKAADTSVVKAYMDTFASLSPQLLEFVINMADGNGNTALHY
Sticklebakc Kank2     AAPPKQVRLDLSDSLMSTLHALQKALGE---PNAFS-QQGA--RAAYTTVLQEWLRVSCHKAADTAVVEAYMGAFASVSPQLLEFVINMADGNGNTALHY
Tilapia Kank2         LN-----RLELSDSLMLALHALQKALGE---PNAFS-QQGA--RAAYTTVLQEWLRVSCHKTADTAVVKAYMDTFASISPQLLEFVINMADGNGNTALHY
Amazon molly Kank2    -------RLELSESLMSALYTLQKALGE---PNAFS-QQAA--RTAYTIVLQEWLRVSCHKAANTAVVKAYMDTFASISPQLLEFVINMADGNGNTALHY
Platyfish Kank2       QESSKPVRLELSESLMSALYTLQKALGD---PNAFS-QQAA--RTAYTIVLQEWLRVSCHKAANTADVKTYMDTFASISPQLLEFVINMADGNGNTALHY
```

```
Coelacanth KANK2     TEQ----KFQMSESLLSASMTLQKYLEN---PDAVS-NMAM--KAAYATVLQDWLRISCHKAADQNVVRHHMIVFQAMSPQLLEFVVNMADGNGNTALHY
Anole lizard KANK2   KEQPSTTGMVLSKELLSACEVLQKYLES---PNAHM-DEEM--KLAYTTVLPYWLRLSCHKEADPELVSLHLAAFRAISPDLLEFIINMADANGNTALHY
Turtle KANK2         AAPGSGTGTGLSPELLAACETLQKYLES---PDMLM-DKEM--KAAYTLVLQDWLQLSCHREAAPEAVGGHLAAYRALSPRLLEFVLNMADGNGNTALHY
Tetradon Kank1b      CNILFHFRCKLSEKMFSACQALKVHLSD---SRALS-SREL--LDCLQTVQQEWFSVSSQKSAVPDTVDTYLSAFRSVSPSVLQHIANMADGNGNTALHY
Medaka Kank1b        -------RYRLNEKMFSACQILKAHLSE---ATPLS-NKEL--QECLHTISQEWFSVSSHKSASPDTVDKYLSMFELISTSVLQHIANMADGSGNTALHY
Amazon molly Kank1b  SRQH---RCNLSERMFSACQALKMHLSE---NPAMS-SREL--QDCLCSLQQEWFSVSSQKSAAPDIVGEYLSAFRMISPSVLQHIVNMADSNGNTALHY
Platyfish Kank1b     TQH----RCNLSERMFSACQDLKMHLSE---NPATS-SREL--QDCLRSLQQEWFSVSSQKSAAPDTVGEYLSAFRMISPSVLQHVVNMADGNGNTALHY
Frog Kank4           VTQ----RCTVSDAFRSECQKLSKHLAG---LQGTT-DNQL--RQTLYTVCQEWFRVSSQKTSSSDLVTVYLEELRSICPQLLQMVVNMSDENGNTALHY
Zebrafish Kank1b     IRG---------IDLVRRC-FLRAKLSR---FTSMA-ARPY----PIENI---------QKRVCPQIVEDFLFACCQVSPDVLCYVANMPDQNGNTALHY
Tilapia Kank1b       IRG---------IDLVRRC-FLRAKLSR---FTSMA-ARPY----PIENI---------QKRVCPQIVEDFLFACCQVSPDVLCYVANMPDQNGNTALHY
Fugu Kank1b          IRG---------IDLVRRC-FLRAKLSR---FTSMA-ARPY----PIENI---------QKRVCPQIVEDFLFACCQVSPDVLCYVANMPDQNGNTALHY
Cavefish Kank1b      RRE----KYEMSEKVLSYCHSLKAHLNG---SKALS-NREL--RSCLNTIQQEWFRVSSQKKACPDTVEDFLCLCRHVSPAVLTHVANMADQNGNTALHY
Spotted gar Kank4    ILHHLHFRETVDEDFMAACLFLKDHLSE-----VISPSREL--RQVLSVVYYEWFRVSSQKASQAHALALYLQELKAVSPEVLEFVINSADGNGNTALHY
Coelacanth KANK4     KTE----RVQIGEDFLAACLLLSHHLSE---IRTTT-DRQL--KQSLTAVCQEWFAVSSQKLSVPEPVEDYLKEFKSISLELLMLIMNIADGNGNTALHY
Frog Kank1           IRE----RYELSEKMLSACNVLKDTVSD---PKALA-NKDV--RLCLTTVQNEWFRVSSQKSAVAPMVEDYITAFKDLSPAVLSYIINMADGNGNTALHY
Shark Kank1          IRE----RYELSEKMVSACKLLKSQLCD---PKAMA-SKAV--RLCLNSVQHEWFRVSSQKSAVPTMVEDYLTGFREISLEVLKHVVNMADGNGNTALHY
Coelacanth KANK1     IRE----RYELSEKMMSACSVLKNNISD---PKALT-SKDV--RACLNTIQHEWFRVSSQKSAVPAMVGDYLAAFKGVSPDVLRYVVNMADGNGNTALHY
Mouse KANK1          IRE----RYELSEKMLSACNLLKYNIKD---PKALA-SKDM--RICLNTLQHDWFRVSSQKSAVPAMVGDYIAAFEAVSPDVLRYIINMADGNGNTALHY
Rat KANK1            IRE----RYELSEKMLSACSLLKYNIND---PKALA-SKDM--RICLNTLQHEWFRVSSQKSAVPAMVGDYIAAFEAVSPDVLRYIINMADGNGNTALHY
Dog KANK1            IRE----RYELSEKMLSACHLLKNNIND---PKALT-SKDM--RFCLNTLQHEWFRVSSQKSASPAMVGDYIAAFEAISPDVLRYIINMADGNGNTALHY
Human KANK1          IRE----RYELSEKMLSACNLLKNTIND---PKALT-SKDM--RFCLNTLQHEWFRVSSQKSAIPAMVGDYIAAFEAISPDVLRYVINLADGNGNTALHY
Anole lizard KANK1   IRE----RYELSEKMLSACQLLKSSIDD---PKAMN-SKET--RFCLNTIQHEWFRVSSQKSAVPAMVGDYIAAFEEVSPVVLRHIINMADGNGNTALHY
Eagle KANK1          IRE----RYELSEKMLSACHLLRNNIDD---PKALT-NKDV--RFCLNTIQHEWFRVSSQKSAVPEMVGDYITAFEEISPAVLRHIINMADGNGNTALHY
Chicken KANK1        IRE----RYELSEKMLSACHLLKNNIDD---PKALT-NKDV--RFCLNTIQHEWFRVSSQKSAVPEMVGDYITAFEEISPAVLRHIINVADGNGNTALHY
Goose KANK1          IRE----RYELSEKMLSACHLLKNNIDD---PKALT-NKDV--RFCLNTIQHEWFRVSSQKSAVPEMVGDYITAFEEISPTVLRHVINMADGNGNTALHY
Turtle KANK1         IRE----RYELSEKMLSACNLLKSNLDD---PKALA-SKDM--RFCLNTIQHEWFRISSQKSAVPAMVGDYIAAFEELSPPVLRHIINMADGNGNTALHY
Aligator KANK1       TRE----RYELSEKMLSACNLLKKNIDD---SKAMA-TKDM--RFCLNTIQHEWFRVSSQKLAVPEMVGDYIAAFREVSSTVLRHIINMADGNGNTALHY
Spotted gar Kank1    IRE----RYELSEKMLSACTVLRNHLND---SKALT-SKEV--RACLNTIQHEWFRISSQKSALPAMVEDYLSAFREVSPAVLRHIVNMADGNGNTALHY
Herring Kank1        RRE----RYELSEKMVSACNVLKAHLND---SKSLT-SKDV--RASLNTVQQEWFRVSSQKSAVPAMVEDYLAAFRGVSSSVLRHVVNMADGNGNTALHY
Zebrafish Kank1a     ------QRYELSEKMLSACNILKSHLND---PKALS-SKDV--RPCVNTVQHEWFRVSSQKTAVAEMVEDYLNAFRSISPALLQHIVNMADGNGNTALHY
Cave fish Kank1a     TRE----RYELSEKMLAACNVVKAHLND---PKSSS-SKEV--RMCMNTVQHEWFRVSSQKTAVATMVEDYLNAFRNISPALLKHVVNLADGNGNTALHY
Stickleback Kank1a   CVH----RYSLSEKMLSATNVLRAHLSD---PKAVS-SKDL--RTCINTVQHEWFRVSSQKSASAPMVGDYLAAFGAVSPAVLRHVVNMADGNGNTALHY
Medaka Kank1a        -------SYELSEKMAVACGVLKAHLDD---PNAVS-GKDL--RACINTLQHEWFRVSSQKAALAAMVDDYLRRFEAVSPAVLRYIVNMADGNGNTALHY
Fugu Kank1a          IRE----RYKPSEKMLAACNVLKTHLCD---PKAVG-SKDL--RACVNTVQHEWFRVSSQKTALAAMVEDYLSAFRDVSPDVLHHIANMADGNGNTALHY
Tetradon Kank1a      -------RYKPSEKMLAACSVLSAHLCD---SKAVS-SKDL--RACVNTVQHEWFRVSSQKTALATMVEDYLSAFRDVSPAVLNHIANMADGNGNTALHY
Tilapia Kank1a       TRE----RYELSEKMLAACNVLKSHLSD---PKAVT-SKDL--RGCLNTVQHEWFRVSSQKAAVAAMVEDYLGAYADFSPAVLRHIVNMADGNGNTALHY
Amazon molly Kank1a  SENEKRERYELSEKMLAACDVLKTHLSD---SKAVS-SKDL--RASLNTVQHEWFRASSQKAALAPMVEDYLRTFEAVSMDVLRHVVNMADGNGNTALHY
Mouse KANK3          -------RCELNPRLREACIALNQQLNR---PRGVT-SRDG---NAARLVAQEWFRVSSQKRSQAESVAGVLRGVKSLGPELLAYVVNLADGNGNTALHY
Rat KANK3            GREG---RCELNPRLREACIALNQQLMR---PRGVT-SRDG---NAARLVAQEWFRVSSQKRCQAEPVAGVLRGVKSLGPELLAHVVNLADGNGNTALHY
Dog KANK3            AQG----RCELSPRLREACAALQRQLSR---PRGV--ARDG---GAARLVAQEWFRVSSQRRSQAEAVAGVLGAVGRLGPELLAHVVNLADGNGNTALHY
Human KANK3          PQQVAQGRCELSPRLREACVALQRQLSR---PRGVA-SDGG----AVRLVAQEWFRVSSQRRSQAEPVARMLEGVRRLGPELLAHVVNLADGNGNTALHY
Lamprey              EQELCHCRYEMSESMHTACSTLKAHLED---HKSLN-TKEV-------------------------------------------VMADGNGNTALHY
Coelacanth KANK3     VKE----KFELSPKMREACLIMKNHMND---ADTSR-SKDV--MSSVNLVQQEWFLVSSQKTSFSETVADYLMAFAEISPALLGYVVNMSDGNGNTALHY
Frog Kank3           TQHDTKKSCVLSQKMQEACLVVSIHLCE---PNAVK-TKQS--LASSHIVQQEWFRASSQKSSVAETVAAYLKAFMDISPEMLSHVVNLTDGNGNTALHY
Turtle KANK3         VKE----KFELSPRMREACMIVKHHLSH---PSAVK-SKEV--LASSSILLQEWFRVSSQKSSVPELVASHLLAFAEVSPGMLAHVVNLSDGNGNTALHY
Aligator KANK3       VKE----KFELSPRMREACVIVKSHLSH---PSSAK-SKEV--LSSSSLILQEWFRVSSQKSSVSEMVANHLMAFTEVSPALLTHVINLSDGNGNTALHY
Eagle KANK3          VKE----KFELSPRMREACLIVKTHLGH---PSATK-SKEV--LASSSLVLQEWFRLSSQKSSIPDTVANHLLAFAEVSPALLAHVVNLADGNGNTALHY
Chicken KANK3        VKE----KFELSPRMREACLIVKTHLGH---PTATK-SKEV--LASSSLVLQEWFRLSSQKSSVPDTVANHLLAFAELSPALLTHVVNLADGNGNTALHY
Goose KANK3          VKE----KFELSPRMREACLIVKTHLGH---PGATK-SKEA--LASSSLVLQEWFRLSSQKSSVPDTVANHLLAFAELSPALLAHVVNLADGNGNTALHY
Spotted gar Kank3    VKE----KFELSKKMREACLILKNHLND--DINALK-SKEV--LSSTHTVQQEWFRVSSAKTALPSRVADYLMAFSEVSPALLRHVVNMTDSNGNTALHY
Zebrafish Kank3      VKE----KFELSSKMREACLILKNHLND--GEKAVK-SKEV--LSSTHSVQLEWFRVSSAKMAQPLRVSNYLMAFSEVSPVLLEHIVNMTDGNGNTALHY
Cavefish Kank3       VKE----KFELSSKMREACLILKNHLND--GAKAAK-SKEL--LSSTHTVQLEWFRVSSAKMSQPTRVSNYLMAFSEISPVLLGHVVNMTDGNGNTALHY
Herring Kank3        VKE----KFELSMKMREACLILKNHLND--DAKAMK-SKEV--LSSTHTVQLEWFRVSSAKAAQPLRVSNYLMAFSEVSPVLLEHVVNMTDGNGNTALHY
Stickleback Kank3    CDAVKENRFELSSKMREACLILKNHLKD--DIAALKICHPK--LSSTHTVQLEWFRISSAKMAQSPRVSDYLMAFSEVSSALLEHVVNMTDGNGNTALHY
Fugu Kank3           VKENF--RFEFSSKMREACLILRNHLND--DARAPK-SKEV--LSSTHTVQLEWFRISSAKTAQPSRVSDYLMAFSEVSSVLLEHVVNMTDGNGNTALHY
Tetradon Kank3       VKE----KFEFGSKMREACLILRNHLND--EAKALR-SKEV--LSSTHTVQLEWFRVSSAKTAQPSRVSDYLMAFSEVSPVLLEHVVNVTDGNGNTALHY
Medaka Kank3         VKE----KFEFSSKMREACLILKNHLND--DVKTLK-SKEV--LSSTHTVQLEWFRISSAKTAQPSRVSDYLMAFSEVSSALLEHVVNMTDGNGNTALHY
```

```
Tilapia Kank3          VKE----KFELSSKMREACLILKNHLND--DLKTLK-SKEV--LSSTHTVQLEWFRISSAKMAQPPRVSDYLMAFSEVSSMLLEHVVNMTDGNGNTALHY
Amazon molly Kank3     VKE----KFEFSSKMREACLILKNHLND--DVKTLK-SKEV--LSSSHTVQLEWFRVSSAKMAQPSRVSDYLMAFSEVSSALLEHVVNMTDGNGNTALHY
Platyfish Kank3        VKE----KFEFSSKMREACLILKNHLND--DVKTLK-SKEV--LSSTHTVQLEWFRVSSAKMAQTSRVSDYLMAFSEVSLALLEHVVNMTDGNGNTALHY

                                2810      2820      2830      2840      2850      2860      2870      2880      2890      2900
                       ....|....|....|....|....|....|....|....|....|....|....|....|....|....|....|....|....|....|....|....|
Hydra                  AITYKNYAVVNILLD-SGECSVDTINQSGYSPIMMACLTGFE-NKSDKYIMQRLFRMGDINKPVA-ETGETPLMLAVSHGHIGVVELLLDVGCDINAIDD
Trichoplax             CVHNKEFALAEYLLN-TELCQAGLYNQLGYTPIMVAALFDIK-NDDERRIIMKMLSHSDVDAKAT-KTGQTALMLAAAHNKVIFSHLLIQAGADINLQDK
Aplasia                CISHCNFEIANLLLD-SEVCDINRQNKAGYTPIMLAGLATLQ-NMGQREIVRRLFSSGDINARAT-ATMQTALMLAASHGRAETVRLLTEEGADVNLQDV
Snail                  CISHCNFEIASLLLD-SEVCDNNKQNKAGYTPIMLAGLASIQ-NTDQLEVVRRIFSTGDINARAS-ETQQTALMLAASHGKTEMVKLLVEEGAEINLQDV
Fruitfly               AVSHGNFDVVSILLD-SKVCDVNQMNNAGYTSVMLVSLAKLK-QPAHRTVVERLFKMADVNIRAK-KHCQTALMLAVSHGNGDMVNMLLDAGADINIQDE
Hookworm               AVSHGNFGVVSSLLD-SGECQLNLANRAGYSAVMLAALSSLE-NDVEKAVVQRLFELGDVNARAA-QHGQTALMLSVSHGKKNTTELLLQCGAQVNLQDE
C.elegance             AVSHANFAVVSSILD-SGECDLDAPNRAGYTAVMLAALSQLD-DDMEKAVVHRLFQMGNVNAKAS-QHGQTALMLAVSHGKKTTTELLLACGANVNEQDQ
Tunicate               AISHNNMRIVETLLR-IEELDVNKLNHAGYTAAMLGSLCHVE-SEADMKIFKQLFEKSNINTHAK-EAGQTALMLAVSHGRYKTTKLLIDCGAEVNARDN
Spider                 AVSYGNFDVVSILID-SKVCDVNKKNKAGYTSIMLVALADMR-NDAHHYVVQRLFSIGDINIKAT-QNGQTALMLAVSHGKKEMVKMLLDVGAEVNLQDK
Lingula                SVSHCNWDVVSLLLD-TGLCEVDKPNRAGYTAIMLASLADVQ-TEEQKDVVKQLFDAGDVNSRAS-QAGQTALMLAVSHGRLEMVRLLAEAGADVNAQDE
Lancelet               SVSHSNFDIVEILLE-TGQCEVNKQNKAGYTAIMLASLAKLQ-GDRQMEVVKKLFQAGDVNIRAT-QAGQTALMLAVSHGRQEIVKMLIEAGAEVNLQDE
Medaka Kank4b          SVSHCNYSIVSLLLD-TGVADVNVQNKAGYTAVMLASLTAPD-GPDGMETVRKLMELGNLNVRSS-QTGQTALHLAVRHGRDIMVRLLLSCGADTNIQDN
Amazon molly Kank4b    SVSHCNYAVVSLLLD-TGMSDVNVQNNAGYTAVMLASLTAPD-GPAGMEVVRKLMELGNVNICSS-QTGQTALHLAVRHGRVVMVRLLLSCGADANIQDN
Platyfish Kank4b       SVSHCNYGVVSVLLD-TGMSDVNVQNNAGYTAVMLASLTAPD-GPAGMEVVRKLMELGNVNIRSS-QTGQTALHLAVRHGRVVMVRLLLSCGADANIQDK
Stickleback Kank4b     SVSHCNHSIVSLILD-TGVSNVGLQNNAGFTAVMLASLTAPD-GPGGMEVVRRLMEQSNINIRSS-QTGQTALHLAVRHGRVVMVRLLLSFGADTNLQDK
Fugu Kank4b            SVSHCNYSIVGLLLD-TGACDVNLQNNAGYTAVMLAALTAPD-GPGGMEVVRKLMEMGNIHNRSK-QTGQTVLHLAVRHGRVVMVRLLLSYGADANIQDN
Tetraon Kank4b         SVSHCNYGIVGLLLE-TGACDVNLQNNAGYTAVMLAALTAPD-GPGGMEVVRKLMEMGNIHIRSK-QTGQTALHLAVRHGRVVMVRLLLSYGADANIQDH
Cave fish Kank4b       ---NKDFTAACYFLE-------NHMNEVS-------------DPNDDMPLKRLGERNPTHHPAP-----TALRPPTQLGIYRM-------------GD
Medaka Kank4a          SVSHSNFSVVKLLLD-TGLCEIDNVNKAGYTPVMLAALTAAE-SADDLEVAHQLLRLGDVNARSR-QAGQTALMLAVRHGRVAMVKLLLSCGADVNAQDC
Fugu Kank4a            CVSHSNFPVVKLLLD-TGLCETDNMNKAGYTPVMLAALTAAD-SSDDLEVAQQLLKLGNVDTRSR-QAGQTALMLAVSHGRVAMVKLLLSCAADVNLQDY
Tetradon Kank4a        CVSHSNFPVVKLLLD-TGLCETDNMNKAGYTPVMLAALTAAD-SPDDLEVAQQLLKLGNVDACSR-QAGQTALMLAVSHGRVAMVKLLLSCGADVNLQDY
Stickleback Kank4a     SVSHSNFPVVKLLLD-TGLCETDNVNKAGYTPVMLAALTAAE-SSDDLEVAQQLLRMGDVNACSR-QAGQTALMLAVSHGRVAMVKLLLSCGADVNAQDR
Tilapia Kank4a         SVSHSNFPVVKLLLD-TGLCETDNFNKAGYTPVMLAALTAAE-SPDDLEVAQQLLKVGDVNARSR-QAGQTALMLAVSHGRVAMVKLLLSCGADVNAQDR
Platyfish Kank4a       SVSHSNFPVVKLLLD-TGLCETDNVNKAGYTPVMLAALTAAE-SPDDLEVAEQLLKLGDVNARSR-QAGQTALMLAVSHGRVAMVKLLLSCGADVNAQDS
Amazon Molly Kank4a    SVSHSNFPVVKLLLD-TGLCETDNVNKAGYTPVMLAALTAAE-SPDDLEVAEQLLKLGDVNARSR-QAGQTALMLAVSHGRVAMVKLLLSCGADVNAQDS
Herring Kank4          TVSHSNFPVVKLLLD-TELCEVDHQNKAGYTAIMLAALTAAE-SPEDVEVAQELLQKGNMDAHAS-QTGQTALMLAVSHGRTAMVRVLLSCGADVNVQDR
Zebradish Kank4        SVSHSNFTVVKLLLD-TGLCEVDHQNKAGYTAIMLAALTATE-NPEDMEVAQQLLRMGKINARAS-QSGQTALMLAVSHGRATMVQVLLDSGAEVNLQDR
Cave fish Kank4a       SVSHSNFPVVKLLLD-TGLCEVNHQNKAGYTAIMLAALTAAE-GPEDMEVAQQLLSLGNINAKAG-QSGQTALMLAVSHGRAAMVQVLLDCKADVNVQDQ
Mouse KANK4            SVSHSNFAIVKLLLD-TGVCNVDHQNKAGYTAVMITPLASAE-TKEDMAVVWKLLREGNVNIQAT-QGGQTALMLGVSHDREDMVQALLSCQADVNLQDN
Rat KANK4              SVSHSNFAIAKLLLD-TGVCNVDHQNKAGYTAVMITPLASPK-TKEDMAVVWKLLREGNVNIQAT-QGGQTALMLGVSHDREDMVQALLSCKADVNLQDH
Dog KANK4              SVSHSNFSVVRLLLE-TGVCNVDHQNKAGYTAVMITPLASAE-TDEDMAVVWKLLREGNVNIQAT-QGGQTALMLGVSHDREDMVQALLSCQADVNVQDH
Human KANK4            SVSHSNFSIVKLLLE-TGVCNVDHQNKAGYTAVMITPLASAE-TNEDMAVVWKLLREGNVNIQAT-QGGQTALMLGVSHDREDMVQALLSCQADVNLQDH
Eagle KANK4            SVSHSNFPIAKLLLD-TGVCRLDLQNRAGYTAVMLTPLAAAE-TGQDMEVVMKLLKEGDVNLRAA-QGGQTALMLGVSHERDDMVRALLSCQADVNLQDE
Chicken KANK4          SISHSNFTIAKLLLD-TGVCRLDLPNQAGYTAVMLTPLVAAE-TDEDMAVVMRLLKEGDVNVRAA-QGGQTALMLGVSHEREAMVRALLACQADVNLQDE
Goose KANK4            SVSHSNFPIAKLLLD-TGVCRLDLPNRAGYTAVMLTPLAAAE-TSEDMAVVMRLLKEGDVNVRAA-QGGQTALMLGVSHEREDMVRALLSCRADVNLQDE
Anole lizard KANK4     SVSHSNFQIVKLLLE-TGVCDVDHQNKAGYTAVMITPLASAE-TDEEMEVVTKLLREGNVNIRAS-QGGQIALILGVSHDREDMVKALLSSGADINLQDD
Turtle KANK4           SVSHSNFLIVKLLLD-TGVCDVDHQNKAGYTAVMITPLASAE-SDEDMEVVLKLLKEGDVNIRAS-QGGQTALILGVSHDRGDMVKALLSCKADINQQDD
Aligator KANK4         SVSHSNFLIVKLLLD-TGVCDVDHQNKAGYTAVMITPLASAE-TDEDMEVVLKLLKFGNVNIRAS-QGGQTALMLGVSHDREDMVKALLSCKADINLKDD
Frog Kank2             AVSQSNFTAVRQLLD-TGLCDVNRQNKAGFTPLMLTALAAFR-SDEDIETVTQMLRLGDVNCRAS-QAGQTALMLAVSHGRLDVVRALLACGADVNIQDH
Mouse KANK2            SVSHANFPVVRQLLD-SGVCHVDKLNRAGYSPIMLTALATLK-TQDDIETILQLFRLGNVNAKAS-QAGQTALMLAVSHGRVDVVRALLACEADVNIQDE
Rat KANK2              SVSHANFPVVRQLLD-SGVCQVDKLNRAGYSPIMLTALATLK-TQDDIDTILQLFRLGNVNAKAS-QAGQTALMLAVSHGRVDVVKALLACEVDVNMQDE
Dog KANK2              SVSHANFPVVRQLLD-SGVCQVDKQNRAGYSPIMLTALANLK-TQDDIETVLQLFRLGDVNAKAS-QAGQTALMLAVSHGRVDVVKALLACEADVNVQDD
Human KANK2            SVSHANFPVVQQLLD-SGVCKVDKQNRAGYSPIMLTALATLK-TQDDIETVLQLFRLGNINAKAS-QAGQTALMLAVSHGRVDVVKALLACEADVNVQDD
Aligator KANK2         AVSCSNFPLVRRLLD-TGLCDVDKPNQAGYTALMLTALAAAY-PDADRATVLQLLRAGNVDARAT-QAGQTALMLAVSHGRQDVARVLLACGANVNLQDA
Spotted gar Kank2      TVSHSNFPVVKLLLD-TGLCNADKQNKAGYTAIMLTALAAFH-SSSDLETVTQLLRTGDVNAKAS-QAGQTALMLAVSHGRSDMVGALLACGANVNLQDD
Cave fish Kank2        TVSHSNFPVVKLLLD-TG------------NVTVCMLARCV-CD------------------V-QAGQTALMLAVSHGRGDMVRALLTCGAQVNLQDD
Herring Kank2          TVSHSNFPVVKLLLD-TGLCNADKQNKAGYTAIMLTALAAFS-SDSDLQTVLQLLRTGDVNAKAS-QAGQTALMLAVSHGRGDMVRALLSCGAQVNLRDD
Zebrafish Kank2        TVSHSNFPVVKLLLD-TGLCNADKQNKAGYTAIMLTALAAFS-SDSDLQTVLQLLRTGDVNAKAS-QAGQTALMLAVSHGRGDMVKALLACGAQVNLRDD
Tetradon Kank2         TVSHSNFPVVKLLLD-TGLCNADKQNKAGYTAIMLTALAAFH-SDSDFQTVLQTVRTGDVNAKAS-QAGQTALMLAVSHGRGDMVRALLSCGAQVNVRDD
Fugu Kank2             TVSHSNFPVVKLLLD-TGLCNADKQNKAGYTAIMLTALAAFH-SDSDFQTVLQLLRTGDVNAKAS-QAGQTALMLAVSHGRGDMVRALLSCGAQVNIRDD
Medaka Kank2           TVSHSNFPVVKLLLD-TGLCNADKQNKAGYTAIMLTALAAFH-SDSDLQTVLQLLRTGDVNAKAS-QAGQTALMLAVSHGRGDMVRALLSCGAQVNIRDD
```

```
Sticklebakc Kank2     TVSHSNFPVVKLLLDTTGLCNADKQNKAGYTAIMLTALAAFH-SDNDLQTVLQLLRTGDVNAKAS-QAGQTALMLAVSHGRGDMVRALLSCGAQVNIRDD
Tilapia Kank2         TVSHSNFPVVKLLLD-TGLCNADKQNKAGYTAIMLTALAAFH-SDSDLQTVLQLLRTGDVNAKAS-QAGQTALMLAVSHGRGDMVRALLSCGAQVNIRDD
Amazon molly Kank2    TVSHSNFPVVKLLLD-TGLCNADKQNKAGYTAIMLTALAAFH-SDSDLQTVLQLLRTGDVNARAS-QAGQTALMLAVSHGRGDMVRALLSCGAQVNIRDD
Platyfish Kank2       TVSHSNFPVVKLLLD-TGLCNADKQNKAGYTAIMLTALAAFH-SDSDLQTVLQLLRTGDVNARAS-QAGQTALMLAVSHGRGDMVRALLSCGAQVNIRDD
Coelacanth KANK2      TVSHSNFPIVRLLLE-TGMCNVDKQNKAGYTAIMLTALAAFR-SEGDMETVTQLLHHGNVNAKAS-QAGQTALMLAVSHGRIDMVKALLAGGADVNLQDD
Anole lizard KANK2    TVSHSNFPVVAKLLE-TGLCHVDQQNKAGYTAIMLTALAASR-SESDMDTVVQLLKMGNVNAKAS-QAGQTALMLAVSHGRLDMVRALLASSADVNLQDD
Turtle KANK2          TVSHSNFPVVQRLLE-TGLCNVDKQNKAGYTAIMLTALAATR-SDDDMAIIRQLLQLGDVNAKAS-QAGQTALMLAVSHGRQDMVRALLACAADVNLQDA
Tetradon Kank1b       SVSHSNFGIVQKLLE-ADVCNMNHQNKAGYTPIMLAALAAVE-SPDDMKVVEQLFRKGDVNAKAS-QAGQTALMLAVSHGRVGMVRALLELGADVNLQDD
Medaka Kank1b         SLSHSNFEVVKKLLD-AGVCNVNLQNAAGYTPIMLAALSAVE-NPDHMRVVEKLFAAGNVNAKAS-QAGQTALMLAVSHGRRDMVETLLAQGADVNIRDD
Amazon molly Kank1b   SVSHSNFGVVKKLLD-AEVCNVNQQNAAGYTPVMLAALAAVD-RPDDMRVVEQLFSKGDVNAKAI-QAGQTALMLAVSHGRLDMVRALLAQGAEVNIQDD
Platyfish Kank1b      SVSHSNFGVVKKLLD-AEVCNVNQQNAAGYTPVMLAALAAVE-RPDDMRVVEQLFSTGDVNAKAI-QAGQTALMLAVSHGRLDMVRALLAQGAEVNIQDD
Frog Kank4            SVSHSNFSIVKLLLK-TGVCEVDYQNKAGYTPVMLTVLASTE-TDEDMEVVLTLLTEGNVNLCAT-QGRQTSLMLAVSHGRLDMVTVLLNCGADVNLQDE
Zebrafish Kank1b      SVSHSNFSVVKVLLA-AGVCNIDHQNKAGYTPIMLASLAAVE-TKEDMIVVQELFSRGDVNAKAS-QAGQTALMLAVSHGRLDMVQALLAAGAEVNIQDD
Tilapia Kank1b        SVSHSNFSVVKVLLA-AGVCNIDHQNKAGYTPIMLASLAAVE-TKEDMIVVQELFSRGDVNAKAS-QAGQTALMLAVSHGRLDMVQALLAAGAEVNIQDD
Fugu Kank1b           SVSHSNFSVVKVLLA-AGVCNIDHQNKAGYTPIMLASLAAVE-TKEDMIVVQELFSRGDVNAKAS-QAGQTALMLAVSHGRLDMVQALLAAGAEVNIQDD
Cavefish Kank1b       SVSHSNFRVVQKLLD-ADVCNIDQQNKAGYTPIMLAALAAVE-TQEDMRVVEGLFSKGDVNAKAI-QAGQTALMLAVSHGHMDMVKVLLASGSTVNVQDD
Spotted gar Kank4     SVSHSNFRIVKLLLD-TGLCKVDHQNKAGYTAIMLASLTAAD-SREDMDVALQLLKQGDINTRAS-QAGQTALMLAVSHGRIDMVRLLLSCNADVNAQDD
Coelacanth KANK4      SVSHSNFQIVKLLLD-TGVCDVDCQNKAGYTAVMLAPLAATE-TDEDMEVVLQLLKQGNVNIQTS-QGGQTALMLSVSHGRFHMVKALLSCGADLNLQDD
Frog Kank1            SVSHSNFDIVKLLLD-ADVCNVNQQNKAGYTPIMLAALAAVE-APKDMGVVEELFGSGDVNAKAS-QAGQTALMLAVSHGRIDMVRALLACGADVNIQDD
Shark Kank1           SVSHSNFEIVKMLLD-ANVCNVNQQNKAGYTPIMLAALAAVE-TEKDMKIVDELFSQGDVNAKAS-QAGQTALMLAVSHGRMDMVKALLLCAADVNIQDD
Coelacanth KANK1      SVSHSNFEIVKLLLD-AG----------------------------------------------------------------------------------
Mouse KANK1           SVSHSNFQIVKLLLD-ADVCNVDHQNKAGYTPIMLAALAAVE-AEKDMQVVEELFSCGDVNAKAS-QAGQTALMLAVSHGRIDMVKGLLACGADVNIQDD
Rat KANK1             SVSHSNFQIVKLLLD-ADVCNVDHQNKAGYTPIMLAALAAVE-AEKDMQVVEELFSCGDVNAKAS-QAGQTALMLAVSHGRIDMVKGLLACGADVNIQDD
Dog KANK1             SVSHSNFEIVKLLLD-ADVCNVDHQNKAGYTPIMLAALAAVE-AQKDMRVVEELFGCGDVNAKAS-QAGQTALMLAVSHGRIDMVKGLLACGADVNIQDD
Human KANK1           SVSHSNFEIVKLLLD-ADVCNVDHQNKAGYTPIMLAALAAVE-AEKDMRIVEELFGCGDVNAKAS-QAGQTALMLAVSHGRIDMVKGLLACGADVNIQDD
Anole lizard KANK1    SVSHSNFEIVKLLLD-ANVCNVNHQNKAGYTPIMLAALAAVD-AEKDMRIVEELFACGDVNAKAS-QAGQTALMLAVSHGRIDMVKALLACRADVNIQDD
Eagle KANK1           SVSHSNFEIVKLLLD-ANVCNVNHQNKAGYTPIMLAALAAVE-AEKDMRIVEELFSCGDVNAKAS-QAGQTALMLAVSHGRIDMVKALLACGADVNIQDD
Chicken KANK1         SVSHSNFEIVKLLLD-ANVCNVNHQNKAGYTPIMLAALAAVE-AEKDMRIVEELFSCGDVNAKAS-QAGQTALMLAVSHGRIDMVKALLACGADVNIQDD
Goose KANK1           SVSHSNFEIVKLLLD-ANVCNVNHQNKAGYTPIMLAALAAVE-AEKDMRIVEELFSCGDVNAKAS-QAGQTALMLAVSHGRIDMVKALLACGADVNIQDD
Turtle KANK1          SVSHSNFEIVKLLLD-ANVCNVNHQNKAGYTPIMLAALAAVE-AEKDMRIVEELFGCGDVNAKAS-QAGQTALMLAVSHGRIDMVKALLACGADVNIQDD
Aligator KANK1        SVSHSNFEIVKLLLD-ANVCNVNHQNKAGYTPIMLAALAAVE-AEKDMRIVEELFGCGDVNAKAS-QAGQTALMLAVSHGRIDMVKALLACGADVNIQDD
Spotted gar Kank1     SVSHSNFEIVKRLLD-ADVCNVNQQNKAGYTPIMLAALAAVE-AEKDMQIVEELFGKGDVNAKAS-QAGQTGLMLAVSHGRMDMVKALLACRADVNIQDD
Herring Kank1         SVSHSNFDIVKRLLD-ADVCNVNQQNKAGYTPIMLAALAAVE-SPKDMHIVEELFSKGDVNAKAS-QAGQTALMLAVSHGRMDMVKALLACGADVNIQDD
Zebrafish Kank1a      SVSHSNFHIVKKLLE-ADVCNVNQQNKAGYTPIMLAALAAVE-APKDMRVVDELFSKGDVNAKAS-QAGQTGLMLAVSHGRMDMVKALLACGADVNIQDD
Cave fish Kank1a      SVSHSNFHIVKKLLD-ANVCNVNQQNKAGYTPIMLAALAAVE-APKDMRVVEELFSKGDVNSKAS-QAGQTGLMLAVSHGRMDMVKALLACGADVNIQDD
Stickleback Kank1a    SVSHSNFLVVKELLD-AGEGGRGGKGAAGHTPIMLAALAAVE-SPKDMRIVEELFSKGDVNARAS-QAGQTGLMLAVSHGRMDMVRALLAQGADVNVQDD
Medaka Kank1a         SVSHSNFQVVKTLLD-ADVCNVNQQNKAGYTPIMLAALAAVE-TPKDMSVVEELFGKGDVNARAS-QAGQTGLMLAVSHGRMDMVRALLAHGADVNVQDD
Fugu Kank1a           SVSHSNFHVVKKLLD-ADVCNVNQQNKAGYTPIMLATLAAVD-TPKDMRTVQELFSKGDVNARAS-QAGQTGLMLAVSHGRMDMVQALLAHGADVNIQDD
Tetradon Kank1a       SVSHSNFHVVKKLLD-ADVCNVNQQNKAGYTPIMLATLAAVD-TPKDMRTVEELFSKGDVNARAS-QAGQTGLMLAVSHGRMDMVQALLAHGANVNIQDD
Tilapia Kank1a        SVSHSNFQVVKKLLD-ADVCNVNQQNKAGYTPIMLAALAAVE-TPRDMRIVEELFSKGDVNARAS-QAGQTGLMLAVSHGRMDMVRALLAHGADVNIQDD
Amazon molly Kank1a   SVSHSNFQVVKKLLD-ADVCNVNQQNKAGYTPIMLAALAAVE-TAKDMSIVEELFSKGDVNARAS-QAGQTGLMLAVSHGRMDMVRALLAHGADVNIQDD
Mouse KANK3           SVSHGNLAISSLLLD-TGVCDVNHQNRAGYSALMLAALTSVGQEEEDMAVAQRLFSMGDVNAKAS-QTGQTALMLAISHGHQDMVAALLECGADVNVQDA
Rat KANK3             SVSHGNLAISSLLLD-TGVCDVNHQNRAGYSALMLAALTSVGKEEEDMAVVQRLFSMGDVNAKAS-QTGQTALMLAISHGHQDMVAALLECGADVNVQDA
Dog KANK3             SVSHGNLAISSLLLD-TGVCDIDRQNRAGYSALMLAALTSVG-REEDMAVVQRLFHLGNVNAKAT-QTGQTALMLAISHGRQDMVAALLSCGADVNAQDA
Human KANK3           SVSHGNLAIASLLLD-TGACEVNRQNRAGYSALMLAALTSVRQEEEDMAVVQRLFCMGDVNAKAS-QTGQTALMLAISHGRQDMVATLLACGADVNAQDA
Lamprey               SVSHSNFAIVQMLLDTGGECNVDAQNKAGYTAIMLASLAAIN-SERDMAVVQKLLSLGDVNARASQQAGQTALMLAVSHGRKDMVRALLACGADVNVQDD
Coelacanth KANK3      SVSHSSFHIVKLLLE-TGVCNVDRQNKAGYTAIMLAALAAVD-IDEDRNVVRKLFSLGNVNAKAS-QAGQTALMLAVSHGRIEMVRALLDCGADVNIQDD
Frog Kank3            SVSHSNFFIVQLLLD-TGVCNVDRQNKAGYTAIMLAALAAVE-KEDDMNVVSRLFSLGNVNAKAS-QAGQTALMLAVSHGRQEMVQALLACGANVNLQDD
Turtle KANK3          SVSHSNFQIVQLLLD-TGVCNVDHQNKAGYTALMLAALASVE-HEDDMNVVRRLFSMGNVNAKAS-QAGQTALMLAVSHGRQEMVEAVLACGADVNLQDE
Aligator KANK3        SVSHSNFQIVRLLLD-TGVCNVDHQNKAGYTALMLAALAAVE-HEEDMNVVRRLFSMGNVNAKAS-QAGQTALMLAVSHGRQEMVEALLACGADVNLQDE
Eagle KANK3           SVSHSNFHIVRLLLD-TGVCNVDHQNKAGYTALMLAALAAVE-QEDDMNVVRRLFSMGNVNAKAS-QAGQTALMLAVSHGRQEMVEALLACGADVNLQDE
Chicken KANK3         SVSHSNFHIVKLLLD-TGVCNVDHQNKAGYTALMLAALAAVE-QEDDMNVVRRLFSMGNVNAKAS-QAGQTALMLAVSHGRQEMVEALLACGADVNLQDE
Goose KANK3           SVSHSNFHIVQLLLD-TGVCNVDHQNKAGYTALMLAALAAVE-QEDDMNVVRRLFSMGNVNAKAS-QAGQTALMLAVSHGRQEMVEALLACGADVNLQDE
Spotted gar Kank3     SVSHSNFTVVGLLLD-TGVCNVDHQNKAGYTAIMLAALAVVK-EEEDMRVVRKLFSLGSVNAKAS-QFSQTIQVLAVKMGSIDDL-FLLSTVNLVSFHKD
Zebrafish Kank3       SVSHSNFAVVDLLLD-TGMCNVNQQNKAGYTAVMLAALSAVK-EEDEMAVVQKLFRLGNVNAKAS-QAGQTALMLAVSHGRQEMVRALLDCGANVNIQDD
Cavefish Kank3        SVSHSNFTVVGLLLD-TGKCNVDQQNKAGYTAVMLAALSAVK-EEEDMVVVKKLFSQGNVNAKAS-QAGQTALMLAVSHGRQEMVRALLECGASVNIQDD
Herring Kank3         SVSHSNFSVVGLLLD-TGVCCVNQQNKAGYTAIMLAALSAVK-EEDDMVVVKKLFSQGNVNAKAS-QAGQTALMLAVSHGRQEMVRALLECGANVNVQDD
```

```
Stickleback Kank3     SVSHSNFSVVGLLLD-TGLCRVDKQNKAGYTAIMLAALSTVK-EEKDMAVVKRLFSQGNVNAKAS-QAGQTALMLAVSHGRQEMVRALLECGADVNVQDD
Fugu Kank3            SVSHSNFGVVELLLD-TGVCSVDKPNKAGYTAIMLAALSTVKEEEEDMVVVKKLFCQGNVNAKAS-QAGQTALMLAVSHGRQEMVRALLECGADVNVQDD
Tetradon Kank3        SVSHSNFGVVELLLD-TGVCSVDKPNKAGYTAIMLAALSTVKEEEEDMGVVKKLFCQGNVNAKAS-QAGQTALMLAVSHGRQEMVRALLECGADVNVQDD
Medaka Kank3          SVSHSNFHVVRLLLD-TGVCCVDKQNKAGYTAIMLAALSTVK-EEEDMAVVRKLFSQGNVNAKAS-QAGQTALMLAVSHGRQEMVRALLECGADVNVQDD
Tilapia Kank3         SVSHSNFGVVELLLD-TGVCSVDKQNKAGYTAIMLAALSTVK-KEEDMVVVKKLFSQGNVNAKAS-QAGQTALMLAVSHGRQEMVRALLECGADVNVQDD
Amazon molly Kank3    SVSHSNFGVVRLLLD-TGVCCVDKQNKAGYTAIMLAALSTVK-EEEDMAVVKKLFGQGNVNAKAS-QAGQTALMLAVSHGRQEMVQALLECGADVNVQDD
Platyfish Kank3       SVSHSNFGVVQLLLD-TGVCCVDKQNKAGYTAIMLAALSTVK-EEEDMAVVKKLFGQGNVNAKAS-QAGQTALMLAVSHGRQEMVQALLECGADVNVQDD

                               2910      2920      2930      2940      2950      2960      2970      2980      2990      3000
                      ....|....|....|....|....|....|....|....|....|....|....|....|....|....|....|....|....|....|....|....|
Hydra                 DGATALMCACDHGNVNIVKTLLGN-PDCDVTIEDN-----------------------------------------------------------------
Trichoplax            DGSTALMTACEHGLKDIVKLLLAQ-PNINVTTLDY-----------DNYDALS-IAMGNNFKDCGILIYSYI----------------------------
Aplasia               DGSTALMCACEHGHMDIVSYLLSQ-PNIDANLMDN-----------ENSTALT-IAMEAGHKDVGVVLYKHL-NFSTK----ASSP--------------
Snail                 DGSTALMCACEHGHLEIVNFLLSQ-PNIDAKITDN-----------ENSTALS-IAMEAGHKDVGVVLYKHL-NFS-K----PTSP--------------
Fruitfly              DGSTALMCAAEHGRVDVIKHLLSH-PECDSLVTDV-----------DGSTAFK-IAWQAGHRDVGLLIYVHE-QML-R----SKLP--------------
Hookworm              EGSTALMCAAEHGHKDIVKLLLAQ-PGIDAALTDC-----------DSSTALS-IAVENGHRDIGVLIYAHL-NYS-R----------------------
C.elegance            DGSTALMCAAEHGHKELVKMLLAE-NLVNASLTDV-----------DNSTALS-IALENDHREIGVMIYAYL-NYG------------------------
Tunicate              DGSTPLMCAVEHGQIEIVKLLLNH-PMCDAAAKDS-----------DGSTAMN-IAMDAGHREIAILLYAQL-NFK-P------------------NS--
Spider                DGSTALMCAAEHGHLEIVKLLLSH-PECDPTIVDN-----------DECNALT-IAMEAGHKDIGLLIYTNM-NFS-R----GSSPYSTLKNRKRSTPRS
Lingula               DGSTALMCASEHGHSEIVRLLLSM-AECDASLADN-----------DGSTALS-IAMEAGHKDIGILLYARQ-NIK-S----SPGL----------AR--
Lancelet              DGSTALMCASEHGHLEIVKLLLAQ-PGCDATITDN-----------DDSTALS-IAMEAGHRDIGVLLYAQV-NMG-K----------------------
Medaka Kank4b         QGTTALMFAAEKGHTHVARLLLER-SQSDLTLTDK-----------RGRTALS-IALQGSHSDTAALLQAHA-----K----ARAL--------------
Amazon molly Kank4b   QGMTSLMFASERGHTHIARLLLER-SQCDLTLTDK-------------------VRLQPTAKSSSGLSQSTT----------------------------
Platyfish Kank4b      QGMTSLMFASERGHTHIARLLLER-SQCDLTLTDK-----------RGQTALS-IAKQGSHTDTAALLQAHA-----K----ARAL--------------
Stickleback Kank4b    QGTTALMFASERGHTHIARLLLER-SRCDLTLTDK-----------RGQTALS-IAMQGSHTDTAALLQAHA-----K----ARAL--------------
Fugu Kank4b           QGTTALMFASERGHTHVARLLLER-SQCDLSLTDK-----------RGRTALA-IAMQGSHTDTATLLQAHA-----K----VRAL--------------
Tetraon Kank4b        QGTTALMFASERGHTHVARLLLER-SHCDLSLTDK-------------------V----------------------R----------------------
Cave fish Kank4b      PPNPPKTC-----------------PH-NAPLAPD-----------------------------------------------------------------
Medaka Kank4a         EGSTALMCASEHGHTHIVRLLLET-GRCNASLTDK-----------NGRTALS-AAEEAFHQDIAELL---------R----------------------
Fugu Kank4a           EGSTALMCASEHGHTHIVGLLLET-GRCDLSLTDK-----------NGQTAQK-VAAGSSHQDIVDLLKAHA-----K----------------------
Tetradon Kank4a       EGSTALMCASEHGHAHIVGLLLET-GRCDLSLADK-----------NGQTAQK-VAEGASHQDIIDLLKAHA----------------------------
Stickleback Kank4a    EGSTALMCASEHGHTNIVRMLLET-GRCDSTLTDK-----------NGQTARS-VAEGAAHQEIRDLLKAHA-----E----------------------
Tilapia Kank4a        EGSTALMCASEHGHTHIARLLLET-GRCDTSLKDK-----------NGQTALL-VAEGASQQDIIDLLKANA-----E----------------------
Platyfish Kank4a      EGSTALMCASEHGHTHIACLLLET-GRCDIGLTDK-----------NGQTALS-VAEAAAHQDIIDLLKSHA----------------------------
Amazon Molly Kank4a   EGSTALMCASEHGHTHIACLLLET-GHCDIGLTDK-----------NGQTALS-VAEAAAHQDIIDLLKSHA----------------------------
Herring Kank4         DGSTALMCACEHGHAEITRMLLKHPPGCDTSLKDK-----------DGHTALS-VATQASHMEVIDLLKAHT-GTP------------------------
Zebradish Kank4       DGSTALMCACEHGHTEIAKILLER-PDCDLTLTDK-----------DGHTALS-VAMKASHTEIIDLLNARS----------DPGP--------------
Cave fish Kank4a      DGSTALMCASEHGHAEIAKLLLDV-PECDTSLEDK-----------AGHTALS-VAIKASHPEIVDLLKAHA----------------------------
Mouse KANK4           DGSSALMLACHQGNADLVRLLLAH-PACNSSLTDK-----------AGRTALSLVLNSPAHVEIAELLRAHS-EPG------------------------
Rat KANK4             DGSSALMLACHQGNADLVRLLLAH-ADCNSNLTDK-----------AGQTALSIVLNSPAHVEIAELLRAHS-EQG------------------------
Dog KANK4             EGSSALMLACRHGNADMVRLLLAH-PACDSSLTDK-----------AGRTALSIVLKSPAHVEIAGLLRAHA-EQG-R----SLGP--------------
Human KANK4           DGSSALMVACHHGNVDLVRLLLAH-PACDSSLTDK-----------AGRTALSIALKSPTHMEIAGLLRAHA-EQG-R----SLGL--------------
Eagle KANK4           EGTTALMVACRQGNADIVRLLLAQ-PGCQVALTDK-----------GGNSALS----LARRGDIAALLRAHA----------------------------
Chicken KANK4         QGSTALMVACRQGSTDMVRLLLAQ-PGCQLALTDK-----------DGDTALS-IAQRSAHEDIAALLHSAQ-----H----GPND--------------
Goose KANK4           HGTTALMVACRQGNADMVRLLLAQ-PGCQLALTDKLEPTGKRHPSACTEPGRSARSSDPGQVPAGITQAQGDTVMSGQ----TAAP--------------
Anole lizard KANK4    TGLSPLMVASQYGNMDIVKLLLDH-PGCDTTLNDK-----------AGNSALS-MALKSAHVEIAEILQSHK-QHS-W----PLNT--------------
Turtle KANK4          NGTSALMVACQHGNAEIAKLLLAH-PGCNTTLTDK-----------SGNSALS-VALKSAHMEIAELLQAHK-EQS-R----SLAK--------------
Aligator KANK4        NGVSALMVACQQGNAEIVKLFLAH-PDCNATLTDK-----------DGNSALS-VALKSAHVEIAELLQAHI-EQS-S----SLSK--------------
Frog Kank2            DGSTALMCACEHGHADIVSLLLAV-PACDVSLTDN-----------DGSTALS-IALEAGQNDIAMLLSAHN-SKI-S----NQDE----------GT--
Mouse KANK2           DGSTALMCACEHGHKEITGLLLAV-PSCDISLTDR-----------DGSTALM-VALDAGQSEIASMLYSRM-NIK-C----SFAP--------------
Rat KANK2             DGSTALMCACEHGHKEITGLLLAV-PSCDISLTDR-----------DGSTALM-VALDAGQSEIASMLYSRM-NIK-C----SFAP--------------
Dog KANK2             DGSTALMCACEHGHKEITGLLLAV-PSCDISLTDR-----------DGSTALM-VALDAGQSEIASMLYSRM-NIK-C----SFAP--------------
Human KANK2           DGSTALMCACEHGHKEIAGLLLAV-PSCDISLTDR-----------DGSTALM-VALDAGQSEIASMLYSRM-NIK-C----SFAP--------------
Aligator KANK2        DGSTALMCACEHGHAAIAHLLLAA-PACRVDLADN-----------DGSTALS-IAQEAGQTDIVNMIEACL-GAP-A----LGNP----------LP--
Spotted gar Kank2     DGSTALMCACEHGHVDIVKQLLAV-PGCDATLTDN-----------DGSTALS-IAMEANQNDIAVLLYAHL-NFA-K----PPSP----------VQVL
Cave fish Kank2       DGSTALMCACEHGHVDIVRQLLSV-PGCDATLTDN-----------DGSTALS-IALEASQNDIAVLLYAHL-NFA-K----PPSP----------VS--
Herring Kank2         DGSTALMCACEHGHVDIVRQLLSV-PGCDATLTDN-----------DGSTALS-IALEASQNDIAVLLYAHL-NFA-K----PPSP----------VS--
```

```
Zebrafish Kank2       DGSTALMCACEHGHVDIVRQLLSV-PGCDATLTDN-----------DGSTALS-IALEASQNDIAVLLYAHL-NFA-K----PPSP----------VS--
Tetradon Kank2        DGSTALMCACEHGHVDIVRQLLSV-PGCDASLTDN-----------DGSTALS-IALEASQNDIAVLLYAHL-NFA-K----PPSP--------------
Fugu Kank2            DGSTALMCACEHGHVDIVRQLLSV-PGCDATLTDN-----------DGSTALS-IALEASQNDIAVLLYAHL-NFA-K----PPSP--------------
Medaka Kank2          DGSTALMCACEHGHVDIVRQLLSV-PGCDATLTDN-----------DGSTALS-IALEASQNDIAVLLYAHL-NFA-K----PPSP--------------
Sticklebakc Kank2     DGSTALMCACEHGHVDIVRQLLSV-PGCDATLTDN-----------DGSTALS-IALEASQNDIAVLLYAQL-NFA-K----PPSP--------------
Tilapia Kank2         DGSTALMCACEHGHVDIVRQLLSV-PGCDATLTDN-----------DGSTALS-IALEASQNDIAVLLYAHL-NFA-K----PPSP--------------
Amazon molly Kank2    DGSTALMCACEHGHVDIVRQLLSV-PGCDATLTDN-----------DGSTALS-IALEASQNDIAVLLYAHL-NFA-K----PPSP--------------
Platyfish Kank2       DGSTALMCACEHGHVDIVRQLLSV-PGCDATLTDN-----------DGSTALS-IALEASQNDIAVLLYAHL-NFA-K----PPSPVS------------
Coelacanth KANK2      DGSTALMCACEHGHVEIVRLLLAV-PGCDVTLTDN-----------DGSTALS-VALEANQNEIAMLLHSHM-NLT-K----ALAPV-------------
Anole lizard KANK2    DGSTALMCACEHGHAEIVRLLLAT-PGCDVALSDN-----------DGSTALS-IAVEAGQNDIAIMLYAQL-NFA-K----STSP----------GT--
Turtle KANK2          DGSTALMCACEHGHAEIARLLLAT-PACHVGLADH-----------DGSTALS-IALEAGQNDIAVMLYAHL-NFA-K----PPSP----------GT--
Tetradon Kank1b       EGSTALMCAGEHGHADMVRLLLAQ-AHCDATLTDS-----------DGSTALS-IALEAGHND-AVLLYAHT-NYS-R----------------------
Medaka Kank1b         EGSTALMCASEHGYADIVKLLLAQ-PECDATLTDS-----------DESTALS-IALEAGHSDIAVLLYAHT-TFS-R----QTG-----------AG--
Amazon molly Kank1b   EGSTALMCASEHGHADIVKLLLAQ-PDCDATLTDS-----------DDSSALS-VALEAGHNDIAVLLYAHA-NFS-R----CTA-----------GA--
Platyfish Kank1b      EGSTALMCASEHGHADIVKLLLAQ-PDCDATLTDS-----------DDSSALS-VALEAGHNDIAVLLYAHA-NFS-R----CTV-----------GA--
Frog Kank4            DGETALMMACRLGNMDLVKLLLAQ-PGCDTELSDK-----------VGNTALS-IVLNSAHSEIAEFLQAHT-EHR-R----------------------
Zebrafish Kank1b      EGSSALMCAGEHGHADIVKLLLAQ-PGCDATLTDN-----------DESTALS-IALEAGHKDIAMLIYAHV-NYS-K----SQPRSQ--------GS--
Tilapia Kank1b        EGSSALMCAGEHGHADIVKLLLAQ-PGCDATLTDN-----------DESTALS-IALEAGHKDIAMLIYAHV-NYS-K----SQPRSQ--------GS--
Fugu Kank1b           EGSSALMCAGEHGHADIVKLLLAQ-PGCDATLTDN-----------DESTALS-IALEAGHKDIAMLIYAHV-NYS-K----SQPRSQ--------GS--
Cavefish Kank1b       EGSTALMCASEHGHADIVKLLLAQ-PGCDATLSDN-----------DDSTALS-IALEAGHKDIAMLLYAHV-NYA-K----GQ--AM--------GT--
Spotted gar Kank4     DGSTALMCACEHGHADIVRLLLAL-PACDASITDN-----------EGNSALS-IALEASHTEIVDLLKAHT----------------------------
Coelacanth KANK4      TGSTALMCACEQGNVEIVKLLLTQ-PSCCVELTDK-----------VGRNAFS-IALEVSHKGIAELLNAHV-SHC------------------------
Frog Kank1            EGSTALMCASEHGHVEIVKLLLAQ-HGCNAALEDN-----------DGSSALS-IALDAGHKDIAVILYAHL-NFS-K----TPSP----------GT--
Shark Kank1           EGSTALMCASEHGHAEIVKLLLAQ-PGCDATLTDN-----------DESTALS-IALDAGHKDIAVLLYAHV-NFS-K----AQSP----------GT--
Coelacanth KANK1     ----------------------------------------------------------------------------------------------------
Mouse KANK1           EGSTALMCASEHGHVEIVKLLLAQ-PGCNGHLEDN-----------DGSTALS-IALEAGHKDIAVLLYAHL-NFS-K----AQSP----------ST--
Rat KANK1             EGSTALMCASEHGHVEIVKLLLAQ-PGCSGHLEDN-----------DGSTALS-IALEAGHKDIAVLLYAHL-NFS-K----AQSP----------ST--
Dog KANK1             EGSTALMCASEHGHVEIVKLLLAQ-PGCNGHLEDN-----------DGSTALS-IALEAGHKDIAVLLYAHV-NFA-K----APSP----------GT--
Human KANK1           EGSTALMCASEHGHVEIVKLLLAQ-PGCNGHLEDN-----------DGSTALS-IALEAGHKDIAVLLYAHV-NFA-K----AQSP----------GT--
Anole lizard KANK1    EGSTALMCASEHGHVEIVKLLLAQ-SGCNGTLEDN-----------DGSTALS-IALEAGHKDIAVLLYAHA-NFS-K----TQSP----------GT--
Eagle KANK1           EGSTALMCASEHGHVEIVKLLLAQ-PGCNGTLEDN-----------DGSTALS-IALEAGHKDIAVLLYAHV-NFS-K----TQSP----------GT--
Chicken KANK1         EGSTALMCASEHGHVEIVKLLLAQ-PGCNGTLEDN-----------DGSTALS-IALEAGHKDIAVLLYAHV-NFS-K----TQSP----------GT--
Goose KANK1           EGSTALMCASEHGHVEIVKLLLAQ-PGCNGTLEDN-----------DGSTALS-IALEAGHKDIAVLLYAHV-NFS-K----TQSP----------GT--
Turtle KANK1          EGSTALMCASEHGHVEIVKLLLAQ-SGCNGTLQDN-----------DGSTALS-IALEAGHKDIAVLLYAHV-NFS-K----PQSP----------ST--
Aligator KANK1        EGSTALMCASEHGHVEIVKLLLAQ-PGCNGTLEDN-----------DGSTALS-IALEAGHKDIAVLLYAHV-NFS-K----TQSP----------GT--
Spotted gar Kank1     EGSTALMCASEHGHVEIVKLLLAQ-PGCDATLTDG-----------DESTALS-IALEAGHKDIAVLLYAHV-NFS-K----AQSP----------GT--
Herring Kank1         EGSTALMCASEHGHVEIVKLLLAQ-SGCDATLSDS-----------DESNALS-IALEAGHKDIAVLLYAHV-NFS-K----AQSP----------GT--
Zebrafish Kank1a      EGSTALMCASEHGHVEIVKLLLAQ-PGCDPTLSDN-----------DESNALS-IALEAGHKDIAVLLYAHM-NFS-K----AQSPSVPVNLSVTGIL--
Cave fish Kank1a      EGSTALMCASEHGHVEIVKLLLAQ-PGCDATLSDN-----------DESTALS-IALEAGHKDIAVLLYAHV-NFS-K----AQSP----------GT--
Stickleback Kank1a    EGSTALMCASEHGHVEIVKLLLAR-AGCDATLSDS-----------DESNALS-IALEAGHKDIAMLLYAHV-NFS-K----AQSP----------GT--
Medaka Kank1a         EGSTALMCASEHGHVEIVKLLLAQ-PGCDATLSDG-----------DGSSALS-IALEAGHKDIAVLLYAHV-NFS-K----AQSP----------GT--
Fugu Kank1a           EGSTALMCASEHGHVEIVKLLLAQ-PGCDATLSDS-----------DESNALS-IALEAGHKDIAVLLYAHV-NFS-K----AQSP----------GT--
Tetradon Kank1a       EGSTALMCASEHGHVEIVKLLLAQ-PGCDATLSDS-----------DESNALS-IALEAGHKDIAVLLYAHV-NFS-K----AQSP----------GT--
Tilapia Kank1a        EGSTALMCASEHGHVEIVKLLLAQ-PGCDATLSDS-----------DESSALS-IALEAGHKDIAVLLYAHV-NFS-K----AQSP----------GT--
Amazon molly Kank1a   EGSTALMCASEHGHVEIVKLLLGQ-AGCNATLSDS-----------DGSNALS-IALEAGHKDIAVLLYAHV-NFS-K----AQSP----------GT--
Mouse KANK3           DGATALMCASEYGRLDTVQLLLAQ-PGCDLTILDN-----------EGTSALA-IALEAEQDEVAALLHAHL-----T----------------------
Rat KANK3             DGATALMCASEYGRLDTVQLLLAQ-PGCDLTILDN-----------EGTSALA-IALEAEQDEVAALLHAHL-TSNHHDSQEQSTP--------------
Dog KANK3             DGATALMCASEYGRLDTVRLLLAQ-PGCDPALLDN-----------EGTSALA-IALEAEQDEVAALLHAHL-TSG-QPDPLNQSP--------------
Human KANK3           DGATALMCASEYGRLDTVRLLLTQ-PGCDPAILDN-----------EGTSALA-IALEAEQDEVAALLHAHL-SSG-Q----PDTQ--------------
Lamprey               DGSTALMCASEHGHVDIVRLLLGQ-AGCNAAIADNLWTHLGCVCAQDGSTALS-IALEAGHNDIAVLLYAHV-NYA-K----------------------
Coelacanth KANK3      EGSTALMCASEHGRVEIVKLLLAQ-PGCDISILDN-----------DGSSALS-IALEANHNDIAVLLYAHM-NFS-K----AQSPQ---------AT--
Frog Kank3            EGSTALMCASEHGRAEIVKLLLAQ-TDCDVSITDN-----------DGSNALS-IALEAGQNDIAMLLYAHV-NFS-K----VQSP--------------
Turtle KANK3          EGSTALMCACEHGRLETVRLLLAQ-PACDVSIVDN-----------DGNNAVA-IALEAGHSNIAVLMYAHL-NNS-K----AQSP--------------
Aligator KANK3        EGSTALMCACEHGRVETVRLLLAQ-PVCDLSIVDN-----------DGNNAVA-IALEAGHSNIAVLMYAHL-NSS-K----AQSP--------------
Eagle KANK3           EGSTALMCACEHGRVETVKLLLAQ-PTCNISIVDS-----------DGNNAVA-IALEAGHSNIAVLLYAHL-NGT-K----AQLPQ---------GT--
Chicken KANK3         EGSTALMCACEHGRVETVKLLLAQ-PTCDVSIVDS-----------DGNNAVT-IALEAGHSNIAVLLYAHL-NGT-K----AQPQQ---------GT--
Goose KANK3           EGSTALMCACEHGRVETVKLLLAQ-PTCNISIVDS-----------DGNNAVT-IALEAGHSNIAVLLYAHL-NGT-K----AQPHQ---------GT--
```

```
Spotted gar Kank3     YKRQICRCNTNHKRTKKIHNVLCF-DFLQASFWNL-----------DGSNALS-IALEASHNDIAVLLYAHM-NYS-N----SQAA-----------R--
Zebrafish Kank3       EGSTALMCASEHGRAEIVSLLLEQ-PGCDISIVDN-----------DGSNALS-IALEASHNDIAVLLYAHM-NYS-K----TQADA---------S---
Cavefish Kank3        EGSTALMCASEHGRAEIVSLLLEQ-AGCDISIVDN-----------DGSNALS-IALEASHNDIAVLLYAHM-NYS-K----AQGEA---------QV--
Herring Kank3         EGSTALMCASEHGRAEIVAMLLDQ-LDCDISVVDN-----------DGSNALS-IALEASHNDIAVLLYAHM-NYS-K----SQAA----------GV--
Stickleback Kank3     EGSTALMCASEHGRAEIVKLLLEQ-PGCDISIVDN-----------DGSNALS-IALEASHNDTAVLLYAHM-NYA-R----TPPV----------GS--
Fugu Kank3            EGSTALMCACEHGRAEIVKLLLER-PGCDISIVDN-----------DGSNALS-IALEASHNDTAVLLYAHM-NYA-K----TQSAV-------------
Tetradon Kank3        EGSTALMCACEHGRAEIVQLLLEQ-PGCDISIVDN-----------DGSNALS-IALEASHNDTAVLLYAHM-NYA-K----NQSAG-------------
Medaka Kank3          EGSTALMCASEHGRAEIVKLLLEQ-PGCDISIVDN-----------DGSNALS-IALEAAHNDTAVLLYAHM-NYA-K----APSVA---------GS--
Tilapia Kank3         EGSTALMCASEHGRTEIVKLLLEQ-PGCDISIVDN-----------DGSNALS-IALEASHNDTAVLLYAHM-NYA-K----------------------
Amazon molly Kank3    EGSTALMCASEHGRAEIVKLLLEQ-PGCDISIVDN-----------DGSNALS-IALEAAHNDTAVLLYAHM-KYA-K----SQTTL---------GS--
Platyfish Kank3       EGSTALMCASEHGRAEIVKLLLEQ-PGCDISIVDN-----------DGSNALS-IALEAAHNDTAVLLYAHM-NYA-K----SQTTL---------GS--

                              3010      3020      3030      3040      3050      3060      3070      3080      3090
                      ....|....|....|....|....|....|....|....|....|....|....|....|....|....|....|....|....|....|....|....
Hydra                 ---------------------------------------------------------------------------------------------------
Trichoplax            ---------------SKTSKAHS----------------------------------------------------------------------------
Aplasia               -----------GVHRKRKSSSSPTPSSFELR--------------------------------------------------------------------
Snail                 -------RPARGYHKKRKSSSSPTPSSFEIHF-------------------------------------------------------------------
Fruitfly              -------NRGDATRSSLISPPRHKRQPSQ----------------------------------------------------------------------
Hookworm              ---------------AETIEDT-----------------------------------------------------------------------------
C.elegance            ---------------RLDISGPTSTI-------------------------------------------------------------------------
Tunicate              -------RSRPRVDGAKQKSSPVHRKTKS----------------------------------------------------------------------
Spider                TPPPRTPTLR-----TPPLPSPSARSTNSIPASFHFPS-------------------------------------------------------------
Lingula               -------SHKEGSMASRLPVSLSKK--------------------------------------------------------------------------
Lancelet              -------TAPGGGKGRRLSPSPTRRKSP-----------------------------------------------------------------------
Medaka Kank4b         ---------------------------------------------------------------------------------------------------
Amazon molly Kank4b   ---------------LSAVRPPDFNDNMPPVRLLD----------------------------------------------------------------
Platyfish Kank4b      ---------------------------------------------------------------------------------------------------
Stickleback Kank4b    ---------------------------------------------------------------------------------------------------
Fugu Kank4b           ---------------------------------------------------------------------------------------------------
Tetraon Kank4b        ---------------------------------------------------------------------------------------------------
Cave fish Kank4b      ---------------------------------------------------------------------------------------------------
Medaka Kank4a         ---------------------------------------------------------------------------------------------------
Fugu Kank4a           ---------------------------------------------------------------------------------------------------
Tetradon Kank4a       ---------------EASSTADL----------------------------------------------------------------------------
Stickleback Kank4a    ---------------TRGPESFFTT--------------------------------------------------------------------------
Tilapia Kank4a        ---------------VQTSESTSTADLL-----------------------------------------------------------------------
Platyfish Kank4a      -------------------SEPMSAADLL----------------------------------------------------------------------
Amazon Molly Kank4a   -------------------SEPMSAADLL----------------------------------------------------------------------
Herring Kank4         ---------------TLQAAAPH----------------------------------------------------------------------------
Zebradish Kank4       ---------------VTDSAAPL----------------------------------------------------------------------------
Cave fish Kank4a      ---------------EKAGSSQAAGLL------------------------------------------------------------------------
Mouse KANK4           ---------------RSLGPKELQKN-------------------------------------------------------------------------
Rat KANK4             ---------------RSLGLKELQKN-------------------------------------------------------------------------
Dog KANK4             ---------------------------------------------------------------------------------------------------
Human KANK4           ---------------------------------------------------------------------------------------------------
Eagle KANK4           ----------------EQSPSLSA---------------------------------------------------------------------------
Chicken KANK4         ---------------------------------------------------------------------------------------------------
Goose KANK4           ---------------CRASPRPGLLGAHLSPGGGVGWRGLVQPHPR-----------------------------------------------------
Anole lizard KANK4    ---------------------------------------------------------------------------------------------------
Turtle KANK4          ---------------------------------------------------------------------------------------------------
Aligator KANK4        ---------------------------------------------------------------------------------------------------
Frog Kank2            ------DPKR-----QRVNPSDESQ--------------------------------------------------------------------------
Mouse KANK2           ---------------MSDYESPASSSAEE----------------------------------------------------------------------
Rat KANK2             ---------------MSDYESPASSSAEE----------------------------------------------------------------------
Dog KANK2             ---------------MSDDESPASSSAEE----------------------------------------------------------------------
Human KANK2           ---------------MSDDESPTSSSAEE----------------------------------------------------------------------
```

```
Aligator KANK2        ------VTTE-----TAVTTEAPGDTQ------------------------------------------------------------------------
Spotted gar Kank2     QNNPQVRKTP-----RQLSPAPSPPIHQDGQTDGGRGASLEVGAGEAWLGLWDKGQQETGPGAESFFHFGFFRPANKPPRPPAALSAERPRGEREVLGS
Cave fish Kank2       ------PKSP-----ILGSPPSSSELK------------------------------------------------------------------------
Herring Kank2         ------PKSP-----LLGSSPPTGEMK------------------------------------------------------------------------
Zebrafish Kank2       ------PKSP-----ILGSSPPSSSELK-----------------------------------------------------------------------
Tetradon Kank2        ---------------QVSPKSPLLGSSPP----------------------------------------------------------------------
Fugu Kank2            ---------------VGKDQSPTSASAKK----------------------------------------------------------------------
Medaka Kank2          ---------------EKKNSAPKSDGFEQ----------------------------------------------------------------------
Sticklebakc Kank2     ---------------SSIQQSPQGKSATE----------------------------------------------------------------------
Tilapia Kank2         ---------------LSNKSLPRLSTHPH----------------------------------------------------------------------
Amazon molly Kank2    -------VSPKSPLLGSSPPSGEPK--------------------------------------------------------------------------
Platyfish Kank2       ---------------TELSWNVPGSGKLEN---------------------------------------------------------------------
Coelacanth KANK2      ---------------KETQPGPPPNAPAEAQQ-------------------------------------------------------------------
Anole lizard KANK2    ------PKQP-----KPDNTALTSSDNNTKDDGGTSH--------------------------------------------------------------
Turtle KANK2          ------PKQP-----KAETAAAVLPPEAQ----------------------------------------------------------------------
Tetradon Kank1b       ---------------------------------------------------------------------------------------------------
Medaka Kank1b         ------ARRS-----GRFSSSSGGRSVFE----------------------------------------------------------------------
Amazon molly Kank1b   ------ARHG-----GRFPSSSGGWSIFET---------------------------------------------------------------------
Platyfish Kank1b      ------ARHS-----GRFPSSSGGWSIFET---------------------------------------------------------------------
Frog Kank4            ---------------SRSAAEMEKGAPL-----------------------------------------------------------------------
Zebrafish Kank1b      ------PR-------SKTPPASRTSS-SD----------------------------------------------------------------------
Tilapia Kank1b        ------PR-------SKTPPASRTSS-SD----------------------------------------------------------------------
Fugu Kank1b           ------PR-------SKTPPASRTSS-SD----------------------------------------------------------------------
Cavefish Kank1b       ------PR-------SRTPPAAAARGFFE----------------------------------------------------------------------
Spotted gar Kank4     ---------------DHGTPDTS----------------------------------------------------------------------------
Coelacanth KANK4      ---------------------------------------------------------------------------------------------------
Frog Kank1            ------PRLS-----RKFPPSPTQKGPTDH---------------------------------------------------------------------
Shark Kank1           ------PRLG-----RKTSPSPTRRSKFE----------------------------------------------------------------------
Coelacanth KANK1      ---------------------------------------------------------------------------------------------------
Mouse KANK1           ------PRLG-----RKTSPGPTHRGSFD----------------------------------------------------------------------
Rat KANK1             ------PRLG-----RKTSPGPTHRGSFD----------------------------------------------------------------------
Dog KANK1             ------PRLG-----RKTSPAPTHRSSFD----------------------------------------------------------------------
Human KANK1           ------PRLG-----RKTSPGPTHRGSFD----------------------------------------------------------------------
Anole lizard KANK1    ------PRLG-----RKPSPGPAHRSPFDC---------------------------------------------------------------------
Eagle KANK1           ------PRLS-----RRTSPGPTHRATFE----------------------------------------------------------------------
Chicken KANK1         ------PRLS-----RKTSPGPTHRATFE----------------------------------------------------------------------
Goose KANK1           ------PRLS-----RKTSPGPTHRATFE----------------------------------------------------------------------
Turtle KANK1          ------PRLS-----RKTSPGPTRRAMFD----------------------------------------------------------------------
Aligator KANK1        ------PRLG-----RKTSPGPTHRGTFE----------------------------------------------------------------------
Spotted gar Kank1     ------PRLG-----RKTSPSPTRRSMFD----------------------------------------------------------------------
Herring Kank1         ------PRLS-----RKMSPSPTRKSAFD----------------------------------------------------------------------
Zebrafish Kank1a      ------CRQGVEYIINRIVPTNLFRPMVEDNVSSSVCMPA-----------------------------------------------------------
Cave fish Kank1a      ------PRLG-----RKTSPSPTRRGMFD----------------------------------------------------------------------
Stickleback Kank1a    ------PRLS-----RKTSPSPTQRGVFD----------------------------------------------------------------------
Medaka Kank1a         ------PRLS-----RKASPSPTRRSVFD----------------------------------------------------------------------
Fugu Kank1a           ------PRLG-----RKMSPSPTRRSMFD----------------------------------------------------------------------
Tetradon Kank1a       ------PRLG-----RKMSPSPTRRGMFD----------------------------------------------------------------------
Tilapia Kank1a        ------PRLG-----RKTSPSPTRRGMFD----------------------------------------------------------------------
Amazon molly Kank1a   ------PRLG-----RKTSPSPTRRGLFD----------------------------------------------------------------------
Mouse KANK3           -------SNH---QGQSSTGSPTAKECNDK---------------------------------------------------------------------
Rat KANK3             -------GSPTATLQRDTGPQPL----------------------------------------------------------------------------
Dog KANK3             ---------------LESTPDVPDEGECSDHREDPQPQ-------------------------------------------------------------
Human KANK3           -------AGVQRHNLSSLQPPPPRFKKFSCLSLPSSWDYNSCEPSRLAQLTIF----------------------------------------------
Lamprey               ------------------PPSPVSTSG------------------------------------------------------------------------
Coelacanth KANK3      ------PKMG-----LKSAANTAKKSVVE----------------------------------------------------------------------
Frog Kank3            -----------SSDVLAMGKASSSHGKPE----------------------------------------------------------------------
Turtle KANK3          ---------------QETPPIALKSSSNLMKKH------------------------------------------------------------------
```

```
Aligator KANK3        ---------------QGKSPTIPRKQH-------------------------------------------------------------------------
Eagle KANK3           ------SLTA-----MKSPGSPKKPN--------------------------------------------------------------------------
Chicken KANK3         ------STAG-----TKSPGSPKKPN--------------------------------------------------------------------------
Goose KANK3           ------STAG-----TKSPGNPKKLN--------------------------------------------------------------------------
Spotted gar Kank3     ------SLLA-----SRSDSSRVRTSPSL-----------------------------------------------------------------------
Zebrafish Kank3       ------PKAN-----SRSPSSPRKTWPSE-----------------------------------------------------------------------
Cavefish Kank3        ------PKAN-----PRSPSSPRKLWPSE-----------------------------------------------------------------------
Herring Kank3         ------NKGN-----TRSPTSPQKPWPAE-----------------------------------------------------------------------
Stickleback Kank3     ------PTVQ-----PRSPTSSHKSWPSD-----------------------------------------------------------------------
Fugu Kank3            ---------------VSPSSRPSLPGDKM-----------------------------------------------------------------------
Tetradon Kank3        ---------------VSPSSRPCQPG--------------------------------------------------------------------------
Medaka Kank3          ------PKSQ-----ARSPTSPHKSWPAE-----------------------------------------------------------------------
Tilapia Kank3         ---------------TQTAAETLLHTAIECNSE-------------------------------------------------------------------
Amazon molly Kank3    ------PKAQ-----PRSPTNPHKSWPAD-----------------------------------------------------------------------
Platyfish Kank3       ------PKAQ-----PRSPTNPHKSWPAD-----------------------------------------------------------------------
```
